# rs641738C>T near *MBOAT7* is associated with liver fat, ALT and fibrosis in NAFLD: A meta-analysis

**DOI:** 10.1016/j.jhep.2020.08.027

**Published:** 2021-01

**Authors:** Kevin Teo, Kushala W.M. Abeysekera, Leon Adams, Elmar Aigner, Quentin M. Anstee, Jesus M. Banales, Rajarshi Banerjee, Priyadarshi Basu, Thomas Berg, Pallav Bhatnagar, Stephan Buch, Ali Canbay, Sonia Caprio, Ankita Chatterjee, Yii-Der Ida Chen, Abhijit Chowdhury, Ann K. Daly, Christian Datz, Dana de Gracia Hahn, Johanna K. DiStefano, Jiawen Dong, Amedine Duret, EU-PNAFLD Investigators, Anita Vreugdenhil, Anna Alisi, Piotr Socha, Wojciech Jańczyk, Ulrich Baumann, Sanjay Rajwal, Indra van Mourik, Florence Lacaille, Myriam Dabbas, Deirdre A. Kelly, Valerio Nobili, Connor Emdin, Madison Fairey, Glenn S. Gerhard, GOLD Consortium, Gudny Eiriksdottir, Melissa E. Garcia, Vilmundur Gudnason, Tamara B. Harris, Lauren J. Kim, Lenore J. Launer, Michael A. Nalls, Albert V. Smith, Jeanne M. Clark, Ruben Hernaez, W.H. Linda Kao, Braxton D. Mitchell, Alan R. Shuldiner, Laura M. Yerges-Armstrong, Ingrid B. Borecki, J. Jeffrey Carr, Mary F. Feitosa, Jun Wu, Johannah L. Butler, Caroline S. Fox, Joel N. Hirschhorn, Udo Hoffmann, Shih-Jen Hwang, Joseph M. Massaro, Christopher J. O'Donnell, Cameron D. Palmer, Dushyant V. Sahani, Elizabeth K. Speliotes, Xiuqing Guo, Jochen Hampe, Matthew Hickman, Lena Heintz, Christian Hudert, Harriet Hunter, Matt Kelly, Julia Kozlitina, Marcin Krawczyk, Frank Lammert, Claudia Langenberg, Joel Lavine, Lin Li, Hong Kai Lim, Rohit Loomba, Panu K. Luukkonen, Phillip E. Melton, Trevor A. Mori, Nicholette D. Palmer, Constantinos A. Parisinos, Sreekumar G. Pillai, Faiza Qayyum, Matthias C. Reichert, Stefano Romeo, Jerome I. Rotter, Yu Ri Im, Nicola Santoro, Clemens Schafmayer, Elizabeth K. Speliotes, Stefan Stender, Felix Stickel, Christopher D. Still, Pavel Strnad, Kent D. Taylor, Anne Tybjærg-Hansen, Giuseppina Rosaria Umano, Mrudula Utukuri, Luca Valenti, Lynne E. Wagenknecht, Nicholas J. Wareham, Richard M. Watanabe, Julia Wattacheril, Hanieh Yaghootkar, Hannele Yki-Järvinen, Kendra A. Young, Jake P. Mann

**Affiliations:** 1 School of Clinical Medicine, University of Cambridge, Cambridge, UK; 2 MRC Integrative Epidemiology Unit (IEU), University of Bristol, Bristol, UK; 3 Medical School, Faculty of Health and Medical Sciences, University of Western Australia, Perth, WA, Australia; 4 Department of Hepatology, Sir Charles Gairdner Hospital, Perth, WA, Australia; 5 First Department of Medicine, Paracelsus Medical University Salzburg, Austria; 6 Translational and Clinical Research Institute, Faculty of Medical Sciences, Newcastle University, Newcastle upon Tyne, UK; 7 Newcastle NIHR Biomedical Research Centre, Newcastle upon Tyne Hospitals NHS Foundation Trust, Newcastle upon Tyne, UK; 8 Department on Liver and Gastrointestinal Diseases, Biodonostia Health Research Institute, Donostia University Hospital, University of the Basque Country (UPV/EHU), CIBERehd, Ikerbasque, San Sebastian, Spain; 9 Perspectum Ltd, Oxford, UK; 10 National Institute of Biomedical Genomics, Kalyani, India; 11 Division of Hepatology, Department of Medicine II, Leipzig University Medical Center, Leipzig, Germany; 12 Eli Lilly and Company, Indianapolis, IN, USA; 13 Medical Department 1, University Hospital Dresden, Technische Universität Dresden (TU Dresden), Dresden, Germany; 14 Gastroenterology, Hepatology and Infectiology, Otto-von-Guericke University Magdeburg, Magdeburg, Germany; 15 Yale University, Department of Pediatrics, New Haven, CT, USA; 16 The Institute for Translational Genomics and Population Sciences, Department of Pediatrics, The Lundquist Institute for Biomedical Innovation at Harbor-UCLA Medical Center, Torrance, CA, USA; 17 Institute of Post Graduate Medical Education and Research, Kolkata, India; 18 Department of Internal Medicine, General Hospital Oberndorf, Teaching Hospital of the Paracelsus Medical University Salzburg, Oberndorf, Austria; 19 Diabetes and Fibrotic Disease Unit Translational Genomics Research Institute (TGen), Phoenix, AZ, USA; 20 Program in Medical and Population Genetics, Broad Institute of Harvard and MIT, Boston, MA, USA; 21 Department of Medical Genetics and Molecular Biochemistry, Lewis Katz School of Medicine at Temple University, Philadelphia, PA, USA; 22 Department of Medicine II, Saarland University Medical Center, Saarland University, Homburg, Germany; 23 Department of Pediatric Gastroenterology, Charité - Universitätsmedizin Berlin, Berlin, Germany; 24 Eugene McDermott Center for Human Growth and Development, University of Texas Southwestern Medical Center, Dallas, TX, USA; 25 Laboratory of Metabolic Liver Diseases, Department of General, Transplant and Liver Surgery, Centre for Preclinical Research, Medical University of Warsaw, Warsaw, Poland; 26 MRC Epidemiology Unit, Institute of Metabolic Science, University of Cambridge, Cambridge, UK; 27 Department of Pediatrics, Columbia University, New York, NY, USA; 28 BioStat Solutions LLC, Frederick, MD, USA; 29 NAFLD Research Center, Division of Gastroenterology and Epidemiology, University of California at San Diego, La Jolla, CA, USA; 30 Minerva Foundation Institute for Medical Research, Helsinki, Finland; 31 Department of Medicine, University of Helsinki and Helsinki University Hospital, Helsinki, Finland; 32 Yale University School of Medicine, New Haven, CT, USA; 33 School of Global Population Health, Faculty of Health and Medical Sciences, The University of Western Australia, Perth, WA, Australia; 34 School of Pharmacy and Biomedical Sciences, Faculty of Health Sciences, Curtin University, Perth, WA, Australia; 35 Menzies Institute for Medical Research, College of Health and Medicine, University of Tasmania, Hobart, Australia; 36 Department of Biochemistry, Wake Forest School of Medicine, Winston-Salem, NC, USA; 37 Institute of Health Informatics, Faculty of Population Health Sciences, University College London, London, UK; 38 Department of Clinical Biochemistry, Rigshospitalet Copenhagen University Hospital, Copenhagen, Denmark; 39 Department of Molecular and Clinical Medicine, University of Gothenburg, Gothenburg, Sweden; 40 Cardiology Department, Sahlgrenska University Hospital, Gothenburg, Sweden; 41 Clinical Nutrition Unit, Department of Medical and Surgical Sciences, University Magna Graecia, Catanzaro, Italy; 42 Department of Medicine and Health Sciences ‘V. Tiberio’ University of Molise, Campobasso, Italy; 43 Department of Visceral and Thoracic Surgery, Kiel University, Kiel, Germany; 44 Division of Gastroenterology and Hepatology, Department of Medicine, University of Michigan Health System, Ann Arbor, MI, USA; 45 Department of Computational Medicine and Bioinformatics, University of Michigan Medical School, Ann Arbor, MI, USA; 46 Department of Gastroenterology and Hepatology, University Hospital of Zurich, Zurich, Switzerland; 47 Geisinger Obesity Institute, Danville, PA, USA; 48 Medical Clinic III, University Hospital RWTH Aachen, Aachen, Germany; 49 Department of the Woman, the Child, of General and Specialized Surgery, University of Campania Luigi Vanvitelli, Naples, Italy; 50 Department of Pathophysiology and Transplantation, Università degli Studi di Milano, Milan, Italy; 51 Translational Medicine, Department of Transfusion Medicine and Hematology, Fondazione IRCCS Ca’ Granda Ospedale Maggiore Policlinico Milano, Milan, Italy; 52 Division of Public Health Sciences, Wake Forest School of Medicine, Winston-Salem, NC, USA; 53 Department of Preventive Medicine, Keck School of Medicine, University of Southern California, Los Angeles, CA, USA; 54 Department of Medicine, Center for Liver Disease and Transplantation, Columbia University College of Physicians and Surgeons, New York Presbyterian Hospital, New York, NY, USA; 55 Genetics of Complex Traits, College of Medicine and Health, University of Exeter, Exeter, UK; 56 Department of Epidemiology, Colorado School of Public Health, University of Colorado Denver, Aurora, CO, USA

**Keywords:** *MBOAT7*, Fibrosis, NAFLD, Triglyceride, Diabetes, ALSPAC

## Abstract

**Background & Aims:**

A common genetic variant near *MBOAT7* (rs641738C>T) has been previously associated with hepatic fat and advanced histology in NAFLD; however, these findings have not been consistently replicated in the literature. We aimed to establish whether rs641738C>T is a risk factor across the spectrum of NAFLD and to characterise its role in the regulation of related metabolic phenotypes through a meta-analysis.

**Methods:**

We performed a meta-analysis of studies with data on the association between rs641738C>T genotype and liver fat, NAFLD histology, and serum alanine aminotransferase (ALT), lipids or insulin. These included directly genotyped studies and population-level data from genome-wide association studies (GWAS). We performed a random effects meta-analysis using recessive, additive and dominant genetic models.

**Results:**

Data from 1,066,175 participants (9,688 with liver biopsies) across 42 studies were included in the meta-analysis. rs641738C>T was associated with higher liver fat on CT/MRI (+0.03 standard deviations [95% CI 0.02–0.05], *p*_*z*_ = 4.8×10^–5^) and diagnosis of NAFLD (odds ratio [OR] 1.17 [95% CI 1.05–1.3], *p*_*z*_ = 0.003) in Caucasian adults. The variant was also positively associated with presence of advanced fibrosis (OR 1.22 [95% CI 1.03–1.45], *p*_*z*_ = 0.021) in Caucasian adults using a recessive model of inheritance (CC + CT *vs.* TT). Meta-analysis of data from previous GWAS found the variant to be associated with higher ALT (*p*_*z*_ = 0.002) and lower serum triglycerides (*p*_*z*_ = 1.5×10^–4^). rs641738C>T was not associated with fasting insulin and no effect was observed in children with NAFLD.

**Conclusions:**

Our study validates rs641738C>T near *MBOAT7* as a risk factor for the presence and severity of NAFLD in individuals of European descent.

**Lay summary:**

Fatty liver disease is a common condition where fat builds up in the liver, which can cause liver inflammation and scarring (including ‘cirrhosis’). It is closely linked to obesity and diabetes, but some genes are also thought to be important. We did this study to see whether one specific change (‘variant’) in one gene (*‘MBOAT7’*) was linked to fatty liver disease. We took data from over 40 published studies and found that this variant near *MBOAT7* is linked to more severe fatty liver disease. This means that drugs designed to work on *MBOAT7* could be useful for treating fatty liver disease.

## Highlights

• Meta-analysis of 42 studies (>1 million participants) to assess the role of rs641738C>T near *MBOAT7* in NAFLD.
• rs641738C>T positively associated with liver fat, ALT, fibrosis and HCC.
• rs641738C>T negatively associated with serum triglycerides.
• Consistent associations found in studies of Caucasian populations only.

## Graphical abstract

**Figure:**
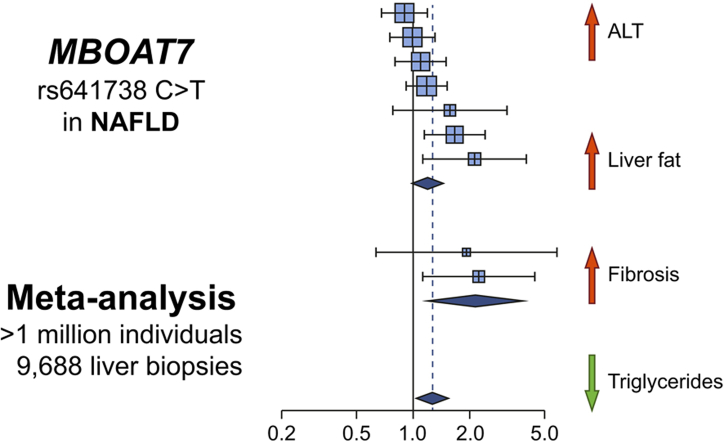

## Introduction

Since the first genome-wide association study (GWAS) of liver fat,^1^ >20 genetic single nucleotide variants (SNVs) have been associated with NAFLD.^2^ These studies have deepened our understanding of the condition, its heritability and its relationship with cardiometabolic disease.

rs641738C>T near membrane-bound *O*-acyltransferase domain-containing 7 (*MBOAT7*) was initially identified as a genome-wide significant risk variant for alcohol-related cirrhosis [odds ratio (OR) = 1.35, *p* = 1.03×10^–9^],^3^ although this was not replicated in a more recent analysis.^4^ It has since been implicated in the pathogenesis of NAFLD,^5^ HCC,^6^ as well as in fibrosis development in chronic HBV and HCV,^7^^,^^8^ and primary sclerosing cholangitis.^9^ However, unlike variants in patatin-like phospholipase domain containing protein 3 (*PNPLA3*), transmembrane 6 superfamily member 2 (*TM6SF2*), and 17β-hydroxysteroid dehydrogenase type 13 (*HSD17B13*), it was not identified to have genome-wide significance for liver fat or serum alanine aminotransferase (ALT).^1^^,^^10^^,^^11^

Rs641738 is located a few hundred base pairs downstream of the 3′-untranslated region of *MBOAT7*, which belongs to a family of genes that encode specific acyl donors and acceptors.^12^ *MBOAT7* encodes lysophosphatidylinositol acyltransferase 1 (LPIAT1), which contributes to the regulation of free arachidonic acid in cells.^13^^,^^14^ Rs641738C>T is associated with lower hepatic expression of *MBOAT7* at both the mRNA^15^ and protein levels.^5^ Given its role in inflammatory lipid pathways, most mechanistic work relating to rs641738 has focussed on *MBOAT7*.^16^

In NAFLD, the rs641738C>T variant was first demonstrated to be associated with increased hepatic fat content and severity of fibrosis in individuals of European descent.^5^ Proton magnetic resonance spectroscopy data from 2,736 individuals showed a modest increase in hepatic fat in those with the TT-genotype (4.1%) compared with those with the CT- (3.6%) or CC-genotype (3.5%, *p* = 0.005). Follow-up studies of European subjects corroborated the initial findings, and suggested a role in development of HCC.^17^^,^^18^ However, these results were not replicated in adults of other ancestries^5^^,^[19], [20], [21] or in children.^22^

In addition, bi-allelic loss-of-function mutations in *MBOAT7* cause autosomal recessive mental retardation 57 (Online Mendelian Inheritance in Man #617188) and no liver phenotype has been reported in these patients to date.^14^^,^^23^ However, rare likely pathogenic (coding) variants in *MBOAT7* are associated with HCC in NAFLD.^24^

In summary, the association between rs641738C>T and hepatic fat content, as well as its effects on severity of NAFLD, remain unclear. Moreover, the broader metabolic effects of this SNV, including its association with markers of insulin resistance and dyslipidaemia, have not been assessed. Understanding the broader metabolic effects of rs641738C>T is important if *MBOAT7* were to be investigated as a drug target in NAFLD.

Here, we conducted a large meta-analysis to determine whether rs641738C>T influences the development or stage of NAFLD and related traits.

## Methods

### Data sources and study selection

Two data sources were included in the meta-analysis: (i) studies that looked at the effect of the variant on traits of interest by genotyping the variant; and (ii) look-up from GWAS of traits of interest.

Studies were sourced through Medline, Embase, HuGe Navigator, Web of Science, bioRxiv and medRxiv. The search terms used were: ‘(*MBOAT7* or membrane-bound-o-acyltransferase) or (rs641738 or rs626283) or (*TMC4*)’. In addition, HuGe Navigator Phenopedia was searched using terms related to liver disease (see Supplementary Methods). There were no restrictions on either date or language. The search was completed on July 28, 2020. Reference lists of publications were also reviewed.

A separate search was conducted for all potentially relevant GWAS through GWAS Catalogue,^25^ Phenoscanner,^26^ Type 2 diabetes knowledge portal^27^ and Cardiovascular disease knowledge portal^28^ (see Supplementary Methods).

After removal of duplicates, titles and abstracts were screened for eligibility independently by 2 authors (investigators), with inclusion/exclusion criteria applied to potentially eligible full texts.

HuGENet guidelines^29^ were followed throughout and MOOSE reporting guidelines^30^ were used. This study was prospectively registered on the PROSPERO Database of Systematic Reviews (CRD42018105507; www.crd.york.ac.uk/PROSPERO/display_record.php?ID=CRD42018105507).

### Inclusion and exclusion criteria

Studies were included if genotyping of rs641738C>T [or rs626283G>C (R^2^ >0.98 in European and American populations^31^)/rs2576452C>T (R^2^ = 0.92 in Guzman *et al.*^32^), which are in strong linkage disequilibrium with rs641738C>T] was conducted and data on one of the outcomes of interest were reported. Narrative review articles, *in vitro* studies and investigations involving animals, fish and invertebrates were excluded. Studies that investigated liver disease of other aetiologies were also excluded. There was no restriction on ethnicity or ancestry. The types of study eligible for inclusion were case-control, cohort, GWAS, systematic reviews and meta-analyses. Preprint and abstract publications were not eligible for inclusion. Several studies reported on the same cohort (or patient sample) in more than 1 article. In these instances, data only from the larger of the overlapping cohorts were included in analyses. A full list of overlapping cohorts and articles is provided in Table S1.

### Data collection

Details of the recruitment of controls and cases were obtained from each study and, where necessary, clarified by discussion with the authors of the study. In particular, it was noted when cases and controls were not recruited from the same population or clinics.

Hepatic steatosis or NAFLD (as diagnosis) was evaluated as a dichotomous variable where radiological (liver ultrasound, controlled attenuation parameter [CAP, with cut-off >248 dB/m], CT, MRI, or histological assessment were used. Hepatic fat content was collected as a continuous variable from CT, magnetic resonance spectroscopy (MRS), MRI, and proton density fat fraction (PDFF). Non-invasive assessment of hepatic fat content was also assessed using semiquantitative scoring in the Fenland cohort, as previously described,^33^ and using CAP.

Individual participant-level histology data were extracted according to the NASH Clinical Research Network scoring system^34^ and, where not otherwise diagnosed by a pathologist’s assessment, NASH was defined using the Fatty Liver Inhibition of Progression algorithm.^35^ The above data were collected for each genotype separately (CC, CT, and TT).

Participant demographics and characteristics meta-data were collected from each study, including sex, age, ethnicity, presence of type 2 diabetes mellitus (T2DM), and body mass index (BMI). Where possible, individual patient-level data were obtained.

The authors of 59 studies were contacted for additional data or clarification, of whom 49 replied. Data from 11 potentially relevant studies could not be included, which are listed in the Supplementary Methods.

Additional details regarding cohorts with genome-wide data, the Avon Longitudinal Study of Parents and Children (ALSPAC)[36], [37], [38] data extracted from the UK BioBank (UKBB), quality assessment and statistical analysis are found in the Supplementary Methods.

## Results

Database searches identified 1,167 articles (Fig. S1), of which 44 articles were included: 42 primary studies (Tables S2–S4), 1 systematic review, and 1 meta-analysis (Table S5).

In total, 1,066,175 individuals (5,711 children) were included in the meta-analysis. Most studies were in adults (32/42, 76%) and in individuals from predominantly Caucasian populations (26/42, 62%). Of the 42 studies included, 14 (9,688 participants, including 584 children) reported data on liver histology.

Studies were generally of high quality, although, in 5 studies^11^^,^^22^^,^[39], [40], [41] (4 in adults and 1 in children), the control group was recruited from a different population or sample to the cases (Table S3).

One previous meta-analysis was included,^42^ which used data from 5 case-control studies to assess the effect of rs641738C>T on the diagnosis of NAFLD. The meta-analysis included 2,560 cases and 8,738 controls and found no evidence of an association between this variant and a diagnosis of NAFLD (Table S5). One previous systematic review^43^ found positive associations between rs641738C>T in adults of Caucasian, Hispanic, and Black descent, with limited data in children (Table S5).

### Liver fat, NAFLD and severe steatosis in adults

**Fig. 1:**
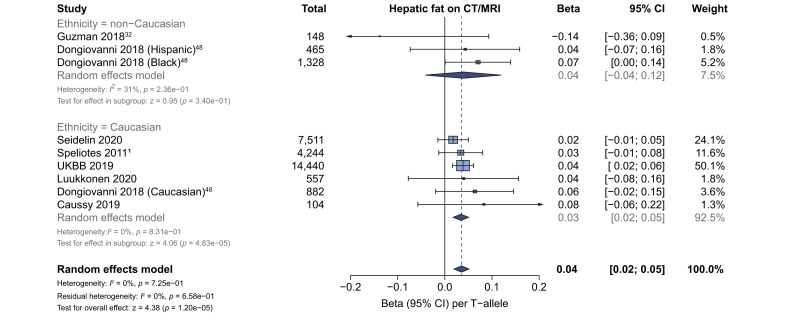
The effect of rs641738C>T on liver fat. Data from 29,679916 individuals with CT, MRI or MRS liver fat. rs641738C>T was positively associated with liver fat in Caucasian populations (using an additive model of inheritance), where data represent SD change in normalised liver fat per T-allele. Meta-analysis was performed using random effects with DerSimonian-Laird method for estimation of tau^2^. Additional references are available in the Supplementary Data. MRS, magnetic resonance spectroscopy; UKBB, UK BioBank.

Seven studies (29,679 participants) reported data on hepatic fat as a continuous variable assayed by CT or MRI. On meta-analysis, rs641738C>T was associated with higher liver fat in studies in Caucasian populations using an additive model of inheritance, with a per T-allele change of β 0.034 (95% CI 0.018–0.051), *p*_*z*_ = 4.8x10^–5^) standard deviations in inverse-normalised liver fat (Fig. 1), whereas no consistent effect was observed in non-Caucasian populations. A similar trend was observed using a dominant model of inheritance in studies of Caucasian populations: mean difference in hepatic fat +0.18% (95% CI 0.2–0.34; *p*_*z*_ = 0.04; Table S6).

Given the difference in sensitivity and specificity of modalities used to assess liver fat, a subanalysis by modality of imaging was performed. No significant differences were observed between studies using CT, MRI, or MRS for quantification of liver fat (Fig. S2).

A similar trend was observed using CAP and semiquantitative ultrasound to assess steatosis severity in 12,224 adults (β 0.02 [95% CI -0.002–0.04], *p*_*z*_ = 0.08; Fig. S3).

**Fig. 2:**
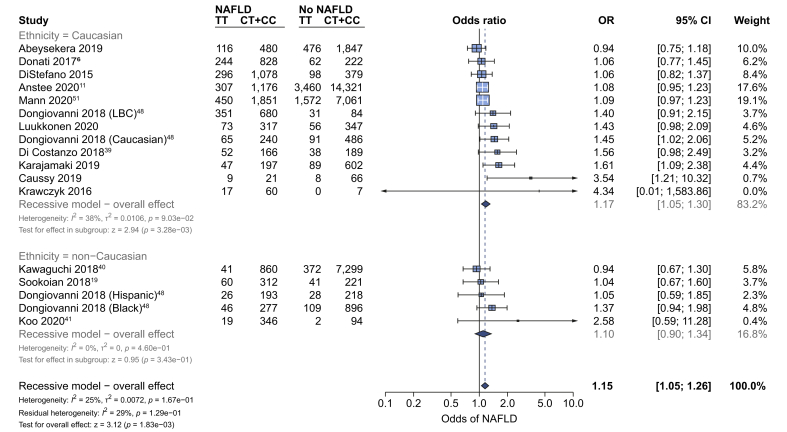
rs641738C>T is associated with higher odds of diagnosis of NAFLD. Data from 52,17333,263 adults (11,3019,713 cases and 40,87223,550 controls) with radiologically or histologically defined steatosis for presence *vs*. absence of NAFLD using a recessive model of inheritance (CC + CT *vs.* TT). Meta-analysis was performed using random effects with DerSimonian-Laird method for estimation of tau^2^. Additional references are available in the Supplementary Data. LBC, Liver Biopsy Cohort; OR, odds ratio.

Data from a range of diverse modalities were used to assess the effect of this variant on the diagnosis of NAFLD, to reflect real-world diagnostic practice. rs641738C>T was associated with NAFLD as a trait (OR 1.15 [95% CI 1.05–1.26], *p*_*z*_ = 0.002) using a recessive model of inheritance (Fig. 2) but not using additive or dominant models (Table S7). The effect was only observed in studies of Caucasian populations (OR 1.17 [95% CI 1.05–1.3], *p*_*z*_ = 0.003). Subgroup analysis by modality of diagnosis found that the 95% CIs for all modalities overlapped, except for MRI-PDFF, which had only 1 study (Fig. S4). The association remained after excluding 4 studies in which there was a lack of similarity between cases and controls (OR 1.19 [95% CI 1.07–1.33], *p*_*z*_ = 0.0017) using a recessive model of inheritance.

However, Egger's test suggested evidence of study distribution (publication) bias (*p* = 0.013) and when using the Trim and Fill method to account for this bias, the positive association remained but was attenuated (OR 1.11 [95% CI 1.01–1.23], *p*_*z*_ = 0.037; Fig. S5).

In patients with NAFLD, data from 8 studies (6,206 participants) showed that rs641738C>T was not significantly associated with the presence of severe steatosis (S1-S2 *vs.* S3) on liver biopsy (OR 1.08 [95% CI 0.78–1.5], *p*_z_ = 0.64; Table 1 and Fig. S6).

**Table 1:** Summary of results in adults from meta-analyses for dichotomous outcomes.

Outcome	Genetic model	Subanalysis	No. of studies	Heterogeneity		Effect summary	
				I^2^	*p*_*Q*_	OR (95% CI)	*p*_*Z*_
NAFLD diagnosis (control *vs.* NAFLD)	Recessive	Overall	17	0.25	0.17	1.15 (1.05–1.26)	0.0018
		Non-Caucasian	5	0	0.46	1.1 (0.9–1.34)	0.343
		Caucasian	12	0.38	0.09	1.17 (1.05–1.3)	0.0033
Severe steatosis (S1–S2 *vs.* S3)	Recessive	Overall	8	0.67	0	1.08 (0.78–1.5)	0.642
		Non-Caucasian	1	NA	NA	1.11 (0.39–3.16)	0.852
		Caucasian	7	0.72	0	1.08 (0.76–1.54)	0.676
NASH (NAFL *vs.* NASH)	Recessive	Overall	9	0.33	0.15	1.14 (0.96–1.36)	0.128
		Non-Caucasian	3	0	0.58	1.24 (0.81–1.9)	0.324
		Caucasian	6	0.53	0.06	1.14 (0.93–1.41)	0.213
Any fibrosis (F0 *vs.* F1–F4)	Recessive	Overall	9	0.52	0.03	1.27 (1.04–1.54)	0.0183
		Non-Caucasian	2	0	0.82	2.14 (1.2–3.84)	0.0105
		Caucasian	7	0.51	0.06	1.19 (0.99–1.45)	0.068
Advanced fibrosis (F0–F2 *vs.* F3–F4)	Recessive	Overall	8	0	0.65	1.2 (1.02–1.42)	0.027
		Non-Caucasian	2	0	0.64	0.96 (0.5–1.85)	0.911
		Caucasian	6	0	0.5	1.22 (1.03–1.45)	0.0206
HCC (NAFLD-HCC *vs.* NAFLD no-HCC)	Recessive	Overall	4	0	0.95	1.4 (0.99–1.98)	0.056

Meta-analyses were performed using random effects with subgroup analysis for Caucasian and non-Caucasian populations. Additive, recessive and dominant genetic models were tested for all outcomes. Results using a recessive model of inheritance (CC + CT *vs.* TT) are shown for all outcomes. Given the use of 3 genetic models, the critical *p* value for effect summary is *p*_*z*_ <0.017. Full results (with all genetic models) are in Table S5. Meta-analyses were performed using random effects with DerSimonian-Laird method for estimation of tau^2^. OR, odds ratio.

### Histological NASH in adults

Data from 9 studies (7,719 participants) found that rs641738C>T was not associated with the presence of NASH on biopsy in adults (OR 1.24 [95% 0.96–1.36], *p*_*z*_ = 0.128; Fig. S7).

### Fibrosis in adults

**Fig. 3:**
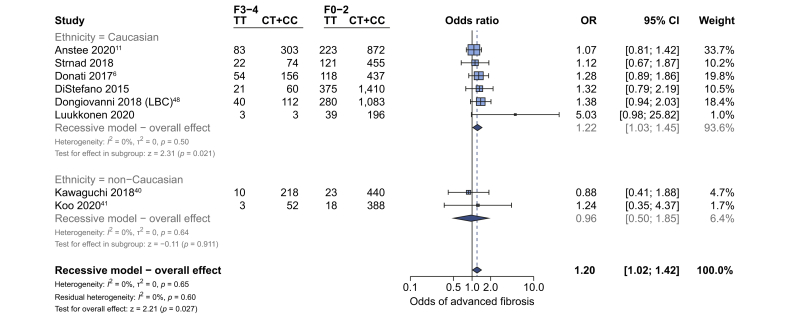
The effect of rs641738C>T on the presence of advanced fibrosis in adult patients with NAFLD. Data from 7,6926,211 adults (1,214828 cases and 6,4785,383 controls) with biopsy-proven NAFLD comparing advanced fibrosis (F3–F4) *vs*. F0–F2, using a recessive model of inheritance (CC + CT *vs.* TT). Meta-analysis was performed using random effects with DerSimonian-Laird method for estimation of tau^2^. Additional references are available in the Supplementary Data. LBC, Liver Biopsy Cohort; OR, odds ratio.

Liver biopsy data on the presence of advanced fibrosis were available from 8 studies (7,692 adults). Our primary outcome, presence of advanced fibrosis in adults (stage F0–F2 *vs*. stage F3–F4), showed a borderline positive association with rs641738C>T in Caucasian populations (OR 1.22 [95% 1.03–1.45], *p*_*z*_ = 0.021; Fig. 3). In addition, 2 studies used International Statistical Classification of Diseases and Related Health Problems (ICD) codes in the UKBB cohort to identify individuals with NAFLD and advanced fibrosis or cirrhosis.^44^^,^^45^ Both found positive associations below genome-wide significance: for example, using an additive model of inheritance, Emdin *et al.* found the association between rs641738C>T and cirrhosis as β 1.22 (SE 0.06, *p* = 0.03), using an additive genetic model.^44^

Data from 9 studies (8,389 participants) found that the presence of any fibrosis (F0 *vs*. F1–F4) was also borderline positively associated with rs641738C>T overall (OR 1.27 [95% 1.04–1.54], *p*_*z*_ = 0.018) as well as in non-Caucasian populations as a subgroup (Fig. S8).

### Development of HCC

**Fig. 4:**
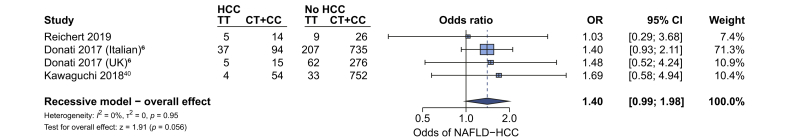
rs641738C>T is associated with higher odds of NAFLD-HCC. Data from 2,328 adults with NAFLD assessing for the presence *vs*. absence of HCC, using a recessive model of inheritance (CC + CT *vs.* CT). Meta-analsysis was performed using random effects with DerSimonian-Laird method for estimation of tau^2^.

Four cohorts (2,328 participants, 228 cases of NAFLD-HCC) reported on the development of HCC in patients with NAFLD. rs641738C>T was associated with increased odds of HCC in NAFLD only when using a dominant model (CC *vs.* CT + TT) of inheritance (OR 1.64 [95% CI 1.18–2.27], *p*_*z*_ = 0.003, Fig. 4).

### Effect on alanine aminotransferase

**Fig. 5:**
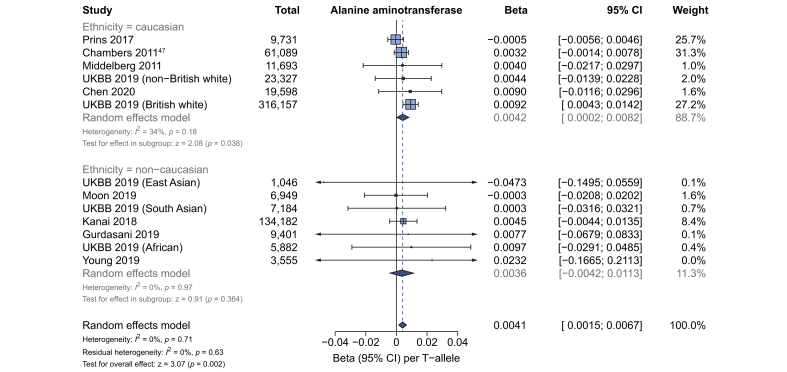
rs641738C>T is positively associated with ALT in Caucasian populations in GWAS. Meta-analysis using linear regression of GWAS summary statistics from 609,794 participants for the association between rs641738C>T and logarithmically transformed ALT. Meta-analysis was performed using random effects with DerSimonian-Laird method for estimation of tau^2^. Additional references are available in the Supplementary Data. GWAS, genome-wide association studies; UKBB, UK BioBank.

Data from GWAS using log-transformed ALT (609,794 participants) were available for meta-analysis to investigate the effect of rs641738C>T on ALT. The variant showed a positive association with ALT (β 0.004 [95% CI 0.002–0.007], *p*_*z*_ = 0.002), which was observed in Caucasian populations but not in non-Caucasian populations on subanalysis (Fig. 5; Table S7).

Additionally, in the UKBB cohort, rs641738C>T was associated with a small, but statistically significant (*p* = 2.0×10^–8^) increase in untransformed ALT: 0.18 IU/L higher ALT per T-allele in this variant (Table S8).

In the remaining cohort and case-control studies included in the meta-analysis (15,208 adults), rs641738C>T was not found to be significantly associated with a change in ALT; for example, the mean difference using a recessive model (CC + CT *vs.* TT) was +0.32 IU/L ([95% CI -0.06–0.7], *p*_*z*_ = 0.08; Table S9) in Caucasian populations.

### Effect on serum lipids and insulin

Data from GWAS using log-transformed serum triglycerides (850,241 participants) found that rs641738C>T was associated with lower triglycerides (β -0.01 [95% CI -0.018, -0.006], *p*_*z*_ = 1.5×10^–4^), which was observed in Caucasian populations but not in non-Caucasian populations on subanalysis (Fig. S9). Similar findings were obtained from a meta-analysis of cohort and case-control studies, particularly when using an additive model (β -0.03 [95% CI -0.05, -0.01], *p*_*z*_ = 0.00091; Table S9).

Data from GWAS (852,409 participants) found rs641738C>T to be positively associated with total cholesterol in Caucasian populations (β 0.007 [95% CI 0.003–0.01], *p*_*z*_ = 2.1×10^–4^), which was not observed in non-Caucasian populations (Fig. S10). A borderline positive association was also observed between rs641738C>T and HDL cholesterol (β 0.009 [95% CI 0.001–0.02], *p*_*z*_ = 0.02; Table S7). There was no effect on fasting insulin levels found in population-level GWAS (β 0.009 [95% CI -0.03–0.04], p_z_ = 0.64; Table S7). However, a negative association was observed using data from cohort and case-control studies with a dominant genetic model (mean difference -1.4 pmol/L [95% CI -2.1, -0.65], *p*_*z*_ = 0.004; Table S9).

### Effect of rs641738C>T on paediatric NAFLD

Data from 10 studies (5,711 children) were used in the meta-analysis. rs641738C>T was not significantly associated with the diagnosis of NAFLD, liver fat content, stage of liver histology, or serum biochemistry in children (Table S10).

### Meta-regression shows interaction between rs641738C>T and type 2 diabetes mellitus

Finally, we aimed to determine using meta-regression whether baseline participant characteristics influenced the association of rs641738C>T with histological outcomes. There was a negative association with the presence of T2DM and effect size for NASH *vs.* NAFL (β -1.8 [SE 0.65], *p* = 0.006; Fig. S11A). A similar negative trend with T2DM was observed for severe steatosis (S1–S2 *vs.* S3, β -2.6 [SE 1.5], *p* = 0.08) and presence of fibrosis (F0 *vs.* F1-4, β -1.5 [SE 0.8], *p* = 0.06; Table S11). In addition, the effect size for any fibrosis was greater in cohorts with an older mean age (β 0.05 [SE 0.02], *p* = 0.014; Fig. S11D).

### Discussion

Identification of genetic variants associated with NAFLD has the potential to inform preclinical research and our understanding of hepatic metabolism. In this meta-analysis, we validated rs641738C>T near *MBOAT7* as a risk factor for the full spectrum of NAFLD in Caucasian adults.

A 2-stage GWAS initially identified rs641738C>T as a genome-wide significant locus for alcohol-related cirrhosis.^3^ *MBOAT7* was a potentially interesting target as an enzyme involved in (phospho)lipid metabolism, conceptually similar to other SNVs at GWAS significance in alcoholic and non-alcoholic liver disease, namely *TM6SF2* and *PNPLA3*. Later studies found the variant to influence the full spectrum of fatty liver disease, from steatosis to NASH, to fibrosis, cirrhosis, and HCC.^5^^,^^17^ However, these associations have not been consistently replicated in the literature.^19^ We conducted a meta-analysis to firmly establish the association of rs641738C>T with the presence and severity of NAFLD and associated metabolic traits.

### Main findings

We found that rs641738C>T was associated with higher liver fat content, higher ALT, and with higher odds of NAFLD diagnosis, fibrosis, and HCC, particularly in Caucasian adults and in the homozygous ‘TT’ genotype. The effect sizes of rs641738C>T reported here are small compared with those of *PNPLA3* p.I148M and *TM6SF2* p.E167K, the 2 strongest steatogenic variants.^46^ Also, the magnitude of change in ALT is small relative to that associated with variants in *PNPLA3*, *HSD17B13*, mitochondrial amidoxime reducing component 1 (*MTARC1*), and *TM6SF2*. This might account for the absence of this variant (or others near *MBOAT7*) from GWAS for NAFLD in the general population.^1^^,^^10^^,^^11^^,^^45^^,^^47^ The effect size (and associated *p* value) was too small to be identified as significant genome-wide. The marginal positive effect on hepatic triglyceride content suggests that this variant acts through alterations in the composition as well as quantity of hepatic lipid.^17^ This is consistent with preclinical data on lipotoxicity, where the composition of hepatic fats influences the development of NASH. By contrast, a recent Mendelian randomisation study using these variables as instruments to assess causality of fatty liver in determining fibrosis showed that the effect of steatosis highly correlates with fibrosis in all the genetic variables, indicating that quantity of lipid rather than quality might be more important.^48^ Functional studies are needed to understand the relationship between quality/quantity of fat and hepatotoxic/-protective mechanisms in causing progression of disease.

The function of this variant is still relatively poorly understood and there is conflicting evidence as to whether rs641738C>T is associated with changes in the hepatic expression of *MBOAT7*. Results from the GTEx Consortium showed a strong negative association with T-allele,^15^ which is supported by data from Schadt *et al.*^49^ *MBOAT7* protein expression correlated with mRNA in liver biopsies from Mancina *et al.*,^5^ but this finding was not replicated by Sookoian *et al.*^19^ *MBOAT7* encodes LPIAT1, a 6-transmembrane domain protein involved in acyl-chain remodelling of membranes that influence intracellular membrane composition and circulating phosphatidylinositols.^50^ Furthermore, recent metabolite profiling data implicated *MBOAT7* as the causal gene for this SNV.^51^ Moreover, transmembrane channel-like 4 (TMC4) was found to have a low expression in the liver,^5^ which is consistent with no mechanistic data supporting its role in NAFLD.

The hypothesis that *MBOAT7* is the causal gene underlying the association with liver disease at the locus is supported by the observation that mice deficient for *MBOAT7* have altered hepatic concentrations of polyunsaturated phosphatidylinositol.^50^ Similarly, metabolite data from humans are strongly suggestive that rs641738C>T reduces MBOAT7 function.^52^ In addition, 2 independent groups found that loss of *MBOAT7* (but not *TMC4)* increases the severity of NAFLD in mice fed a high-fat diet.^53^^,^^54^

These analyses suggest that rs641738C>T impacts the severity of NAFLD through a recessive model of inheritance, although some analyses using an additive genetic model were suggestive of a role (*e.g.* for liver fat and ALT). Other genetic variants are known to impact all-cause mortality in a recessive manner, notably variants that perturb homeostatic iron regulator protein (*HFE*).^44^ Further mechanistic work is required to understand the extent to which the haplo-insufficient state affects hepatocyte function.

We found no evidence of an effect of rs641738C>T on insulin resistance (the key driver of hepatic steatosis) as determined by unaltered fasting insulin concentrations. GWAS meta-analyses of T2DM have implicated p.I148M in *PNPLA3* and p.E167K in *TM6SF2* as significant risk loci (albeit with very modest effect sizes compared with their effects on liver disease)^55^ and Mendelian randomisation studies indicate a causal role in determining insulin resistance mediated by the degree of liver damage.^48^^,^^56^ Similarly, these 2 variants are associated with reduced risk of coronary artery disease; although our analysis did find lower serum triglycerides to be associated with this variant, it has not been associated with lower rates of cardiovascular disease.^57^ However, we did observe a negative association between effect size and prevalence of diabetes on meta-regression, suggesting that this variant has the greatest effect in less insulin-resistant individuals.

A strength of this meta-analysis is the large number of individuals with liver biopsy-derived phenotypic data as well as the use of population-based GWAS data. The larger number of included studies and participants is likely to account for the different conclusions reached in this study compared with the previous meta-analysis by Xia *et al.*^42^

### Limitations and quality of evidence

An important practical consideration is the population frequency of this variant in different ethnicities. The mean allelic frequency of the effect (T) allele is highly variable: from 0.24 in East Asians compared with 0.53 in those of South Asian ancestry.^58^ Moreover, the majority of studies included in this meta-analysis used self-reported ethnicity, rather than genetic ancestry.

Although this analysis did include data from individuals of multiple ethnicities (and genetic ancestries), we only found evidence of an effect of this variant in Caucasian individuals. This is consistent with the initial discovery and it is likely that rs641738C>T is a proxy for the true causal variant. However, because of differences in patterns of linkage disequilibrium, we cannot exclude the possibility that a different nearby locus is associated with liver-related phenotypes in individuals of other genetic ancestries.

A limitation of using meta-analysis for a single variant is the lack of adjustment for population stratification. When further genome-wide data are available, a formal GWAS meta-analysis might be able to address this. We found significant differences between adult and paediatric histological analyses. Although there were fewer clinical events (*e.g.* with advanced fibrosis) in children, the analyses did not show a trend congruous with those in adults. Paediatric NAFLD has a different histological phenotype to that of adults (with prominent periportal inflammation) and, therefore, it is plausible that this is a true lack of association in children with NAFLD.

Data from multiple diagnostic or imaging modalities were combined in several analyses. Although we observed minimal heterogeneity between modalities, these techniques have differing accuracy for the diagnosis of steatosis, which has the potential to affect results. The subgroup analysis of hepatic fat by modality suggested a marginally greater effect size in studies using MRS, which is regarded as a highly sensitive technique. There is potential that, through the inclusion of other modalities (*e.g.* CT), we have underestimated the effect size associated with this variant.

The magnitude of effect observed across all associations was small compared with other well-established variants. The clinical relevance of rs738409C>G in *PNPLA3* has been validated with hard end-points,^59^ but large cohorts will be required to prospectively demonstrate the clinical risk associated with this variant near *MBOAT7*.

Although there was minimal heterogeneity across included studies, there was evidence of publication bias, but the effect on diagnosis of NAFLD appeared to persist after attempting to account for this. Also of note, the numbers of individuals with NAFLD and HCC were comparatively low, limiting the power to assess for an association of this variant with non-cirrhotic HCC, as has been previously reported.^6^ The HCC analysis was also unique in only demonstrating an effect in the dominant, rather than recessive, model of inheritance. Further work in this area might improve the accuracy of effect estimates.

### Conclusions

rs641738C>T near *MBOAT7* is positively associated with liver fat, ALT and histological severity in Caucasian adults with NAFLD, but negatively associated with serum triglycerides and with relatively small effect sizes throughout. These data validate this locus as significant in the pathogenesis of NAFLD.

### Abbreviations

ALSPAC, Avon Longitudinal Study of Parents and Children; BMI, body mass index; CAP, controlled attenuation parameter; GWAS, genome-wide association study; *HFE*, homeostatic iron regulator protein; HSD17B13, 17β-hydroxysteroid dehydrogenase type 13; LPIAT1, lysophosphatidylinositol acyltransferase 1; *MBOAT7*, membrane bound O-acyltransferase domain containing 7; *MTARC1*, mitochondrial amidoxime reducing component 1; OR, odds ratio; MRS, magnetic resonance spectroscopy; PDFF, proton density fat fraction; *PNPLA3*, patatin-like phospholipase domain containing protein 3; SNV, single nucleotide variant; T2DM, type 2 diabetes mellitus; *TM6SF2*, transmembrane 6 superfamily member 2; *TMC4*, transmembrane channel-like 4; UKBB, UK BioBank.

## Financial support

J.P.M. is supported by a 10.13039/100010269Wellcome Trust Fellowship (216329/Z/19/Z), a European Paediatric Research Society award and a 10.13039/501100000290Children's Liver Disease Foundation grant. The EU-PNAFLD is supported by an EASL Registry Grant. 10.13039/100000002NIH grants: R01HD028016 (S.C.), R01DK111038 (S.C)., R01DK114504 (N.S.), DK091601 (J.K.D.) and UL1TR001105 (J.K.). Supported by the Nonalcoholic Steatohepatitis Clinical Research Network (NASH CRN) of the 10.13039/100000062National Institute of Diabetes and Digestive and Kidney Diseases (NIDDK) (U01DK061718, U01DK061728, U01DK061731, U01DK061732, U01DK061734, U01DK061737, U01DK061738, U01DK061730, and U01DK061713); the 10.13039/100006108National Center for Advancing Translational Sciences (UL1TR000439, UL1TR000436, UL1TR000006, UL1TR000448, UL1TR000100, UL1TR000004, UL1TR000423, UL1TR000058, and UL1TR001881); and the 10.13039/100000062NIDDK (DK063491 to the Southern California Diabetes Endocrinology Research Center). This study was supported by the German Federal Ministry for Education and Research (BmBF) through the Livers Systems Medicine (LiSyM) project. This work was supported by grants from the Swiss National Funds (SNF no. 310030_169196) and the 10.13039/501100011595Swiss Foundation for Alcohol Research (SSA) to F.S. This Raine Study was supported by the 10.13039/501100000925National Health and Medical Research Council of Australia (grant numbers 403981, 353514 and 572613). The UK 10.13039/501100000265Medical Research Council and 10.13039/100004440Wellcome (grant ref: 102215/2/13/2) and the 10.13039/501100000883University of Bristol provide core support for ALSPAC. ALSPAC GWAS data was generated by Sample Logistics and Genotyping Facilities at Wellcome Sanger Institute and LabCorp (Laboratory Corporation of America) using support from 23andMe. A comprehensive list of grants funding is available on the ALSPAC website (www.bristol.ac.uk/alspac/external/documents/grant-acknowledgements.pdf); this research was specifically funded by grants from 10.13039/501100000265MRC and 10.13039/501100000280Alcohol Research UK (MR/L022206/1) and 10.13039/100000002NIH (5R01AA018333-05) to K.W.M.A. and M.H. L.V. was supported by MyFirst Grant 10.13039/501100005010AIRC n.16888, Ricerca Finalizzata Ministero della Salute RF-2016-02364358, Ricerca corrente Fondazione IRCCS Ca’ Granda Ospedale Maggiore Policlinico. German 10.13039/501100002347Federal Ministry of Education and Research (BMBF LiSyM 031L0051 to F.L.). P.L. is supported by grants from the 10.13039/501100006306Sigrid Jusélius Foundation and the 10.13039/501100009708Novo Nordisk Foundation. The Fenland study was funded by grants to the MRC Epidemiology Unit (MC UU12015/1, MC UU 12015/5). R.B. and M.K. are employees of and shareholders in Perspectum Diagnostics Ltd. C.A.P. is funded by a 10.13039/100010269Wellcome Trust Clinical PhD Programme (206274/Z/17/Z). J.M.B. is supported by the Spanish 10.13039/501100004587Carlos III Health Institute (ISCIII; PI15/01132, PI18/01075, CIBERehd, and Miguel Servet Program CON14/00129) co-financed by 10.13039/501100008530Fondo Europeo de Desarrollo Regional (FEDER) and La Caixa Scientific Foundation (HR17-00601). Research from the GUARDIAN Study was supported DK085175 and DK118062, and phenotyping in IRASFS was supported by HL060944, HL061019, HL060919 and HL060894 IRASFS from the 10.13039/100000062National Institute of Diabetes and Digestive and Kidney Diseases (NIDDK). H.Y. is funded by a 10.13039/501100000361Diabetes UK RD Lawrence fellowship (17/0005594). Q.M.A. and A.D. supported by the 10.13039/501100009058EPoS (Elucidating Pathways of Steatohepatitis) consortium funded by the 10.13039/501100007601Horizon 2020 Framework Program of the 10.13039/501100000780European Union under Grant Agreement 634413. Q.M.A., A.D., L.V. and A.G. are members of the LITMUS (Liver Investigation: Testing Biomarker Utility in Steatohepatitis) consortium funded by the 10.13039/501100000780European Union 10.13039/501100010767Innovative Medicines Initiative 2 (IMI2) Joint Undertaking under grant agreement 777377. Q.M.A. is a Newcastle 10.13039/100006662NIHR Biomedical Research Center Investigator.

## Authors' contributions

Study concept and design: J.P.M.; acquisition of data: all; analysis and interpretation of data: K.T., J.K., S.S., L.V., J.P.M.; drafting of the manuscript: K.T., J.P.M.; critical revision of the manuscript for important intellectual content: all authors; statistical analysis: K.T., J.K., S.S., L.V., J.P.M.; obtained funding: J.P.M., S.C., N.S., J.K.D., J.K., P.L., J.M.B., C.A.P., H.Y., K.W.M.A., L.A., Q.M.A., A.D.K., T.B., G.S.G., C.H., J.H., J.L., P.E.M., T.A.M., N.D.P., S.R., J.I.R., E.K.S., S.S., A.T.H., L.E.W., L.V., H.Y.J., K.A.Y.; study supervision: J.P.M.

## Conflict of interest

C.E. reports receiving personal fees from Navitor Pharma and Novartis. The other authors declare no conflicts of interest that pertain to this work.

Please refer to the accompanying ICMJE disclosure forms for further details.

## Supplementary data

Supplementary information.pdf

CTAT table.pdf
