# Supplementary material for: rs641738C>T near MBOAT7 is associated with liver fat, ALT and fibrosis in NAFLD: A meta-analysis
Source: J Hepatol. 2021 Jan;74(1):20–30. doi: 10.1016/j.jhep.2020.08.027 (PMC7755037; doi:10.1016/j.jhep.2020.08.027)
Supplement: Supplementary information.pdf [file mmc1.pdf]

rs641738C>T near *MBOAT7* is positively associated with liver fat, ALT, and fibrosis in NAFLD: a meta-analysis

Kevin Teo, Kushala W. M. Abeysekera, Leon Adams, Elmar Aigner, Quentin M. Anstee, Jesus M. Banales, Rajarshi Banerjee, Priyadarshi Basu, Thomas Berg, Pallav Bhatnagar, Stephan Buch, Ali Canbay, Sonia Caprio, Ankita Chatterjee, Yii-Der Ida Chen, Abhijit Chowdhury, Ann K. Daly, Christian Datz, Dana de Gracia Hahn, Johanna K. DiStefano, Jiawen Dong, Amedine Duret, EU-PNAFLD Investigators, Connor Emdin, Madison Fairey, Glenn S Gerhard, GOLD Consortium, Xiuqing Guo, Jochen Hampe, Matthew Hickman, Lena Heintz, Christian Hudert, Harriet Hunter, Matt Kelly, Julia Kozlitina, Marcin Krawczyk, Frank Lammert, Claudia Langenberg, Joel Lavine, Lin Li, Hong Kai Lim, Rohit Loomba, Panu K. Luukkonen, Phillip E. Melton, Trevor A. Mori, Nicholette D. Palmer, Constantinos A. Parisinos, Sreekumar G Pillai, Faiza Qayyum, Matthias C. Reichert, Stefano Romeo, Jerome I. Rotter, Yu Ri Im, Nicola Santoro, Clemens Schafmayer, Elizabeth K. Speliotes, Stefan Stender, Felix Stickel, Christopher D. Still, Pavel Strnad, Kent D. Taylor, Anne Tybjærg-Hansen, Giuseppina Rosaria Umano, Mrudula Utukuri, Luca Valenti, Lynne E. Wagenknecht, Nicholas J. Wareham, Richard M. Watanabe, Julia Wattacheril, Hanieh Yaghootkar, Hannele Yki-Järvinen, Kendra A. Young, Jake P. Mann

Table of contents

Supplementary methods 3
Supplementary figures 10
Supplementary tables 26
Supplementary references 97
Code used in analyses 109
CTAT methods 310

Supplementary methods

Search terms

For identification of published studies:

PubMed (28/7/20): (MBOAT7[All Fields] OR membrane-bound-o-acyltransferase[All Fields]) OR (rs641738[All Fields] OR rs626283[All Fields]) OR TMC4[All Fields]

Embase (28/7/20): ‘MBOAT7' or 'rs641738' or 'rs626283' or 'TMC4’ {Including Limited Related Terms}

HuGe Navigator (28/7/20). HuGE Lit finder: ‘mboat7’, 'rs641738', 'rs626283', 'TMC4’. Phenopedia: ‘fatty liver’, ‘liver cirrhosis’, ‘hepatocellular carcinoma’, ‘fibrosis’, ‘End Stage Liver Disease’, ‘Esophageal and Gastric Varices’, ‘Hepatic Encephalopathy’, ‘Hepatic Insufficiency’ ‘Hepatitis, Chronic’, ‘Hypertension, Portal’, ‘Liver Diseases’, ‘Liver Failure’, ‘Liver Neoplasms’. Genopedia: ‘mboat7’, 'TMC4’

Web of Science: (28/7/20): MBOAT7 or rs641738 or rs626283 or TMC4 OR membrane-bound-o-acyltransferase

bioRxiv & medRxiv (28/7/20): "‘MBOAT7' or 'rs641738' or 'rs626283' or 'TMC4’ or 'membrane-bound-o-acyltransferase'"

For identification of relevant genome-wide association studies:

GWAS catalogue[1] (28/7/20). Traits: liver disease, liver enzyme measurement, liver disease biomarker, liver fibrosis measurement, non-alcoholic fatty liver disease, non-alcoholic fatty liver disease severity measurement, serum alanine aminotransferase measurement, aspartate aminotransferase measurement, non-alcoholic steatohepatitis, Hepatic fibrosis, hepatocellular carcinoma, Hepatitis. Genes: MBOAT7, TMC4. Variants: rs641738, 'rs626283'

Phenoscanner v2[2] (28/7/20). Variants: rs641738, rs626283. Traits: liver, liver enzyme, fatty liver, liver fibrosis, non-alcoholic fatty liver disease, alanine aminotransferase, non-alcoholic steatohepatitis, hepatocellular carcinoma, cirrhosis. Searches conducted with no p-value cut-off.

Type 2 diabetes and cardiovascular disease knowledge portals [3,4](28/7/20). Variants: rs641738, rs626283.

Cohorts with genome-wide data

Relevant GWASs were identified as described above. Data were included where densely imputed genotyping results were available for rs641738C>T or rs626283C>G in all with >0.98 call rate. These cohorts have been described elsewhere and detailed description of the quality control processes for genome-wide data is available in their original descriptions (Table 1), but in brief, single nucleotide polymorphisms (SNP) with Hardy-Weinberg equilibrium p-values $<1x10^{-6}$ were excluded prior to imputation. Association analysis was performed for variants with mean allele frequencies >0.01 and with minimum imputation quality of >0.3. Liver fat data from the UK BioBank was extracted under Application ID 9914 (‘Determining the Outcomes of People with Liver Disease’).

ALSPAC

Data was included from the Avon Longitudinal Study of Parents and Children (ALSPAC) [5–7]. This is a prospective, longitudinal study that originally enrolled 14,541 pregnancies with expected delivery dates between 1st April 1991-31st December 1992. After enrolment of 913 additional children at age 7, the total sample size is 15,454 pregnancies, resulting in 15,589 foetuses. Of these

14,901 were alive at 1 year of age. Ethical approval for the study was obtained from the ALSPAC Ethics and Law Committee and the Local Research Ethics Committees. Data included in this meta-analysis was from individuals who had attended a study visit between 22 and 26 years of age for transient elastography measurement with controlled attenuation parameter (CAP). Please note that the ALSPAC study website contains details of all the data that is available through a fully searchable data dictionary and variable search tool (http://www.bristol.ac.uk/alspac/researchers/our-data). Data was included for meta-analysis where a CAP measurement was recorded and the participant had genotyping data for rs641738C>T, which left 2,919 individuals for inclusion.

UK BioBank (UKBB)

Four studies included in the meta-analysis used data from the UKBB[8–11], though three of these did not provide the required data in a suitable format for meta-analysis. GWAS summary statistics for the association between rs641738C>T and biochemical traits were extracted from http://www.nealelab.is/uk-biobank/, however biochemical traits were unadjusted and therefore could not be pooled with other GWAS summary statistics. Therefore, GWAS summary statistics logarithmically-adjusted biochemical traits were extracted from The Global BioBank Engine[12]. The authors would like to thank the Rivas lab for making the resource available. Data on the quantitative assessment of liver fat from the UK BioBank cohort was extracted under Application ID 9914 (‘Determining the Outcomes of People with Liver Disease’).

Studies excluded due to lack of data

11 studies were potentially relevant however were not included in meta-analysis due to unavailability of the data after contacting their authors: [13–23]

Outcomes of interest

Given the strong evidence base for fibrosis stage as a prognostic marker in NAFLD[24], the primary outcome for the meta-analysis was the association of rs641738C>T with presence of advanced fibrosis (F3-4 versus F0-2) in individuals with NAFLD.

Dichotomous secondary outcomes were: radiological diagnosis of NAFLD (or hepatic steatosis); presence of severe steatosis on liver biopsy (S3 versus S1-2); presence of non-alcoholic steatohepatitis (NASH) on liver biopsy (NASH versus non-alcoholic fatty liver (NAFL)); presence of any fibrosis (F1-4 versus F0); presence of hepatocellular carcinoma (HCC) in patients with NAFLD (NAFLD-HCC versus NAFLD).

Continuous secondary outcomes were: quantitative, radiological hepatic fat content; total cholesterol; high-density lipoprotein cholesterol (HDL); low-density lipoprotein cholesterol (LDL); triglycerides (TG).

Patient and Public Involvement

Patients and public were not directly involved in the planning of this meta-analysis; however, many of the original studies included were co-produced with patient and public involvement.

Study quality assessment

Two reviewers independently assessed risk of bias in each study by applying the Cochrane Risk of Bias in Cohort Studies tool[25].

Statistical Analysis

Genotype frequencies for each study were assessed for Hardy-Weinberg equilibrium using chi-squared test.

Due to the unclear effect of this variant on liver disease and previous studies using multiple different models of inheritance, genetic association analyses were performed using additive, dominant, and recessive models for each outcome.

For dichotomous outcomes, the effect statistic was calculated as an odds ratio between groups.

For analysis of diagnosis of liver fat, a sensitivity analysis was performed by excluding studies where there was a risk of confounding due to differences between cases and controls.

For continuous variables, effect summaries were calculated as mean differences for recessive (CC+CT versus TT) and dominant (CC versus CT+TT) genetic models. Effect summary for the additive genetic model was calculated as a pooled beta regression coefficient using inverse-variance weighting with Fisher's z-transformation. The beta regression coefficient was calculated for each study using linear regression (coding number of T alleles as 0, 1, and 2). In addition, for

For analysis of effect on liver fat (continuous quantitative liver fat data from CT, MRI, MRS, or PDFF and semi-quantitative data using ultrasound or CAP), using the additive genetic model, data were inverse normalized and standardised (to mean = 0, standard deviation = 1). Linear regression was then used (coding the number of T alleles as 0, 1, and 2), adjusted for age, sex, and (where available) principal components of genetic ancestry.

For other continuous variables where raw data was available (i.e. triglycerides, HDL, LDL, ALT, and total cholesterol), effect summary was calculated as a mean difference between CC and TT groups.

For meta-analysis of GWAS summary statistics, pooled effect summaries were calculated where traits had been logarithmically transformed and an additive model had been used in analyses. Beta regression coefficients were pooled using inverse-variance pooling with Fisher's z-transformation.

Meta-analysis was performed using random effects throughout using DerSimonian-Laird method for estimation of tau^2.

Summary statistics were reported with 95% confidence intervals (CI). Data from paediatric and adult studies were analyzed separately. Sub-analysis was performed using only studies conducted in Caucasian populations (self-reported white, Non-Finnish-, or Finnish-European ethnicity) where data were available from at least four studies. This sub-analysis was selected due to initial identification of this variant in Caucasian individuals and further sub-analysis by ethnicity may be affected by differences in linkage disequilibrium between genetic ancestries. For studies including cohorts of multiple ethnicities, where available, data were analysed separately for each ethnicity.

An additional sub-analysis was performed for diagnosis of NAFLD (as a trait) by modality used to diagnose NAFLD. This was selected due to the different sensitivity and specificity of each modality for diagnosis of NAFLD.

Heterogeneity between groups was described using the Q statistic, tau^2, and I^2.

For dichotomous outcomes: p-value<0.017 (i.e. p<0.05/3) was considered statistically significant due to testing outcomes using three genetic association models. For other outcomes, where only a single genetic model was used, p-value<0.05 was considered significant.

Bias was assessed using Egger's test and funnel plots where more than 10 studies were included. Where Egger's test suggested bias ($p<0.05$), a funnel plot was generated and missing studies were imputed using Duval & Tweedie's trim-and-fill procedure[26]. Meta-regression was performed for random effects meta-analysis for histological outcomes and diagnosis of NAFLD as a trait. Study-level characteristics used as independent variables in meta-regression were: female sex (%), mean age (years), presence of type 2 diabetes (%), body mass index (kg/m^2), prevalence of rs738409C>G in *PNPLA3*, and only for HCC, presence of cirrhosis (%).

Analysis was performed using STATAv14 for Windows (StataCorp. 2015. Stata Statistical Software: Release 14. College Station, TX: StataCorp LP) and R 3.6.1[27,28]. Script used in analyses in R is available below.

Supplementary figures

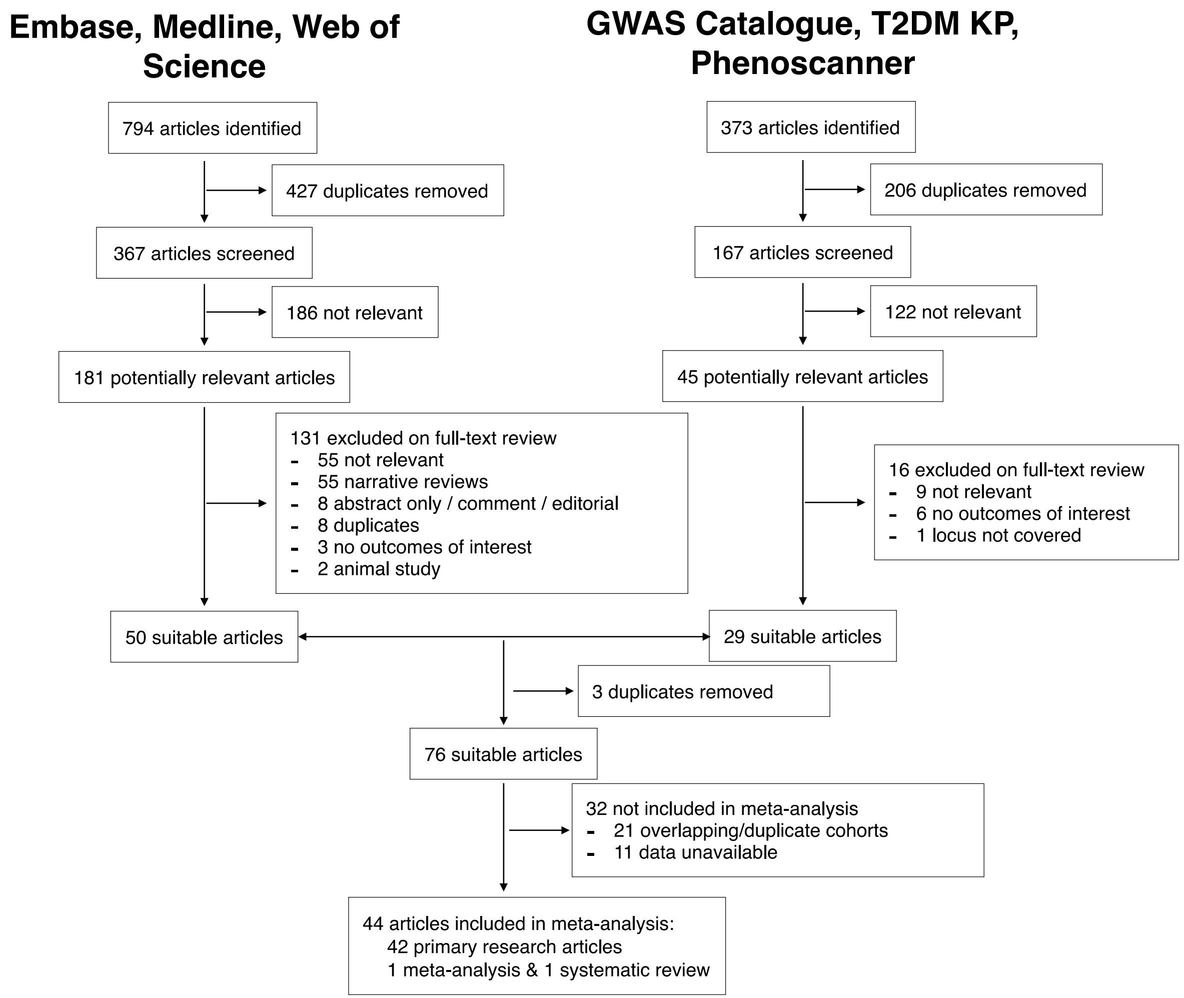

Fig. S1.

Inclusion-exclusion flow chart for the meta-analysis. Two separate sets of searches were performed: for genome-wide association studies (GWAS), using GWAS Catalogue, Type 2 Diabetes Mellitus Knowledge Portal (T2DM KP), cardiovascular

disease knowledge portal, and Phenoscanner; and for all other studies, using Embase, Medline, Web of Science, HugeNet Navigator, medRxiv and bioRxiv.

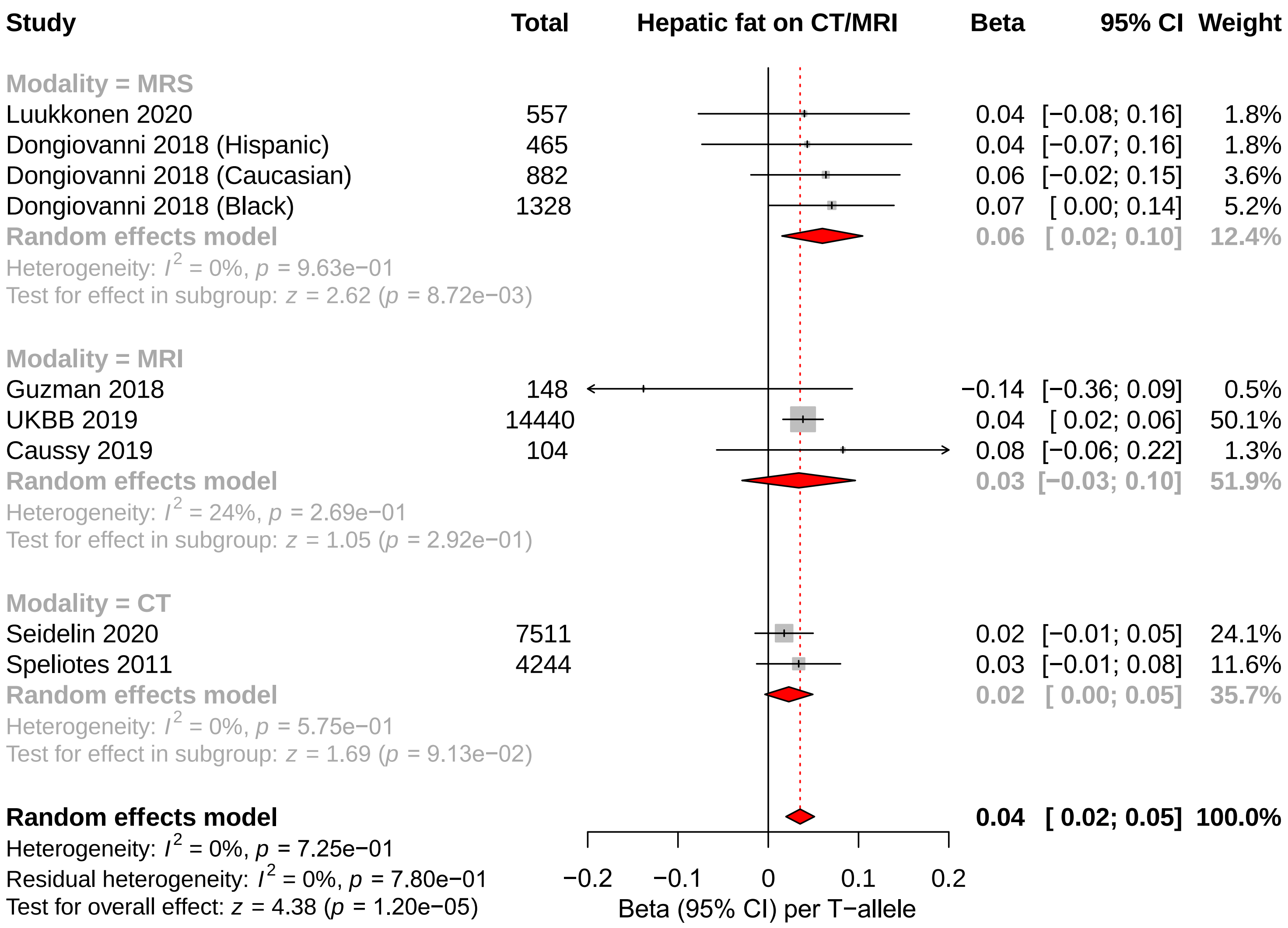
Study
Total
Hepatic fat on CT/MRI
Beta
95% CI
Weight
Modality = MRS
Luukkonen 2020 557 0.04 [−0.08; 0.16] 1.8%
Dongiovanni 2018 (Hispanic) 465 0.04 [−0.07; 0.16] 1.8%
Dongiovanni 2018 (Caucasian) 882 0.06 [−0.02; 0.15] 3.6%
Dongiovanni 2018 (Black) 1328 0.07 [0.00; 0.14] 5.2%
Random effects model 0.06 [0.02; 0.10] 12.4%
Heterogeneity: I2 = 0%, p = 9.63e−01
Test for effect in subgroup: z = 2.62 (p = 8.72e−03)
Modality = MRI
Guzman 2018 148 −0.14 [−0.36; 0.09] 0.5%
UKBB 2019 14440 0.04 [0.02; 0.06] 50.1%
Caussy 2019 104 0.08 [−0.06; 0.22] 1.3%
Random effects model 0.03 [−0.03; 0.10] 51.9%
Heterogeneity: I2 = 24%, p = 2.69e−01
Test for effect in subgroup: z = 1.05 (p = 2.92e−01)
Modality = CT
Seidelin 2020 7511 0.02 [−0.01; 0.05] 24.1%
Speliotes 2011 4244 0.03 [−0.01; 0.08] 11.6%
Random effects model 0.02 [0.00; 0.05] 35.7%
Heterogeneity: I2 = 0%, p = 5.75e−01
Test for effect in subgroup: z = 1.69 (p = 9.13e−02)
Random effects model 0.04 [0.02; 0.05] 100.0%
Heterogeneity: I2 = 0%, p = 7.25e−01
Residual heterogeneity: I2 = 0%, p = 7.80e−01
Test for overall effect: z = 4.38 (p = 1.20e−05)
−0.2
−0.1
0
0.1
0.2
Beta (95% CI) per T−allele

Fig. S2.

The effect of rs641738C>T on liver fat with sub-analysis by imaging modality. Data from 29,679 individuals with CT, MRI, or MRS liver fat. rs641738C>T positively associated with liver fat in Caucasian populations, where data represents standard deviation change in normalized liver fat per T-allele. CI, confidence interval; UKBB, UK BioBank.

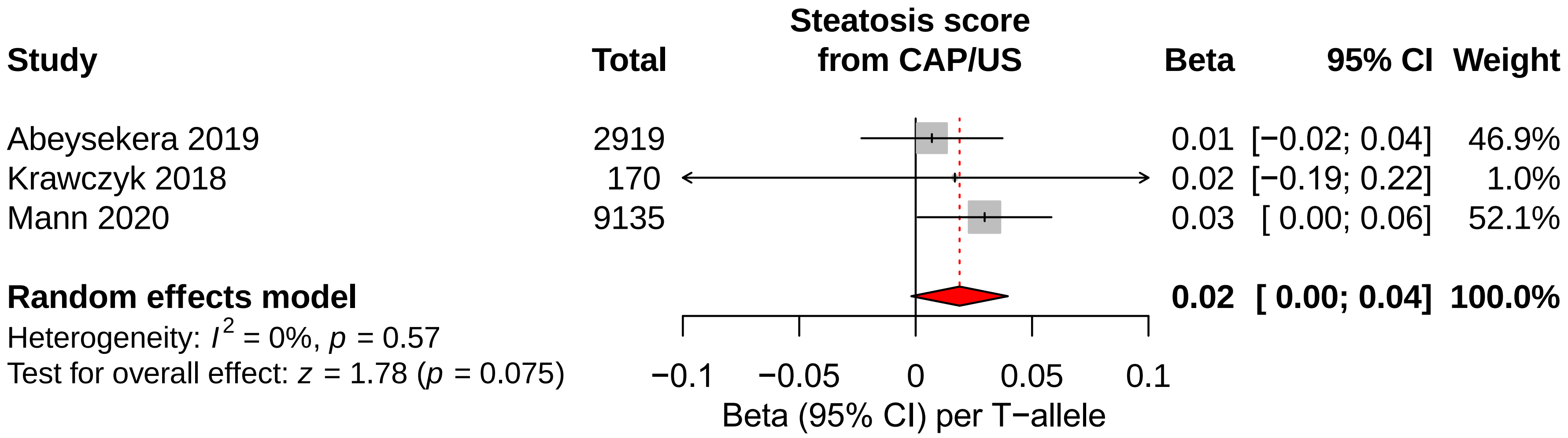

Fig. S3.

The effect of rs641738C>T on non-invasive measures of hepatic steatosis. rs641738C>T was positively associated with attenuation parameter (CAP) and semi-quantitative ultrasound score (US), where Beta regression-coefficient represents standard deviation change in inverse-normalised controlled CAP/US score. CI, confidence interval.

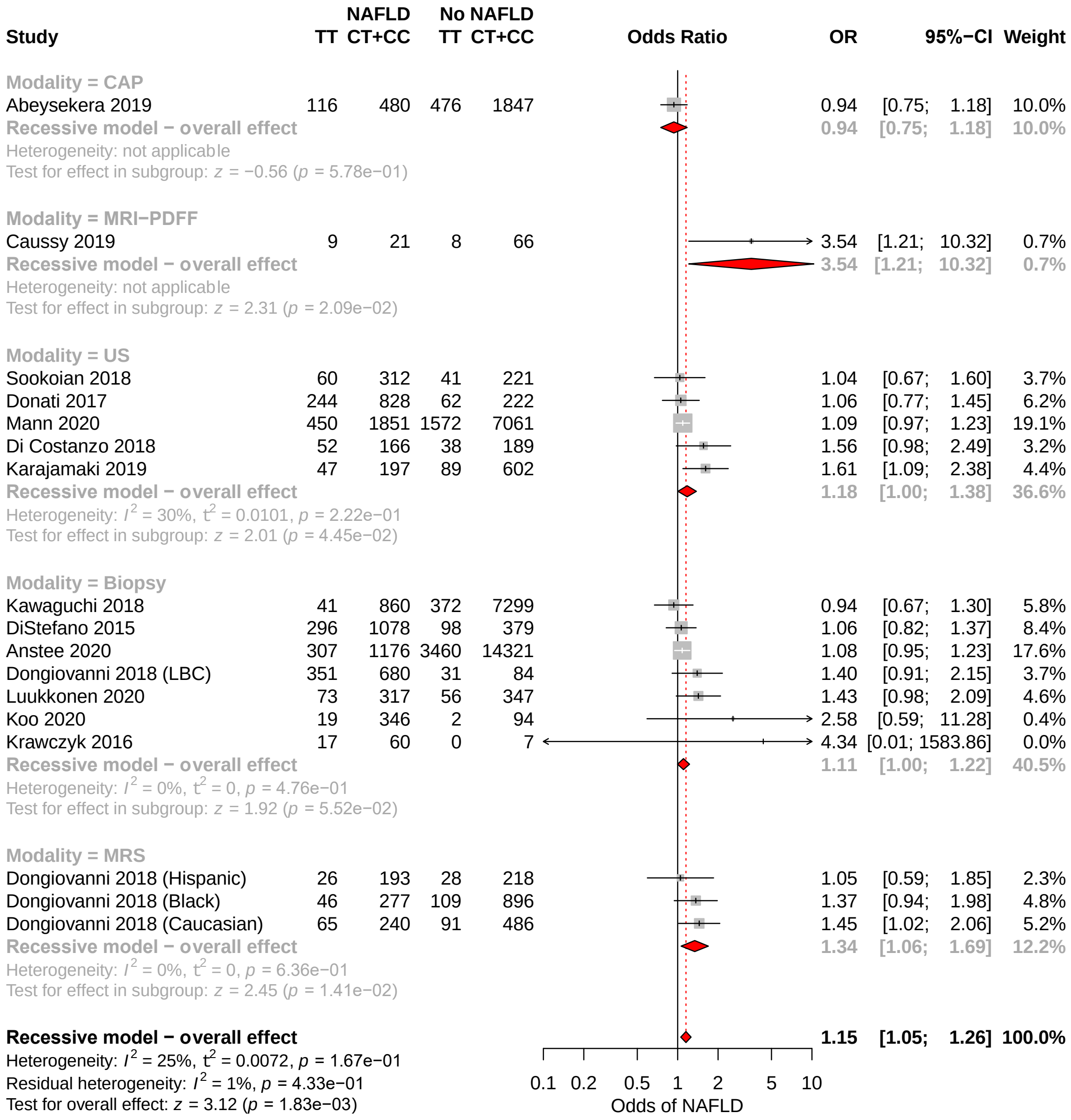

Fig. S4.

The effect of rs641738C>T on diagnosis of NAFLD with sub-analysis by modality. Data from 52,173 adults (11,301 cases and 40,872 controls) with radiologically or histologically defined steatosis for presence versus absence of NAFLD, separated by

modality used to diagnose NAFLD cases. CAP, controlled attenuation parameter; CI, confidence interval; LBC, Liver Biopsy Cohort; MRI-PDFF, magnetic resonance imaging proton density fat fraction; MRS, magnetic resonance spectroscopy; OR, odds ratio; US, ultrasound.

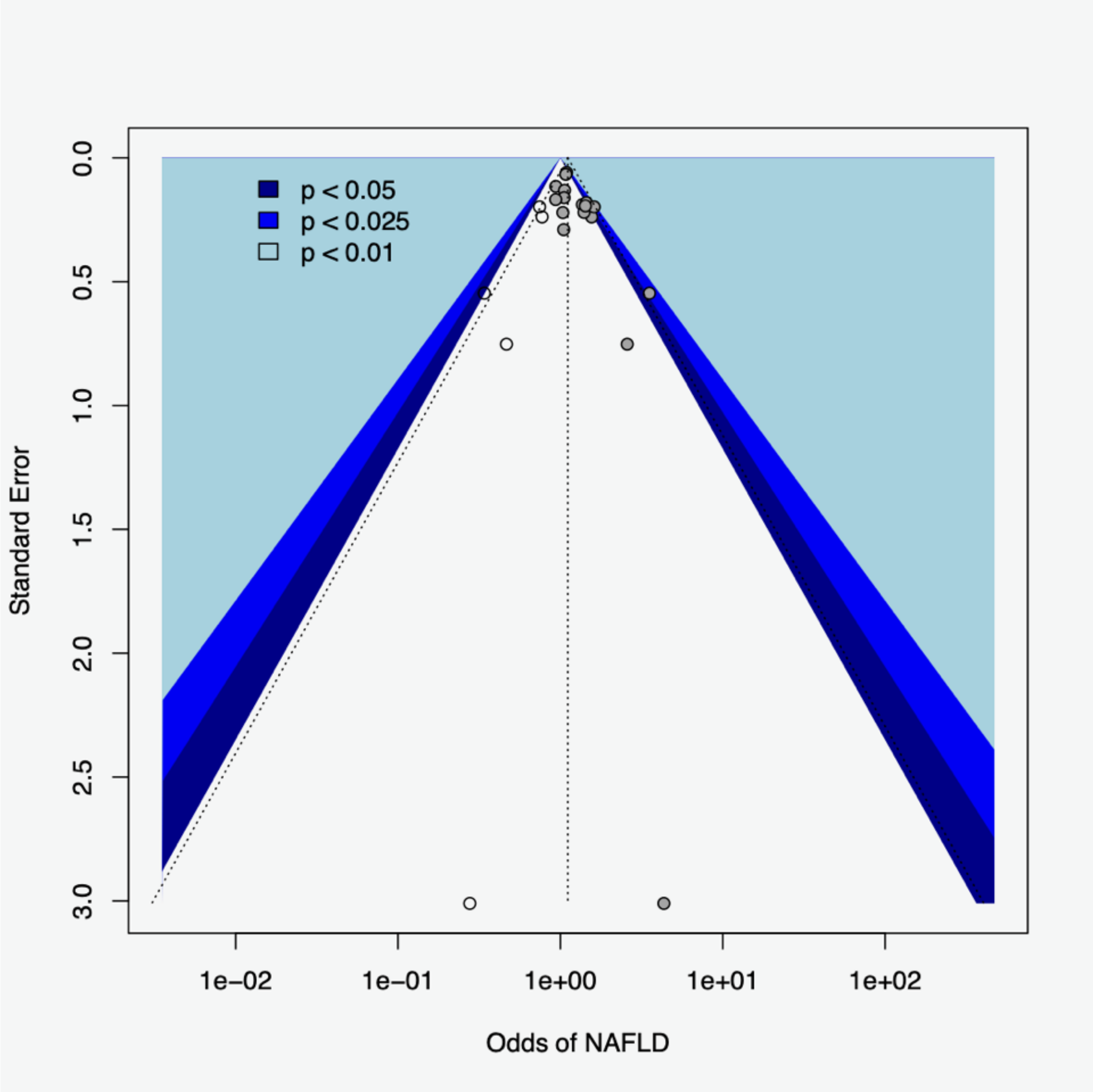
p < 0.05
p < 0.025
p < 0.01
Standard Error
0.0
0.5
1.0
1.5
2.0
2.5
3.0
1e−02
1e−01
1e+00
1e+01
1e+02
Odds of NAFLD

Fig. S5.

Funnel plot with filled studies for diagnosis of NAFLD in adults using a recessive model of inheritance. Funnel plot illustrating study distribution (publication) bias in 17 original studies (solid grey circles) with 5 added studies (from trim and fill). Egger's test p-value = 0.01 using the original studies. The statistical significance associated with each study is illustrated with the coloured background.

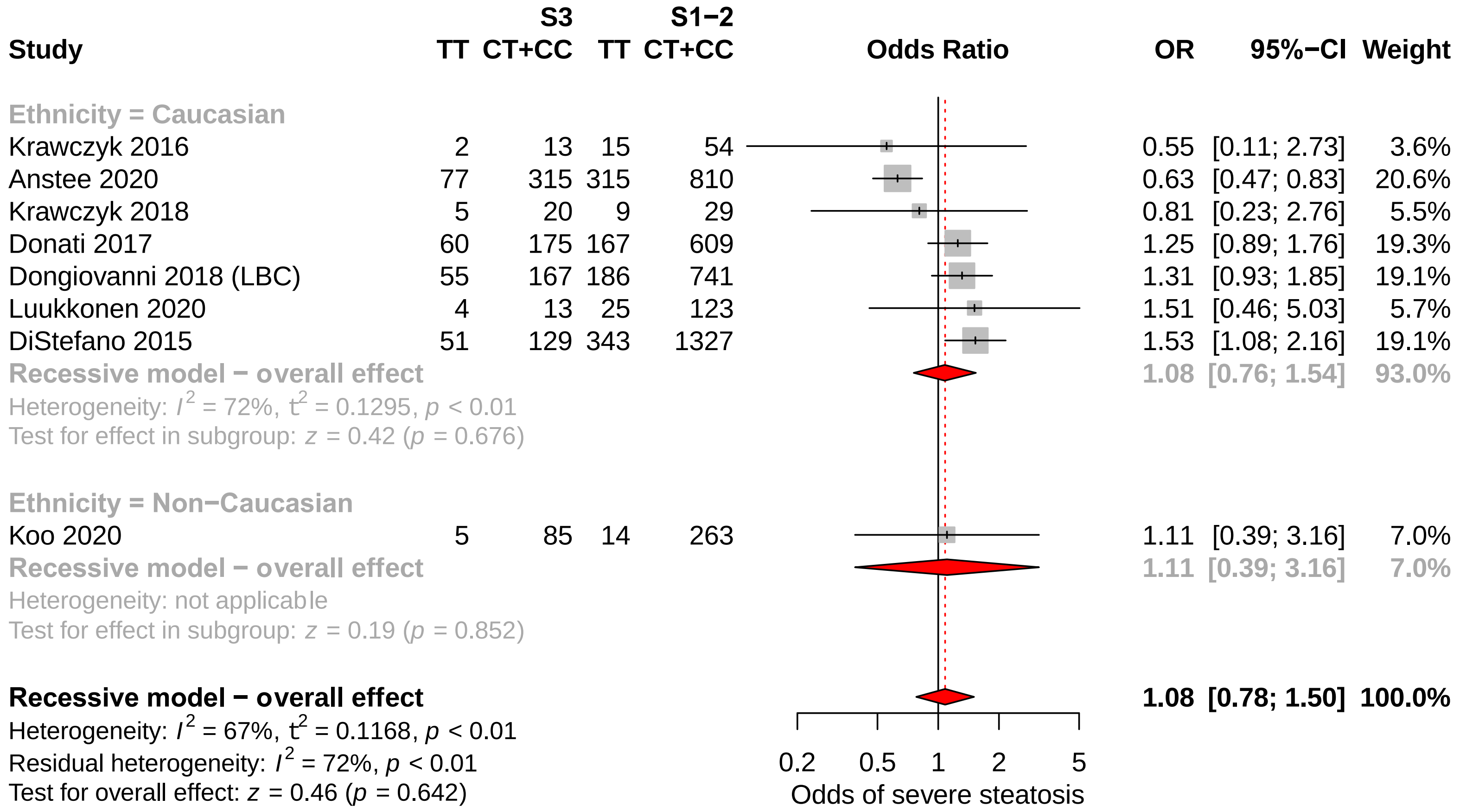

Fig. S6.

The effect of rs641738C>T on severe steatosis in adult patients with NAFLD. Data from 6,206 adults (1,176 cases and 5,030 controls) with NAFLD for the presence of severe steatosis (S3) mild/moderate steatosis (S1-2) using a recessive model of inheritance (CC+CT vs. TT) . CI, confidence interval; LBC, Liver Biopsy Cohort; OR, odds ratio.

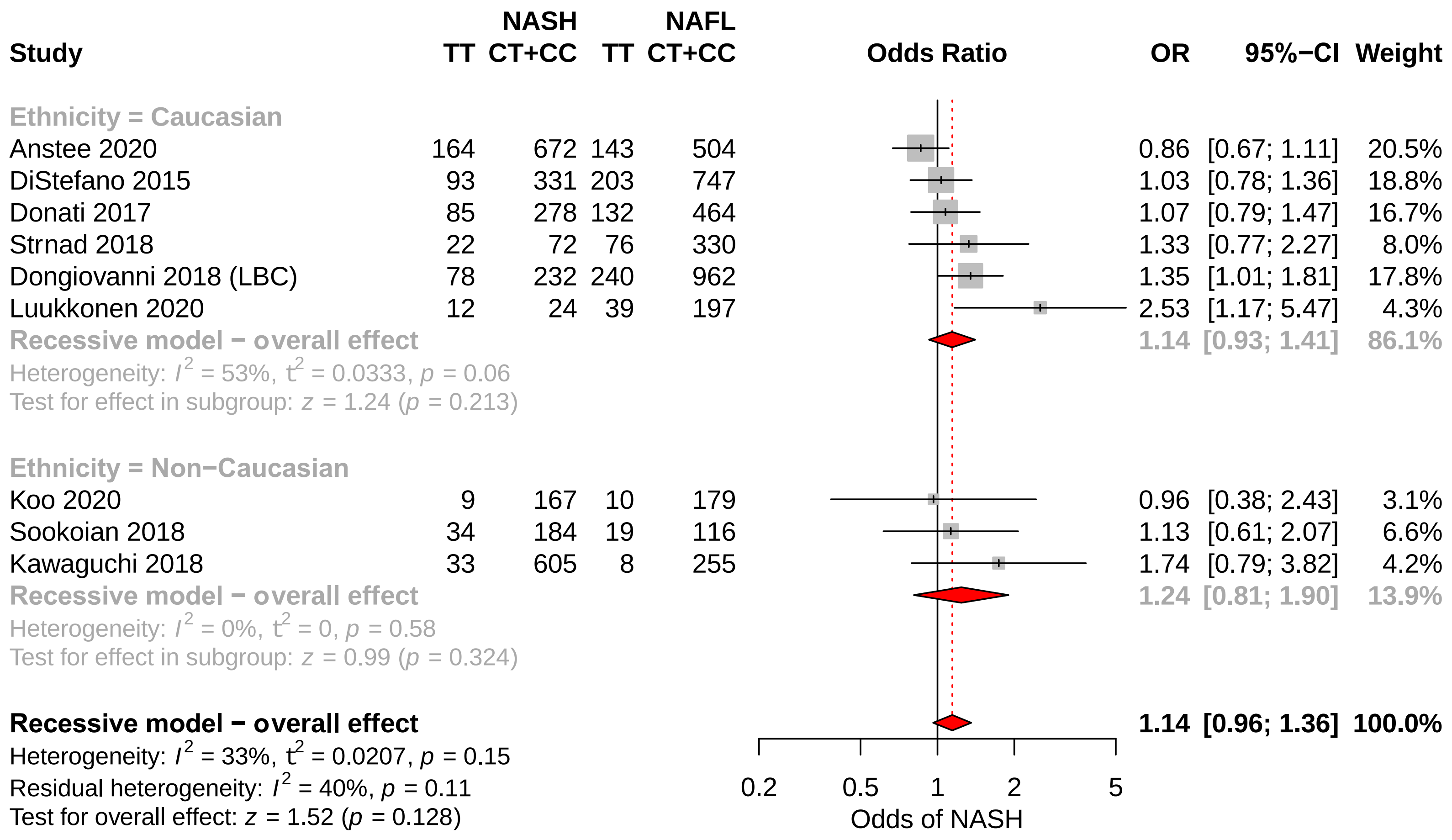

Fig. S7.

The effect of rs641738C>T on diagnosis of NASH in adult patients with NAFLD. Data from 7,719 adults (3,095 cases and 4,624 controls) with NAFLD for the presence of non-alcoholic steatohepatitis (NASH) versus non-alcoholic fatty liver (NAFL) using a recessive model of inheritance (CC+CT vs. TT) . Critical p-value for association: $p<0.017$. CI, confidence interval; LBC, Liver Biopsy Cohort; OR, odds ratio.

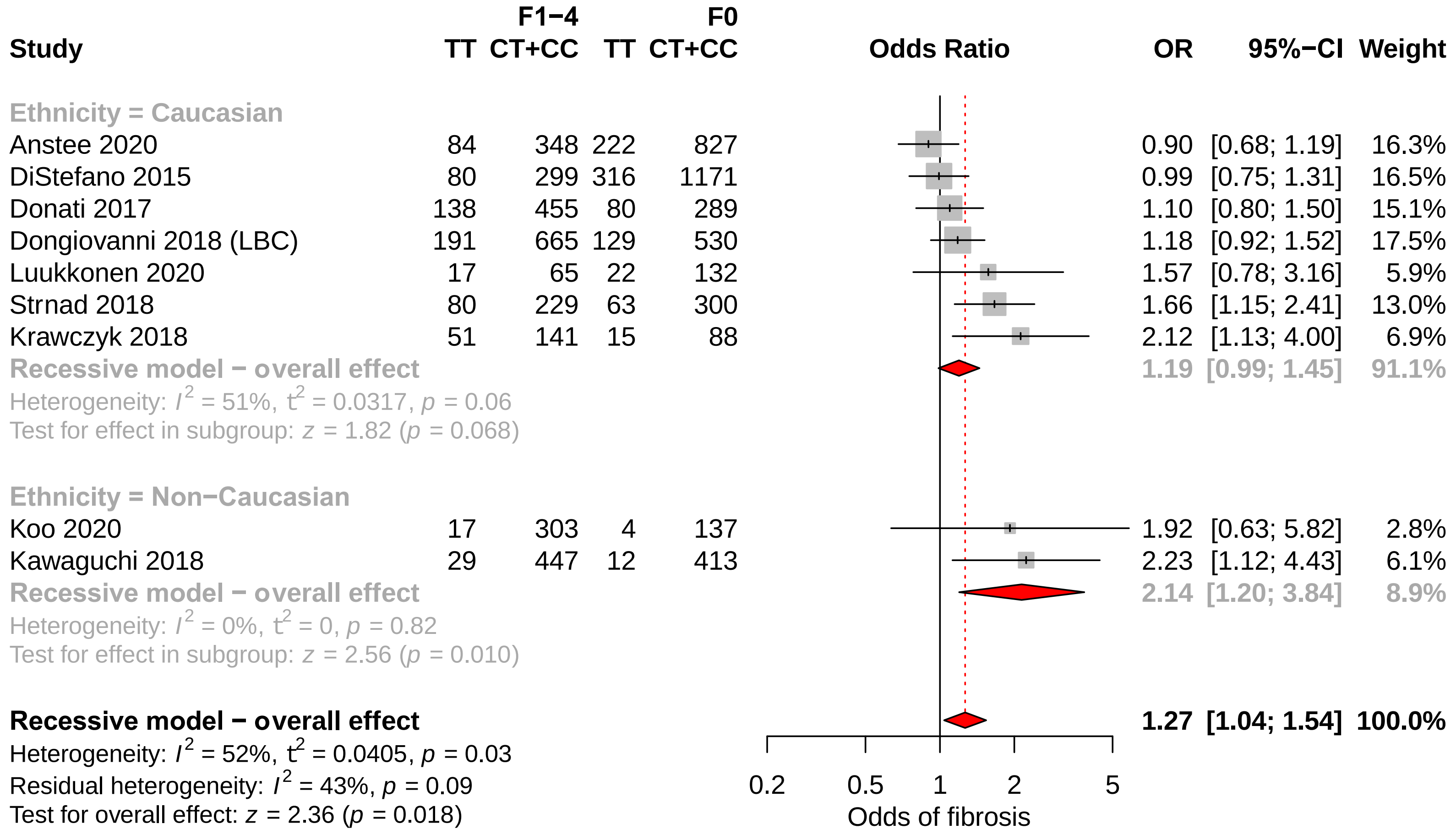

Fig. S8.

The effect of rs641738C>T on presence of any fibrosis in adult patients with NAFLD. Data from 8,389 adults (3,639cases and 4,750 controls) with NAFLD for the presence of fibrosis (F1-4) versus no fibrosis (F0) using a recessive model of inheritance (CC+CT vs. TT) . CI, confidence interval; LBC, Liver Biopsy Cohort; OR, odds ratio.

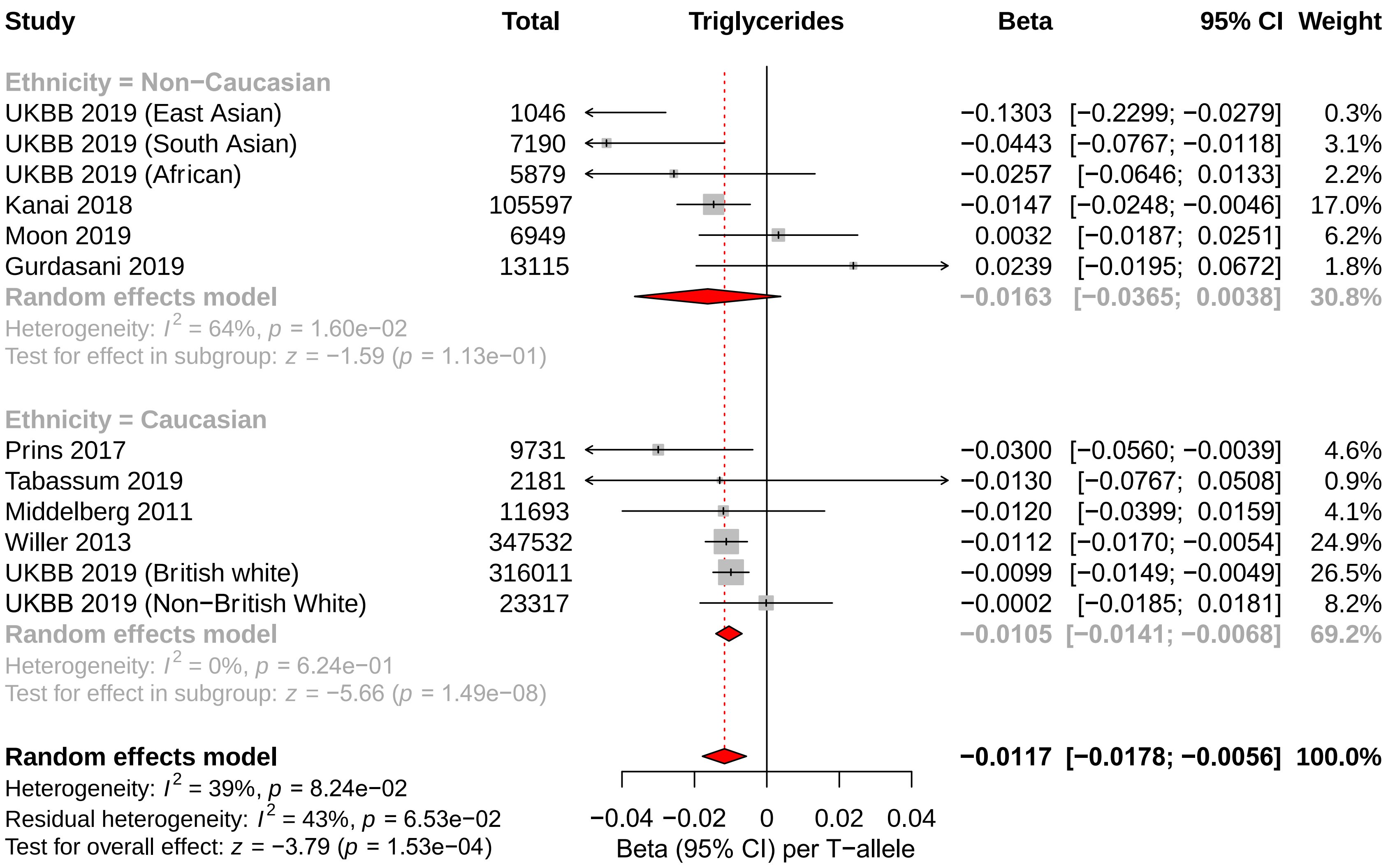

Fig. S9.

rs641738C>T is negatively associated with serum triglycerides in Caucasian populations in genome-wide association studies (GWAS). Meta-analysis of GWAS summary statistics for the association between rs641738C>T on logarithmically-transformed triglycerides using linear regression. CI, confidence interval; UKBB, UK BioBank.

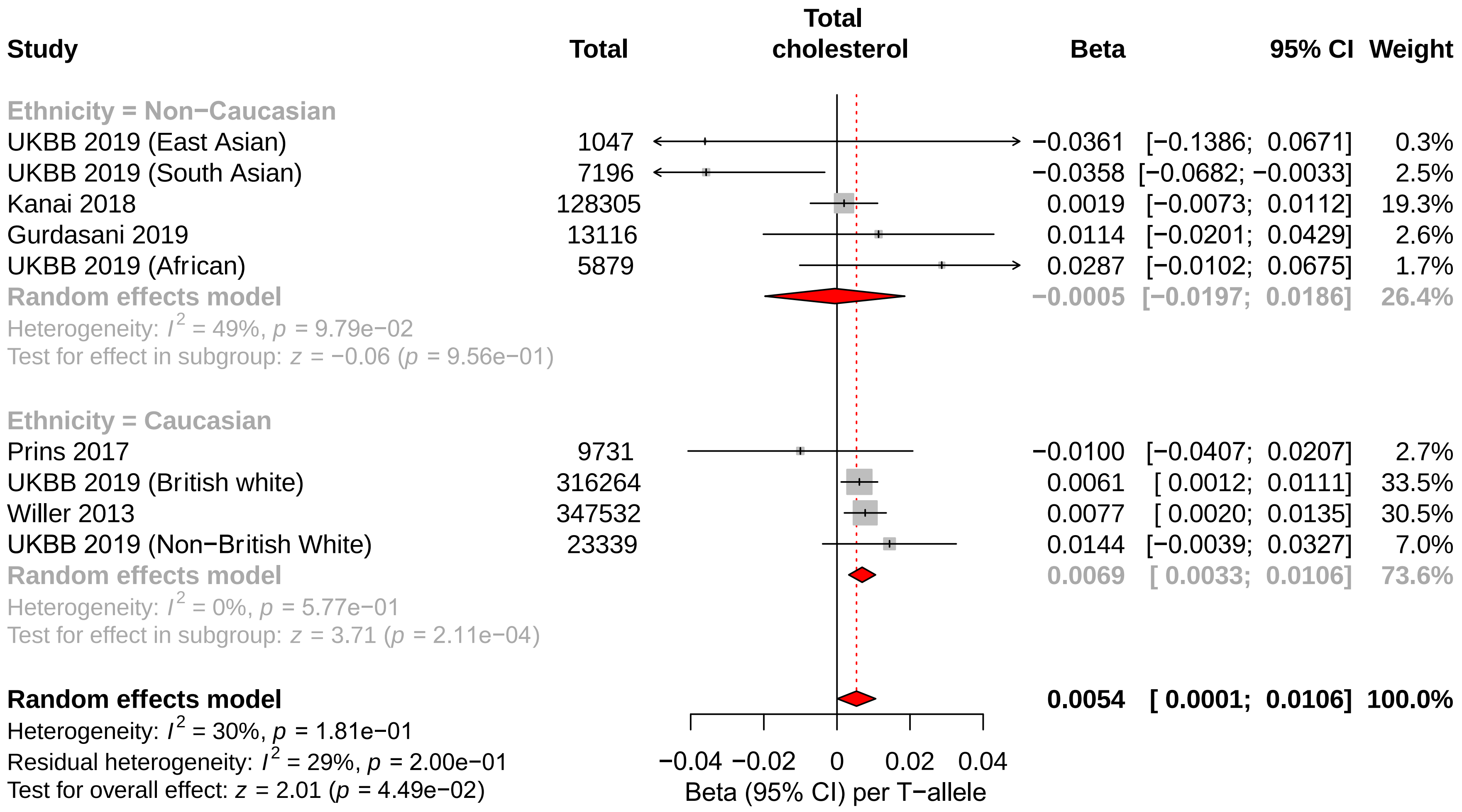

Fig. S10.

rs641738C>T is positively associated with serum total cholesterol in Caucasian populations in genome-wide association studies (GWAS). Meta-analysis of GWAS summary statistics for the association between rs641738C>T on logarithmically-transformed total cholesterol using linear regression. CI, confidence interval; UKBB, UK BioBank.

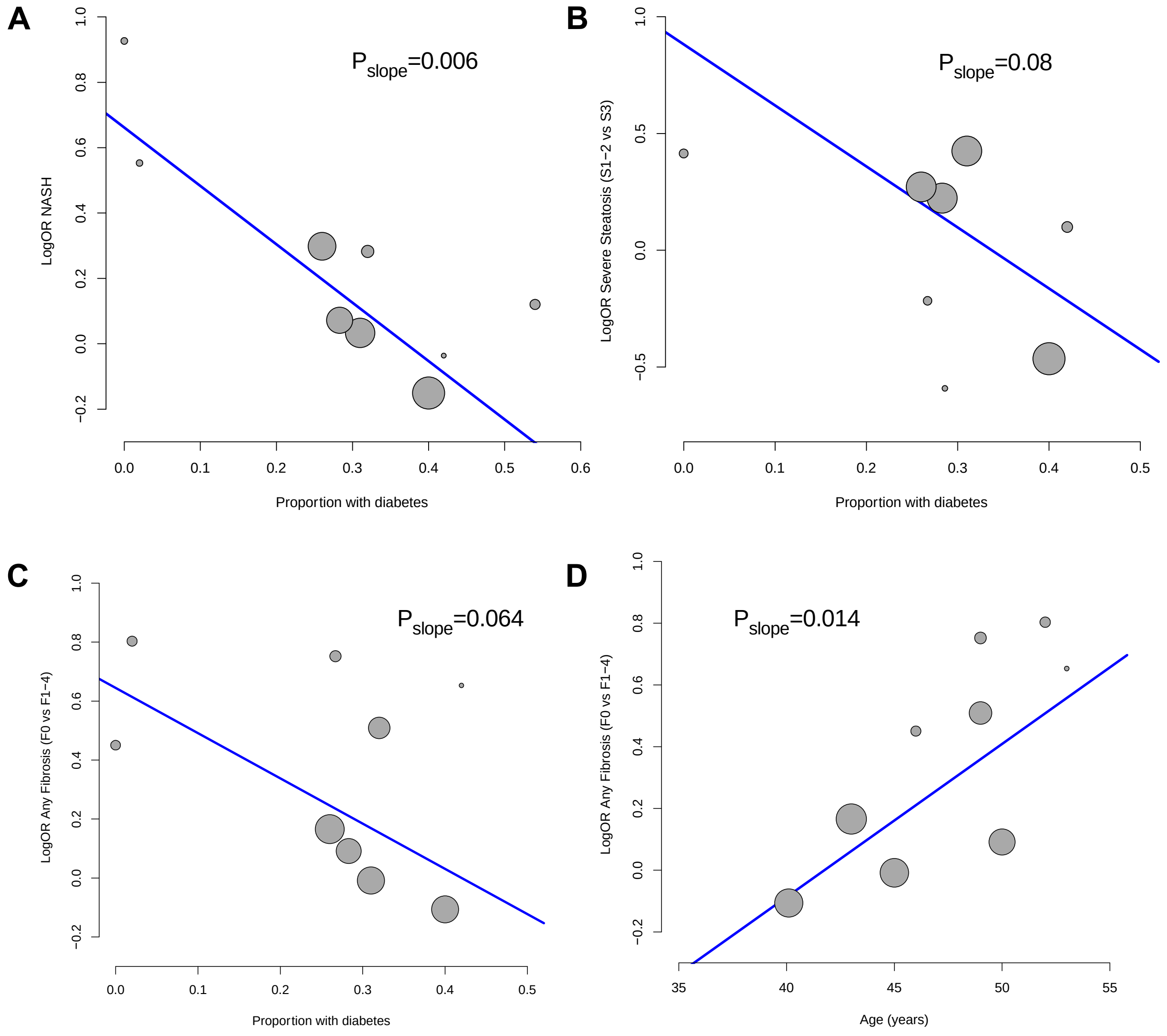

Fig. S11.

Meta-regression analyses for the interaction between prevalence of type 2 diabetes or age and histological associations with rs641738C>T . All analyses were performed from random effects meta-analyses using a recessive model of inheritance. A,

proportion with type 2 diabetes and NASH versus NAFL; B, proportion with type 2 diabetes and severe steatosis; C, proportion with type 2 diabetes and any fibrosis; D, mean age and any fibrosis. P_{slope} refers to the beta regression coefficient from univariate meta-regression.

Supplementary tables

**Principle study**	**Cohort(s)**	**Other studies using same cohort**	**Notes**
Speliotes 2011[29]	Genetics of Liver Disease (GOLD) Consortium	[30]	
Di Costanzo 2019[31]	Rome (paediatric)	[32]	
Di Sessa 2018[33]	Naples (paediatric)	[34]	
Chalasani 2010[22]	NASH-CRN (adult)	[35]	
Kanai 2018[36]	BioBank Japan	[37]	
Kawaguchi 2018[38]	Japan (Honshu)	[39]	
UK BioBank (UKBB)[11]	UKBB	[8,10,40]	Chen 2020[9] also included data from the UKBB, however this study also included unique data from the Michigan Genomics Initiative (MGI), therefore "Chen 2020" will refer to MGI data.
Koo 2020	Boramae	[41,42]	
Krawczyk 2018[43]	German NAFLD CSG	[44–46]	

Luukkonen 2020[47]	Helsinki	[48,49]	
Dongiovanni 2018[50]	DHS & LBC	[51–54]	
Krawczyk 2016[55]	San Sebastian (bariatric)	[56]	
Chambers 2011[57]	Multiple	[58]	
Donati 2017[59]	Italian NAFLD-HCC & UK NAFLD HCC	[60]	
Strnad 2018[61]	GER-AUS cohort	[62]	

Table S1.

Overlapping articles/cohorts included in the meta-analysis. Several articles were noted to report data from the same cohort and where this happened, the data from the largest reported cohort was included. The ‘Principle study’ is the article that is referred to in the main text, figures, and analyses.

Study; Age group	Study design and sample size (N)	Liver biopsy (N)	Features and participant characteristics	Outcomes
Abeysekera 2019[63]; Adult	Population-based cohort N=2,919	NA	GWAS data from the ALSPAC birth cohort study with steatosis diagnosed using CAP >240kPa	NAFLD diagnosis, liver fat (CAP)
Adams 2013[64]; Paediatric	Population-based cohort N=948	NA	GWAS of adolescents with hepatic steatosis measured by US	NAFLD diagnosis, biochemistry
Anstee 2020[65]; Adult	Hospital-based cases, plus population controls; N=19,264	1517	GWAS adults with cases from the EPoS Consortium with NAFLD diagnosed with LB, and healthy population controls	NAFLD diagnosis, histology
Caussy 2019[66]; Adult	Family, twin, & sibling-paired study N=104	NA	Community-based family case-control study with NAFLD diagnosed by MRI-PDFF	NAFLD diagnosis, liver fat, biochemistry
Chambers 2011[57];	Population-based cohorts N=61,089	NA	GWAS of adults from 21 population-based cohorts for concentration of serum liver enzymes	ALT (GWAS)

Adult				
Chatterjee 2018[67]; Adult	Hospital-based case-control N=354	132	Exome-wide association study of adults with NAFLD diagnosed by LB or MRI	NAFLD diagnosis, liver fat, histology
Chen 2020[9]; Adult	Hospital-based cohort N=19,598	NA	GWAS of adults undergoing elective surgery with linkage to electronic health records	Biochemistry (GWAS)
Di Costanzo 2018[68]; Adult	Hospital-based case-control N=445	NA	Sample from adult obesity clinic with NAFLD diagnosed using US	NAFLD diagnosis, biochemistry
Di Costanzo 2019[31]; Paediatric	Hospital-based case-control N=230	NA	Sample from paediatric obesity clinic with NAFLD diagnosed using MRI hepatic fat fraction	NAFLD diagnosis, liver fat, biochemistry
Di Sessa 2018[33]; Paediatric	Hospital-based case-control N=1,002	NA	Sample from paediatric obesity clinic with NAFLD diagnosed using US	NAFLD diagnosis, biochemistry
DiStefano 2015[69];	Hospital-based case-control	1868	GWAS of adults undergoing bariatric surgery with NAFLD diagnosed by LB	NAFLD diagnosis, histology

Adult	N=1,868			
Donati 2017[59]; Adult	Hospital-based case-control<br>N=1073 (Italian)<br>N=358 (UK)	1123	Cohorts from adult liver/obesity clinics with NAFLD diagnosed using US and/or LB	NAFLD diagnosis, histology, HCC
Dongiovanni 2018 (DHS)[50]; Adult	Population-based cohort<br>N=2,675	NA	GWAS of adults from population-based cohort with NAFLD diagnosed using MRS	NAFLD diagnosis, liver fat, biochemistry
Dongiovanni 2018 (LBC)[50]; Adult	Hospital-based case-control<br>N=1,515 (LBC)	1515	Samples from adult liver/obesity clinics and bariatric surgery with NAFLD diagnosed using LB	NAFLD diagnosis, histology, biochemistry
Gurdasani 2019[70]; Adult	Population-based cohorts<br>N=13,116	NA	GWAS of adults from 4 population-based cohort studies, including concentration of serum liver enzymes and biochemistry	Biochemistry (GWAS)
Guzman 2018[71];	Clinical trial-based case-control	NA	Sample of adults with type 2 diabetes who had taken part in one of two interventional diabetes trials, with	Liver fat

Adult	N=148		NAFLD diagnosed on MRI	
Hudert 2019[72]; Paediatric	Hospital-based case-control N=270	70	Cases from paediatric liver clinic with diagnosis of NAFLD using LB	NAFLD diagnosis, liver fat, histology
Kanai 2018[36]; Adult	Population-based cohort N=134,182	NA	GWAS of adults from a population cohort study with biobank, including concentration of serum liver enzymes and biochemistry	Biochemistry (GWAS)
Karajamaki 2019[73]; Adult	Population-based case-control N=935	NA	Population-level case-control study with recruitment of cases with hypertension; NAFLD diagnosed using US	NAFLD diagnosis, biochemistry
Kawaguchi 2018[38]; Adult	Mixed hospital- and population-based case-control N=8,571	936	Sample of adults from liver clinics with NAFLD diagnosed by LB	NAFLD diagnosis, histology, HCC, biochemistry
Koo 2020[74]; Adult	Hospital-based case-control N=461	461	Sample of adults from hospital clinics with NAFLD diagnosed by LB	NAFLD diagnosis, histology, biochemistry

Krawczyk 2016[55]; Adult	Hospital-based case-control N=84	84	Samples from adults undergoing bariatric surgery with NAFLD diagnosed using LB	Histology, biochemistry
Krawczyk 2018[43]; Adult	Hospital-based case-control N=515	295	Sample of adults from liver clinics with NAFLD diagnosed by US or LB	Histology, liver fat (CAP), biochemistry
Lin 2018[75]; Paediatric	Population-based cohort N=819	NA	Cohort of children recruited from schools with diagnosis of NAFLD using US	NAFLD diagnosis, biochemistry
Luukkonen 2020[47]; Adult	Hospital-based case-control N=793	236	Sample of adults undergoing bariatric surgery with NAFLD diagnosed by LB or MRS	NAFLD diagnosis, liver fat, histology, biochemistry
Mann 2018[76]; Paediatric	Hospital-based case-control N=379	306	Sample from paediatric obesity and liver clinics with diagnosis of NAFLD using LB or US	NAFLD diagnosis, histology, biochemistry
Mann 2020[77] Adult	Population-based cohort N=10,934	NA	Cohort study of adults from population with NAFLD diagnosed by US	NAFLD diagnosis, liver fat (US), biochemistry

Middelberg 2011[78]; Adult & paediatric	Population-based cohort N=11,693	NA	Combination of two cohort studies: adolescent twins and siblings; and adult twins, both population-based, with GWAS including concentration of serum liver enzymes and biochemistry	Biochemistry (GWAS)
Moon 2019[79]; Adult	Population-based cohort N=6,949	NA	GWAS of adults from a population cohort study with biobank, including concentration of serum liver enzymes and biochemistry	Biochemistry (GWAS)
Prins 2017[80]; Adult	Population-based cohort N=9,731	NA	GWAS of adults from a population cohort study with biobank, including concentration of serum liver enzymes and biochemistry	Biochemistry (GWAS)
Reichert 2019[81]; Adult	Hospital-based case-control N= 54	NA	Sample of adults from liver clinics with NAFLD cirrhosis diagnosed by LB, or US/MRI/CT	HCC
Seidelin 2020[82]; Adult	Population-based cohort N=7511	NA	Hepatic steatosis measured by CT, part of the Copenhagen General Population Study	Liver fat
Sookoian 2018[83];	Hospital-based case-control	372	Samples from adult liver clinics and bariatric surgery patients with NAFLD diagnosed by LB or US	NAFLD diagnosis, histology

Adult	N=634			
Speliotes 2011[29]; Adult	Population-based cohorts N=4,244	NA	GWAS of adults from 4 population-based cohorts for hepatic fat content as determined by CT	Liver fat
Strnad 2018[61]; Adult	Hospital-based case-control N=672	672	Sample of adults from liver clinics with NAFLD diagnosed by LB	Histology
Tabassum 2019[84]; Adult	Population-based cohorts N=2,181	NA	GWAS of adults from 2 population-based cohorts with data on serum triglycerides	Biochemistry (GWAS)
UKBB 2019[11]; Adult	Population-based N=14,440 (liver fat); N=77,464 (coded fibrosis); N= 353,596 (biochemistry)	NA	GWAS of hepatic steatosis measured by MRI from the UK BioBank; adults with coded diagnosis of NAFLD and cirrhosis; and concentration of serum biochemistry available from GWAS	Liver fat, biochemistry (GWAS), fibrosis (coding data)
Umano 2018[85];	Hospital-based case-control	NA	Sample from paediatric obesity clinic with NAFLD diagnosed using MRI hepatic fat fraction	Liver fat, biochemistry

Paediatric	N=878			
Verma 2017[86]; Adult	Hospital-based cohort N=31,466	NA	GWAS of adult hospital patients with linkage to electronic health records	Biochemistry (GWAS)
Viitasalo 2016[87]; Paediatric	Population-based cohort N=467	NA	Population cohort of children recruited from schools with measurement of ALT	Biochemistry
Wattacheril 2017[88]; Paediatric	Cases-only N=208	208	GWAS of Hispanic boys with NAFLD diagnosed by LB	Histology
Willer 2013[89]; Adult	Population-based cohorts N=347,532	NA	GWAS of adults from 45 population-based cohorts with data on serum lipids	Biochemistry (GWAS)
Young 2019[90]; Adult	Population-based cohorts N=3,555	NA	GWAS of adults from 5 population-based cohorts with data on ALT	Biochemistry (GWAS)
Zusi 2019[91];	Hospital-based case-	NA	Sample from paediatric obesity clinic with diagnosis of	NAFLD diagnosis,

Paediatric	control<br><br>N=510		NAFLD using US	biochemistry

Table S2.

Characteristics of studies included in the meta-analysis. Further characteristics available in Supplementary Tables 2-3. ALSPAC, Avon Longitudinal Study of Parents and Children; CAP, controlled attenuation parameter; CT, computerized tomography; DHS, Dallas Heart Study; GWAS, genome-wide association study; LB, liver biopsy; LBC, Liver Biopsy Cohort; MRI, magnetic resonance imaging; MRS, magnetic resonance spectroscopy; NA, not applicable; US, ultrasound.

**Study**<br>**Age group**	**TT**	**CT**	**CC**	**EAF**	**Total**	**HWE p-value**	**Variant**	**Genotyping method**	**Risk of bias**
Abeysekera 2019[63]; Adult	592 (20%)	1425 (49%)	902 (31%)	0.45	2919	0.4998	rs641738 C>T	Illumina SNP arrays: 317, 610, and 550 (custom)	5
Adams 2013[64]; Paediatric	177 (19%)	453 (48%)	318 (34%)	0.43	948	0.2372	rs641738 C>T	lluminaHuman660-W Quad Array	5
Anstee 2020[65]; Adult	3767 (20%)	9414 (49%)	6083 (32%)	0.44	19264	0.3008	rs641738 C>T	Illumina SNP arrays: 1.2M-Duo, 1M-Duo, HumanOmni2.5Exome, HumanCoreExome, Human 660W Quad, InfiniumCoreExome-24v1.1, InfiniumOmniExpressExome-8v1.4	4 (a)
Caussy 2019[66]; Adult	17 (16%)	42 (40%)	45 (43%)	0.37	104	0.5626	rs641738 C>T	Taqman assay	5

Chambers 2011[57]; Adult	-	-	-	NS	61089	-	rs641738 C>T	Affymetrix 500k/6.0, Illumina 300K/370K/317K/550K/610K, Perlegen 284K/600K	5
Chen 2020[9]; Adult	GWAS	GWAS	GWAS	0.43	19598	-	rs626283 G>C	Illumina HumanCoreExome v.12.1 array	5
Di Costanzo 2018[68]; Adult	90 (20%)	216 (49%)	139 (31%)	0.44	445	0.8884	rs641738 C>T	Taqman assay	3 (a,c)
Di Costanzo 2019[31]; Paediatric	59 (26%)	104 (45%)	67 (29%)	0.48	230	0.7879	rs641738 C>T	Taqman assay	5
Di Sessa 2018[33]; Paediatric	218 (22%)	494 (49%)	290 (29%)	0.46	1002	0.5535	rs641738 C>T	Taqman assay	5

DiStefano 2015[69]; Adult	396 (21%)	896 (48%)	574 (31%)	0.45	1866	0.7018	rs641738 C>T	Infinium HD Ultra BeadChip assay	5
Donati 2017[59]; Adult	311 (22%)	683 (48%)	437 (31%)	0.46	1431	0.1062	rs641738 C>T	Taqman assay	5
Dongiovanni 2018 (DHS)[50]; Adult	365 (14%)	1181 (44%)	1129 (42%)	0.36	2675	0.3086	rs641738 C>T	Illumina Human Exome BeadChip	5
Dongiovanni 2018 (LBC)[50]; Adult	320 (21%)	684 (45%)	511 (34%)	0.44	1515	0.0378	rs641738 C>T	Taqman assay	5
Gurdasani 2019[70]; Adult	-	-	-	0.29	13116	-	rs641738 C>T	Illumina HumanOmni 2.5M BeadChip, HumanOmni Multi- Ethnic GWAS/Exome Array, Illumina MEGA array & Affymetrix Axiom	5

								Genome-Wide PanAFR Array	
Guzman 2018[71]; Adult	25 (17%)	61 (41%)	62 (42%)	0.38	148	0.2345	rs2576452 C>T	Affymetrix Axiom array	5
Hudert 2019[72]; Paediatric	49 (18%)	137 (51%)	84 (31%)	0.44	270	0.1633	rs641738 C>T	Taqman assay	3 (a,c)
Kanai 2018[36]; Adult	-	-	-	0.22	134182	-	rs641738 C>T	Illumina HumanOmniExpress / HumanExome BeadChips	5
Karajamaki 2019[73]; Adult	136 (15%)	465 (50%)	334 (36%)	0.39	935	0.0314	rs641738 C>T	Taqman assay	5
Kawaguchi 2018[38]; Adult	413 (5%)	2937 (34%)	5222 (61%)	0.22	8572	0.7972	rs641738 C>T	Illumina Human 610-Quad, Illumina Human Omni 2.5-8, and Illumina	4 (a)

								Infinium Core Exome arrays	
Koo 2020[74]; Adult	21 (5%)	161 (35%)	279 (61%)	0.22	461	0.2163	rs641738 C>T	Taqman assay	4 (a)
Krawczyk 2016[55]; Adult	17 (20%)	45 (54%)	22 (26%)	0.47	84	0.7459	rs641738 C>T	Taqman assay	
Krawczyk 2018[43]; Adult	114 (22%)	242 (47%)	159 (31%)	0.46	515	0.3857	rs641738 C>T	Taqman assay	4 (c)
Lin 2018[75]; Paediatric	47 (6%)	274 (33%)	498 (61%)	0.22	819	0.1482	rs641738 C>T	Taqman assay	5
Luukkonen 2020[47]; Adult	129 (16%)	377 (48%)	287 (36%)	0.40	793	0.8673	rs641738 C>T	Taqman assay	5
Mann 2018[76]; Paediatric	88 (23%)	185 (49%)	106 (28%)	0.48	379	0.5991	rs641738 C>T	Taqman assay	5

Mann 2020[77]; Adult	2022 (18%)	5494 (50%)	3418 (31%)	0.44	10934	0.0173	rs641738 C>T	Affymetrix 500K Array Set, Affymetrix Axiom UKBiobank, and Illumina Infinium Core Exome 24v1	5
Middelberg 2011[78]; Adult & paediatric	-	-	-	0.446	11693	-	rs641738 C>T	Illumina 610K, 317K, or 370K	5
Moon 2019[79]; Adult	-	-	-	0.20	6949	-	rs641738 C>T	KoreanChip 833K	5
Prins 2017[80]; Adult	-	-	-	0.442	9731	-	rs641738 C>T	Illumina Human CoreExome v12.1	5
Reichert 2019[81]; Adult	14 (26%)	36 (67%)	4 (7%)	0.59	54	0.0362	rs641738 C>T	Taqman assay	4 (c)
Seidelin 2020[82]; Adult	1427 (19%)	3605 (48%)	2479 (33%)	0.43	7511	0.3836	rs641738 C>T	Taqman assay	5

Sookoian 2018[83]; Adult	101 (16%)	315 (50%)	218 (34%)	0.41	634	0.2263	rs641738 C>T	Taqman assay	5
Speliotes 2011[29]; Adult	-	-	-	0.41	4244	-	rs641738 C>T	Illumina, Affymetrix 500K & Affymetrix 50K, and iPLEX Sequenom MassARRAY arrays	5
Strnad 2018[61]; Adult	143 (21%)	336 (50%)	193 (29%)	0.46	672	0.6813	rs641738 C>T	Taqman assay	5
Tabassum 2019[84]; Adult	-	-	-	0.39	2181	-	rs641738 C>T	Illumina HumanCoreExome BeadChip	5
UKBB 2019[11]; Adult	67183 (19%)	173262 (49%)	113151 (32%)	0.44	353596	0.9742	rs641738 C>T	UK Biobank Axiom Array	5
Umano 2018[85]; Paediatric	151 (17%)	404 (46%)	323 (37%)	0.4	878	0.3777	rs626283 G>C	Taqman assay	4 (c)

Verma 2017[86]; Adult	-	-	-	0.45	31466	-	rs641738 C>T	Illumina Human Omni Express plus Exome beadchip	5
Viitasalo 2016[87]; Paediatric	78 (17%)	232 (50%)	157 (34%)	0.42	467	0.7446	rs641738 C>T	Taqman assay	3 (c,d)
Wattacheril 2017[88]; Paediatric	30 (14%)	89 (43%)	89 (43%)	0.36	208	0.0984	rs641738 C>T	Illumina HumanCNV370-Quadv3 BeadChips	5
Willer 2013[89]; Adult	-	-	-	0.40	347532	-	rs641738 C>T	Illumina iSelect Metabochip	5
Young 2019[90]; Adult	-	-	-	0.34	3555	-	rs641738 C>T	Illumina HumanOmniExpress BeadChip	5
Zusi 2019[91]; Paediatric	100 (20%)	253 (50%)	157 (31%)	0.44	510	0.629	rs641738 C>T	Taqman assay	5

Table S3.

Genotype details and characteristics of included studies. Genotype frequencies for rs641738C>T for each study, with its Hardy-Weinberg Equilibrium p-value (calculated using chi-squared test), where data is available. Mean effect allele frequency (EAF) for T-allele across the whole study. A modified Cochrane Risk-of-Bias Tool (0-5) was used. Letters indicate risk of bias due to a corresponding component of the score: A: Similarity of cases and controls; B: Quality of genotyping; C: Assessment of potential confounders; D: Method of assessing outcome; E: Adequate follow-up. DHS, Dallas Heart Study; GWAS, genome-wide association study; HWE, Hardy Weinberg Equilibrium; LB, liver biopsy; MRI, magnetic resonance imaging; MRS, magnetic resonance spectroscopy; NA, not applicable PDFF, proton-density fat fraction; T2DM, type 2 diabetes mellitus; UKBB, UK BioBank; US, ultrasound scan.

**Study**<br>**Age group**	**Population(s) (country)**	**Type of control**	**Identifi-cation of control**	**Female, n (%)**	**Age**	**T2DM**	**BMI**	**Individual participant-level data available**	**Relevant findings of original study**	**Notes**
Abeysekera 2019[63]; Adult	Non-Finnish European (UK)	Without NAFLD	Cohort study	1,781 (61%)	24 (0.5)	0	24.1 (4.9)	Yes	Not reported in original analysis	-
Adams 2013[64]; Paediatric	Non-Finnish European (Australia)	Without NAFLD	Cohort study	444 (46.8%)	17 (0.24)	0	0.27 (0.94, z-BMI)	Yes	Not reported in original analysis	-
Anstee 2020[65]; Adult	Non-Finnish European (Multiple, Europe)	Without NAFLD	Healthy populati on controls	701 (47.3%) [cases]	50.1 (13.0) [cases]	593 (40%) [cases]	35.2 (7.9) [cases]	Yes	No significant associations observed.	-
Caussy 2019[66]; Adult	Mixed (>80% Caucasian) (USA)	Without NAFLD	Family or sibling controls	70 (67%)	49.5 (18.3)	16 (15%)	27.57 (6.9)	Yes	rs641738C>T not associated with pro-C3 levels	rs641738C>T genotype data only available for a subset of

										study participants who did not undergo liver biopsy, who were family/sibling participants that were screened for NAFLD using MRI-PDFF.
Chambers 2011[57]; Adult	Mixed (>80% Caucasian) (Multiple)	NA	Cohort study	30,698 (50.2%)	52.8 (11.8)	4,661 (7.6%)	26.6 (4.6)	No	Not reported in original analysis	-
Chen 2020[9]; Adult	Non-Finnish European (USA)	Without NAFLD	Cohort study	10,406 (53%)	54.2 (15.9)	4,214 (22%)	29.7 (7.0)	No	rs626283G>C associated with: increased	-

									cirrhosis in UKBB (but not MGI), higher alkaline phosphatase, lower serum triglycerides, and no effect on ALT	
Di Costanzo 2018[68]; Adult	Non-Finnish European (Italy)	Without NAFLD	Healthy blood donors	150 (33.7%)	51.5 (4.5)	57 (13%)	27.1 (1.4)	No	rs641738C>T showed borderline positive association with NAFLD diagnosis using recessive genetic model	-

									only; positively associated with ultrasound-defined severity of hepatic steatosis	
Di Costanzo 2019[31]; Paediatric	Non-Finnish European (Italy)	Without NAFLD	Identified on MRI from same clinic	99 (43%)	10.29 (2.87602)	0	26.17 (4.13)	Yes	rs641738C>T not associated with hepatic fat fraction	-
Di Sessa 2018[33]; Paediatric	Non-Finnish European (Italy)	Without NAFLD	Identified on US from same clinic	466 (46.5%)	10.6 (3.0)	0	3.0 (0.8, z-BMI)	No	rs641738C>T associated with higher ALT and positively contributed to a genetic model	-

									associated with diagnosis of NAFLD	
DiStefano 2015[69]; Adult	Non-Finnish European (USA)	Without NAFLD	Identified on LB from same cohort	1,512 (80.9%)	45 (11)	574 (31%)	47 (8.2)	Yes	Not reported in original analysis	-
Donati 2017[59]; Adult	Non-Finnish European (Italy / UK)	Without NAFLD and NAFLD (histology & HCC analyses)	Identified on US/LB from same clinics	406 (28.3%0	50.9 (10.7)	403 (28.3%	33.9 (7.0)	Yes	rs641738C>T associated with presence of HCC in patient with NAFLD, with larger odds ratio in patients without advanced fibrosis	This study included two independent cohorts (Italian and UK), who have been included in the meta-analysis as separate cohorts

Dongiovanni 2018 (DHS)[50]; Adult	Mixed: Non-Finnish European, African American, Hispanic (USA)	Without NAFLD	Cohort study	1,525 (57%)	45 (11)	321 (12%)	29.4 (6.7)	Yes	rs641738C>T associated with higher hepatic fat fraction on MRS	Dongiovanni 2018 includes data from two separate cohorts: the Dallas Heart Study (DHS) and the Liver Biopsy Cohort (LBC). They are different in design, populations, and outcomes measured therefore have been described and
Dongiovanni 2018 (LBC)[50]; Adult	European (Italy / Finland)	Without NAFLD and NAFLD (histology analyses)	Identified on LB from same clinics	722 (48)	43 (16)	400 (26)	32.1 (9.6)	Yes	rs641738C>T associated with higher degree of steatosis, stage of fibrosis, and degree of lobular inflammation on biopsy	

										analysed separately throughout the meta-analysis.
Gurdasani 2019[70]; Adult	African (Uganda)	NA	Cohort study	8,026 (61%)	45.1 (12.5)	7,827 (60%)	25.6 (3.2)	No	Not reported in original analysis	-
Guzman 2018[71]; Adult	Mixed: Hispanic and non-Hispanic (USA)	Without NAFLD	Identified on MRI from same interventional studies	59 (40%)	57.8 (8.7)	148 (100%)	31.5 (4.8)	Yes	rs2576452C>T in strong LD with rs641738C>T and no 'meaningful' association found between the variant and ALT or HFF	-

Hudert 2019[72]; Paediatric	Non-Finnish European (Germany)	Without NAFLD	Healthy population (adult) controls	92 (34%)	Cases = 14.1 (2.2) Controls = 46.7 (16.0)	0	Cases = 2.76 (.6 (z-BMI))	Yes	rs641738C>T not associated with diagnosis of NAFLD or severity of histology	Data included in histological analyses (e.g. presence of fibrosis) is only from paediatric cases with liver biopsies
Kanai 2018[36]; Adult	East Asian (Japan)	NA	Cohort study	61,455 (46%)	63.2 (13.2)	36,832 (27%)	22.9 (3.6)	No	Not reported in original analysis	-
Karajamaki 2019[73]; Adult	Finnish European (Finland)	Without NAFLD	Identified on US from same cohort	508 (53.0%)	51.2 (6)	97 (10%)	27.7 (4.17)	No	rs641738C>T positively associated with diagnosis of NAFLD in recessive models but not	-

									NAFLD-related mortality.	
Kawaguchi 2018[38]; Adult	East Asian (Japan)	Without NAFLD and NAFLD (histology & HCC analyses)	Healthy population controls	5111 (59.6%)	52.4 (13.8)	209 (2)	22.9 (4.2)	No	rs641738C>T not associated with NAFLD, HCC, or severity of histology	-
Koo 2020[74]; Adult	East Asian (Korea)	Without NAFLD and NAFLD (histology	Healthy individuals undergoing LB for donor	264 (50.3%)	53.2 (14.9)	192 (42)	26.7 (1.4)	No	rs641738C>T not associated with NAFLD or severity of histology, but was associated with lower	-

		analyses)	transplant assessment or non-malignant liver tumours						eGFR (Koo 2019)	
Krawczyk 2016[55]; Adult	Non-Finnish European (Spain)	NAFLD	Identified on LB from same clinics	59 (70.2%)	43.8 (11.5)	24 (28.6%)	46.3 (6.0)	Yes	rs641738C>T not associated with severity of steatosis	Only 7 participants without hepatic steatosis therefore insufficient to be included in analysis of NAFLD diagnosis

Krawczyk 2018[43]; Adult	Non-Finnish European (Germany)	NAFLD	Identified on US / LB from same clinics	171 (58.1%)	49 (18)	79 (26.7%)	32 (13.2)	Yes	rs641738C>T was positively associated with severity of fibrosis using recessive and allelic contrast models, and positively associated with hepatic steatosis on multivariable (but not univariable) analysis	-
Lin 2018[75];	East Asian (Taiwan)	Without NAFLD	Cohort study	257 (31.4%)	11.5 (2.2)	0	26.9 (3.9)	No	rs641738C>T not associated	-

Paediatric									with NAFLD or serum CK-18 fragment levels	
Luukkonen 2020[47]; Adult	Finnish European (Finland)	Without NAFLD and NAFLD (histology analyses)	Identified on LB from same cohort	482 (61%)	46.8 (11.8)	196 (25%)	34.1 (6.9)	Yes	rs641738C>T positively associated with fibrosis stage	Data available from two separate cohorts: individuals attending liver/obesity clinics undergoing MRS and those with LB undergoing bariatric surgery. Where

										possible this data has been analysed separately.
Mann 2018[76]; Paediatric	Non-Finnish European (Italy)	Without NAFLD and NAFLD (histology analyses)	Identified on US from same clinics	165 (44%)	12.06 (3.07)	48 (13%)	1.83 (0.8 (z-BMI))	Yes	rs641738C>T not associated with NAFLD or severity of histology	-
Mann 2020[77]; Adult	Non-Finnish European (United Kingdom)	Without NAFLD	Cohort study	5,823 (53.2%)	49 (7.5)	280 (2.6%)	26.9 (4.8)	Yes	rs641738C>T not associated with NAFLD or ALT but positively associated with	-

									serum alkaline phosphatase.	
Middelberg 2011[78]; Adult & paediatric	Non-Finnish European (Australia)	NA	Cohort study	7,019 (60%)	40.0 (10.5)	-	24.2 (4.3)	No	Not reported in original analysis	-
Moon 2019[79]; Adult	East Asian (Korea)	NA	Cohort study	3499 (50.4%)	52.1 (89)	609 (8.8%)	24.6 (3.2)	No	Not reported in original analysis	-
Prins 2017[80]; Adult	Non-Finnish European (United Kingdom)	NA	Cohort study	5,445 (56%)	52.4 (18.5)	234 (2.4%)	28.1 (4.9)	No	Not reported in original analysis	-
Reichert 2019[81]; Adult	Non-Finnish European (Germany)	NAFLD	Identified on LB from same clinics	24 (42.1%)	61 (11.1)	34 (63%)	-	Yes	Not reported in original analysis	-

Seidelin 2020[82]; Adult	Non-Finnish European (Denmark)	NA	Cohort study	4,131 (55%)	58 (13.4)	451 (6%)	26 (4.3)	No	Not reported in original analysis	-
Sookoian 2018[83]; Adult	Latino (Argentina)	Without NAFLD and NAFLD (histology analyses)	Identified on US from same clinics	360 (57%)	49.7 (7.8)	342 (54%)	33.5 (6.5)	No	rs641738C>T not associated with NAFLD or severity of histology	-
Speliotes 2011[29]; Adult	Non-Finnish European (USA, Iceland, Europe)	NA	Cohort study	2,317 (55%)	62.9 (8.7)	437 (10.3%)	27.5 (4.9)	No	Not reported in original analysis	-
Strnad 2018[61]; Adult	Non-Finnish European	NAFLD	Identified on LB from	336 (50%)	49 (14)	215 (32%)	37 (12)	Yes	rs641738C>T not affect associations	-

	(Germany, Austria, & Switzerland)		same clinics						observed when included as a covariate	
Tabassum 2019[84]; Adult	Finnish European (Finland)	NA	Cohort study	1157 (53%)	46.1 (14.2)	210 (9.6%)	26.3 (4.6)	No	Not reported in original analysis (different variants in *MBOAT7* were GWAS-significant for specific lipid species)	-
UKBB 2019[11]; Adult	Non-Finnish European (UK)	Without NAFLD and NAFLD (coded fibrosis	Cohort study	192,370 (54%)	56.5 (8.1)	19,878 (5.6%)	27.3 (4.8)	No	rs641738C>T positively associated with all-cause cirrhosis (Emdin 2020); not	See Supplementary Methods for details of articles using UKBB data.

		analysis)							reported in other original analyses	
Umano 2018[85]; Paediatric	Mixed: Non-Finnish European, African American, Hispanic (USA)	Without NAFLD	Identified on MRI from same clinics	520 (59.2%)	13.4 (3.4)	35 (4%)	2.16 (.07 (z-BMI))	Yes	rs626283G>C positively associated with hepatic fat in and insulin resistance in Caucasians only	-
Verma 2017[86]; Adult	Mixed (USA)	NA	Cohort study	18,217 (58%)	60.7 (17.7)	9,362 (29.8%)	31.0 (14.9)	No	Not reported in original analysis	-
Viitasalo 2016[87]; Paediatric	Finnish European (Finland)	NA	Cohort study	222 (47.5%)	7.6 (0.4)	0	-.19 (1.1 (z-BMI))	No	rs641738C>T positively associated with serum ALT	-

Wattacheril 2017[88]; Paediatric	Hispanic (USA)	NAFLD	Identified on LB from same clinics	0 (all male)	12.0 (2.2)	4 (2%)	2.4 (.37 (z-BMI))	Yes	Not reported in original analysis	-
Willer 2013[89]; Adult	Mixed (>80% Caucasian) (Multiple)	NA	Cohort study	166,898 (48%)	55.2 (10.8)	-	-	No	Not reported in original analysis	-
Young 2019[90]; Adult	Hispanic (USA)	NA	Cohort study	2273 (64%)	36.8 (10.8)	0	29.0 (5.7)	Yes	Not reported in original analysis	-
Zusi 2019[91]; Paediatric	Non-Finnish European (Italy)	Without NAFLD	Identified on US from same clinics	235 (46%)	11.2 (2.8)	0	3.3 (0.8 (z-BMI))	Yes	rs641738C>T not associated with NAFLD	-

Table S4.

Additional characteristics and findings of included studies. BMI is given in kg/m^2 for adult studies and, where available, given as an age-/sex-adjusted z-score for children. BMI, body mass index; DHS, Dallas Heart Study; GWAS, genome-wide association study; LB, liver biopsy; MRI, magnetic resonance imaging; MRS, magnetic resonance spectroscopy; NA, not applicable PDFF, proton-density fat fraction; T2DM, type 2 diabetes mellitus; UKBB, UK BioBank; US, ultrasound scan.

Meta-analyses	Study detail	No. studies	No. cases	No. controls	Genetic model	Overall	Caucasian only
						OR [95% CI]	OR [95% CI]
Xia 2019[92]; Adult	Meta-analysis of diagnosis of NAFL(D)/NASH from case-control studies	5	2560	8738	CC vs. TT	0.91 [0.75, 1.11]	0.75 [0.54, 1.05]
					CC vs. CT+TT	0.95 [0.87, 1.04]	0.97 [0.79, 1.20]
					CC+CT vs. TT	0.91 [0.76, 1.10]	0.75 [0.55, 1.01]
**Systematic reviews**	**Study detail**	**No. studies**	**Principle findings**				
Ismaiel 2020[93]; Adult	Systematic review of effect of rs641738C>T on MAFLD	22	In Caucasian, Hispanic, African American individuals: rs641738C>T associated with reduced hepatic expression of MBOAT7 and positively associated with hepatic fat content, NASH, advanced fibrosis, and HCC.<br>rs641738C>T not associated with features of MAFLD in Asian populations.<br>rs611738C>T positively associated with ALT in children but limited data on histology.				

	plus associated biochemical and cardiovascular phenotypes		No associations observed with coronary artery disease but limited data.

Table S5.

Characteristics of previous systematic reviews & meta-analyses on rs641738C>T. One previous meta-analysis was identified, which assessed the association with diagnosis of NAFLD across a range of genetic models. No significant associations were identified by this meta-analysis. MAFLD, metabolic-dysfunction-associated fatty liver disease.

Outcome	Genetic model	Sub-analysis	No. of studies	Heterogeneity		Effect summary	
				I^2	p_Q	Beta regression coefficient [95% CI]	p_Z
Liver fat (continuous)	Additive	Caucasian	6	0.00	0.83	.03 (.02, .05)	4.83E-05
Liver fat (continuous)	Additive	Non-Caucasian	3	0.31	0.24	.04 (-.04, .12)	.34
Liver fat (continuous)	Additive	Overall	9	0.00	0.72	.04 (.02, .05)	1.20E-05
**Outcome**	**Genetic model**	**Sub-analysis**	**No. of studies**	**Heterogeneity**		**Effect summary**	
				**I^2**	**p_Q**	**Mean difference [95% CI]**	**p_Z**
Liver fat (continuous)	Dominant	Caucasian	5	0.00	0.57	.18 (.02, .34)	0.04
Liver fat (continuous)	Dominant	Non-Caucasian	2	0.72	0.06	-.31 (-7.7, 7.09)	0.69
Liver fat (continuous)	Dominant	Overall	7	0.12	0.34	.16 (-.04, .36)	0.10
Liver fat (continuous)	Recessive	Caucasian	5	0.20	0.29	.27 (-.1, .64)	0.11
Liver fat (continuous)	Recessive	Non-Caucasian	2	0.00	0.56	.2 (-2.08, 2.47)	0.47
Liver fat (continuous)	Recessive	Overall	7	0.00	0.50	.21 (.03, .39)	0.03
**Outcome**		**Sub-analysis**		**Heterogeneity**		**Effect summary**	

	Genetic model		No. of studies	I^2	p_Q	Beta regression coefficient [95% CI]	p_Z
Liver fat (semi-quantitative)	Additive	Overall	3	0.00	0.57	.02 (-.002, .04)	.08
**Outcome**	**Genetic model**	**Sub-analysis**	**No. of studies**	**Heterogeneity**		**Effect summary**	
				I^2	p_Q	**Mean difference [95% CI]**	p_Z
Liver fat (semi-quantitative)	Dominant	Overall	3	0.13	0.32	.02 (-.09, .13)	.49
Liver fat (semi-quantitative)	Recessive	Overall	3	0.77	0.01	.05 (-.34, .43)	.66

Table S6.

Meta-analyses for liver fat in adults. Each outcome was tested using dominant (CC vs. CT+TT), recessive, (CC+CT vs. TT), and additive (CC vs. CT vs. TT) models of inheritance with sub-analysis by studies with Caucasian and non-Caucasian populations. Heterogeneity is quantified using I^2. Effect summaries for additive models are pooled beta regression coefficients. Effect summaries for recessive and dominant models are mean differences. Effect summary units represent standardised (mean=0, standard deviation=1) inverse-transformed change in liver fat, except for recessive and dominant models for liver fat (continuous), where mean difference in hepatic fat fraction (%) is given. The p-value for the effect summary (p_Z) should be assessed against a critical p-value of 0.017, due to testing three genetic models for each dichotomous outcome.

Outcome	Genetic model	Sub-analysis	No. of studies	Heterogeneity		Effect summary	
				I^2	p_Q	OR [95% CI]	p_Z
NAFLD diagnosis (control vs NAFLD)	Additive	Caucasian	12	0.00	0.97	1.07 (.98, 1.17)	0.125
NAFLD diagnosis (control vs NAFLD)	Additive	Non-Caucasian	5	0.00	0.96	1.03 (.82, 1.29)	0.784
NAFLD diagnosis (control vs NAFLD)	Additive	Overall	17	0.00	1.00	1.06 (.98, 1.15)	0.126
NAFLD diagnosis (control vs NAFLD)	Dominant	Caucasian	12	0.50	0.03	1.08 (.97, 1.19)	0.158
NAFLD diagnosis (control vs NAFLD)	Dominant	Non-Caucasian	5	0.00	0.43	1.02 (.92, 1.13)	0.732
NAFLD diagnosis (control vs NAFLD)	Dominant	Overall	17	0.38	0.05	1.05 (.97, 1.14)	0.210
NAFLD diagnosis (control vs NAFLD)	Recessive	Caucasian	12	0.38	0.09	1.17 (1.05, 1.3)	0.0033
NAFLD diagnosis (control vs NAFLD)	Recessive	Non-Caucasian	5	0.00	0.46	1.1 (.9, 1.34)	0.343

NAFLD diagnosis (control vs NAFLD)	Recessive	Overall	17	0.25	0.17	1.15 (1.05, 1.26)	0.0018
Severe steatosis (S1-2 vs S3)	Additive	Caucasian	7	0.00	0.57	1.06 (.86, 1.31)	0.597
Severe steatosis (S1-2 vs S3)	Additive	Non-Caucasian	1	NA	NA	.99 (.32, 3.1)	0.986
Severe steatosis (S1-2 vs S3)	Additive	Overall	8	0.00	0.69	1.06 (.86, 1.3)	0.605
Severe steatosis (S1-2 vs S3)	Dominant	Caucasian	7	0.73	0.00	1.14 (.81, 1.59)	0.460
Severe steatosis (S1-2 vs S3)	Dominant	Non-Caucasian	1	NA	NA	.94 (.58, 1.53)	0.795
Severe steatosis (S1-2 vs S3)	Dominant	Overall	8	0.69	0.00	1.11 (.82, 1.49)	0.503
Severe steatosis (S1-2 vs S3)	Recessive	Caucasian	7	0.72	0.00	1.08 (.76, 1.54)	0.676
Severe steatosis (S1-2 vs S3)	Recessive	Non-Caucasian	1	NA	NA	1.11 (.39, 3.16)	0.852

Severe steatosis (S1-2 vs S3)	Recessive	Overall	8	0.67	0.00	1.08 (.78, 1.5)	0.642
NASH (NAFL vs NASH)	Additive	Caucasian	6	0.00	0.87	1.07 (.9, 1.27)	0.467
NASH (NAFL vs NASH)	Additive	Non-Caucasian	3	0.00	0.98	1.11 (.69, 1.79)	0.675
NASH (NAFL vs NASH)	Additive	Overall	9	0.00	0.98	1.07 (.91, 1.26)	0.408
NASH (NAFL vs NASH)	Dominant	Caucasian	6	0.19	0.29	1.11 (.96, 1.27)	0.159
NASH (NAFL vs NASH)	Dominant	Non-Caucasian	3	0.00	0.97	1.1 (.89, 1.36)	0.390
NASH (NAFL vs NASH)	Dominant	Overall	9	0.00	0.62	1.1 (.99, 1.22)	0.085
NASH (NAFL vs NASH)	Recessive	Caucasian	6	0.53	0.06	1.14 (.93, 1.41)	0.213
NASH (NAFL vs NASH)	Recessive	Non-Caucasian	3	0.00	0.58	1.24 (.81, 1.9)	0.324
NASH (NAFL vs NASH)	Recessive	Overall	9	0.33	0.15	1.14 (.96, 1.36)	0.128
Any fibrosis (F0 vs F1-4)	Additive	Caucasian	7	0.00	0.77	1.07 (.91, 1.26)	0.390
Any fibrosis (F0 vs F1-4)	Additive	Non-Caucasian	2	0.00	0.90	1.19 (.64, 2.2)	0.585
Any fibrosis (F0 vs F1-4)	Additive	Overall	9	0.00	0.90	1.08 (.92, 1.26)	0.332
Any fibrosis (F0 vs F1-4)	Dominant	Caucasian	7	0.57	0.03	1.08 (.91, 1.29)	0.384
Any fibrosis (F0 vs F1-4)	Dominant	Non-Caucasian	2	0.00	0.36	1.0 (.8, 1.25)	0.993
Any fibrosis (F0 vs F1-4)	Dominant	Overall	9	0.47	0.06	1.06 (.92, 1.23)	0.387
Any fibrosis (F0 vs F1-4)	Recessive	Caucasian	7	0.51	0.06	1.19 (.99, 1.45)	0.068

Any fibrosis (F0 vs F1-4)	Recessive	Non-Caucasian	2	0.00	0.82	2.14 (1.2, 3.84)	0.0105
Any fibrosis (F0 vs F1-4)	Recessive	Overall	9	0.52	0.03	1.27 (1.04, 1.54)	0.0183
Advanced fibrosis (F0-2 vs F3-4)	Additive	Caucasian	6	0.00	0.94	1.07 (.85, 1.34)	0.552
Advanced fibrosis (F0-2 vs F3-4)	Additive	Non-Caucasian	2	0.00	0.91	1.0 (.5, 2.01)	0.998
Advanced fibrosis (F0-2 vs F3-4)	Additive	Overall	8	0.00	0.99	1.06 (.86, 1.32)	0.572
Advanced fibrosis (F0-2 vs F3-4)	Dominant	Caucasian	6	0.34	0.18	1.02 (.83, 1.26)	0.858
Advanced fibrosis (F0-2 vs F3-4)	Dominant	Non-Caucasian	2	0.00	0.96	1.01 (.76, 1.34)	0.935
Advanced fibrosis (F0-2 vs F3-4)	Dominant	Overall	8	0.08	0.37	1.0 (.87, 1.16)	0.974
Advanced fibrosis (F0-2 vs F3-4)	Recessive	Caucasian	6	0.00	0.50	1.22 (1.03, 1.45)	0.0206
Advanced fibrosis (F0-2 vs F3-4)	Recessive	Non-Caucasian	2	0.00	0.64	.96 (.5, 1.85)	0.911

Advanced fibrosis (F0-2 vs F3-4)	Recessive	Overall	8	0.00	0.65	1.2 (1.02, 1.42)	0.027
HCC (NAFLD-HCC vs NAFLD no-HCC)	Additive	Overall	4	0.00	1.00	1.41 (.86, 2.29)	0.170
HCC (NAFLD-HCC vs NAFLD no-HCC)	Dominant	Overall	4	0.00	0.65	1.64 (1.18, 2.27)	0.0031
HCC (NAFLD-HCC vs NAFLD no-HCC)	Recessive	Overall	4	0.00	0.95	1.4 (.99, 1.98)	0.056

Table S7.

Full results of meta-analyses for all dichotomous outcomes in adults. Each outcome was tested using dominant (CC vs. CT+TT), recessive, (CC+CT vs. TT), and additive (CC vs. CT vs. TT) models of inheritance and, where there were sufficient studies, with sub-analysis by studies with Caucasian and non-Caucasian populations. Heterogeneity is quantified using I^2 and odds ratios (OR) are given with 95% confidence intervals (CI). The p-value for the effect summary (p_z) should be assessed against a critical p-value of 0.017, due to testing three genetic models for each dichotomous outcome. HCC, hepatocellular carcinoma.

Outcome	Population	No. studies	Heterogeneity		Effect summary	
			I^2 (%)	p_Q	Beta [95%CI]	p_z
Alanine aminotransferase	Overall	13	0	0.71	0.0041 [0.0015; 0.0067]	0.0021
Alanine aminotransferase	Non-Caucasian	7	0	0.97	0.0036 [-0.0042; 0.0113]	0.36
Alanine aminotransferase	Caucasian	6	34	0.18	0.0042 [0.0002; 0.0082]	0.038
High density lipoprotein	Overall	11	56.9	0.01	0.0092 [0.0013; 0.0171]	0.022
High density lipoprotein	Non-Caucasian	6	58	0.04	0.0081 [-0.0067; 0.0229]	0.28
High density lipoprotein	Caucasian	5	54	0.07	0.0112 [0.0016; 0.0208]	0.022
Insulin	Overall	3	50.1	0.13	0.0088 [-0.0264; 0.0440]	0.62
Low density lipoprotein	Overall	11	50.4	0.03	0.0050 [-0.0015; 0.0114]	0.13
Low density lipoprotein	Non-Caucasian	6	49	0.08	0.0000 [-0.0141; 0.0142]	1.00
Low density lipoprotein	Caucasian	5	55	0.06	0.0074 [0.0002; 0.0146]	0.044
Cholesterol (total)	Overall	9	29.7	0.18	0.0054 [0.0001; 0.0106]	0.045
Cholesterol (total)	Non-Caucasian	5	49	0.10	-0.0005 [-0.0197; 0.0186]	0.96
Cholesterol (total)	Caucasian	4	0	0.58	0.0069 [0.0033; 0.0106]	0.00021
Triglycerides	Overall	12	38.8	0.08	-0.0117 [-0.0178; -0.0056]	0.00015
Triglycerides	Non-Caucasian	6	64	0.02	-0.0163 [-0.0365; 0.0038]	0.11
Triglycerides	Caucasian	6	0	0.62	-0.0105 [-0.0141; -0.0068]	1.49E-08

Table S7.

Summary of results from meta-analyses for serum biochemical traits using data from genome-wide association studies. Meta-analyses were performed using random effects for all studies (‘Overall’) with subgroup analyses for Caucasian and Non-Caucasian populations. [Except for insulin, where there was insufficient data to perform a sub-analysis by population.) Beta represents the change in logarithmically-transformed outcome measure per T-allele from rs641738C>T as calculated using linear regression. CI, confidence interval; HDL, high-density lipoprotein cholesterol; LDL, low-density lipoprotein cholesterol.

Outcome	Study	Cohort	Ethnicity	Beta	pval	num	Transformation
ALT	Chambers 2011	Chambers LFT meta-analysis	Caucasian	3.20E-03	1.76E-01	61089	Log
ALT	Chen 2020	Michigan Genomics Initiative	Caucasian	9.00E-03	3.92E-01	19598	Log
ALT	Gurdasani 2019	Uganda Genome Resource	Non-Caucasian	7.74E-03	8.41E-01	9401	Log
ALT	Kanai 2018	BioBank Japan	Non-Caucasian	4.53E-03	3.22E-01	134182	Log
ALT	Middelberg 2011	NSW twin / sibling cohorts	Caucasian	4.00E-03	7.60E-01	11693	Log
ALT	Moon 2019	Korea BioBank	Non-Caucasian	-2.93E-04	9.78E-01	6949	Log
ALT	Prins 2017	UKHLS	Caucasian	-5.05E-04	8.47E-01	9731	Log
ALT	UKBB (Neale)	UKBB (Neale summary stats)	Caucasian	1.84E-01	2.04E-08	344136	None
ALT	UKBB 2019 (African)	UKBB (GBE summary stats)	Non-Caucasian	9.74E-03	6.23E-01	5882	Log

ALT	UKBB 2019 (British white)	UKBB (GBE summary stats)	Caucasian	9.25E-03	2.66E-04	316157	Log
ALT	UKBB 2019 (East Asian)	UKBB (GBE summary stats)	Non-Caucasian	-4.74E-02	3.69E-01	1046	Log
ALT	UKBB 2019 (Non-British White)	UKBB (GBE summary stats)	Caucasian	4.45E-03	6.34E-01	23327	Log
ALT	UKBB 2019 (South Asian)	UKBB (GBE summary stats)	Non-Caucasian	2.65E-04	9.87E-01	7184	Log
ALT	Verma 2017	Geisinger EHR	Mixed	1.38E+00	1.65E-01	31466	None
ALT	Young 2019	GUARDIAN study	Non-Caucasian	2.32E-02	8.12E-01	3555	Log
Cholesterol (total)	Gurdasani 2019	Uganda Genome Resource	Non-Caucasian	1.14E-02	4.77E-01	13116	Log
Cholesterol (total)	Kanai 2018	BioBank Japan	Non-Caucasian	1.95E-03	6.78E-01	128305	Log
Cholesterol (total)	Prins 2017	UKHLS	Caucasian	-1.00E-02	5.24E-01	9731	Log

Cholesterol (total)	UKBB 2019 (African)	UKBB (GBE summary stats)	Non-Caucasian	2.87E-02	1.48E-01	5879	Log
Cholesterol (total)	UKBB 2019 (British white)	UKBB (GBE summary stats)	Caucasian	6.15E-03	1.54E-02	316264	Log
Cholesterol (total)	UKBB 2019 (East Asian)	UKBB (GBE summary stats)	Non-Caucasian	-3.61E-02	4.94E-01	1047	Log
Cholesterol (total)	UKBB 2019 (Non-British White)	UKBB (GBE summary stats)	Caucasian	1.44E-02	1.24E-01	23339	Log
Cholesterol (total)	UKBB 2019 (South Asian)	UKBB (GBE summary stats)	Non-Caucasian	-3.58E-02	3.10E-02	7196	Log
Cholesterol (total)	UKBB (Neale)	UKBB (Neale summary stats)	Caucasian	4.90E-03	6.71E-02	344278	None
Cholesterol (total)	Willer 2013	GLGC GWAS	Caucasian	7.75E-03	7.93E-03	347532	Log
HDL	Gurdasani 2019	Uganda Genome Resource	Non-Caucasian	-7.73E-03	6.43E-01	13114	Log
HDL	Kanai 2018	BioBank Japan	Non-Caucasian	6.44E-03	3.05E-01	70657	Log

HDL	Middelberg 2011	NSW twin / sibling cohorts	Caucasian	-1.00E-02	4.70E-01	11693	Log
HDL	Moon 2019	Korea BioBank	Non-Caucasian	2.98E-03	5.43E-01	6949	Log
HDL	Prins 2017	UKHLS	Caucasian	4.00E-02	1.65E-02	9731	Log
HDL	UKBB 2019 (African)	UKBB (GBE summary stats)	Non-Caucasian	3.56E-02	8.34E-02	5448	Log
HDL	UKBB 2019 (British white)	UKBB (GBE summary stats)	Caucasian	1.20E-02	6.50E-06	289389	Log
HDL	UKBB 2019 (East Asian)	UKBB (GBE summary stats)	Non-Caucasian	1.65E-01	2.44E-03	953	Log
HDL	UKBB 2019 (Non-British White)	UKBB (GBE summary stats)	Caucasian	-2.45E-03	8.02E-01	21320	Log
HDL	UKBB 2019 (South Asian)	UKBB (GBE summary stats)	Non-Caucasian	-2.69E-03	8.77E-01	6563	Log
HDL	UKBB (Neale)	UKBB (Neale summary stats)	Caucasian	3.67E-03	3.44E-05	315133	None
HDL	Willer 2013	GLGC GWAS	Caucasian	1.78E-02	1.21E-03	188577	Log

Insulin	Lyssenko 2009	FUSION exome chip analysis	Caucasian	6.92E-02	6.95E-02	3400	Log
Insulin	Middelberg 2011	NSW twin / sibling cohorts	Caucasian	5.00E-03	8.60E-01	11693	Log
Insulin	Scott 2012	MAGIC GWAS	Caucasian	-6.20E-03	9.88E-02	5318	Log
LDL	Gurdasani 2019	Uganda Genome Resource	Non-Caucasian	9.93E-03	5.43E-01	13086	Log
LDL	Kanai 2018	BioBank Japan	Non-Caucasian	1.19E-02	5.63E-02	72866	Log
LDL	Middelberg 2011	NSW twin / sibling cohorts	Caucasian	1.30E-02	3.60E-01	11693	Log
LDL	Moon 2019	Korea BioBank	Non-Caucasian	-8.88E-03	1.88E-01	6949	Log
LDL	Prins 2017	UKHLS	Caucasian	-1.00E-02	3.27E-01	9731	Log
LDL	UKBB 2019 (African)	UKBB (GBE summary stats)	Non-Caucasian	1.87E-02	3.47E-01	5869	Log
LDL	UKBB 2019 (British white)	UKBB (GBE summary stats)	Caucasian	3.56E-03	1.61E-01	315682	Log

LDL	UKBB 2019 (East Asian)	UKBB (GBE summary stats)	Non-Caucasian	-3.89E-02	4.61E-01	1047	Log
LDL	UKBB 2019 (Non-British White)	UKBB (GBE summary stats)	Caucasian	1.91E-02	4.08E-02	23294	Log
LDL	UKBB 2019 (South Asian)	UKBB (GBE summary stats)	Non-Caucasian	-2.73E-02	1.01E-01	7179	Log
LDL	UKBB (Neale)	UKBB (Neale summary stats)	Caucasian	2.22E-03	2.84E-01	343621	None
LDL	Willer 2013	GLGC GWAS	Caucasian	1.17E-02	9.82E-05	347532	Log
Triglycerides	Gurdasani 2019	Uganda Genome Resource	Non-Caucasian	2.39E-02	2.81E-01	13115	Log
Triglycerides	Kanai 2018	BioBank Japan	Non-Caucasian	-1.47E-02	4.40E-03	105597	Log
Triglycerides	Middelberg 2011	NSW twin / sibling cohorts	Caucasian	-1.20E-02	4.00E-01	11693	Log
Triglycerides	Moon 2019	Korea BioBank	Non-Caucasian	3.19E-03	7.75E-01	6949	Log
Triglycerides	Prins 2017	UKHLS	Caucasian	-3.00E-02	2.42E-02	9731	Log

Triglycerides	Tabassum 2019	FinnMet	Caucasian	-1.30E-02	6.90E-01	2181	Log
Triglycerides	UKBB 2019 (African)	UKBB (GBE summary stats)	Non-Caucasian	-2.57E-02	1.97E-01	5879	Log
Triglycerides	UKBB 2019 (British white)	UKBB (GBE summary stats)	Caucasian	-9.92E-03	9.32E-05	316011	Log
Triglycerides	UKBB 2019 (East Asian)	UKBB (GBE summary stats)	Non-Caucasian	-1.31E-01	1.29E-02	1046	Log
Triglycerides	UKBB 2019 (Non-British White)	UKBB (GBE summary stats)	Caucasian	-1.99E-04	9.83E-01	23317	Log
Triglycerides	UKBB 2019 (South Asian)	UKBB (GBE summary stats)	Non-Caucasian	-4.43E-02	7.64E-03	7190	Log
Triglycerides	UKBB (Neale)	UKBB (Neale summary stats)	Caucasian	-9.15E-03	1.60E-04	343992	None
Triglycerides	Willer 2013	GLGC GWAS	Caucasian	-1.12E-02	1.53E-04	347532	Log

Table S8.

Summary statistics of genome-wide association studies (GWAS) included in the meta-analysis. Only log-transformed outcomes were included in pooled effect estimates. ALT, alanine aminotransferase; EHR, electronic health records; GBE, Global BioBank

Engine; GLGC, Global Lipids Genetics Consortium; HDL, high-density lipoprotein; LDL, low-density lipoprotein; LFT, liver function test; NSW, New South Wales (Australia); UKBB, UK BioBank; UKHLS, UK Household Longitudinal Study.

Outcome	Genetic model	Sub-analysis	No. of studies	Heterogeneity		Effect summary	
				I^2	p_Q	Beta regression coefficient [95% CI]	p_Z
ALT (IU/L)	Additive	Overall	15	0.00	0.63	.09 (-.18, .36)	0.54
ALT (IU/L)	Additive	Non-Caucasian	5	0.06	0.38	.12 (-.31, .56)	0.57
ALT (IU/L)	Additive	Caucasian	10	0.00	0.59	.06 (-.31, .42)	0.76
HDL (mmol/L)	Additive	Overall	12	0.00	0.95	-.004 (-.01, .004)	0.32
HDL (mmol/L)	Additive	Non-Caucasian	3	0.00	0.90	-.01 (-.03, .01)	0.27
HDL (mmol/L)	Additive	Caucasian	9	0.00	0.88	-.003 (-.01, .01)	0.55
Insulin (pmol/L)	Additive	Overall	7	0.00	0.84	-.56 (-1.49, .37)	0.24
Insulin (pmol/L)	Additive	Non-Caucasian	1	NA	NA	-2.1 (-9.5, 5.3)	0.58
Insulin (pmol/L)	Additive	Caucasian	6	0.00	0.76	-.53 (-1.47, .4)	0.26
LDL (mmol/L)	Additive	Overall	10	0.30	0.17	.004 (-.03, .04)	0.79
LDL (mmol/L)	Additive	Non-Caucasian	2	0.00	0.56	-.03 (-.08, .02)	0.25
LDL (mmol/L)	Additive	Caucasian	8	0.29	0.19	.01 (-.02, .05)	0.46
Total cholesterol (mmol/L)	Additive	Overall	16	0.14	0.29	.003 (-.02, .03)	0.83
Total cholesterol (mmol/L)	Additive	Non-Caucasian	5	0.45	0.12	-.01 (-.06, .04)	0.82

Total cholesterol (mmol/L)	Additive	Caucasian	11	0.00	0.48	-.001 (-.02, .02)	0.91
Triglycerides (mmol/L)	Additive	Overall	16	0.00	0.87	-.03 (-.05, -.01)	0.0009
Triglycerides (mmol/L)	Additive	Non-Caucasian	5	0.00	0.47	-.05 (-.08, -.02)	0.0019
Triglycerides (mmol/L)	Additive	Caucasian	11	0.00	0.97	-.02 (-.04, .001)	0.062
**Outcome**	**Genetic model**	**Sub-analysis**	**No. of studies**	**Heterogeneity**		**Effect summary**	
				**I^2**	**p_Q**	**MD [95% CI]**	**p_Z**
ALT (IU/L)	Dominant	Overall	15	0.21	0.22	.11 (-.48, .71)	0.69
ALT (IU/L)	Dominant	Non-Caucasian	5	0.00	0.79	-.001 (-.47, .47)	1.00
ALT (IU/L)	Dominant	Caucasian	10	0.44	0.07	.69 (-.8, 2.18)	0.32
HDL (mmol/L)	Dominant	Overall	12	0.00	1.00	.002 (-.004, .01)	0.46
HDL (mmol/L)	Dominant	Non-Caucasian	3	0.00	0.63	-.004 (-.04, .04)	0.69
HDL (mmol/L)	Dominant	Caucasian	9	0.00	1.00	.004 (-.001, .01)	0.14
Insulin (pmol/L)	Dominant	Overall	7	0.00	0.98	-1.38 (-2.11, -.65)	0.0036
Insulin (pmol/L)	Dominant	Non-Caucasian	1	NA	NA	-2.3 (-10.8, 6.12)	0.59
Insulin (pmol/L)	Dominant	Caucasian	6	0.00	0.96	-1.36 (-2.19, -.52)	0.009
LDL (mmol/L)	Dominant	Overall	10	0.37	0.11	-.01 (-.07, .05)	0.80
LDL (mmol/L)	Dominant	Non-Caucasian	2	0.00	0.67	-.06 (-.26, .14)	0.17
LDL (mmol/L)	Dominant	Caucasian	8	0.36	0.14	.01 (-.07, .09)	0.82

Total cholesterol (mmol/L)	Dominant	Overall	16	0.00	0.48	.004 (-.02, .03)	0.77
Total cholesterol (mmol/L)	Dominant	Non-Caucasian	5	0.32	0.21	-.003 (-.07, .07)	0.90
Total cholesterol (mmol/L)	Dominant	Caucasian	11	0.00	0.65	-.01 (-.04, .03)	0.60
Triglycerides (mmol/L)	Dominant	Overall	16	0.49	0.01	-.02 (-.07, .03)	0.41
Triglycerides (mmol/L)	Dominant	Non-Caucasian	5	0.00	0.43	-.07 (-.12, -.02)	0.014
Triglycerides (mmol/L)	Dominant	Caucasian	11	0.56	0.01	.02 (-.08, .11)	0.69
ALT (IU/L)	Recessive	Overall	15	0.00	0.59	.23 (-.27, .74)	0.34
ALT (IU/L)	Recessive	Non-Caucasian	5	0.59	0.04	.58 (-1.5, 2.7)	0.49
ALT (IU/L)	Recessive	Caucasian	10	0.00	0.99	.33 (-.06, .71)	0.085
HDL (mmol/L)	Recessive	Overall	12	0.33	0.13	-.03 (-.06, -.0001)	0.050
HDL (mmol/L)	Recessive	Non-Caucasian	3	0.00	0.81	-.04 (-.08, -.0004)	0.049
HDL (mmol/L)	Recessive	Caucasian	9	0.46	0.06	-.03 (-.07, .01)	0.16
Insulin (pmol/L)	Recessive	Overall	7	0.15	0.31	1.2 (-5.8, 8.1)	0.69
Insulin (pmol/L)	Recessive	Non-Caucasian	1	NA	NA	-3.4 (-22.3, 15.5)	0.72
Insulin (pmol/L)	Recessive	Caucasian	6	0.28	0.22	2.3 (-7.9, 12.5)	0.59
LDL (mmol/L)	Recessive	Overall	10	0.00	0.68	.03 (-.01, .06)	0.10
LDL (mmol/L)	Recessive	Non-Caucasian	2	0.00	0.57	-.003 (-.4, .39)	0.95
LDL (mmol/L)	Recessive	Caucasian	8	0.00	0.55	.03 (-.01, .07)	0.11

Total cholesterol (mmol/L)	Recessive	Overall	16	0.10	0.34	.01 (-.04, .06)	0.68
Total cholesterol (mmol/L)	Recessive	Non-Caucasian	5	0.00	0.40	.02 (-.08, .13)	0.60
Total cholesterol (mmol/L)	Recessive	Caucasian	11	0.21	0.25	.004 (-.07, .08)	0.92
Triglycerides (mmol/L)	Recessive	Overall	16	0.00	0.95	-.01 (-.04, .02)	0.41
Triglycerides (mmol/L)	Recessive	Non-Caucasian	5	0.00	0.99	.02 (-.01, .04)	0.20
Triglycerides (mmol/L)	Recessive	Caucasian	11	0.00	0.77	-.02 (-.05, .02)	0.31

Table S9.

Summary of results from meta-analysis of association between rs641738C>T on serum biochemical parameters in adults. Sub-analysis was performed by Caucasian and non-Caucasian populations. Random effects were used throughout. Effect summaries for additive models are pooled beta regression coefficients. Effect summaries for recessive and dominant models are mean differences. Heterogeneity is quantified using I^2 and mean differences (MD) are given with 95% confidence intervals (CI) in the units specified for each outcome. ALT, alanine aminotransferase; HDL, high-density lipoprotein; LDL, low-density lipoprotein.

Outcome	Genetic model	Sub-analysis	No. of studies	Heterogeneity		Effect summary	
				I^2	p_Q	OR [95% CI]	p_Z
NAFLD diagnosis (control vs NAFLD)	Additive	Overall	7	0	1.00	1.0017 [0.7790; 1.2879]	0.99
NAFLD diagnosis (control vs NAFLD)	Dominant	Overall	7	0	0.61	1.0090 [0.8567; 1.1882]	0.92
NAFLD diagnosis (control vs NAFLD)	Recessive	Overall	7	0	0.47	1.0085 [0.8292; 1.2265]	0.94
Severe steatosis (S1-2 vs S3)	Additive	Overall	3	0	0.81	1.0565 [0.6042; 1.8475]	0.86
Severe steatosis (S1-2 vs S3)	Dominant	Overall	3	76.4	0.014	0.9543 [0.4315; 2.1104]	0.92
Severe steatosis (S1-2 vs S3)	Recessive	Overall	3	0	0.56	1.3357 [0.8667; 2.0585]	0.19
NASH (NAFL vs NASH)	Additive	Overall	3	0	1.00	0.9492 [0.5197; 1.7334]	0.87
NASH (NAFL vs NASH)	Dominant	Overall	3	0	0.73	1.0263 [0.6876; 1.5317]	0.91

NASH (NAFL vs NASH)	Recessive	Overall	3	0	0.62	0.8226 [0.5178; 1.3068]	0.42
Any fibrosis (F0 vs F1-4)	Additive	Overall	3	0	0.97	0.8885 [0.4890; 1.6142]	0.71
Any fibrosis (F0 vs F1-4)	Dominant	Overall	3	0	0.80	1.0970 [0.7359; 1.6352]	0.66
Any fibrosis (F0 vs F1-4)	Recessive	Overall	3	0	0.60	0.6571 [0.4172; 1.0347]	0.94
Advanced fibrosis (F0-2 vs F3-4)	Additive	Overall	3	0	0.86	0.8315 [0.3363; 2.0560]	0.70
Advanced fibrosis (F0-2 vs F3-4)	Dominant	Overall	3	0	0.63	0.9710 [0.5966; 1.5804]	0.91
Advanced fibrosis (F0-2 vs F3-4)	Recessive	Overall	3	42	0.18	0.4568 [0.1552; 1.3444]	0.16
**Outcome**	**Genetic model**	**Sub-analysis**	**No. of studies**	**Heterogeneity**		**Effect summary**	
				**I^2**	**p_Q**	**Mean difference [95% CI]**	**p_Z**
Hepatic fat fraction (%)	Dominant	Overall	5	0.71	0.01	1.34 (-2.47, 5.15)	0.39

Hepatic fat fraction (%)	Dominant	Caucasian	3	0.80	0.01	1.45 (-8.24, 11.14)	0.59
Hepatic fat fraction (%)	Dominant	Non-Caucasian	2	0.72	0.06	1.29 (-24.01, 26.58)	0.63
Hepatic fat fraction (%)	Recessive	Overall	5	0.00	0.50	.58 (-1.8, 2.96)	0.53
Hepatic fat fraction (%)	Recessive	Caucasian	3	0.01	0.36	1.04 (-3.96, 6.04)	0.47
Hepatic fat fraction (%)	Recessive	Non-Caucasian	2	0.00	0.35	-.28 (-19.55, 19.)	0.89
ALT (IU/L)	Dominant	Overall	9	0.23	0.24	.29 (-1.49, 2.08)	0.72
ALT (IU/L)	Dominant	Non-Caucasian	3	0.00	0.75	-.97 (-3.17, 1.23)	0.20
ALT (IU/L)	Dominant	Caucasian	6	0.29	0.22	1.24 (-1.83, 4.3)	0.35
ALT (IU/L)	Recessive	Overall	9	0.05	0.39	-.88 (-2.52, .76)	0.25
ALT (IU/L)	Recessive	Non-Caucasian	3	0.00	0.65	-2.37 (-5.94, 1.21)	0.10
ALT (IU/L)	Recessive	Caucasian	6	0.09	0.36	-.28 (-2.61, 2.06)	0.77
Total cholesterol (mmol/L)	Dominant	Overall	8	0.00	0.52	.06 (-.005, .12)	0.07
Total cholesterol (mmol/L)	Dominant	Non-Caucasian	3	0.26	0.26	.02 (-.25, .28)	0.81
Total cholesterol (mmol/L)	Dominant	Caucasian	5	0.00	0.67	.08 (.01, .16)	0.04
Total cholesterol (mmol/L)	Recessive	Overall	8	0.00	0.50	-.02 (-.1, .05)	0.51
Total cholesterol (mmol/L)	Recessive	Non-Caucasian	3	0.00	0.55	.01 (-.25, .28)	0.85
Total cholesterol (mmol/L)	Recessive	Caucasian	5	0.19	0.30	-.03 (-.15, .1)	0.60
HDL (mmol/L)	Dominant	Overall	7	0.00	0.75	.01 (-.02, .03)	0.44

HDL (mmol/L)	Dominant	Non-Caucasian	2	0.47	0.17	.03 (-.42, .48)	0.53
HDL (mmol/L)	Dominant	Caucasian	5	0.00	0.96	.001 (-.02, .02)	0.91
HDL (mmol/L)	Recessive	Overall	7	0.04	0.40	.01 (-.03, .04)	0.75
HDL (mmol/L)	Recessive	Non-Caucasian	2	0.62	0.11	.02 (-.76, .8)	0.78
HDL (mmol/L)	Recessive	Caucasian	5	0.00	0.46	.0041 (-.04, .05)	0.80
Insulin (pmol/L)	Dominant	Overall	6	0.69	0.01	2.83 (-29.09, 34.75)	0.83
Insulin (pmol/L)	Dominant	Non-Caucasian	2	0.87	0.00	-24.6 (-587.96, 538.75)	0.68
Insulin (pmol/L)	Dominant	Caucasian	4	0.52	0.10	8. (-18.67, 34.68)	0.41
Insulin (pmol/L)	Recessive	Overall	6	0.31	0.20	-4.52 (-22.31, 13.28)	0.54
Insulin (pmol/L)	Recessive	Non-Caucasian	2	0.00	0.64	-37.96 (-138.32, 62.4)	0.13
Insulin (pmol/L)	Recessive	Caucasian	4	0.00	0.43	-1.55 (-14.24, 11.15)	0.72
LDL (mmol/L)	Dominant	Overall	6	0.19	0.29	.06 (-.03, .15)	0.14
LDL (mmol/L)	Dominant	Non-Caucasian	2	0.68	0.08	.01 (-1.49, 1.51)	0.96
LDL (mmol/L)	Dominant	Caucasian	4	0.00	0.45	.07 (-.02, .17)	0.10
LDL (mmol/L)	Recessive	Overall	6	0.00	0.55	-.00003 (-.08, .08)	1.00
LDL (mmol/L)	Recessive	Non-Caucasian	2	0.00	0.47	-.01 (-.75, .73)	0.93
LDL (mmol/L)	Recessive	Caucasian	4	0.13	0.33	.01 (-.12, .13)	0.90

Triglycerides (mmol/L)	Dominant	Overall	9	0.00	0.45	-.02 (-.05, .02)	0.24
Triglycerides (mmol/L)	Dominant	Non-Caucasian	3	0.00	0.43	-.05 (-.18, .07)	0.21
Triglycerides (mmol/L)	Dominant	Caucasian	6	0.00	0.46	-.01 (-.05, .03)	0.60
Triglycerides (mmol/L)	Recessive	Overall	9	0.00	0.66	-.02 (-.06, .01)	0.19
Triglycerides (mmol/L)	Recessive	Non-Caucasian	3	0.00	0.92	-.08 (-.14, -.02)	0.03
Triglycerides (mmol/L)	Recessive	Caucasian	6	0.00	0.59	-.01 (-.06, .03)	0.56
**Outcome**	**Genetic model**	**Sub-analysis**	**No. of studies**	**Heterogeneity**		**Effect summary**	
				**I^2**	**p_Q**	**Beta regression coefficient [95% CI]**	**p_Z**
Hepatic fat fraction (inv-norm SD)	Linear	Overall	5	0.96	0.00	.13 (-.06, .32)	0.17
Hepatic fat fraction (inv-norm SD)	Linear	Non-Caucasian	2	0.81	0.02	-.01 (-.09, .07)	0.78
Hepatic fat fraction (inv-norm SD)	Linear	Caucasian	3	0.97	0.00	.24 (-.31, .79)	0.39
ALT (IU/L)	Additive	Overall	9	0.34	0.14	.1 (-1.16, 1.36)	0.88
ALT (IU/L)	Additive	Non-Caucasian	3	0.00	0.76	-.87 (-2.3, .56)	0.23
ALT (IU/L)	Additive	Caucasian	6	0.44	0.11	.83 (-1.14, 2.8)	0.41
HDL (mmol/L)	Additive	Overall	7	0.00	0.52	.01 (-.01, .02)	0.51
HDL (mmol/L)	Additive	Non-Caucasian	2	0.70	0.07	.02 (-.04, .09)	0.45
HDL (mmol/L)	Additive	Caucasian	5	0.00	0.94	.001 (-.02, .02)	0.91

Insulin (pmol/L)	Additive	Overall	6	0.67	0.01	-.28 (-12.31, 11.75)	0.96
Insulin (pmol/L)	Additive	Non-Caucasian	2	0.80	0.02	-20.85 (-70.04, 28.35)	0.41
Insulin (pmol/L)	Additive	Caucasian	4	0.52	0.10	3.87 (-5.86, 13.59)	0.44
LDL (mmol/L)	Additive	Overall	6	0.00	0.46	.03 (-.01, .06)	0.17
LDL (mmol/L)	Additive	Non-Caucasian	2	0.60	0.11	.0005 (-.15, .15)	0.99
LDL (mmol/L)	Additive	Caucasian	4	0.00	0.57	.03 (-.01, .07)	0.16
Total cholesterol (mmol/L)	Additive	Overall	8	0.00	0.47	.02 (-.02, .06)	0.32
Total cholesterol (mmol/L)	Additive	Non-Caucasian	3	0.26	0.26	.01 (-.08, .1)	0.83
Total cholesterol (mmol/L)	Additive	Caucasian	5	0.00	0.43	.02 (-.02, .07)	0.32
Triglycerides (mmol/L)	Additive	Overall	9	0.00	0.76	-.01 (-.03, .01)	0.15
Triglycerides (mmol/L)	Additive	Non-Caucasian	3	0.00	0.69	-.05 (-.1, -.0003)	0.05
Triglycerides (mmol/L)	Additive	Caucasian	6	0.00	0.85	-.01 (-.03, .01)	0.49

Table S10.

Summary of all results from meta-analysis of the role of rs641738C>T in paediatric NAFLD. Each dichotomous outcome was tested using dominant (CC vs. CT+TT), recessive, (CC+CT vs. TT), and additive (CC vs. CT vs. TT) models of inheritance and, where there were sufficient studies, with sub-analysis by studies with Caucasian and non-Caucasian populations. Effect summary is given as odds ratios (OR) with 95% confidence intervals (CI) for dichotomous outcomes. For continuous traits: effect summaries

for additive models are pooled beta regression coefficients; and for recessive and dominant models are mean differences. For analysis of hepatic fat fraction, effect summary using an additive model is beta (regression coefficient) of inverse-normalised (inv-norm) hepatic fat, in terms of standard deviations (SD). The p-value for the effect summary (p_z) should be assessed against a critical p-value of 0.017, due to testing three genetic models. ALT, alanine aminotransferase; HDL, high-density lipoprotein; LDL, low-density lipoprotein.

**Outcome**	**Variable**	**k**	**Beta**	**SE**	**p-value**	**R^2**
NAFLD diagnosis (control vs NAFLD)	Female	17	-0.05	0.37	0.89	0
NAFLD diagnosis (control vs NAFLD)	Age	17	0.01	0.01	0.12	19.8
NAFLD diagnosis (control vs NAFLD)	BMI	17	0.00	0.01	0.83	0
NAFLD diagnosis (control vs NAFLD)	T2DM	17	-0.02	0.30	0.96	0
NAFLD diagnosis (control vs NAFLD)	*PNPLA3*	14	-0.47	0.42	0.26	0
Severe steatosis (S1-2 vs S3)	Female	8	0.47	1.01	0.64	0
Severe steatosis (S1-2 vs S3)	Age	8	0.04	0.04	0.26	30.8
Severe steatosis (S1-2 vs S3)	BMI	8	0.01	0.03	0.65	0
Severe steatosis (S1-2 vs S3)	T2DM	8	-2.61	1.49	0.080	64.8
Severe steatosis (S1-2 vs S3)	*PNPLA3*	7	-0.29	2.35	0.90	0
NASH (NAFL vs NASH)	Female	9	0.36	0.57	0.52	0
NASH (NAFL vs NASH)	Age	9	0.02	0.02	0.33	0.8
NASH (NAFL vs NASH)	BMI	9	-0.01	0.02	0.72	0
NASH (NAFL vs NASH)	T2DM	9	-1.79	0.65	0.006	100
NASH (NAFL vs NASH)	*PNPLA3*	8	-1.07	1.04	0.30	4.7
Any fibrosis (F0 vs F1-4)	Female	9	0.17	0.67	0.80	0
Any fibrosis (F0 vs F1-4)	Age	9	0.05	0.02	0.014	67.9

Any fibrosis (F0 vs F1-4)	BMI	9	-0.02	0.02	0.15	2.0
Any fibrosis (F0 vs F1-4)	T2DM	9	-1.53	0.83	0.064	46.5
Any fibrosis (F0 vs F1-4)	*PNPLA3*	8	0.22	1.36	0.87	0
Advanced fibrosis (F0-2 vs F3-4)	Female	8	0.17	0.59	0.77	0
Advanced fibrosis (F0-2 vs F3-4)	Age	8	0.00	0.02	0.81	0
Advanced fibrosis (F0-2 vs F3-4)	BMI	8	0.01	0.02	0.52	0
Advanced fibrosis (F0-2 vs F3-4)	T2DM	8	-0.63	0.91	0.49	0
Advanced fibrosis (F0-2 vs F3-4)	*PNPLA3*	7	-1.39	1.21	0.25	0
HCC (NAFLD-HCC vs NAFLD no-HCC)	Female	4	0.28	1.54	0.86	0
HCC (NAFLD-HCC vs NAFLD no-HCC)	Age	4	-0.03	0.07	0.68	0
HCC (NAFLD-HCC vs NAFLD no-HCC)	T2DM	3	-0.81	1.39	0.56	0
HCC (NAFLD-HCC vs NAFLD no-HCC)	Cirrhosis	4	-0.34	0.71	0.63	0

Table S11.

Summary of all meta-regression analyses. All analyses were performed from random effects meta-analyses using a recessive model of inheritance. Univariable meta-regression was performed where >2 studies reported the variable of interest. '*PNPLA3*' refers to the proportion of participants carrying the rs738409C>G allele. BMI, body mass index; SE, standard error; T2DM, type 2 diabetes mellitus.

Supplementary references

[1] MacArthur J, Bowler E, Cerezo M, Gil L, Hall P, Hastings E, et al. The new NHGRI-EBI Catalog of published genome-wide association studies (GWAS Catalog). Nucleic Acids Res 2017;45:D896–901.

[2] Kamat MA, Blackshaw JA, Young R, Surendran P, Burgess S, Danesh J, et al. PhenoScanner V2: an expanded tool for searching human genotype-phenotype associations. Bioinformatics 2019;35:4851–3.

[3] Cardiovascular Disease Knowledge Portal n.d. http://www.broadcvdi.org/ (accessed July 28, 2020).

[4] Type 2 diabetes knowledge portal n.d. http://www.type2diabetesgenetics.org/ (accessed July 28, 2020).

[5] Boyd A, Golding J, Macleod J, Lawlor DA, Fraser A, Henderson J, et al. Cohort Profile: the “children of the 90s”--the index offspring of the Avon Longitudinal Study of Parents and Children. Int J Epidemiol 2013;42:111–27.

[6] Northstone K, Lewcock M, Groom A, Boyd A, Macleod J, Timpson N, et al. The Avon Longitudinal Study of Parents and Children (ALSPAC): an update on the enrolled sample of index children in 2019. Wellcome Open Res 2019;4:51.

[7] Harris PA, Taylor R, Thielke R, Payne J, Gonzalez N, Conde JG. Research electronic data capture (REDCap)--a metadata-driven methodology and workflow process for providing translational research informatics support. J Biomed Inform 2009;42:377–81.

[8] Emdin CA, Haas ME, Khera AV, Aragam K, Chaffin M, Klarin D, et al. A missense variant in Mitochondrial Amidoxime Reducing Component 1 gene and protection against liver disease. PLoS Genet 2020;16:e1008629.

[9] Chen VL, Chen Y, Du X, Handelman SK, Speliotes EK. Genetic variants that associate with cirrhosis have pleiotropic effects on human traits. Liver Int 2020;40:405–15.

[10] Kichaev G, Bhatia G, Loh P-R, Gazal S, Burch K, Freund MK, et al. Leveraging Polygenic Functional Enrichment to Improve GWAS Power. Am J Hum Genet 2019;104:65–75.

[11] Parisinos CA, Wilman HR, Thomas EL, Kelly M, Nicholls RC, McGonigle J, et al. Genome-wide and Mendelian randomisation studies of liver MRI yield insights into the pathogenesis of steatohepatitis. J Hepatol 2020;73:241–51.

[12] McInnes G, Tanigawa Y, DeBoever C, Lavertu A, Olivieri JE, Aguirre M, et al. Global Biobank Engine: enabling genotype-phenotype browsing for biobank summary statistics. Bioinformatics 2019;35:2495–7.

[13] Benjamin EJ, Dupuis J, Larson MG, Lunetta KL, Booth SL, Govindaraju DR, et al. Genome-wide association with select biomarker traits in the Framingham Heart Study. BMC Med Genet 2007;8 Suppl 1:S11.

[14] Basyte-Bacevice V, Skieceviciene J, Valantiene I, Sumskiene J, Petrenkiene V, Kondrackiene J, et al. TM6SF2 and MBOAT7 Gene Variants in Liver Fibrosis and Cirrhosis. Int J Mol Sci 2019;20. https://doi.org/10.3390/ijms20061277.

[15] Chung GE, Lee Y, Yim JY, Choe EK, Kwak M-S, Yang JI, et al. Genetic Polymorphisms of PNPLA3 and SAMM50 Are Associated with Nonalcoholic Fatty Liver Disease in a Korean Population. Gut Liver 2017;12:316–23.

[16] Clifford RJ, Zhang J, Meerzaman DM, Lyu M-S, Hu Y, Cultraro CM, et al. Genetic variations at loci involved in the immune response are risk factors for hepatocellular carcinoma. Hepatology 2010;52:2034–43.

[17] Kim YJ, Go MJ, Hu C, Hong CB, Kim YK, Lee JY, et al. Large-scale genome-wide association studies in East Asians identify new genetic loci influencing metabolic traits. Nat Genet 2011;43:990–5.

[18] Kitamoto T, Kitamoto A, Yoneda M, Hyogo H, Ochi H, Nakamura T, et al. Genome-wide scan revealed that polymorphisms in the PNPLA3, SAMM50, and PARVB genes are associated with development and progression of nonalcoholic fatty liver

disease in Japan. Hum Genet 2013;132:783–92.

[19] Park T-J, Hwang J-Y, Go MJ, Lee H-J, Jang HB, Choi Y, et al. Genome-wide association study of liver enzymes in korean children. Genomics Inform 2013;11:149–54.

[20] Raksayot M, Chuaypen N, Khlaiphuengsin A, Pinjaroen N, Treeprasertsuk S, Poovorawan Y, et al. Independent and additive effects of PNPLA3 and TM6SF2 polymorphisms on the development of non-B, non-C hepatocellular carcinoma. J Gastroenterol 2019;54:427–36.

[21] Zemunik T, Boban M, Lauc G, Janković S, Rotim K, Vatavuk Z, et al. Genome-wide association study of biochemical traits in Korcula Island, Croatia. Croat Med J 2009;50:23–33.

[22] Chalasani N, Guo X, Loomba R, Goodarzi MO, Haritunians T, Kwon S, et al. Genome-wide association study identifies variants associated with histologic features of nonalcoholic fatty liver disease. Gastroenterology 2010;139:1567–76.e6.

[23] Danford CJ, Connelly MA, Shalaurova I, Kim M, Herman MA, Nasser I, et al. A Pathophysiologic Approach Combining Genetics and Insulin Resistance to Predict the Severity of Nonalcoholic Fatty Liver Disease. Hepatol Commun 2018;2:1467–78.

[24] Taylor RS, Taylor RJ, Bayliss S, Hagström H, Nasr P, Schattenberg JM, et al. Association Between Fibrosis Stage and Outcomes of Patients With Nonalcoholic Fatty Liver Disease: A Systematic Review and Meta-Analysis. Gastroenterology 2020. https://doi.org/10.1053/j.gastro.2020.01.043.

[25] Cochrane Collaboration. Tool to assess risk of bias in cohort studies 2017.

[26] Duval S, Tweedie R. Trim and fill: A simple funnel-plot-based method of testing and adjusting for publication bias in meta-

analysis. Biometrics 2000;56:455–63.

[27] R Core Team. A language and environment for statistical computing. Vienna, Austria: R Foundation for Statistical Computing 2019.

[28] Harrer M, Cuijpers P, Furukawa TA, Ebert DD. Doing Meta-Analysis in R: A Hands-on Guide. PROTECT Lab Erlangen 2019.

[29] Speliotes EK, Yerges-armstrong LM, Wu J, Hernaez R, Lauren J, Palmer CD, et al. Genome-Wide Association Analysis Identifies Variants Associated with Nonalcoholic Fatty Liver Disease That Have Distinct Effects on Metabolic Traits. PLoS Genet 2011;7:e1001324.

[30] Barata L, Feitosa MF, Bielak LF, Halligan B, Baldridge AS, Guo X, et al. Insulin Resistance Exacerbates Genetic Predisposition to Nonalcoholic Fatty Liver Disease in Individuals Without Diabetes. Hepatol Commun 2019;3:894–907.

[31] Di Costanzo A, Pacifico L, Chiesa C, Perla FM, Ceci F, Angeloni A, et al. Genetic and metabolic predictors of hepatic fat content in a cohort of Italian children with obesity. Pediatr Res 2019;85:671–7.

[32] Di Costanzo A, Pacifico L, D'Erasmo L, Polito L, Martino MD, Perla FM, et al. Nonalcoholic Fatty Liver Disease (NAFLD), But not Its Susceptibility Gene Variants, Influences the Decrease of Kidney Function in Overweight/Obese Children. Int J Mol Sci 2019;20. https://doi.org/10.3390/ijms20184444.

[33] Di Sessa A, Umano GR, Cirillo G, Del Prete A, Iacomino R, Marzuillo P, et al. The Membrane-bound O-Acyltransferase7 rs641738 Variant in Pediatric Nonalcoholic Fatty Liver Disease. J Pediatr Gastroenterol Nutr 2018;67:69–74.

[34] Di Sessa A, Umano GR, Cirillo G, Marzuillo P, Arienzo MR, Pedullà M, et al. The rs72613567: TA Variant in the Hydroxysteroid 17-beta Dehydrogenase 13 Gene Reduces Liver Damage in Obese Children. J Pediatr Gastroenterol Nutr 2020;70:371–4.

[35] Gawrieh S, Guo X, Tan J, Lauzon M, Taylor KD, Loomba R, et al. A Pilot Genome-Wide Analysis Study Identifies Loci Associated With Response to Obeticholic Acid in Patients With NASH. Hepatol Commun 2019;3:1571–84.

[36] Kanai M, Akiyama M, Takahashi A, Matoba N, Momozawa Y, Ikeda M, et al. Genetic analysis of quantitative traits in the Japanese population links cell types to complex human diseases. Nat Genet 2018;50:390–400.

[37] Kamatani Y, Matsuda K, Okada Y, Kubo M, Hosono N, Daigo Y, et al. Genome-wide association study of hematological and biochemical traits in a Japanese population. Nat Genet 2010;42:210–5.

[38] Kawaguchi T, Shima T, Mizuno M, Mitsumoto Y, Umemura A, Kanbara Y, et al. Risk estimation model for nonalcoholic fatty liver disease in the Japanese using multiple genetic markers. PLoS One 2018;13:1–16.

[39] Kawaguchi T, Sumida Y, Umemura A, Matsuo K, Takahashi M, Takamura T, et al. Genetic polymorphisms of the human PNPLA3 gene are strongly associated with severity of non-alcoholic fatty liver disease in Japanese. PLoS One 2012;7:1–10.

[40] Liu Y, Basty N, Whitcher B, Bell J, van Bruggen N, Thomas EL, et al. Systematic quantification of health parameters from UK Biobank abdominal MRI using deep learning. bioRxiv 2020:2020.07.14.187070. https://doi.org/10.1101/2020.07.14.187070.

[41] Koo BK, An JN, Joo SK, Kim D, Lee S, Bae JM, et al. Association Between a Polymorphism in MBOAT7 and Chronic Kidney Disease in Patients With Biopsy-Confirmed Nonalcoholic Fatty Liver Disease. Clin Gastroenterol Hepatol 2019. https://doi.org/10.1016/j.cgh.2019.09.017.

[42] Koo BK, Joo SK, Kim D, Bae JM, Park JH, Kim JH, et al. Additive effects of PNPLA3 and TM6SF2 on the histological severity of non-alcoholic fatty liver disease. J Gastroenterol Hepatol 2018;33:1277–85.

[43] Krawczyk M, Bantel H, Rau M, Schattenberg JM, Grünhage F, Pathil A, et al. Could inherited predisposition drive non-obese

fatty liver disease? Results from German tertiary referral centers. J Hum Genet 2018;63:621–6.

[44] Krawczyk M, Rau M, Schattenberg JM, Bantel H, Pathil A, Demir M, et al. Combined effects of the *PNPLA3* rs738409, *TM6SF2* rs58542926, and *MBOAT7* rs641738 variants on NAFLD severity: a multicenter biopsy-based study. J Lipid Res 2017;58:247–55.

[45] Schulte M, Arslanow A, Weber SN, Lammert F, Krawczyk M. HSD17B13 hepatoprotective variant limits liver disease severity in homozygous carriers of the PNPLA3 p. 148MM risk genotype. Viszeralmedizin 2019, vol. 57, Georg Thieme Verlag KG; 2019, p. KV 244.

[46] Krawczyk M, Rau M, Schattenberg JM, Bantel H, Pathil A, Demir M, et al. Presence of the MBOAT7 rs641738 variant might enhance liver fibrosis in patients with fatty liver: analysis of the German NAFLD CSG cohort. 33. Jahrestagung der Deutschen Arbeitsgemeinschaft zum Studium der Leber, vol. 54, Georg Thieme Verlag KG; 2016, p. A1.11.

[47] Luukkonen PK, Tukiainen T, Juuti A, Sammalkorpi H, Haridas PAN, Niemelä O, et al. Hydroxysteroid 17-β dehydrogenase 13 variant increases phospholipids and protects against fibrosis in nonalcoholic fatty liver disease. JCI Insight 2020;5. https://doi.org/10.1172/jci.insight.132158.

[48] Luukkonen PK, Zhou Y, Hyötyläinen T, Leivonen M, Arola J, Orho-Melander M, et al. The MBOAT7 variant rs641738 alters hepatic phosphatidylinositols and increases severity of non-alcoholic fatty liver disease in humans. J Hepatol 2016;65:1263–5.

[49] Luukkonen PK, Sädevirta S, Zhou Y, Kayser B, Ali A, Ahonen L, et al. Saturated fat is more metabolically harmful for the human liver than unsaturated fat or simple sugars. Diabetes Care 2018;41:1732–9.

[50] Dongiovanni P, Stender S, Pietrelli A, Mancina RM, Cespiati A, Petta S, et al. Causal relationship of hepatic fat with liver

damage and insulin resistance in nonalcoholic fatty liver. J Intern Med 2018;283:356–70.

[51] Mancina RM, Dongiovanni P, Petta S, Pingitore P, Meroni M, Rametta R, et al. The MBOAT7-TMC4 Variant rs641738 Increases Risk of Nonalcoholic Fatty Liver Disease in Individuals of European Descent. Gastroenterology 2016;150:1219–30e6.

[52] Dongiovanni P, Meroni M, Mancina RM, Baselli G, Rametta R, Pelusi S, et al. Protein phosphatase 1 regulatory subunit 3B gene variation protects against hepatic fat accumulation and fibrosis in individuals at high risk of nonalcoholic fatty liver disease. Hepatology Communications 2018;2:666–75.

[53] Meroni M, Dongiovanni P, Longo M, Carli F, Baselli G, Rametta R, et al. Mboat7 down-regulation by hyper-insulinemia induces fat accumulation in hepatocytes. EBioMedicine 2020;52:102658.

[54] M Kubiliun, JC Cohen, HH Hobbs, J Kozlitina. Effects of high-risk genetic polymorphisms on alcoholic and nonalcoholic fatty liver disease in a multi-ethnic population. Hepatology 2019;70:152A.

[55] Krawczyk M, Jiménez-Agüero R, Alustiza JM, Emparanza JI, Perugorria MJ, Bujanda L, et al. PNPLA3 p.I148M variant is associated with greater reduction of liver fat content after bariatric surgery. Surg Obes Relat Dis 2016;12:1838–46.

[56] A Santos-Laso, L Velaz, C Alonso, E Eizaguirre, I Martinez-Arranz, MJ Pareka, I Riano, J Andersen, E Arretxe, I Minchole, P Oritz, MJ Perugorria, A Landa, M Krawczyk, F Lammert, RE Castro, P Aspichueta, M Romero-Gomez, L Bujanda, PM Rodrigues, J Banales. Obese Patients Carrying Nafld-Associated Genetic Variants Present Specific Serum and Liver Lipidomic Profiles: Identification of a Lipidomic Signature in Serum to Estimate the Liver Fat Content. Hepatology 2019;70:168A.

[57] Chambers JC, Zhang W, Sehmi J, Li X, Wass MN, Van Der Harst P, et al. Genome-wide association study identifies loci

influencing concentrations of liver enzymes in plasma. Nat Genet 2011;43:1131–8.

[58] Yuan X, Waterworth D, Perry JRB, Lim N, Song K, Chambers JC, et al. Population-Based Genome-wide Association Studies Reveal Six Loci In influencing Plasma Levels of Liver Enzymes. J Hum Genet 2008:520–8.

[59] Donati B, Dongiovanni P, Romeo S, Meroni M, McCain M, Miele L, et al. MBOAT7 rs641738 variant and hepatocellular carcinoma in non-cirrhotic individuals. Sci Rep 2017;7:4492.

[60] Pelusi S, Baselli G, Pietrelli A, Dongiovanni P, Donati B, McCain MV, et al. Rare Pathogenic Variants Predispose to Hepatocellular Carcinoma in Nonalcoholic Fatty Liver Disease. Sci Rep 2019;9:3682.

[61] Strnad P, Buch S, Hamesch K, Fischer J, Rosendahl J, Schmelz R, et al. Heterozygous carriage of the alpha1-antitrypsin Pi*Z variant increases the risk to develop liver cirrhosis. Gut 2019;68:1099–107.

[62] Thangapandi VR, Knittelfelder O, Brosch M, Patsenker E, Vvedenskaya O, Buch S, et al. Loss of hepatic Mboat7 leads to liver fibrosis. Gut 2020. https://doi.org/10.1136/gutjnl-2020-320853.

[63] Abeysekera KWM, Fernandes GS, Hammerton G, Portal AJ, Gordon FH, Heron J, et al. Prevalence of steatosis and fibrosis in young adults in the UK: a population-based study. Lancet Gastroenterol Hepatol 2020;5:295–305.

[64] Adams LA, White SW, Marsh JA, Lye SJ, Connor KL, Maganga R, et al. Association between liver-specific gene polymorphisms and their expression levels with nonalcoholic fatty liver disease. Hepatology 2013;57:590–600.

[65] Anstee QM, Darlay R, Cockell S, Meroni M, Govaere O, Tiniakos D, et al. Genome-wide association study of non-alcoholic fatty liver and steatohepatitis in a histologically-characterised cohort. J Hepatol 2020. https://doi.org/10.1016/j.jhep.2020.04.003.

[66] Caussy C, Bhargava M, Villesen IF, Gudmann NS, Leeming DJ, Karsdal MA, et al. Collagen Formation Assessed by N-Terminal Propeptide of Type 3 Procollagen Is a Heritable Trait and Is Associated With Liver Fibrosis Assessed by Magnetic Resonance Elastography. Hepatology 2019;70:127–41.

[67] Chatterjee A, Das K, Singh P, Mondal D, Ghosh R, Chowdhury A, et al. Exome-wide association study with hepatic fat content in nonalcoholic fatty liver disease reveals significant association with 5 novel QTLs. Hepatol Int 2018;12:181.

[68] Di Costanzo A, Belardinilli F, Bailetti D, Sponziello M, D'Erasmo L, Polimeni L, et al. Evaluation of Polygenic Determinants of Non-Alcoholic Fatty Liver Disease (NAFLD) By a Candidate Genes Resequencing Strategy. Sci Rep 2018;8:1–10.

[69] DiStefano JK, Kingsley C, Craig Wood G, Chu X, Argyropoulos G, Still CD, et al. Genome-wide analysis of hepatic lipid content in extreme obesity. Acta Diabetol 2014;52:373–82.

[70] Gurdasani D, Carstensen T, Fatumo S, Chen G, Franklin CS, Prado-Martinez J, et al. Uganda Genome Resource Enables Insights into Population History and Genomic Discovery in Africa. Cell 2019;179:984–1002.e36.

[71] Guzman CB, Duvvuru S, Akkari A, Bhatnagar P, Battioui C, Foster W, et al. Coding variants in PNPLA3 and TM6SF2 are risk factors for hepatic steatosis and elevated serum alanine aminotransferases caused by a glucagon receptor antagonist. Hepatology Communications 2018;2:561–70.

[72] Hudert CA, Selinski S, Rudolph B, Bläker H, Loddenkemper C, Thielhorn R, et al. Genetic determinants of steatosis and fibrosis progression in paediatric non-alcoholic fatty liver disease. Liver Int 2019;39:540–56.

[73] Käräjämäki AJ, Hukkanen J, Kauma H, Kesäniemi YA, Ukkola O. Metabolic syndrome but not genetic polymorphisms known to induce NAFLD predicts increased total mortality in subjects with NAFLD (OPERA study). Scand J Clin Lab Invest 2019:1–8.

[74] Koo BK, Joo SK, Kim D, Lee S, Bae JM, Park JH, et al. Development and Validation of a Scoring System, Based on Genetic and Clinical Factors, to Determine Risk of Steatohepatitis in Asian Patients with Nonalcoholic Fatty Liver Disease. Clin Gastroenterol Hepatol 2020. https://doi.org/10.1016/j.cgh.2020.02.011.

[75] Lin YC, Chang PF, Chang MH, Ni YH. Genetic determinants of hepatic steatosis and serum cytokeratin-18 fragment levels in Taiwanese children. Liver Int 2018;38:1300–7.

[76] Mann JP, Vreugdenhil A, Socha P, Jańczyk W, Baumann U, Rajwal S, et al. European paediatric non-alcoholic fatty liver disease registry (EU-PNAFLD): Design and rationale. Contemp Clin Trials 2018;75:67–71.

[77] Mann JP, Pietzner M, Wittemans LB, De Lucia Rolfe E, Nicola D, Imamura F, et al. Insights into genetic variants associated with NASH-fibrosis from metabolite profiling. Hum Mol Genet 2020:doi: 10.1093/hmg/ddaa162.

[78] Middelberg RPS, Ferreira MAR, Henders AK, Heath AC, Madden PAF, Montgomery GW, et al. Genetic variants in LPL, OASL and TOMM40/APOE-C1-C2-C4 genes are associated with multiple cardiovascular-related traits. BMC Med Genet 2011;12:123.

[79] Moon S, Kim YJ, Han S, Hwang MY, Shin DM, Park MY, et al. The Korea Biobank Array: Design and Identification of Coding Variants Associated with Blood Biochemical Traits. Sci Rep 2019;9:1382.

[80] Prins BP, Kuchenbaecker KB, Bao Y, Smart M, Zabaneh D, Fatemifar G, et al. Genome-wide analysis of health-related biomarkers in the UK Household Longitudinal Study reveals novel associations. Sci Rep 2017;7:11008.

[81] Reichert MC, Ripoll C, Casper M, Greinert R, Vandieken E, Grünhage F, et al. Common NOD2 Risk Variants as Major Susceptibility Factors for Bacterial Infections in Compensated Cirrhosis. Clin Transl Gastroenterol 2019;10:e00002.

[82] Seidelin A-S, Nordestgaard BG, Tybjærg-Hansen A, Stender S. Genetic Variation at PPP1R3B Increases Hepatic CT Attenuation and Interacts With Prandial Status on Plasma Glucose. J Clin Endocrinol Metab 2020;105. https://doi.org/10.1210/clinem/dgaa151.

[83] Sookoian S, Flichman D, Garaycoechea ME, Gazzi C, Martino JS, Castaño GO, et al. Lack of evidence supporting a role of TMC4-rs641738 missense variant - MBOAT7- intergenic downstream variant - In the Susceptibility to Nonalcoholic Fatty Liver Disease. Sci Rep 2018;8:5097.

[84] Tabassum R, Ramo JT, Ripatti P, Koskela JT, Kurki M, Karjalainen J, et al. Genetic architecture of human plasma lipidome and its link to cardiovascular disease. Nat Commun 2019;10:4329.

[85] Umano GR, Caprio S, Di Sessa A, Chalasani N, Dykas DJ, Pierpont B, et al. The rs626283 variant in the MBOAT7 gene is associated with insulin resistance and fatty liver in Caucasian obese youth. Am J Gastroenterol 2018;113:376–83.

[86] Verma SS, Lucas AM, Lavage DR, Leader JB, Metpally R, Krishnamurthy S, et al. IDENTIFYING GENETIC ASSOCIATIONS WITH VARIABILITY IN METABOLIC HEALTH AND BLOOD COUNT LABORATORY VALUES: DIVING INTO THE QUANTITATIVE TRAITS BY LEVERAGING LONGITUDINAL DATA FROM AN EHR. Pac Symp Biocomput 2017;22:533–44.

[87] Viitasalo A, Eloranta A-M, Atalay M, Romeo S, Pihlajamaki J, Lakka TA. Association of MBOAT7 gene variant with plasma ALT levels in children: the PANIC study. Pediatr Res 2016;80:651–5.

[88] Wattacheril J, Lavine JE, Chalasani NP, Guo X, Kwon S, Schwimmer J, et al. Genome-Wide Associations Related to Hepatic Histology in Nonalcoholic Fatty Liver Disease in Hispanic Boys. J Pediatr 2017;190:100–7.e2.

[89] Willer CJ, Schmidt EM, Sengupta S, Peloso GM, Gustafsson S, Kanoni S, et al. Discovery and refinement of loci associated

with lipid levels. Nat Genet 2013;45:1274–83.

[90] Young KA, Palmer ND, Fingerlin TE, Langefeld CD, Norris JM, Wang N, et al. Genome-Wide Association Study Identifies Loci for Liver Enzyme Concentrations in Mexican Americans: The GUARDIAN Consortium. Obesity 2019;27:1331–7.

[91] Zusi C, Mantovani A, Olivieri F, Morandi A, Corradi M, Miraglia Del Giudice E, et al. Contribution of a genetic risk score to clinical prediction of hepatic steatosis in obese children and adolescents. Dig Liver Dis 2019;51:1586–92.

[92] Xia Y, Huang CX, Li GY, Chen KH, Han L, Tang L, et al. Meta-analysis of the association between MBOAT7 rs641738, TM6SF2 rs58542926 and nonalcoholic fatty liver disease susceptibility. Clin Res Hepatol Gastroenterol 2019:1–9.

[93] Ismaiel A, Dumitrascu DL. Genetic predisposition in metabolic-dysfunction-associated fatty liver disease and cardiovascular outcomes-Systematic review. Eur J Clin Invest 2020:e13331.

Code used in analyses

```
## R 3.6.1

library(readxl)
library(meta)
library(metafor)
library(dmetar)
library(forestplot)

***for histology

setwd("~/InputData")

**NAFLD Diagnosis in adults

NAFLD_Dx_adult <- read_excel("NAFLD_Dx_adult.xlsx")
```

```
NAFLD_Dx_adult_add <- metagen(add_logOR, add_seOR, studlab = Author, method.tau = "DL", sm = "OR", data =
NAFLD_Dx_adult)
NAFLD_Dx_adult_add_ethnic <- update(NAFLD_Dx_adult_add, byvar = Ethnicity, bylab = "Ethnicity")
NAFLD_Dx_adult_rec <- metagen(rec_logOR, rec_seOR, studlab = Author, method.tau = "DL", sm = "OR", data =
NAFLD_Dx_adult)
NAFLD_Dx_adult_rec_ethnic <- update(NAFLD_Dx_adult_rec, byvar = Ethnicity, bylab = "Ethnicity")
NAFLD_Dx_adult_rec_mod <- update(NAFLD_Dx_adult_rec, byvar = Modality, bylab = "Modality")
NAFLD_Dx_adult_dom <- metagen(dom_logOR, dom_seOR, studlab = Author, method.tau = "DL", sm = "OR", data =
NAFLD_Dx_adult)
NAFLD_Dx_adult_dom_ethnic <- update(NAFLD_Dx_adult_dom, byvar = Ethnicity, bylab = "Ethnicity")


** recessive - overall
NAFLD_Dx_adult_rec_tab <- data.frame(NAFLD_Dx_adult_rec_ethnic[["TE.random"]])
row.names(NAFLD_Dx_adult_rec_tab) <- "Overall"
NAFLD_Dx_adult_rec_tab$OR <- NAFLD_Dx_adult_rec_tab$NAFLD_Dx_adult_rec_ethnic...TE.random...
NAFLD_Dx_adult_rec_tab$OR <- exp(NAFLD_Dx_adult_rec_tab$OR)
NAFLD_Dx_adult_rec_tab <- NAFLD_Dx_adult_rec_tab[-c(1)]
NAFLD_Dx_adult_rec_tab$lower <- NAFLD_Dx_adult_rec_ethnic[["lower.random"]]
```

```
NAFLD_Dx_adult_rec_tab$lower <- exp(NAFLD_Dx_adult_rec_tab$lower)

NAFLD_Dx_adult_rec_tab$upper <- NAFLD_Dx_adult_rec_ethnic[["upper.random"]]

NAFLD_Dx_adult_rec_tab$upper <- exp(NAFLD_Dx_adult_rec_tab$upper)

NAFLD_Dx_adult_rec_tab$k <- NAFLD_Dx_adult_rec_ethnic[["k"]]

NAFLD_Dx_adult_rec_tab$p_z <- NAFLD_Dx_adult_rec_ethnic[["pval.random"]]

NAFLD_Dx_adult_rec_tab$I2 <- NAFLD_Dx_adult_rec_ethnic[["I2"]]

NAFLD_Dx_adult_rec_tab$p_q <- NAFLD_Dx_adult_rec_ethnic[["pval.Q"]]

NAFLD_Dx_adult_rec_tab$group <- "Overall"

NAFLD_Dx_adult_rec_tab$model <- "Recessive"

NAFLD_Dx_adult_rec_tab$outcome <- "NAFLD_Dx"


** recessive - subgroups

NAFLD_Dx_adult_rec_tab2 <- data.frame(NAFLD_Dx_adult_rec_ethnic[["TE.random.w"]])

row.names(NAFLD_Dx_adult_rec_tab2) <- NAFLD_Dx_adult_rec_ethnic[["bylevs"]]

NAFLD_Dx_adult_rec_tab2$OR <- NAFLD_Dx_adult_rec_tab2$NAFLD_Dx_adult_rec_ethnic...TE.random.w...

NAFLD_Dx_adult_rec_tab2$OR <- exp(NAFLD_Dx_adult_rec_tab2$OR)

NAFLD_Dx_adult_rec_tab2 <- NAFLD_Dx_adult_rec_tab2[-c(1)]

NAFLD_Dx_adult_rec_tab2$lower <- NAFLD_Dx_adult_rec_ethnic[["lower.random.w"]]
```

```
NAFLD_Dx_adult_rec_tab2$lower <- exp(NAFLD_Dx_adult_rec_tab2$lower)

NAFLD_Dx_adult_rec_tab2$upper <- NAFLD_Dx_adult_rec_ethnic[["upper.random.w"]]

NAFLD_Dx_adult_rec_tab2$upper <- exp(NAFLD_Dx_adult_rec_tab2$upper)

NAFLD_Dx_adult_rec_tab2$k <- NAFLD_Dx_adult_rec_ethnic[["k.w"]]

NAFLD_Dx_adult_rec_tab2$p_z <- NAFLD_Dx_adult_rec_ethnic[["pval.random.w"]]

NAFLD_Dx_adult_rec_tab2$I2 <- NAFLD_Dx_adult_rec_ethnic[["I2.w"]]

NAFLD_Dx_adult_rec_tab2$p_q <- NAFLD_Dx_adult_rec_ethnic[["pval.Q.w"]]

NAFLD_Dx_adult_rec_tab2$group <- row.names(NAFLD_Dx_adult_rec_tab2)

NAFLD_Dx_adult_rec_tab2$model <- "Recessive"

NAFLD_Dx_adult_rec_tab2$outcome <- "NAFLD_Dx"


** additive - overall

NAFLD_Dx_adult_add_tab <- data.frame(NAFLD_Dx_adult_add_ethnic[["TE.random"]])

row.names(NAFLD_Dx_adult_add_tab) <- "Overall"

NAFLD_Dx_adult_add_tab$OR <- NAFLD_Dx_adult_add_tab$NAFLD_Dx_adult_add_ethnic...TE.random...

NAFLD_Dx_adult_add_tab$OR <- exp(NAFLD_Dx_adult_add_tab$OR)

NAFLD_Dx_adult_add_tab <- NAFLD_Dx_adult_add_tab[-c(1)]

NAFLD_Dx_adult_add_tab$lower <- NAFLD_Dx_adult_add_ethnic[["lower.random"]]
```

```
NAFLD_Dx_adult_add_tab$lower <- exp(NAFLD_Dx_adult_add_tab$lower)

NAFLD_Dx_adult_add_tab$upper <- NAFLD_Dx_adult_add_ethnic[["upper.random"]]

NAFLD_Dx_adult_add_tab$upper <- exp(NAFLD_Dx_adult_add_tab$upper)

NAFLD_Dx_adult_add_tab$k <- NAFLD_Dx_adult_add_ethnic[["k"]]

NAFLD_Dx_adult_add_tab$p_z <- NAFLD_Dx_adult_add_ethnic[["pval.random"]]

NAFLD_Dx_adult_add_tab$I2 <- NAFLD_Dx_adult_add_ethnic[["I2"]]

NAFLD_Dx_adult_add_tab$p_q <- NAFLD_Dx_adult_add_ethnic[["pval.Q"]]

NAFLD_Dx_adult_add_tab$group <- "Overall"

NAFLD_Dx_adult_add_tab$model <- "Additive"

NAFLD_Dx_adult_add_tab$outcome <- "NAFLD_Dx"


** additive - subgroups

NAFLD_Dx_adult_add_tab2 <- data.frame(NAFLD_Dx_adult_add_ethnic[["TE.random.w"]])

row.names(NAFLD_Dx_adult_add_tab2) <- NAFLD_Dx_adult_add_ethnic[["bylevs"]]

NAFLD_Dx_adult_add_tab2$OR <- NAFLD_Dx_adult_add_tab2$NAFLD_Dx_adult_add_ethnic...TE.random.w...

NAFLD_Dx_adult_add_tab2$OR <- exp(NAFLD_Dx_adult_add_tab2$OR)

NAFLD_Dx_adult_add_tab2 <- NAFLD_Dx_adult_add_tab2[-c(1)]

NAFLD_Dx_adult_add_tab2$lower <- NAFLD_Dx_adult_add_ethnic[["lower.random.w"]]
```

```
NAFLD_Dx_adult_add_tab2$lower <- exp(NAFLD_Dx_adult_add_tab2$lower)

NAFLD_Dx_adult_add_tab2$upper <- NAFLD_Dx_adult_add_ethnic[["upper.random.w"]]

NAFLD_Dx_adult_add_tab2$upper <- exp(NAFLD_Dx_adult_add_tab2$upper)

NAFLD_Dx_adult_add_tab2$k <- NAFLD_Dx_adult_add_ethnic[["k.w"]]

NAFLD_Dx_adult_add_tab2$p_z <- NAFLD_Dx_adult_add_ethnic[["pval.random.w"]]

NAFLD_Dx_adult_add_tab2$I2 <- NAFLD_Dx_adult_add_ethnic[["I2.w"]]

NAFLD_Dx_adult_add_tab2$p_q <- NAFLD_Dx_adult_add_ethnic[["pval.Q.w"]]

NAFLD_Dx_adult_add_tab2$group <- row.names(NAFLD_Dx_adult_add_tab2)

NAFLD_Dx_adult_add_tab2$model <- "Additive"

NAFLD_Dx_adult_add_tab2$outcome <- "NAFLD_Dx"


** dominant - overall

NAFLD_Dx_adult_dom_tab <- data.frame(NAFLD_Dx_adult_dom_ethnic[["TE.random"]])

row.names(NAFLD_Dx_adult_dom_tab) <- "Overall"

NAFLD_Dx_adult_dom_tab$OR <- NAFLD_Dx_adult_dom_tab$NAFLD_Dx_adult_dom_ethnic...TE.random...

NAFLD_Dx_adult_dom_tab$OR <- exp(NAFLD_Dx_adult_dom_tab$OR)

NAFLD_Dx_adult_dom_tab <- NAFLD_Dx_adult_dom_tab[-c(1)]

NAFLD_Dx_adult_dom_tab$lower <- NAFLD_Dx_adult_dom_ethnic[["lower.random"]]
```

```
NAFLD_Dx_adult_dom_tab$lower <- exp(NAFLD_Dx_adult_dom_tab$lower)

NAFLD_Dx_adult_dom_tab$upper <- NAFLD_Dx_adult_dom_ethnic[["upper.random"]]

NAFLD_Dx_adult_dom_tab$upper <- exp(NAFLD_Dx_adult_dom_tab$upper)

NAFLD_Dx_adult_dom_tab$k <- NAFLD_Dx_adult_dom_ethnic[["k"]]

NAFLD_Dx_adult_dom_tab$p_z <- NAFLD_Dx_adult_dom_ethnic[["pval.random"]]

NAFLD_Dx_adult_dom_tab$I2 <- NAFLD_Dx_adult_dom_ethnic[["I2"]]

NAFLD_Dx_adult_dom_tab$p_q <- NAFLD_Dx_adult_dom_ethnic[["pval.Q"]]

NAFLD_Dx_adult_dom_tab$group <- "Overall"

NAFLD_Dx_adult_dom_tab$model <- "Dominant"

NAFLD_Dx_adult_dom_tab$outcome <- "NAFLD_Dx"


** dominant - subgroups

NAFLD_Dx_adult_dom_tab2 <- data.frame(NAFLD_Dx_adult_dom_ethnic[["TE.random.w"]])

row.names(NAFLD_Dx_adult_dom_tab2) <- NAFLD_Dx_adult_dom_ethnic[["bylevs"]]

NAFLD_Dx_adult_dom_tab2$OR <- NAFLD_Dx_adult_dom_tab2$NAFLD_Dx_adult_dom_ethnic...TE.random.w...

NAFLD_Dx_adult_dom_tab2$OR <- exp(NAFLD_Dx_adult_dom_tab2$OR)

NAFLD_Dx_adult_dom_tab2 <- NAFLD_Dx_adult_dom_tab2[-c(1)]

NAFLD_Dx_adult_dom_tab2$lower <- NAFLD_Dx_adult_dom_ethnic[["lower.random.w"]]
```

```
NAFLD_Dx_adult_dom_tab2$lower <- exp(NAFLD_Dx_adult_dom_tab2$lower)
NAFLD_Dx_adult_dom_tab2$upper <- NAFLD_Dx_adult_dom_ethnic[["upper.random.w"]]
NAFLD_Dx_adult_dom_tab2$upper <- exp(NAFLD_Dx_adult_dom_tab2$upper)
NAFLD_Dx_adult_dom_tab2$k <- NAFLD_Dx_adult_dom_ethnic[["k.w"]]
NAFLD_Dx_adult_dom_tab2$p_z <- NAFLD_Dx_adult_dom_ethnic[["pval.random.w"]]
NAFLD_Dx_adult_dom_tab2$I2 <- NAFLD_Dx_adult_dom_ethnic[["I2.w"]]
NAFLD_Dx_adult_dom_tab2$p_q <- NAFLD_Dx_adult_dom_ethnic[["pval.Q.w"]]
NAFLD_Dx_adult_dom_tab2$group <- row.names(NAFLD_Dx_adult_dom_tab2)
NAFLD_Dx_adult_dom_tab2$model <- "Dominant"
NAFLD_Dx_adult_dom_tab2$outcome <- "NAFLD_Dx"

NAFLD_Dx_sumtab <- rbind(NAFLD_Dx_adult_rec_tab, NAFLD_Dx_adult_rec_tab2, NAFLD_Dx_adult_add_tab,
NAFLD_Dx_adult_add_tab2, NAFLD_Dx_adult_dom_tab, NAFLD_Dx_adult_dom_tab2)

pdf(file="NAFLD_Dx_adult_rec_ethnic.pdf",width=10,height=8)
NAFLD_Dx_adult_rec_ethnic_forest <- forest(NAFLD_Dx_adult_rec_ethnic, comb.fixed=FALSE, col.random="red",
col.diamond="red", xlab="Odds of NAFLD", digits.se=2, text.random = "Recessive model - overall effect", leftcols = c("studlab", "TT
cases", "CT+CC cases", "TT controls", "CT+CC controls"), leftlabs = c("Study", "TT", "CT+CC", "TT", "CT+CC"), lab.e = "NAFLD",
```

```
lab.c = "No NAFLD", digits.addcols.left=0, just.studlab="left", just.addcols.left="right", lab.e.attach.to.col="CT+CC cases",
lab.c.attach.to.col="CT+CC controls", sortvar=rec_logOR, scientific.pval=TRUE, digits.pval=2, test.overall.random=TRUE,
xlim=c(0.1,10), test.effect.subgroup.random=TRUE)
dev.off()

pdf(file="NAFLD_Dx_adult_rec_mod.pdf",width=10,height=12)
NAFLD_Dx_adult_rec_mod_forest <- forest(NAFLD_Dx_adult_rec_mod, comb.fixed=FALSE, col.random="red",
col.diamond="red", xlab="Odds of NAFLD", digits.se=2, text.random = "Recessive model - overall effect", leftcols = c("studlab", "TT
cases", "CT+CC cases", "TT controls", "CT+CC controls"), leftlabs = c("Study", "TT", "CT+CC", "TT", "CT+CC"), lab.e = "NAFLD",
lab.c = "No NAFLD", digits.addcols.left=0, just.studlab="left", just.addcols.left="right", lab.e.attach.to.col="CT+CC cases",
lab.c.attach.to.col="CT+CC controls", sortvar=rec_logOR, scientific.pval=TRUE, digits.pval=2, test.overall.random=TRUE,
xlim=c(0.1,10), test.effect.subgroup.random=TRUE)
dev.off()

**Re-run using recessive model after excluding 3 studies with potential differences between controls & cases

NAFLD_Dx_adult_exclconf <- read_excel("NAFLD_Dx_adult_exclconf.xlsx")
```

```
NAFLD_Dx_adult_rec_exclconf <- metagen(rec_logOR, rec_seOR, studlab = Author, method.tau = "DL", sm = "OR", data = 
NAFLD_Dx_adult_exclconf)
NAFLD_Dx_adult_rec_ethnic_exclconf <- update(NAFLD_Dx_adult_rec_exclconf, byvar = Ethnicity, bylab = "Ethnicity")

sink("NAFLD_Dx_adult_rec_ethnic_exclconf.txt")
print(NAFLD_Dx_adult_rec_ethnic_exclconf)
sink()

pdf(file="NAFLD_Dx_adult_rec_ethnic_exclconf.pdf",width=10,height=8)
NAFLD_Dx_adult_rec_ethnic_forest <- forest(NAFLD_Dx_adult_rec_ethnic_exclconf, comb.fixed=FALSE, col.random="red", 
col.diamond="red", xlab="Odds of NAFLD", digits.se=2, text.random = "Recessive model - overall effect", leftcols = c("studlab", "TT 
cases", "CT+CC cases", "TT controls", "CT+CC controls"), leftlabs = c("Study", "TT", "CT+CC", "TT", "CT+CC"), lab.e = "NAFLD", 
lab.c = "No NAFLD", digits.addcols.left=0, just.studlab="left", just.addcols.left="right", lab.e.attach.to.col="CT+CC cases", 
lab.c.attach.to.col="CT+CC controls", sortvar=rec_logOR, scientific.pval=TRUE, digits.pval=2, test.overall.random=TRUE, 
xlim=c(0.1,10), test.effect.subgroup.random=TRUE)
dev.off()

**NAFLD Diagnosis in children
```

```
NAFLD_Dx_paed <- read_excel("NAFLD_Dx_paed.xlsx")

NAFLD_Dx_paed_add <- metagen(add_logOR, add_seOR, studlab = Author, method.tau = "DL", sm = "OR", data =
NAFLD_Dx_paed)

NAFLD_Dx_paed_rec <- metagen(rec_logOR, rec_seOR, studlab = Author, method.tau = "DL", sm = "OR", data =
NAFLD_Dx_paed)

NAFLD_Dx_paed_dom <- metagen(dom_logOR, dom_seOR, studlab = Author, method.tau = "DL", sm = "OR", data =
NAFLD_Dx_paed)



** recessive - overall

NAFLD_Dx_paed_rec_tab <- data.frame(NAFLD_Dx_paed_rec[["TE.random"]])

row.names(NAFLD_Dx_paed_rec_tab) <- "Overall"

NAFLD_Dx_paed_rec_tab$OR <- NAFLD_Dx_paed_rec_tab$NAFLD_Dx_paed_rec...TE.random...

NAFLD_Dx_paed_rec_tab$OR <- exp(NAFLD_Dx_paed_rec_tab$OR)

NAFLD_Dx_paed_rec_tab <- NAFLD_Dx_paed_rec_tab[-c(1)]

NAFLD_Dx_paed_rec_tab$lower <- NAFLD_Dx_paed_rec[["lower.random"]]

NAFLD_Dx_paed_rec_tab$lower <- exp(NAFLD_Dx_paed_rec_tab$lower)
```

```
NAFLD_Dx_paed_rec_tab$upper <- NAFLD_Dx_paed_rec[["upper.random"]]

NAFLD_Dx_paed_rec_tab$upper <- exp(NAFLD_Dx_paed_rec_tab$upper)

NAFLD_Dx_paed_rec_tab$k <- NAFLD_Dx_paed_rec[["k"]]

NAFLD_Dx_paed_rec_tab$p_z <- NAFLD_Dx_paed_rec[["pval.random"]]

NAFLD_Dx_paed_rec_tab$I2 <- NAFLD_Dx_paed_rec[["I2"]]

NAFLD_Dx_paed_rec_tab$p_q <- NAFLD_Dx_paed_rec[["pval.Q"]]

NAFLD_Dx_paed_rec_tab$group <- "Overall"

NAFLD_Dx_paed_rec_tab$model <- "Recessive"

NAFLD_Dx_paed_rec_tab$outcome <- "NAFLD_Dx"


** additive - overall

NAFLD_Dx_paed_add_tab <- data.frame(NAFLD_Dx_paed_add[["TE.random"]])

row.names(NAFLD_Dx_paed_add_tab) <- "Overall"

NAFLD_Dx_paed_add_tab$OR <- NAFLD_Dx_paed_add_tab$NAFLD_Dx_paed_add...TE.random...

NAFLD_Dx_paed_add_tab$OR <- exp(NAFLD_Dx_paed_add_tab$OR)

NAFLD_Dx_paed_add_tab <- NAFLD_Dx_paed_add_tab[-c(1)]

NAFLD_Dx_paed_add_tab$lower <- NAFLD_Dx_paed_add[["lower.random"]]

NAFLD_Dx_paed_add_tab$lower <- exp(NAFLD_Dx_paed_add_tab$lower)
```

```
NAFLD_Dx_paed_add_tab$upper <- NAFLD_Dx_paed_add[["upper.random"]]

NAFLD_Dx_paed_add_tab$upper <- exp(NAFLD_Dx_paed_add_tab$upper)

NAFLD_Dx_paed_add_tab$k <- NAFLD_Dx_paed_add[["k"]]

NAFLD_Dx_paed_add_tab$p_z <- NAFLD_Dx_paed_add[["pval.random"]]

NAFLD_Dx_paed_add_tab$I2 <- NAFLD_Dx_paed_add[["I2"]]

NAFLD_Dx_paed_add_tab$p_q <- NAFLD_Dx_paed_add[["pval.Q"]]

NAFLD_Dx_paed_add_tab$group <- "Overall"

NAFLD_Dx_paed_add_tab$model <- "Additive"

NAFLD_Dx_paed_add_tab$outcome <- "NAFLD_Dx"


** dominant - overall

NAFLD_Dx_paed_dom_tab <- data.frame(NAFLD_Dx_paed_dom[["TE.random"]])

row.names(NAFLD_Dx_paed_dom_tab) <- "Overall"

NAFLD_Dx_paed_dom_tab$OR <- NAFLD_Dx_paed_dom_tab$NAFLD_Dx_paed_dom...TE.random...

NAFLD_Dx_paed_dom_tab$OR <- exp(NAFLD_Dx_paed_dom_tab$OR)

NAFLD_Dx_paed_dom_tab <- NAFLD_Dx_paed_dom_tab[-c(1)]

NAFLD_Dx_paed_dom_tab$lower <- NAFLD_Dx_paed_dom[["lower.random"]]

NAFLD_Dx_paed_dom_tab$lower <- exp(NAFLD_Dx_paed_dom_tab$lower)
```

```
NAFLD_Dx_paed_dom_tab$upper <- NAFLD_Dx_paed_dom[["upper.random"]]

NAFLD_Dx_paed_dom_tab$upper <- exp(NAFLD_Dx_paed_dom_tab$upper)

NAFLD_Dx_paed_dom_tab$k <- NAFLD_Dx_paed_dom[["k"]]

NAFLD_Dx_paed_dom_tab$p_z <- NAFLD_Dx_paed_dom[["pval.random"]]

NAFLD_Dx_paed_dom_tab$I2 <- NAFLD_Dx_paed_dom[["I2"]]

NAFLD_Dx_paed_dom_tab$p_q <- NAFLD_Dx_paed_dom[["pval.Q"]]

NAFLD_Dx_paed_dom_tab$group <- "Overall"

NAFLD_Dx_paed_dom_tab$model <- "Dominant"

NAFLD_Dx_paed_dom_tab$outcome <- "NAFLD_Dx"


NAFLD_Dx_paed_sumtab <- rbind(NAFLD_Dx_paed_rec_tab, NAFLD_Dx_paed_add_tab, NAFLD_Dx_paed_dom_tab)



**Presence of severe steatosis in adults


Steat_adult <- read_excel("Steat_adult.xlsx")

Steat_adult_add <- metagen(add_logOR, add_seOR, studlab = Author, method.tau = "DL", sm = "OR", data = Steat_adult)

Steat_adult_add_ethnic <- update(Steat_adult_add, byvar = Ethnicity, bylab = "Ethnicity")
```

```
Steat_adult_rec <- metagen(rec_logOR, rec_seOR, studlab = Author, method.tau = "DL", sm = "OR", data = Steat_adult)

Steat_adult_rec_ethnic <- update(Steat_adult_rec, byvar = Ethnicity, bylab = "Ethnicity")

Steat_adult_dom <- metagen(dom_logOR, dom_seOR, studlab = Author, method.tau = "DL", sm = "OR", data = Steat_adult)

Steat_adult_dom_ethnic <- update(Steat_adult_dom, byvar = Ethnicity, bylab = "Ethnicity")


** recessive - overall

Steat_adult_rec_tab <- data.frame(Steat_adult_rec_ethnic[["TE.random"]])

row.names(Steat_adult_rec_tab) <- "Overall"

Steat_adult_rec_tab$OR <- Steat_adult_rec_tab$Steat_adult_rec_ethnic...TE.random...

Steat_adult_rec_tab$OR <- exp(Steat_adult_rec_tab$OR)

Steat_adult_rec_tab <- Steat_adult_rec_tab[-c(1)]

Steat_adult_rec_tab$lower <- Steat_adult_rec_ethnic[["lower.random"]]

Steat_adult_rec_tab$lower <- exp(Steat_adult_rec_tab$lower)

Steat_adult_rec_tab$upper <- Steat_adult_rec_ethnic[["upper.random"]]

Steat_adult_rec_tab$upper <- exp(Steat_adult_rec_tab$upper)

Steat_adult_rec_tab$k <- Steat_adult_rec_ethnic[["k"]]

Steat_adult_rec_tab$p_z <- Steat_adult_rec_ethnic[["pval.random"]]

Steat_adult_rec_tab$I2 <- Steat_adult_rec_ethnic[["I2"]]
```

```
Steat_adult_rec_tab$p_q <- Steat_adult_rec_ethnic[["pval.Q"]]

Steat_adult_rec_tab$group <- "Overall"

Steat_adult_rec_tab$model <- "Recessive"

Steat_adult_rec_tab$outcome <- "Steat"


** recessive - subgroups

Steat_adult_rec_tab2 <- data.frame(Steat_adult_rec_ethnic[["TE.random.w"]])

row.names(Steat_adult_rec_tab2) <- Steat_adult_rec_ethnic[["bylevs"]]

Steat_adult_rec_tab2$OR <- Steat_adult_rec_tab2$Steat_adult_rec_ethnic...TE.random.w...

Steat_adult_rec_tab2$OR <- exp(Steat_adult_rec_tab2$OR)

Steat_adult_rec_tab2 <- Steat_adult_rec_tab2[-c(1)]

Steat_adult_rec_tab2$lower <- Steat_adult_rec_ethnic[["lower.random.w"]]

Steat_adult_rec_tab2$lower <- exp(Steat_adult_rec_tab2$lower)

Steat_adult_rec_tab2$upper <- Steat_adult_rec_ethnic[["upper.random.w"]]

Steat_adult_rec_tab2$upper <- exp(Steat_adult_rec_tab2$upper)

Steat_adult_rec_tab2$k <- Steat_adult_rec_ethnic[["k.w"]]

Steat_adult_rec_tab2$p_z <- Steat_adult_rec_ethnic[["pval.random.w"]]

Steat_adult_rec_tab2$I2 <- Steat_adult_rec_ethnic[["I2.w"]]
```

```
Steat_adult_rec_tab2$p_q <- Steat_adult_rec_ethnic[["pval.Q.w"]]

Steat_adult_rec_tab2$group <- row.names(Steat_adult_rec_tab2)

Steat_adult_rec_tab2$model <- "Recessive"

Steat_adult_rec_tab2$outcome <- "Steat"


** additive - overall

Steat_adult_add_tab <- data.frame(Steat_adult_add_ethnic[["TE.random"]])

row.names(Steat_adult_add_tab) <- "Overall"

Steat_adult_add_tab$OR <- Steat_adult_add_tab$Steat_adult_add_ethnic...TE.random...

Steat_adult_add_tab$OR <- exp(Steat_adult_add_tab$OR)

Steat_adult_add_tab <- Steat_adult_add_tab[-c(1)]

Steat_adult_add_tab$lower <- Steat_adult_add_ethnic[["lower.random"]]

Steat_adult_add_tab$lower <- exp(Steat_adult_add_tab$lower)

Steat_adult_add_tab$upper <- Steat_adult_add_ethnic[["upper.random"]]

Steat_adult_add_tab$upper <- exp(Steat_adult_add_tab$upper)

Steat_adult_add_tab$k <- Steat_adult_add_ethnic[["k"]]

Steat_adult_add_tab$p_z <- Steat_adult_add_ethnic[["pval.random"]]

Steat_adult_add_tab$I2 <- Steat_adult_add_ethnic[["I2"]]
```

```
Steat_adult_add_tab$p_q <- Steat_adult_add_ethnic[["pval.Q"]]

Steat_adult_add_tab$group <- "Overall"

Steat_adult_add_tab$model <- "Additive"

Steat_adult_add_tab$outcome <- "Steat"


** additive - subgroups

Steat_adult_add_tab2 <- data.frame(Steat_adult_add_ethnic[["TE.random.w"]])

row.names(Steat_adult_add_tab2) <- Steat_adult_add_ethnic[["bylevs"]]

Steat_adult_add_tab2$OR <- Steat_adult_add_tab2$Steat_adult_add_ethnic...TE.random.w...

Steat_adult_add_tab2$OR <- exp(Steat_adult_add_tab2$OR)

Steat_adult_add_tab2 <- Steat_adult_add_tab2[-c(1)]

Steat_adult_add_tab2$lower <- Steat_adult_add_ethnic[["lower.random.w"]]

Steat_adult_add_tab2$lower <- exp(Steat_adult_add_tab2$lower)

Steat_adult_add_tab2$upper <- Steat_adult_add_ethnic[["upper.random.w"]]

Steat_adult_add_tab2$upper <- exp(Steat_adult_add_tab2$upper)

Steat_adult_add_tab2$k <- Steat_adult_add_ethnic[["k.w"]]

Steat_adult_add_tab2$p_z <- Steat_adult_add_ethnic[["pval.random.w"]]

Steat_adult_add_tab2$I2 <- Steat_adult_add_ethnic[["I2.w"]]
```

```
Steat_adult_add_tab2$p_q <- Steat_adult_add_ethnic[["pval.Q.w"]]

Steat_adult_add_tab2$group <- row.names(Steat_adult_add_tab2)

Steat_adult_add_tab2$model <- "Additive"

Steat_adult_add_tab2$outcome <- "Steat"


** dominant - overall

Steat_adult_dom_tab <- data.frame(Steat_adult_dom_ethnic[["TE.random"]])

row.names(Steat_adult_dom_tab) <- "Overall"

Steat_adult_dom_tab$OR <- Steat_adult_dom_tab$Steat_adult_dom_ethnic...TE.random...

Steat_adult_dom_tab$OR <- exp(Steat_adult_dom_tab$OR)

Steat_adult_dom_tab <- Steat_adult_dom_tab[-c(1)]

Steat_adult_dom_tab$lower <- Steat_adult_dom_ethnic[["lower.random"]]

Steat_adult_dom_tab$lower <- exp(Steat_adult_dom_tab$lower)

Steat_adult_dom_tab$upper <- Steat_adult_dom_ethnic[["upper.random"]]

Steat_adult_dom_tab$upper <- exp(Steat_adult_dom_tab$upper)

Steat_adult_dom_tab$k <- Steat_adult_dom_ethnic[["k"]]

Steat_adult_dom_tab$p_z <- Steat_adult_dom_ethnic[["pval.random"]]

Steat_adult_dom_tab$I2 <- Steat_adult_dom_ethnic[["I2"]]
```

```
Steat_adult_dom_tab$p_q <- Steat_adult_dom_ethnic[["pval.Q"]]

Steat_adult_dom_tab$group <- "Overall"

Steat_adult_dom_tab$model <- "Dominant"

Steat_adult_dom_tab$outcome <- "Steat"


** dominant - subgroups

Steat_adult_dom_tab2 <- data.frame(Steat_adult_dom_ethnic[["TE.random.w"]])

row.names(Steat_adult_dom_tab2) <- Steat_adult_dom_ethnic[["bylevs"]]

Steat_adult_dom_tab2$OR <- Steat_adult_dom_tab2$Steat_adult_dom_ethnic...TE.random.w...

Steat_adult_dom_tab2$OR <- exp(Steat_adult_dom_tab2$OR)

Steat_adult_dom_tab2 <- Steat_adult_dom_tab2[-c(1)]

Steat_adult_dom_tab2$lower <- Steat_adult_dom_ethnic[["lower.random.w"]]

Steat_adult_dom_tab2$lower <- exp(Steat_adult_dom_tab2$lower)

Steat_adult_dom_tab2$upper <- Steat_adult_dom_ethnic[["upper.random.w"]]

Steat_adult_dom_tab2$upper <- exp(Steat_adult_dom_tab2$upper)

Steat_adult_dom_tab2$k <- Steat_adult_dom_ethnic[["k.w"]]

Steat_adult_dom_tab2$p_z <- Steat_adult_dom_ethnic[["pval.random.w"]]

Steat_adult_dom_tab2$I2 <- Steat_adult_dom_ethnic[["I2.w"]]
```

```
Steat_adult_dom_tab2$p_q <- Steat_adult_dom_ethnic[["pval.Q.w"]]

Steat_adult_dom_tab2$group <- row.names(Steat_adult_dom_tab2)

Steat_adult_dom_tab2$model <- "Dominant"

Steat_adult_dom_tab2$outcome <- "Steat"


Steat_sumtab <- rbind(Steat_adult_rec_tab, Steat_adult_rec_tab2, Steat_adult_add_tab, Steat_adult_add_tab2,
Steat_adult_dom_tab, Steat_adult_dom_tab2)


pdf(file="Steat_adult_rec_ethnic.pdf",width=10,height=8)

Steat_adult_rec_ethnic_forest <- forest(Steat_adult_rec_ethnic, comb.fixed=FALSE, col.random="red", col.diamond="red",
xlab="Odds of severe steatosis", digits.se=2, text.random = "Recessive model - overall effect", leftcols = c("studlab", "TT cases",
"CT+CC cases", "TT controls", "CT+CC controls"), leftlabs = c("Study", "TT", "CT+CC", "TT", "CT+CC"), lab.e = "S3", lab.c = "S1-
2", digits.addcols.left=0, just.studlab="left", just.addcols.left="right", lab.e.attach.to.col="CT+CC cases", lab.c.attach.to.col="CT+CC
controls", sortvar=rec_logOR, digits.pval=3, test.overall.random=TRUE, test.effect.subgroup.random=TRUE)
dev.off()



**Presence of severe steatosis  in children
```

```
Steat_paed <- read_excel("Steat_paed.xlsx")

Steat_paed_add <- metagen(add_logOR, add_seOR, studlab = Author, method.tau = "DL", sm = "OR", data = Steat_paed)

Steat_paed_rec <- metagen(rec_logOR, rec_seOR, studlab = Author, method.tau = "DL", sm = "OR", data = Steat_paed)

Steat_paed_dom <- metagen(dom_logOR, dom_seOR, studlab = Author, method.tau = "DL", sm = "OR", data = Steat_paed)


** recessive - overall

Steat_paed_rec_tab <- data.frame(Steat_paed_rec[["TE.random"]])

row.names(Steat_paed_rec_tab) <- "Overall"

Steat_paed_rec_tab$OR <- Steat_paed_rec_tab$Steat_paed_rec...TE.random...

Steat_paed_rec_tab$OR <- exp(Steat_paed_rec_tab$OR)

Steat_paed_rec_tab <- Steat_paed_rec_tab[-c(1)]

Steat_paed_rec_tab$lower <- Steat_paed_rec[["lower.random"]]

Steat_paed_rec_tab$lower <- exp(Steat_paed_rec_tab$lower)

Steat_paed_rec_tab$upper <- Steat_paed_rec[["upper.random"]]

Steat_paed_rec_tab$upper <- exp(Steat_paed_rec_tab$upper)

Steat_paed_rec_tab$k <- Steat_paed_rec[["k"]]

Steat_paed_rec_tab$p_z <- Steat_paed_rec[["pval.random"]]
```

```
Steat_paed_rec_tab$I2 <- Steat_paed_rec[["I2"]]

Steat_paed_rec_tab$p_q <- Steat_paed_rec[["pval.Q"]]

Steat_paed_rec_tab$group <- "Overall"

Steat_paed_rec_tab$model <- "Recessive"

Steat_paed_rec_tab$outcome <- "Steat"


** additive - overall

Steat_paed_add_tab <- data.frame(Steat_paed_add[["TE.random"]])

row.names(Steat_paed_add_tab) <- "Overall"

Steat_paed_add_tab$OR <- Steat_paed_add_tab$Steat_paed_add...TE.random...

Steat_paed_add_tab$OR <- exp(Steat_paed_add_tab$OR)

Steat_paed_add_tab <- Steat_paed_add_tab[-c(1)]

Steat_paed_add_tab$lower <- Steat_paed_add[["lower.random"]]

Steat_paed_add_tab$lower <- exp(Steat_paed_add_tab$lower)

Steat_paed_add_tab$upper <- Steat_paed_add[["upper.random"]]

Steat_paed_add_tab$upper <- exp(Steat_paed_add_tab$upper)

Steat_paed_add_tab$k <- Steat_paed_add[["k"]]

Steat_paed_add_tab$p_z <- Steat_paed_add[["pval.random"]]
```

```
Steat_paed_add_tab$I2 <- Steat_paed_add[["I2"]]

Steat_paed_add_tab$p_q <- Steat_paed_add[["pval.Q"]]

Steat_paed_add_tab$group <- "Overall"

Steat_paed_add_tab$model <- "Additive"

Steat_paed_add_tab$outcome <- "Steat"


** dominant - overall

Steat_paed_dom_tab <- data.frame(Steat_paed_dom[["TE.random"]])

row.names(Steat_paed_dom_tab) <- "Overall"

Steat_paed_dom_tab$OR <- Steat_paed_dom_tab$Steat_paed_dom...TE.random...

Steat_paed_dom_tab$OR <- exp(Steat_paed_dom_tab$OR)

Steat_paed_dom_tab <- Steat_paed_dom_tab[-c(1)]

Steat_paed_dom_tab$lower <- Steat_paed_dom[["lower.random"]]

Steat_paed_dom_tab$lower <- exp(Steat_paed_dom_tab$lower)

Steat_paed_dom_tab$upper <- Steat_paed_dom[["upper.random"]]

Steat_paed_dom_tab$upper <- exp(Steat_paed_dom_tab$upper)

Steat_paed_dom_tab$k <- Steat_paed_dom[["k"]]

Steat_paed_dom_tab$p_z <- Steat_paed_dom[["pval.random"]]
```

```
Steat_paed_dom_tab$I2 <- Steat_paed_dom[["I2"]]

Steat_paed_dom_tab$p_q <- Steat_paed_dom[["pval.Q"]]

Steat_paed_dom_tab$group <- "Overall"

Steat_paed_dom_tab$model <- "Dominant"

Steat_paed_dom_tab$outcome <- "Steat"


Steat_paed_sumtab <- rbind(Steat_paed_rec_tab, Steat_paed_add_tab, Steat_paed_dom_tab)
```

**Presence of NASH in adults

```
NASH_adult <- read_excel("NASH_adult.xlsx")

NASH_adult_add <- metagen(add_logOR, add_seOR, studlab = Author, method.tau = "DL", sm = "OR", data = NASH_adult)

NASH_adult_add_ethnic <- update(NASH_adult_add, byvar = Ethnicity, bylab = "Ethnicity")

NASH_adult_rec <- metagen(rec_logOR, rec_seOR, studlab = Author, method.tau = "DL", sm = "OR", data = NASH_adult)

NASH_adult_rec_ethnic <- update(NASH_adult_rec, byvar = Ethnicity, bylab = "Ethnicity")

NASH_adult_dom <- metagen(dom_logOR, dom_seOR, studlab = Author, method.tau = "DL", sm = "OR", data = NASH_adult)
```

```
NASH_adult_dom_ethnic <- update(NASH_adult_dom, byvar = Ethnicity, bylab = "Ethnicity")


** recessive - overall

NASH_adult_rec_tab <- data.frame(NASH_adult_rec_ethnic[["TE.random"]])

row.names(NASH_adult_rec_tab) <- "Overall"

NASH_adult_rec_tab$OR <- NASH_adult_rec_tab$NASH_adult_rec_ethnic...TE.random...

NASH_adult_rec_tab$OR <- exp(NASH_adult_rec_tab$OR)

NASH_adult_rec_tab <- NASH_adult_rec_tab[-c(1)]

NASH_adult_rec_tab$lower <- NASH_adult_rec_ethnic[["lower.random"]]

NASH_adult_rec_tab$lower <- exp(NASH_adult_rec_tab$lower)

NASH_adult_rec_tab$upper <- NASH_adult_rec_ethnic[["upper.random"]]

NASH_adult_rec_tab$upper <- exp(NASH_adult_rec_tab$upper)

NASH_adult_rec_tab$k <- NASH_adult_rec_ethnic[["k"]]

NASH_adult_rec_tab$p_z <- NASH_adult_rec_ethnic[["pval.random"]]

NASH_adult_rec_tab$I2 <- NASH_adult_rec_ethnic[["I2"]]

NASH_adult_rec_tab$p_q <- NASH_adult_rec_ethnic[["pval.Q"]]

NASH_adult_rec_tab$group <- "Overall"

NASH_adult_rec_tab$model <- "Recessive"
```

```
NASH_adult_rec_tab$outcome <- "NASH"


** recessive - subgroups

NASH_adult_rec_tab2 <- data.frame(NASH_adult_rec_ethnic[["TE.random.w"]])

row.names(NASH_adult_rec_tab2) <- NASH_adult_rec_ethnic[["bylevs"]]

NASH_adult_rec_tab2$OR <- NASH_adult_rec_tab2$NASH_adult_rec_ethnic...TE.random.w...

NASH_adult_rec_tab2$OR <- exp(NASH_adult_rec_tab2$OR)

NASH_adult_rec_tab2 <- NASH_adult_rec_tab2[-c(1)]

NASH_adult_rec_tab2$lower <- NASH_adult_rec_ethnic[["lower.random.w"]]

NASH_adult_rec_tab2$lower <- exp(NASH_adult_rec_tab2$lower)

NASH_adult_rec_tab2$upper <- NASH_adult_rec_ethnic[["upper.random.w"]]

NASH_adult_rec_tab2$upper <- exp(NASH_adult_rec_tab2$upper)

NASH_adult_rec_tab2$k <- NASH_adult_rec_ethnic[["k.w"]]

NASH_adult_rec_tab2$p_z <- NASH_adult_rec_ethnic[["pval.random.w"]]

NASH_adult_rec_tab2$I2 <- NASH_adult_rec_ethnic[["I2.w"]]

NASH_adult_rec_tab2$p_q <- NASH_adult_rec_ethnic[["pval.Q.w"]]

NASH_adult_rec_tab2$group <- row.names(NASH_adult_rec_tab2)

NASH_adult_rec_tab2$model <- "Recessive"
```

```
NASH_adult_rec_tab2$outcome <- "NASH"


** additive - overall

NASH_adult_add_tab <- data.frame(NASH_adult_add_ethnic[["TE.random"]])

row.names(NASH_adult_add_tab) <- "Overall"

NASH_adult_add_tab$OR <- NASH_adult_add_tab$NASH_adult_add_ethnic...TE.random...

NASH_adult_add_tab$OR <- exp(NASH_adult_add_tab$OR)

NASH_adult_add_tab <- NASH_adult_add_tab[-c(1)]

NASH_adult_add_tab$lower <- NASH_adult_add_ethnic[["lower.random"]]

NASH_adult_add_tab$lower <- exp(NASH_adult_add_tab$lower)

NASH_adult_add_tab$upper <- NASH_adult_add_ethnic[["upper.random"]]

NASH_adult_add_tab$upper <- exp(NASH_adult_add_tab$upper)

NASH_adult_add_tab$k <- NASH_adult_add_ethnic[["k"]]

NASH_adult_add_tab$p_z <- NASH_adult_add_ethnic[["pval.random"]]

NASH_adult_add_tab$I2 <- NASH_adult_add_ethnic[["I2"]]

NASH_adult_add_tab$p_q <- NASH_adult_add_ethnic[["pval.Q"]]

NASH_adult_add_tab$group <- "Overall"

NASH_adult_add_tab$model <- "Additive"
```

```
NASH_adult_add_tab$outcome <- "NASH"


** additive - subgroups

NASH_adult_add_tab2 <- data.frame(NASH_adult_add_ethnic[["TE.random.w"]])

row.names(NASH_adult_add_tab2) <- NASH_adult_add_ethnic[["bylevs"]]

NASH_adult_add_tab2$OR <- NASH_adult_add_tab2$NASH_adult_add_ethnic...TE.random.w...

NASH_adult_add_tab2$OR <- exp(NASH_adult_add_tab2$OR)

NASH_adult_add_tab2 <- NASH_adult_add_tab2[-c(1)]

NASH_adult_add_tab2$lower <- NASH_adult_add_ethnic[["lower.random.w"]]

NASH_adult_add_tab2$lower <- exp(NASH_adult_add_tab2$lower)

NASH_adult_add_tab2$upper <- NASH_adult_add_ethnic[["upper.random.w"]]

NASH_adult_add_tab2$upper <- exp(NASH_adult_add_tab2$upper)

NASH_adult_add_tab2$k <- NASH_adult_add_ethnic[["k.w"]]

NASH_adult_add_tab2$p_z <- NASH_adult_add_ethnic[["pval.random.w"]]

NASH_adult_add_tab2$I2 <- NASH_adult_add_ethnic[["I2.w"]]

NASH_adult_add_tab2$p_q <- NASH_adult_add_ethnic[["pval.Q.w"]]

NASH_adult_add_tab2$group <- row.names(NASH_adult_add_tab2)

NASH_adult_add_tab2$model <- "Additive"
```

```
NASH_adult_add_tab2$outcome <- "NASH"



** dominant - overall

NASH_adult_dom_tab <- data.frame(NASH_adult_dom_ethnic[["TE.random"]])

row.names(NASH_adult_dom_tab) <- "Overall"

NASH_adult_dom_tab$OR <- NASH_adult_dom_tab$NASH_adult_dom_ethnic...TE.random...

NASH_adult_dom_tab$OR <- exp(NASH_adult_dom_tab$OR)

NASH_adult_dom_tab <- NASH_adult_dom_tab[-c(1)]

NASH_adult_dom_tab$lower <- NASH_adult_dom_ethnic[["lower.random"]]

NASH_adult_dom_tab$lower <- exp(NASH_adult_dom_tab$lower)

NASH_adult_dom_tab$upper <- NASH_adult_dom_ethnic[["upper.random"]]

NASH_adult_dom_tab$upper <- exp(NASH_adult_dom_tab$upper)

NASH_adult_dom_tab$k <- NASH_adult_dom_ethnic[["k"]]

NASH_adult_dom_tab$p_z <- NASH_adult_dom_ethnic[["pval.random"]]

NASH_adult_dom_tab$I2 <- NASH_adult_dom_ethnic[["I2"]]

NASH_adult_dom_tab$p_q <- NASH_adult_dom_ethnic[["pval.Q"]]

NASH_adult_dom_tab$group <- "Overall"
```

```
NASH_adult_dom_tab$model <- "Dominant"

NASH_adult_dom_tab$outcome <- "NASH"


** dominant - subgroups

NASH_adult_dom_tab2 <- data.frame(NASH_adult_dom_ethnic[["TE.random.w"]])

row.names(NASH_adult_dom_tab2) <- NASH_adult_dom_ethnic[["bylevs"]]

NASH_adult_dom_tab2$OR <- NASH_adult_dom_tab2$NASH_adult_dom_ethnic...TE.random.w...

NASH_adult_dom_tab2$OR <- exp(NASH_adult_dom_tab2$OR)

NASH_adult_dom_tab2 <- NASH_adult_dom_tab2[-c(1)]

NASH_adult_dom_tab2$lower <- NASH_adult_dom_ethnic[["lower.random.w"]]

NASH_adult_dom_tab2$lower <- exp(NASH_adult_dom_tab2$lower)

NASH_adult_dom_tab2$upper <- NASH_adult_dom_ethnic[["upper.random.w"]]

NASH_adult_dom_tab2$upper <- exp(NASH_adult_dom_tab2$upper)

NASH_adult_dom_tab2$k <- NASH_adult_dom_ethnic[["k.w"]]

NASH_adult_dom_tab2$p_z <- NASH_adult_dom_ethnic[["pval.random.w"]]

NASH_adult_dom_tab2$I2 <- NASH_adult_dom_ethnic[["I2.w"]]

NASH_adult_dom_tab2$p_q <- NASH_adult_dom_ethnic[["pval.Q.w"]]

NASH_adult_dom_tab2$group <- row.names(NASH_adult_dom_tab2)
```

```
NASH_adult_dom_tab2$model <- "Dominant"

NASH_adult_dom_tab2$outcome <- "NASH"


NASH_sumtab <- rbind(NASH_adult_rec_tab, NASH_adult_rec_tab2, NASH_adult_add_tab, NASH_adult_add_tab2,
NASH_adult_dom_tab, NASH_adult_dom_tab2)


pdf(file="NASH_adult_rec_ethnic.pdf",width=10,height=8)
NASH_adult_rec_ethnic_forest <- forest(NASH_adult_rec_ethnic, comb.fixed=FALSE, col.random="red", col.diamond="red",
xlab="Odds of NASH", digits.se=2, text.random = "Recessive model - overall effect", leftcols = c("studlab", "TT cases", "CT+CC
cases", "TT controls", "CT+CC controls"), leftlabs = c("Study", "TT", "CT+CC", "TT", "CT+CC"), lab.e = "NASH", lab.c = "NAFL",
digits.addcols.left=0, just.studlab="left", just.addcols.left="right", lab.e.attach.to.col="CT+CC cases", lab.c.attach.to.col="CT+CC
controls", sortvar=rec_logOR, digits.pval=3, test.overall.random=TRUE, test.effect.subgroup.random=TRUE)
dev.off()



**Presence of NASH in children


NASH_paed <- read_excel("NASH_paed.xlsx")
```

```
NASH_paed_add <- metagen(add_logOR, add_seOR, studlab = Author, method.tau = "DL", sm = "OR", data = NASH_paed)

NASH_paed_rec <- metagen(rec_logOR, rec_seOR, studlab = Author, method.tau = "DL", sm = "OR", data = NASH_paed)

NASH_paed_dom <- metagen(dom_logOR, dom_seOR, studlab = Author, method.tau = "DL", sm = "OR", data = NASH_paed)


** recessive - overall

NASH_paed_rec_tab <- data.frame(NASH_paed_rec[["TE.random"]])

row.names(NASH_paed_rec_tab) <- "Overall"

NASH_paed_rec_tab$OR <- NASH_paed_rec_tab$NASH_paed_rec...TE.random...

NASH_paed_rec_tab$OR <- exp(NASH_paed_rec_tab$OR)

NASH_paed_rec_tab <- NASH_paed_rec_tab[-c(1)]

NASH_paed_rec_tab$lower <- NASH_paed_rec[["lower.random"]]

NASH_paed_rec_tab$lower <- exp(NASH_paed_rec_tab$lower)

NASH_paed_rec_tab$upper <- NASH_paed_rec[["upper.random"]]

NASH_paed_rec_tab$upper <- exp(NASH_paed_rec_tab$upper)

NASH_paed_rec_tab$k <- NASH_paed_rec[["k"]]

NASH_paed_rec_tab$p_z <- NASH_paed_rec[["pval.random"]]

NASH_paed_rec_tab$I2 <- NASH_paed_rec[["I2"]]

NASH_paed_rec_tab$p_q <- NASH_paed_rec[["pval.Q"]]
```

```
NASH_paed_rec_tab$group <- "Overall"

NASH_paed_rec_tab$model <- "Recessive"

NASH_paed_rec_tab$outcome <- "NASH"


** additive - overall

NASH_paed_add_tab <- data.frame(NASH_paed_add[["TE.random"]])

row.names(NASH_paed_add_tab) <- "Overall"

NASH_paed_add_tab$OR <- NASH_paed_add_tab$NASH_paed_add...TE.random...

NASH_paed_add_tab$OR <- exp(NASH_paed_add_tab$OR)

NASH_paed_add_tab <- NASH_paed_add_tab[-c(1)]

NASH_paed_add_tab$lower <- NASH_paed_add[["lower.random"]]

NASH_paed_add_tab$lower <- exp(NASH_paed_add_tab$lower)

NASH_paed_add_tab$upper <- NASH_paed_add[["upper.random"]]

NASH_paed_add_tab$upper <- exp(NASH_paed_add_tab$upper)

NASH_paed_add_tab$k <- NASH_paed_add[["k"]]

NASH_paed_add_tab$p_z <- NASH_paed_add[["pval.random"]]

NASH_paed_add_tab$I2 <- NASH_paed_add[["I2"]]

NASH_paed_add_tab$p_q <- NASH_paed_add[["pval.Q"]]
```

```
NASH_paed_add_tab$group <- "Overall"

NASH_paed_add_tab$model <- "Additive"

NASH_paed_add_tab$outcome <- "NASH"


** dominant - overall

NASH_paed_dom_tab <- data.frame(NASH_paed_dom[["TE.random"]])

row.names(NASH_paed_dom_tab) <- "Overall"

NASH_paed_dom_tab$OR <- NASH_paed_dom_tab$NASH_paed_dom...TE.random...

NASH_paed_dom_tab$OR <- exp(NASH_paed_dom_tab$OR)

NASH_paed_dom_tab <- NASH_paed_dom_tab[-c(1)]

NASH_paed_dom_tab$lower <- NASH_paed_dom[["lower.random"]]

NASH_paed_dom_tab$lower <- exp(NASH_paed_dom_tab$lower)

NASH_paed_dom_tab$upper <- NASH_paed_dom[["upper.random"]]

NASH_paed_dom_tab$upper <- exp(NASH_paed_dom_tab$upper)

NASH_paed_dom_tab$k <- NASH_paed_dom[["k"]]

NASH_paed_dom_tab$p_z <- NASH_paed_dom[["pval.random"]]

NASH_paed_dom_tab$I2 <- NASH_paed_dom[["I2"]]

NASH_paed_dom_tab$p_q <- NASH_paed_dom[["pval.Q"]]
```

```
NASH_paed_dom_tab$group <- "Overall"
NASH_paed_dom_tab$model <- "Dominant"
NASH_paed_dom_tab$outcome <- "NASH"

NASH_paed_sumtab <- rbind(NASH_paed_rec_tab, NASH_paed_add_tab, NASH_paed_dom_tab)



**Presence of any fibrosis in adults

AnyFib_adult <- read_excel("AnyFib_adult.xlsx")
AnyFib_adult_add <- metagen(add_logOR, add_seOR, studlab = Author, method.tau = "DL", sm = "OR", data = AnyFib_adult)
AnyFib_adult_add_ethnic <- update(AnyFib_adult_add, byvar = Ethnicity, bylab = "Ethnicity")
AnyFib_adult_rec <- metagen(rec_logOR, rec_seOR, studlab = Author, method.tau = "DL", sm = "OR", data = AnyFib_adult)
AnyFib_adult_rec_ethnic <- update(AnyFib_adult_rec, byvar = Ethnicity, bylab = "Ethnicity")
AnyFib_adult_dom <- metagen(dom_logOR, dom_seOR, studlab = Author, method.tau = "DL", sm = "OR", data = AnyFib_adult)
AnyFib_adult_dom_ethnic <- update(AnyFib_adult_dom, byvar = Ethnicity, bylab = "Ethnicity")
```

** recessive - overall

```
AnyFib_adult_rec_tab <- data.frame(AnyFib_adult_rec_ethnic[["TE.random"]])

row.names(AnyFib_adult_rec_tab) <- "Overall"

AnyFib_adult_rec_tab$OR <- AnyFib_adult_rec_tab$AnyFib_adult_rec_ethnic...TE.random...

AnyFib_adult_rec_tab$OR <- exp(AnyFib_adult_rec_tab$OR)

AnyFib_adult_rec_tab <- AnyFib_adult_rec_tab[-c(1)]

AnyFib_adult_rec_tab$lower <- AnyFib_adult_rec_ethnic[["lower.random"]]

AnyFib_adult_rec_tab$lower <- exp(AnyFib_adult_rec_tab$lower)

AnyFib_adult_rec_tab$upper <- AnyFib_adult_rec_ethnic[["upper.random"]]

AnyFib_adult_rec_tab$upper <- exp(AnyFib_adult_rec_tab$upper)

AnyFib_adult_rec_tab$k <- AnyFib_adult_rec_ethnic[["k"]]

AnyFib_adult_rec_tab$p_z <- AnyFib_adult_rec_ethnic[["pval.random"]]

AnyFib_adult_rec_tab$I2 <- AnyFib_adult_rec_ethnic[["I2"]]

AnyFib_adult_rec_tab$p_q <- AnyFib_adult_rec_ethnic[["pval.Q"]]

AnyFib_adult_rec_tab$group <- "Overall"

AnyFib_adult_rec_tab$model <- "Recessive"

AnyFib_adult_rec_tab$outcome <- "AnyFib"
```

** recessive - subgroups

```
AnyFib_adult_rec_tab2 <- data.frame(AnyFib_adult_rec_ethnic[["TE.random.w"]])

row.names(AnyFib_adult_rec_tab2) <- AnyFib_adult_rec_ethnic[["bylevs"]]

AnyFib_adult_rec_tab2$OR <- AnyFib_adult_rec_tab2$AnyFib_adult_rec_ethnic...TE.random.w...

AnyFib_adult_rec_tab2$OR <- exp(AnyFib_adult_rec_tab2$OR)

AnyFib_adult_rec_tab2 <- AnyFib_adult_rec_tab2[-c(1)]

AnyFib_adult_rec_tab2$lower <- AnyFib_adult_rec_ethnic[["lower.random.w"]]

AnyFib_adult_rec_tab2$lower <- exp(AnyFib_adult_rec_tab2$lower)

AnyFib_adult_rec_tab2$upper <- AnyFib_adult_rec_ethnic[["upper.random.w"]]

AnyFib_adult_rec_tab2$upper <- exp(AnyFib_adult_rec_tab2$upper)

AnyFib_adult_rec_tab2$k <- AnyFib_adult_rec_ethnic[["k.w"]]

AnyFib_adult_rec_tab2$p_z <- AnyFib_adult_rec_ethnic[["pval.random.w"]]

AnyFib_adult_rec_tab2$I2 <- AnyFib_adult_rec_ethnic[["I2.w"]]

AnyFib_adult_rec_tab2$p_q <- AnyFib_adult_rec_ethnic[["pval.Q.w"]]

AnyFib_adult_rec_tab2$group <- row.names(AnyFib_adult_rec_tab2)

AnyFib_adult_rec_tab2$model <- "Recessive"

AnyFib_adult_rec_tab2$outcome <- "AnyFib"
```

```
** additive - overall

AnyFib_adult_add_tab <- data.frame(AnyFib_adult_add_ethnic[["TE.random"]])

row.names(AnyFib_adult_add_tab) <- "Overall"

AnyFib_adult_add_tab$OR <- AnyFib_adult_add_tab$AnyFib_adult_add_ethnic...TE.random...

AnyFib_adult_add_tab$OR <- exp(AnyFib_adult_add_tab$OR)

AnyFib_adult_add_tab <- AnyFib_adult_add_tab[-c(1)]

AnyFib_adult_add_tab$lower <- AnyFib_adult_add_ethnic[["lower.random"]]

AnyFib_adult_add_tab$lower <- exp(AnyFib_adult_add_tab$lower)

AnyFib_adult_add_tab$upper <- AnyFib_adult_add_ethnic[["upper.random"]]

AnyFib_adult_add_tab$upper <- exp(AnyFib_adult_add_tab$upper)

AnyFib_adult_add_tab$k <- AnyFib_adult_add_ethnic[["k"]]

AnyFib_adult_add_tab$p_z <- AnyFib_adult_add_ethnic[["pval.random"]]

AnyFib_adult_add_tab$I2 <- AnyFib_adult_add_ethnic[["I2"]]

AnyFib_adult_add_tab$p_q <- AnyFib_adult_add_ethnic[["pval.Q"]]

AnyFib_adult_add_tab$group <- "Overall"

AnyFib_adult_add_tab$model <- "Additive"

AnyFib_adult_add_tab$outcome <- "AnyFib"
```

```
** additive - subgroups

AnyFib_adult_add_tab2 <- data.frame(AnyFib_adult_add_ethnic[["TE.random.w"]])

row.names(AnyFib_adult_add_tab2) <- AnyFib_adult_add_ethnic[["bylevs"]]

AnyFib_adult_add_tab2$OR <- AnyFib_adult_add_tab2$AnyFib_adult_add_ethnic...TE.random.w...

AnyFib_adult_add_tab2$OR <- exp(AnyFib_adult_add_tab2$OR)

AnyFib_adult_add_tab2 <- AnyFib_adult_add_tab2[-c(1)]

AnyFib_adult_add_tab2$lower <- AnyFib_adult_add_ethnic[["lower.random.w"]]

AnyFib_adult_add_tab2$lower <- exp(AnyFib_adult_add_tab2$lower)

AnyFib_adult_add_tab2$upper <- AnyFib_adult_add_ethnic[["upper.random.w"]]

AnyFib_adult_add_tab2$upper <- exp(AnyFib_adult_add_tab2$upper)

AnyFib_adult_add_tab2$k <- AnyFib_adult_add_ethnic[["k.w"]]

AnyFib_adult_add_tab2$p_z <- AnyFib_adult_add_ethnic[["pval.random.w"]]

AnyFib_adult_add_tab2$I2 <- AnyFib_adult_add_ethnic[["I2.w"]]

AnyFib_adult_add_tab2$p_q <- AnyFib_adult_add_ethnic[["pval.Q.w"]]

AnyFib_adult_add_tab2$group <- row.names(AnyFib_adult_add_tab2)

AnyFib_adult_add_tab2$model <- "Additive"

AnyFib_adult_add_tab2$outcome <- "AnyFib"
```

** dominant - overall

```
AnyFib_adult_dom_tab <- data.frame(AnyFib_adult_dom_ethnic[["TE.random"]])

row.names(AnyFib_adult_dom_tab) <- "Overall"

AnyFib_adult_dom_tab$OR <- AnyFib_adult_dom_tab$AnyFib_adult_dom_ethnic...TE.random...

AnyFib_adult_dom_tab$OR <- exp(AnyFib_adult_dom_tab$OR)

AnyFib_adult_dom_tab <- AnyFib_adult_dom_tab[-c(1)]

AnyFib_adult_dom_tab$lower <- AnyFib_adult_dom_ethnic[["lower.random"]]

AnyFib_adult_dom_tab$lower <- exp(AnyFib_adult_dom_tab$lower)

AnyFib_adult_dom_tab$upper <- AnyFib_adult_dom_ethnic[["upper.random"]]

AnyFib_adult_dom_tab$upper <- exp(AnyFib_adult_dom_tab$upper)

AnyFib_adult_dom_tab$k <- AnyFib_adult_dom_ethnic[["k"]]

AnyFib_adult_dom_tab$p_z <- AnyFib_adult_dom_ethnic[["pval.random"]]

AnyFib_adult_dom_tab$I2 <- AnyFib_adult_dom_ethnic[["I2"]]

AnyFib_adult_dom_tab$p_q <- AnyFib_adult_dom_ethnic[["pval.Q"]]

AnyFib_adult_dom_tab$group <- "Overall"

AnyFib_adult_dom_tab$model <- "Dominant"

AnyFib_adult_dom_tab$outcome <- "AnyFib"
```

** dominant - subgroups

```
AnyFib_adult_dom_tab2 <- data.frame(AnyFib_adult_dom_ethnic[["TE.random.w"]])

row.names(AnyFib_adult_dom_tab2) <- AnyFib_adult_dom_ethnic[["bylevs"]]

AnyFib_adult_dom_tab2$OR <- AnyFib_adult_dom_tab2$AnyFib_adult_dom_ethnic...TE.random.w...

AnyFib_adult_dom_tab2$OR <- exp(AnyFib_adult_dom_tab2$OR)

AnyFib_adult_dom_tab2 <- AnyFib_adult_dom_tab2[-c(1)]

AnyFib_adult_dom_tab2$lower <- AnyFib_adult_dom_ethnic[["lower.random.w"]]

AnyFib_adult_dom_tab2$lower <- exp(AnyFib_adult_dom_tab2$lower)

AnyFib_adult_dom_tab2$upper <- AnyFib_adult_dom_ethnic[["upper.random.w"]]

AnyFib_adult_dom_tab2$upper <- exp(AnyFib_adult_dom_tab2$upper)

AnyFib_adult_dom_tab2$k <- AnyFib_adult_dom_ethnic[["k.w"]]

AnyFib_adult_dom_tab2$p_z <- AnyFib_adult_dom_ethnic[["pval.random.w"]]

AnyFib_adult_dom_tab2$I2 <- AnyFib_adult_dom_ethnic[["I2.w"]]

AnyFib_adult_dom_tab2$p_q <- AnyFib_adult_dom_ethnic[["pval.Q.w"]]

AnyFib_adult_dom_tab2$group <- row.names(AnyFib_adult_dom_tab2)

AnyFib_adult_dom_tab2$model <- "Dominant"

AnyFib_adult_dom_tab2$outcome <- "AnyFib"
```

```
AnyFib_sumtab <- rbind(AnyFib_adult_rec_tab, AnyFib_adult_rec_tab2, AnyFib_adult_add_tab, AnyFib_adult_add_tab2, AnyFib_adult_dom_tab, AnyFib_adult_dom_tab2)

pdf(file="AnyFib_adult_rec_ethnic.pdf",width=10,height=8)
AnyFib_adult_rec_ethnic_forest <- forest(AnyFib_adult_rec_ethnic, comb.fixed=FALSE, col.random="red", col.diamond="red", xlab="Odds of fibrosis", digits.se=2, text.random = "Recessive model - overall effect", leftcols = c("studlab", "TT cases", "CT+CC cases", "TT controls", "CT+CC controls"), leftlabs = c("Study", "TT", "CT+CC", "TT", "CT+CC"), lab.e = "F1-4", lab.c = "F0", digits.addcols.left=0, just.studlab="left", just.addcols.left="right", lab.e.attach.to.col="CT+CC cases", lab.c.attach.to.col="CT+CC controls", sortvar=rec_logOR, digits.pval=3, test.overall.random=TRUE, test.effect.subgroup.random=TRUE)
dev.off()
```

**Presence of any fibrosis in children

```
AnyFib_paed <- read_excel("AnyFib_paed.xlsx")
AnyFib_paed_add <- metagen(add_logOR, add_seOR, studlab = Author, method.tau = "DL", sm = "OR", data = AnyFib_paed)
```

```
AnyFib_paed_rec <- metagen(rec_logOR, rec_seOR, studlab = Author, method.tau = "DL", sm = "OR", data = AnyFib_paed)

AnyFib_paed_dom <- metagen(dom_logOR, dom_seOR, studlab = Author, method.tau = "DL", sm = "OR", data = AnyFib_paed)


** recessive - overall

AnyFib_paed_rec_tab <- data.frame(AnyFib_paed_rec[["TE.random"]])

row.names(AnyFib_paed_rec_tab) <- "Overall"

AnyFib_paed_rec_tab$OR <- AnyFib_paed_rec_tab$AnyFib_paed_rec...TE.random...

AnyFib_paed_rec_tab$OR <- exp(AnyFib_paed_rec_tab$OR)

AnyFib_paed_rec_tab <- AnyFib_paed_rec_tab[-c(1)]

AnyFib_paed_rec_tab$lower <- AnyFib_paed_rec[["lower.random"]]

AnyFib_paed_rec_tab$lower <- exp(AnyFib_paed_rec_tab$lower)

AnyFib_paed_rec_tab$upper <- AnyFib_paed_rec[["upper.random"]]

AnyFib_paed_rec_tab$upper <- exp(AnyFib_paed_rec_tab$upper)

AnyFib_paed_rec_tab$k <- AnyFib_paed_rec[["k"]]

AnyFib_paed_rec_tab$p_z <- AnyFib_paed_rec[["pval.random"]]

AnyFib_paed_rec_tab$I2 <- AnyFib_paed_rec[["I2"]]

AnyFib_paed_rec_tab$p_q <- AnyFib_paed_rec[["pval.Q"]]

AnyFib_paed_rec_tab$group <- "Overall"
```

```
AnyFib_paed_rec_tab$model <- "Recessive"

AnyFib_paed_rec_tab$outcome <- "AnyFib"


** additive - overall

AnyFib_paed_add_tab <- data.frame(AnyFib_paed_add[["TE.random"]])

row.names(AnyFib_paed_add_tab) <- "Overall"

AnyFib_paed_add_tab$OR <- AnyFib_paed_add_tab$AnyFib_paed_add...TE.random...

AnyFib_paed_add_tab$OR <- exp(AnyFib_paed_add_tab$OR)

AnyFib_paed_add_tab <- AnyFib_paed_add_tab[-c(1)]

AnyFib_paed_add_tab$lower <- AnyFib_paed_add[["lower.random"]]

AnyFib_paed_add_tab$lower <- exp(AnyFib_paed_add_tab$lower)

AnyFib_paed_add_tab$upper <- AnyFib_paed_add[["upper.random"]]

AnyFib_paed_add_tab$upper <- exp(AnyFib_paed_add_tab$upper)

AnyFib_paed_add_tab$k <- AnyFib_paed_add[["k"]]

AnyFib_paed_add_tab$p_z <- AnyFib_paed_add[["pval.random"]]

AnyFib_paed_add_tab$I2 <- AnyFib_paed_add[["I2"]]

AnyFib_paed_add_tab$p_q <- AnyFib_paed_add[["pval.Q"]]

AnyFib_paed_add_tab$group <- "Overall"
```

```
AnyFib_paed_add_tab$model <- "Additive"

AnyFib_paed_add_tab$outcome <- "AnyFib"


** dominant - overall

AnyFib_paed_dom_tab <- data.frame(AnyFib_paed_dom[["TE.random"]])

row.names(AnyFib_paed_dom_tab) <- "Overall"

AnyFib_paed_dom_tab$OR <- AnyFib_paed_dom_tab$AnyFib_paed_dom...TE.random...

AnyFib_paed_dom_tab$OR <- exp(AnyFib_paed_dom_tab$OR)

AnyFib_paed_dom_tab <- AnyFib_paed_dom_tab[-c(1)]

AnyFib_paed_dom_tab$lower <- AnyFib_paed_dom[["lower.random"]]

AnyFib_paed_dom_tab$lower <- exp(AnyFib_paed_dom_tab$lower)

AnyFib_paed_dom_tab$upper <- AnyFib_paed_dom[["upper.random"]]

AnyFib_paed_dom_tab$upper <- exp(AnyFib_paed_dom_tab$upper)

AnyFib_paed_dom_tab$k <- AnyFib_paed_dom[["k"]]

AnyFib_paed_dom_tab$p_z <- AnyFib_paed_dom[["pval.random"]]

AnyFib_paed_dom_tab$I2 <- AnyFib_paed_dom[["I2"]]

AnyFib_paed_dom_tab$p_q <- AnyFib_paed_dom[["pval.Q"]]

AnyFib_paed_dom_tab$group <- "Overall"
```

```
AnyFib_paed_dom_tab$model <- "Dominant"

AnyFib_paed_dom_tab$outcome <- "AnyFib"


AnyFib_paed_sumtab <- rbind(AnyFib_paed_rec_tab, AnyFib_paed_add_tab, AnyFib_paed_dom_tab)




**Presence of advanced fibrosis in adults


FibAdv_adult <- read_excel("FibAdv_adult.xlsx")

FibAdv_adult_add <- metagen(add_logOR, add_seOR, studlab = Author, method.tau = "DL", sm = "OR", data = FibAdv_adult)

FibAdv_adult_add_ethnic <- update(FibAdv_adult_add, byvar = Ethnicity, bylab = "Ethnicity")

FibAdv_adult_rec <- metagen(rec_logOR, rec_seOR, studlab = Author, method.tau = "DL", sm = "OR", data = FibAdv_adult)

FibAdv_adult_rec_ethnic <- update(FibAdv_adult_rec, byvar = Ethnicity, bylab = "Ethnicity")

FibAdv_adult_dom <- metagen(dom_logOR, dom_seOR, studlab = Author, method.tau = "DL", sm = "OR", data = FibAdv_adult)

FibAdv_adult_dom_ethnic <- update(FibAdv_adult_dom, byvar = Ethnicity, bylab = "Ethnicity")


** recessive - overall

FibAdv_adult_rec_tab <- data.frame(FibAdv_adult_rec_ethnic[["TE.random"]])
```

```
row.names(FibAdv_adult_rec_tab) <- "Overall"

FibAdv_adult_rec_tab$OR <- FibAdv_adult_rec_tab$FibAdv_adult_rec_ethnic...TE.random...

FibAdv_adult_rec_tab$OR <- exp(FibAdv_adult_rec_tab$OR)

FibAdv_adult_rec_tab <- FibAdv_adult_rec_tab[-c(1)]

FibAdv_adult_rec_tab$lower <- FibAdv_adult_rec_ethnic[["lower.random"]]

FibAdv_adult_rec_tab$lower <- exp(FibAdv_adult_rec_tab$lower)

FibAdv_adult_rec_tab$upper <- FibAdv_adult_rec_ethnic[["upper.random"]]

FibAdv_adult_rec_tab$upper <- exp(FibAdv_adult_rec_tab$upper)

FibAdv_adult_rec_tab$k <- FibAdv_adult_rec_ethnic[["k"]]

FibAdv_adult_rec_tab$p_z <- FibAdv_adult_rec_ethnic[["pval.random"]]

FibAdv_adult_rec_tab$I2 <- FibAdv_adult_rec_ethnic[["I2"]]

FibAdv_adult_rec_tab$p_q <- FibAdv_adult_rec_ethnic[["pval.Q"]]

FibAdv_adult_rec_tab$group <- "Overall"

FibAdv_adult_rec_tab$model <- "Recessive"

FibAdv_adult_rec_tab$outcome <- "FibAdv"


** recessive - subgroups

FibAdv_adult_rec_tab2 <- data.frame(FibAdv_adult_rec_ethnic[["TE.random.w"]])
```

```
row.names(FibAdv_adult_rec_tab2) <- FibAdv_adult_rec_ethnic[["bylevs"]]

FibAdv_adult_rec_tab2$OR <- FibAdv_adult_rec_tab2$FibAdv_adult_rec_ethnic...TE.random.w...

FibAdv_adult_rec_tab2$OR <- exp(FibAdv_adult_rec_tab2$OR)

FibAdv_adult_rec_tab2 <- FibAdv_adult_rec_tab2[-c(1)]

FibAdv_adult_rec_tab2$lower <- FibAdv_adult_rec_ethnic[["lower.random.w"]]

FibAdv_adult_rec_tab2$lower <- exp(FibAdv_adult_rec_tab2$lower)

FibAdv_adult_rec_tab2$upper <- FibAdv_adult_rec_ethnic[["upper.random.w"]]

FibAdv_adult_rec_tab2$upper <- exp(FibAdv_adult_rec_tab2$upper)

FibAdv_adult_rec_tab2$k <- FibAdv_adult_rec_ethnic[["k.w"]]

FibAdv_adult_rec_tab2$p_z <- FibAdv_adult_rec_ethnic[["pval.random.w"]]

FibAdv_adult_rec_tab2$I2 <- FibAdv_adult_rec_ethnic[["I2.w"]]

FibAdv_adult_rec_tab2$p_q <- FibAdv_adult_rec_ethnic[["pval.Q.w"]]

FibAdv_adult_rec_tab2$group <- row.names(FibAdv_adult_rec_tab2)

FibAdv_adult_rec_tab2$model <- "Recessive"

FibAdv_adult_rec_tab2$outcome <- "FibAdv"


** additive - overall

FibAdv_adult_add_tab <- data.frame(FibAdv_adult_add_ethnic[["TE.random"]])
```

```
row.names(FibAdv_adult_add_tab) <- "Overall"

FibAdv_adult_add_tab$OR <- FibAdv_adult_add_tab$FibAdv_adult_add_ethnic...TE.random...

FibAdv_adult_add_tab$OR <- exp(FibAdv_adult_add_tab$OR)

FibAdv_adult_add_tab <- FibAdv_adult_add_tab[-c(1)]

FibAdv_adult_add_tab$lower <- FibAdv_adult_add_ethnic[["lower.random"]]

FibAdv_adult_add_tab$lower <- exp(FibAdv_adult_add_tab$lower)

FibAdv_adult_add_tab$upper <- FibAdv_adult_add_ethnic[["upper.random"]]

FibAdv_adult_add_tab$upper <- exp(FibAdv_adult_add_tab$upper)

FibAdv_adult_add_tab$k <- FibAdv_adult_add_ethnic[["k"]]

FibAdv_adult_add_tab$p_z <- FibAdv_adult_add_ethnic[["pval.random"]]

FibAdv_adult_add_tab$I2 <- FibAdv_adult_add_ethnic[["I2"]]

FibAdv_adult_add_tab$p_q <- FibAdv_adult_add_ethnic[["pval.Q"]]

FibAdv_adult_add_tab$group <- "Overall"

FibAdv_adult_add_tab$model <- "Additive"

FibAdv_adult_add_tab$outcome <- "FibAdv"


** additive - subgroups

FibAdv_adult_add_tab2 <- data.frame(FibAdv_adult_add_ethnic[["TE.random.w"]])
```

```
row.names(FibAdv_adult_add_tab2) <- FibAdv_adult_add_ethnic[["bylevs"]]

FibAdv_adult_add_tab2$OR <- FibAdv_adult_add_tab2$FibAdv_adult_add_ethnic...TE.random.w...

FibAdv_adult_add_tab2$OR <- exp(FibAdv_adult_add_tab2$OR)

FibAdv_adult_add_tab2 <- FibAdv_adult_add_tab2[-c(1)]

FibAdv_adult_add_tab2$lower <- FibAdv_adult_add_ethnic[["lower.random.w"]]

FibAdv_adult_add_tab2$lower <- exp(FibAdv_adult_add_tab2$lower)

FibAdv_adult_add_tab2$upper <- FibAdv_adult_add_ethnic[["upper.random.w"]]

FibAdv_adult_add_tab2$upper <- exp(FibAdv_adult_add_tab2$upper)

FibAdv_adult_add_tab2$k <- FibAdv_adult_add_ethnic[["k.w"]]

FibAdv_adult_add_tab2$p_z <- FibAdv_adult_add_ethnic[["pval.random.w"]]

FibAdv_adult_add_tab2$I2 <- FibAdv_adult_add_ethnic[["I2.w"]]

FibAdv_adult_add_tab2$p_q <- FibAdv_adult_add_ethnic[["pval.Q.w"]]

FibAdv_adult_add_tab2$group <- row.names(FibAdv_adult_add_tab2)

FibAdv_adult_add_tab2$model <- "Additive"

FibAdv_adult_add_tab2$outcome <- "FibAdv"
```

** dominant - overall

```
FibAdv_adult_dom_tab <- data.frame(FibAdv_adult_dom_ethnic[["TE.random"]])

row.names(FibAdv_adult_dom_tab) <- "Overall"

FibAdv_adult_dom_tab$OR <- FibAdv_adult_dom_tab$FibAdv_adult_dom_ethnic...TE.random...

FibAdv_adult_dom_tab$OR <- exp(FibAdv_adult_dom_tab$OR)

FibAdv_adult_dom_tab <- FibAdv_adult_dom_tab[-c(1)]

FibAdv_adult_dom_tab$lower <- FibAdv_adult_dom_ethnic[["lower.random"]]

FibAdv_adult_dom_tab$lower <- exp(FibAdv_adult_dom_tab$lower)

FibAdv_adult_dom_tab$upper <- FibAdv_adult_dom_ethnic[["upper.random"]]

FibAdv_adult_dom_tab$upper <- exp(FibAdv_adult_dom_tab$upper)

FibAdv_adult_dom_tab$k <- FibAdv_adult_dom_ethnic[["k"]]

FibAdv_adult_dom_tab$p_z <- FibAdv_adult_dom_ethnic[["pval.random"]]

FibAdv_adult_dom_tab$I2 <- FibAdv_adult_dom_ethnic[["I2"]]

FibAdv_adult_dom_tab$p_q <- FibAdv_adult_dom_ethnic[["pval.Q"]]

FibAdv_adult_dom_tab$group <- "Overall"

FibAdv_adult_dom_tab$model <- "Dominant"

FibAdv_adult_dom_tab$outcome <- "FibAdv"


** dominant - subgroups
```

```
FibAdv_adult_dom_tab2 <- data.frame(FibAdv_adult_dom_ethnic[["TE.random.w"]])

row.names(FibAdv_adult_dom_tab2) <- FibAdv_adult_dom_ethnic[["bylevs"]]

FibAdv_adult_dom_tab2$OR <- FibAdv_adult_dom_tab2$FibAdv_adult_dom_ethnic...TE.random.w...

FibAdv_adult_dom_tab2$OR <- exp(FibAdv_adult_dom_tab2$OR)

FibAdv_adult_dom_tab2 <- FibAdv_adult_dom_tab2[-c(1)]

FibAdv_adult_dom_tab2$lower <- FibAdv_adult_dom_ethnic[["lower.random.w"]]

FibAdv_adult_dom_tab2$lower <- exp(FibAdv_adult_dom_tab2$lower)

FibAdv_adult_dom_tab2$upper <- FibAdv_adult_dom_ethnic[["upper.random.w"]]

FibAdv_adult_dom_tab2$upper <- exp(FibAdv_adult_dom_tab2$upper)

FibAdv_adult_dom_tab2$k <- FibAdv_adult_dom_ethnic[["k.w"]]

FibAdv_adult_dom_tab2$p_z <- FibAdv_adult_dom_ethnic[["pval.random.w"]]

FibAdv_adult_dom_tab2$I2 <- FibAdv_adult_dom_ethnic[["I2.w"]]

FibAdv_adult_dom_tab2$p_q <- FibAdv_adult_dom_ethnic[["pval.Q.w"]]

FibAdv_adult_dom_tab2$group <- row.names(FibAdv_adult_dom_tab2)

FibAdv_adult_dom_tab2$model <- "Dominant"

FibAdv_adult_dom_tab2$outcome <- "FibAdv"
```

```
FibAdv_sumtab <- rbind(FibAdv_adult_rec_tab, FibAdv_adult_rec_tab2, FibAdv_adult_add_tab, FibAdv_adult_add_tab2, FibAdv_adult_dom_tab, FibAdv_adult_dom_tab2)

pdf(file="FibAdv_adult_rec_ethnic.pdf",width=10,height=8)
FibAdv_adult_rec_ethnic_forest <- forest(FibAdv_adult_rec_ethnic, comb.fixed=FALSE, col.random="red", col.diamond="red", xlab="Odds of advanced fibrosis", digits.se=2, text.random = "Recessive model - overall effect", leftcols = c("studlab", "TT cases", "CT+CC cases", "TT controls", "CT+CC controls"), leftlabs = c("Study", "TT", "CT+CC", "TT", "CT+CC"), lab.e = "F3-4", lab.c = "F0-2", digits.addcols.left=0, just.studlab="left", just.addcols.left="right", lab.e.attach.to.col="CT+CC cases", lab.c.attach.to.col="CT+CC controls", sortvar=rec_logOR, digits.pval=3, test.overall.random=TRUE, test.effect.subgroup.random=TRUE)
dev.off()
```

**Presence of advanced fibrosis in children

```
FibAdv_paed <- read_excel("FibAdv_paed.xlsx")
FibAdv_paed_add <- metagen(add_logOR, add_seOR, studlab = Author, method.tau = "DL", sm = "OR", data = FibAdv_paed)
FibAdv_paed_rec <- metagen(rec_logOR, rec_seOR, studlab = Author, method.tau = "DL", sm = "OR", data = FibAdv_paed)
```

```
FibAdv_paed_dom <- metagen(dom_logOR, dom_seOR, studlab = Author, method.tau = "DL", sm = "OR", data = FibAdv_paed)
```

** recessive - overall

```
FibAdv_paed_rec_tab <- data.frame(FibAdv_paed_rec[["TE.random"]])

row.names(FibAdv_paed_rec_tab) <- "Overall"

FibAdv_paed_rec_tab$OR <- FibAdv_paed_rec_tab$FibAdv_paed_rec...TE.random...

FibAdv_paed_rec_tab$OR <- exp(FibAdv_paed_rec_tab$OR)

FibAdv_paed_rec_tab <- FibAdv_paed_rec_tab[-c(1)]

FibAdv_paed_rec_tab$lower <- FibAdv_paed_rec[["lower.random"]]

FibAdv_paed_rec_tab$lower <- exp(FibAdv_paed_rec_tab$lower)

FibAdv_paed_rec_tab$upper <- FibAdv_paed_rec[["upper.random"]]

FibAdv_paed_rec_tab$upper <- exp(FibAdv_paed_rec_tab$upper)

FibAdv_paed_rec_tab$k <- FibAdv_paed_rec[["k"]]

FibAdv_paed_rec_tab$p_z <- FibAdv_paed_rec[["pval.random"]]

FibAdv_paed_rec_tab$I2 <- FibAdv_paed_rec[["I2"]]

FibAdv_paed_rec_tab$p_q <- FibAdv_paed_rec[["pval.Q"]]

FibAdv_paed_rec_tab$group <- "Overall"

FibAdv_paed_rec_tab$model <- "Recessive"
```

```
FibAdv_paed_rec_tab$outcome <- "FibAdv"


** additive - overall

FibAdv_paed_add_tab <- data.frame(FibAdv_paed_add[["TE.random"]])

row.names(FibAdv_paed_add_tab) <- "Overall"

FibAdv_paed_add_tab$OR <- FibAdv_paed_add_tab$FibAdv_paed_add...TE.random...

FibAdv_paed_add_tab$OR <- exp(FibAdv_paed_add_tab$OR)

FibAdv_paed_add_tab <- FibAdv_paed_add_tab[-c(1)]

FibAdv_paed_add_tab$lower <- FibAdv_paed_add[["lower.random"]]

FibAdv_paed_add_tab$lower <- exp(FibAdv_paed_add_tab$lower)

FibAdv_paed_add_tab$upper <- FibAdv_paed_add[["upper.random"]]

FibAdv_paed_add_tab$upper <- exp(FibAdv_paed_add_tab$upper)

FibAdv_paed_add_tab$k <- FibAdv_paed_add[["k"]]

FibAdv_paed_add_tab$p_z <- FibAdv_paed_add[["pval.random"]]

FibAdv_paed_add_tab$I2 <- FibAdv_paed_add[["I2"]]

FibAdv_paed_add_tab$p_q <- FibAdv_paed_add[["pval.Q"]]

FibAdv_paed_add_tab$group <- "Overall"

FibAdv_paed_add_tab$model <- "Additive"
```

```
FibAdv_paed_add_tab$outcome <- "FibAdv"


** dominant - overall

FibAdv_paed_dom_tab <- data.frame(FibAdv_paed_dom[["TE.random"]])

row.names(FibAdv_paed_dom_tab) <- "Overall"

FibAdv_paed_dom_tab$OR <- FibAdv_paed_dom_tab$FibAdv_paed_dom...TE.random...

FibAdv_paed_dom_tab$OR <- exp(FibAdv_paed_dom_tab$OR)

FibAdv_paed_dom_tab <- FibAdv_paed_dom_tab[-c(1)]

FibAdv_paed_dom_tab$lower <- FibAdv_paed_dom[["lower.random"]]

FibAdv_paed_dom_tab$lower <- exp(FibAdv_paed_dom_tab$lower)

FibAdv_paed_dom_tab$upper <- FibAdv_paed_dom[["upper.random"]]

FibAdv_paed_dom_tab$upper <- exp(FibAdv_paed_dom_tab$upper)

FibAdv_paed_dom_tab$k <- FibAdv_paed_dom[["k"]]

FibAdv_paed_dom_tab$p_z <- FibAdv_paed_dom[["pval.random"]]

FibAdv_paed_dom_tab$I2 <- FibAdv_paed_dom[["I2"]]

FibAdv_paed_dom_tab$p_q <- FibAdv_paed_dom[["pval.Q"]]

FibAdv_paed_dom_tab$group <- "Overall"

FibAdv_paed_dom_tab$model <- "Dominant"
```

```
FibAdv_paed_dom_tab$outcome <- "FibAdv"

FibAdv_paed_sumtab <- rbind(FibAdv_paed_rec_tab, FibAdv_paed_add_tab, FibAdv_paed_dom_tab)

**Presence of HCC in adults

HCC_adult <- read_excel("HCC_adult.xlsx")
HCC_adult_add <- metagen(add_logOR, add_seOR, studlab = Author, method.tau = "DL", sm = "OR", data = HCC_adult)
HCC_adult_rec <- metagen(rec_logOR, rec_seOR, studlab = Author, method.tau = "DL", sm = "OR", data = HCC_adult)
HCC_adult_dom <- metagen(dom_logOR, dom_seOR, studlab = Author, method.tau = "DL", sm = "OR", data = HCC_adult)

** recessive - overall
HCC_adult_rec_tab <- data.frame(HCC_adult_rec[["TE.random"]])
row.names(HCC_adult_rec_tab) <- "Overall"
HCC_adult_rec_tab$OR <- HCC_adult_rec_tab$HCC_adult_rec...TE.random...
HCC_adult_rec_tab$OR <- exp(HCC_adult_rec_tab$OR)
```

```
HCC_adult_rec_tab <- HCC_adult_rec_tab[-c(1)]

HCC_adult_rec_tab$lower <- HCC_adult_rec[["lower.random"]]

HCC_adult_rec_tab$lower <- exp(HCC_adult_rec_tab$lower)

HCC_adult_rec_tab$upper <- HCC_adult_rec[["upper.random"]]

HCC_adult_rec_tab$upper <- exp(HCC_adult_rec_tab$upper)

HCC_adult_rec_tab$k <- HCC_adult_rec[["k"]]

HCC_adult_rec_tab$p_z <- HCC_adult_rec[["pval.random"]]

HCC_adult_rec_tab$I2 <- HCC_adult_rec[["I2"]]

HCC_adult_rec_tab$p_q <- HCC_adult_rec[["pval.Q"]]

HCC_adult_rec_tab$group <- "Overall"

HCC_adult_rec_tab$model <- "Recessive"

HCC_adult_rec_tab$outcome <- "HCC"


** additive - overall

HCC_adult_add_tab <- data.frame(HCC_adult_add[["TE.random"]])

row.names(HCC_adult_add_tab) <- "Overall"

HCC_adult_add_tab$OR <- HCC_adult_add_tab$HCC_adult_add...TE.random...

HCC_adult_add_tab$OR <- exp(HCC_adult_add_tab$OR)
```

```
HCC_adult_add_tab <- HCC_adult_add_tab[-c(1)]

HCC_adult_add_tab$lower <- HCC_adult_add[["lower.random"]]

HCC_adult_add_tab$lower <- exp(HCC_adult_add_tab$lower)

HCC_adult_add_tab$upper <- HCC_adult_add[["upper.random"]]

HCC_adult_add_tab$upper <- exp(HCC_adult_add_tab$upper)

HCC_adult_add_tab$k <- HCC_adult_add[["k"]]

HCC_adult_add_tab$p_z <- HCC_adult_add[["pval.random"]]

HCC_adult_add_tab$I2 <- HCC_adult_add[["I2"]]

HCC_adult_add_tab$p_q <- HCC_adult_add[["pval.Q"]]

HCC_adult_add_tab$group <- "Overall"

HCC_adult_add_tab$model <- "Additive"

HCC_adult_add_tab$outcome <- "HCC"


** dominant - overall

HCC_adult_dom_tab <- data.frame(HCC_adult_dom[["TE.random"]])

row.names(HCC_adult_dom_tab) <- "Overall"

HCC_adult_dom_tab$OR <- HCC_adult_dom_tab$HCC_adult_dom...TE.random...

HCC_adult_dom_tab$OR <- exp(HCC_adult_dom_tab$OR)
```

```
HCC_adult_dom_tab <- HCC_adult_dom_tab[-c(1)]

HCC_adult_dom_tab$lower <- HCC_adult_dom[["lower.random"]]

HCC_adult_dom_tab$lower <- exp(HCC_adult_dom_tab$lower)

HCC_adult_dom_tab$upper <- HCC_adult_dom[["upper.random"]]

HCC_adult_dom_tab$upper <- exp(HCC_adult_dom_tab$upper)

HCC_adult_dom_tab$k <- HCC_adult_dom[["k"]]

HCC_adult_dom_tab$p_z <- HCC_adult_dom[["pval.random"]]

HCC_adult_dom_tab$I2 <- HCC_adult_dom[["I2"]]

HCC_adult_dom_tab$p_q <- HCC_adult_dom[["pval.Q"]]

HCC_adult_dom_tab$group <- "Overall"

HCC_adult_dom_tab$model <- "Dominant"

HCC_adult_dom_tab$outcome <- "HCC"


HCC_sumtab <- rbind(HCC_adult_rec_tab, HCC_adult_add_tab, HCC_adult_dom_tab)


pdf(file="HCC_adult_rec.pdf",width=10,height=8)

HCC_adult_rec_forest <- forest(HCC_adult_rec, comb.fixed=FALSE, col.random="red", col.diamond="red", xlab="Odds of NAFLD-
HCC", digits.se=2, text.random = "Recessive model - overall effect", leftcols = c("studlab", "TT cases", "CT+CC cases", "TT
```

controls", "CT+CC controls"), leftlabs = c("Study", "TT", "CT+CC", "TT", "CT+CC"), lab.e = "HCC", lab.c = "No HCC", digits.addcols.left=0, just.studlab="left", just.addcols.left="right", lab.e.attach.to.col="CT+CC cases", lab.c.attach.to.col="CT+CC controls", sortvar=rec_logOR, digits.pval=3, test.overall.random=TRUE, test.effect.subgroup.random=TRUE)

dev.off()

**overall summary table for adult & paed histology

Histol_adult_sumtab <- rbind(NAFLD_Dx_sumtab, Steat_sumtab, NASH_sumtab, AnyFib_sumtab, FibAdv_sumtab, HCC_sumtab)

write.table(Histol_adult_sumtab, file="Histol_adult_sumtab.csv",sep=",")

Histol_paed_sumtab <- rbind(NAFLD_Dx_paed_sumtab, Steat_paed_sumtab, NASH_paed_sumtab, AnyFib_paed_sumtab, FibAdv_paed_sumtab)

write.table(Histol_paed_sumtab, file="Histol_paed_sumtab.csv",sep=",")

** liver fat analyses

HFF_adult <- read_excel("HFF_adult.xlsx")

```
HFF_adult$HFF_CCCT_num <- HFF_adult$HFF_CC_num+HFF_adult$HFF_CT_num
HFF_adult$HFF_CCCT_mean <-
((HFF_adult$HFF_CC_mean*HFF_adult$HFF_CC_num)+(HFF_adult$HFF_CT_mean*HFF_adult$HFF_CT_num))/HFF_adult$HF
F_CCCT_num
HFF_adult$HFF_CCCT_SD <-
((HFF_adult$HFF_CC_SD*HFF_adult$HFF_CC_num)+(HFF_adult$HFF_CT_SD*HFF_adult$HFF_CT_num))/HFF_adult$HFF_CC
CT_num
HFF_adult$HFF_CTTT_num <- HFF_adult$HFF_TT_num+HFF_adult$HFF_CT_num
HFF_adult$HFF_CTTT_mean <-
((HFF_adult$HFF_TT_mean*HFF_adult$HFF_TT_num)+(HFF_adult$HFF_CT_mean*HFF_adult$HFF_CT_num))/HFF_adult$HFF
_CTTT_num
HFF_adult$HFF_CTTT_SD <-
((HFF_adult$HFF_TT_SD*HFF_adult$HFF_TT_num)+(HFF_adult$HFF_CT_SD*HFF_adult$HFF_CT_num))/HFF_adult$HFF_CT
TT_num

HFF_adult_add <- read_excel("HFF_adult_add.xlsx")
HFF_adult_add_reg <- metagen(Beta, SE, data = HFF_adult_add, studlab = HFF_adult_add$Author, sm = "ZCOR", method.tau =
"DL")
```

```
HFF_adult_add_reg_ethnic <- update(HFF_adult_add_reg, byvar = Ethnicity, bylab = "Ethnicity")

pdf(file="HFF_adult_add_reg_ethnic.pdf",width=10,height=6)

forest(HFF_adult_add_reg_ethnic, comb.fixed=FALSE, col.random="red", col.diamond="red", xlab="Beta (95% CI) per T-allele",
digits.se=2, digits.pval=2, scientific.pval=TRUE, sortvar=TE, test.overall.random=TRUE, overall=TRUE, digits=2, print.zval=TRUE,
colgap.studlab="2.5cm", print.tau2=FALSE, rightlabs = c("Beta", "95% CI", "Weight"), smlab="Hepatic fat on CT/MRI", leftcols =
c("studlab", "num"), leftlabs = c("Study", "Total"), print.subgroup.labels = TRUE, xlim=c(-0.2,0.2),
test.effect.subgroup.random=TRUE)

dev.off()


HFF_adult_add_reg_mod <- update(HFF_adult_add_reg, byvar = Method, bylab = "Modality")

pdf(file="HFF_adult_add_reg_mod.pdf",width=10,height=8)

forest(HFF_adult_add_reg_mod, comb.fixed=FALSE, col.random="red", col.diamond="red", xlab="Beta (95% CI) per T-allele",
digits.se=2, digits.pval=2, scientific.pval= TRUE, sortvar=TE, test.overall.random=TRUE, overall=TRUE, digits=2, print.zval=TRUE,
colgap.studlab="2.5cm", print.tau2=FALSE, rightlabs = c("Beta", "95% CI", "Weight"), smlab="Hepatic fat on CT/MRI", leftcols =
c("studlab", "num"), leftlabs = c("Study", "Total"), print.subgroup.labels = TRUE, xlim=c(-0.2,0.2),
test.effect.subgroup.random=TRUE)

dev.off()
```

```
HFF_adult_rec_reg <- metacont(HFF_TT_num, HFF_TT_mean, HFF_TT_SD, HFF_CCCT_num, HFF_CCCT_mean,
HFF_CCCT_SD, data = HFF_adult, studlab = paste(Author), comb.fixed = FALSE, comb.random = TRUE, method.tau = "DL", hakn
= TRUE, prediction = FALSE, sm = "MD")
HFF_adult_rec_reg_ethnic <- update(HFF_adult_rec_reg, byvar = Ethnicity, bylab = "Ethnicity")

HFF_adult_dom_reg <- metacont(HFF_CTTT_num, HFF_CTTT_mean, HFF_CTTT_SD, HFF_CC_num, HFF_CC_mean,
HFF_CC_SD, data = HFF_adult, studlab = paste(Author), comb.fixed = FALSE, comb.random = TRUE, method.tau = "DL", hakn =
TRUE, prediction = FALSE, sm = "MD")
HFF_adult_dom_reg_ethnic <- update(HFF_adult_dom_reg, byvar = Ethnicity, bylab = "Ethnicity")

HFF_adult_add_tab <- data.frame(HFF_adult_add_reg_ethnic[["TE.random"]])
row.names(HFF_adult_add_tab) <- "Overall"
HFF_adult_add_tab$MD <- HFF_adult_add_tab$HFF_adult_add_reg_ethnic...TE.random...
HFF_adult_add_tab <- HFF_adult_add_tab[-c(1)]
HFF_adult_add_tab$lower <- HFF_adult_add_reg_ethnic[["lower.random"]]
HFF_adult_add_tab$upper <- HFF_adult_add_reg_ethnic[["upper.random"]]
HFF_adult_add_tab$k <- HFF_adult_add_reg_ethnic[["k"]]
HFF_adult_add_tab$p_z <- HFF_adult_add_reg_ethnic[["pval.random"]]
```

```
HFF_adult_add_tab$I2 <- HFF_adult_add_reg_ethnic[["I2"]]

HFF_adult_add_tab$p_q <- HFF_adult_add_reg_ethnic[["pval.Q"]]

HFF_adult_add_tab$group <- "Overall"

HFF_adult_add_tab$model <- "Additive"

HFF_adult_add_tab$outcome <- "HFF"


HFF_adult_add_tab2 <- data.frame(HFF_adult_add_reg_ethnic[["TE.random.w"]])

row.names(HFF_adult_add_tab2) <- HFF_adult_add_reg_ethnic[["bylevs"]]

HFF_adult_add_tab2$MD <- HFF_adult_add_tab2$HFF_adult_add_reg_ethnic...TE.random.w...

HFF_adult_add_tab2 <- HFF_adult_add_tab2[-c(1)]

HFF_adult_add_tab2$lower <- HFF_adult_add_reg_ethnic[["lower.random.w"]]

HFF_adult_add_tab2$upper <- HFF_adult_add_reg_ethnic[["upper.random.w"]]

HFF_adult_add_tab2$k <- HFF_adult_add_reg_ethnic[["k.w"]]

HFF_adult_add_tab2$p_z <- HFF_adult_add_reg_ethnic[["pval.random.w"]]

HFF_adult_add_tab2$I2 <- HFF_adult_add_reg_ethnic[["I2.w"]]

HFF_adult_add_tab2$p_q <- HFF_adult_add_reg_ethnic[["pval.Q.w"]]

HFF_adult_add_tab2$group <- row.names(HFF_adult_add_tab2)

HFF_adult_add_tab2$model <- "Additive"
```

```
HFF_adult_add_tab2$outcome <- "HFF"


HFF_adult_dom_tab <- data.frame(HFF_adult_dom_reg_ethnic[["TE.random"]])

row.names(HFF_adult_dom_tab) <- "Overall"

HFF_adult_dom_tab$MD <- HFF_adult_dom_tab$HFF_adult_dom_reg_ethnic...TE.random...

HFF_adult_dom_tab <- HFF_adult_dom_tab[-c(1)]

HFF_adult_dom_tab$lower <- HFF_adult_dom_reg_ethnic[["lower.random"]]

HFF_adult_dom_tab$upper <- HFF_adult_dom_reg_ethnic[["upper.random"]]

HFF_adult_dom_tab$k <- HFF_adult_dom_reg_ethnic[["k"]]

HFF_adult_dom_tab$p_z <- HFF_adult_dom_reg_ethnic[["pval.random"]]

HFF_adult_dom_tab$I2 <- HFF_adult_dom_reg_ethnic[["I2"]]

HFF_adult_dom_tab$p_q <- HFF_adult_dom_reg_ethnic[["pval.Q"]]

HFF_adult_dom_tab$group <- "Overall"

HFF_adult_dom_tab$model <- "Dominant"

HFF_adult_dom_tab$outcome <- "HFF"


HFF_adult_dom_tab2 <- data.frame(HFF_adult_dom_reg_ethnic[["TE.random.w"]])

row.names(HFF_adult_dom_tab2) <- HFF_adult_dom_reg_ethnic[["bylevs"]]
```

```
HFF_adult_dom_tab2$MD <- HFF_adult_dom_tab2$HFF_adult_dom_reg_ethnic...TE.random.w...

HFF_adult_dom_tab2 <- HFF_adult_dom_tab2[-c(1)]

HFF_adult_dom_tab2$lower <- HFF_adult_dom_reg_ethnic[["lower.random.w"]]

HFF_adult_dom_tab2$upper <- HFF_adult_dom_reg_ethnic[["upper.random.w"]]

HFF_adult_dom_tab2$k <- HFF_adult_dom_reg_ethnic[["k.w"]]

HFF_adult_dom_tab2$p_z <- HFF_adult_dom_reg_ethnic[["pval.random.w"]]

HFF_adult_dom_tab2$I2 <- HFF_adult_dom_reg_ethnic[["I2.w"]]

HFF_adult_dom_tab2$p_q <- HFF_adult_dom_reg_ethnic[["pval.Q.w"]]

HFF_adult_dom_tab2$group <- row.names(HFF_adult_dom_tab2)

HFF_adult_dom_tab2$model <- "Dominant"

HFF_adult_dom_tab2$outcome <- "HFF"


HFF_adult_rec_tab <- data.frame(HFF_adult_rec_reg_ethnic[["TE.random"]])

row.names(HFF_adult_rec_tab) <- "Overall"

HFF_adult_rec_tab$MD <- HFF_adult_rec_tab$HFF_adult_rec_reg_ethnic...TE.random...

HFF_adult_rec_tab <- HFF_adult_rec_tab[-c(1)]

HFF_adult_rec_tab$lower <- HFF_adult_rec_reg_ethnic[["lower.random"]]

HFF_adult_rec_tab$upper <- HFF_adult_rec_reg_ethnic[["upper.random"]]
```

```
HFF_adult_rec_tab$k <- HFF_adult_rec_reg_ethnic[["k"]]

HFF_adult_rec_tab$p_z <- HFF_adult_rec_reg_ethnic[["pval.random"]]

HFF_adult_rec_tab$I2 <- HFF_adult_rec_reg_ethnic[["I2"]]

HFF_adult_rec_tab$p_q <- HFF_adult_rec_reg_ethnic[["pval.Q"]]

HFF_adult_rec_tab$group <- "Overall"

HFF_adult_rec_tab$model <- "Recessive"

HFF_adult_rec_tab$outcome <- "HFF"


HFF_adult_rec_tab2 <- data.frame(HFF_adult_rec_reg_ethnic[["TE.random.w"]])

row.names(HFF_adult_rec_tab2) <- HFF_adult_rec_reg_ethnic[["bylevs"]]

HFF_adult_rec_tab2$MD <- HFF_adult_rec_tab2$HFF_adult_rec_reg_ethnic...TE.random.w...

HFF_adult_rec_tab2 <- HFF_adult_rec_tab2[-c(1)]

HFF_adult_rec_tab2$lower <- HFF_adult_rec_reg_ethnic[["lower.random.w"]]

HFF_adult_rec_tab2$upper <- HFF_adult_rec_reg_ethnic[["upper.random.w"]]

HFF_adult_rec_tab2$k <- HFF_adult_rec_reg_ethnic[["k.w"]]

HFF_adult_rec_tab2$p_z <- HFF_adult_rec_reg_ethnic[["pval.random.w"]]

HFF_adult_rec_tab2$I2 <- HFF_adult_rec_reg_ethnic[["I2.w"]]

HFF_adult_rec_tab2$p_q <- HFF_adult_rec_reg_ethnic[["pval.Q.w"]]
```

```
HFF_adult_rec_tab2$group <- row.names(HFF_adult_rec_tab2)

HFF_adult_rec_tab2$model <- "Recessive"

HFF_adult_rec_tab2$outcome <- "HFF"



CAP_adult <- read_excel("CAP_adult.xlsx")


CAP_adult$CAP_CCCT_num <- CAP_adult$CAP_CC_num+CAP_adult$CAP_CT_num

CAP_adult$CAP_CCCT_mean <-
((CAP_adult$CAP_CC_mean*CAP_adult$CAP_CC_num)+(CAP_adult$CAP_CT_mean*CAP_adult$CAP_CT_num))/CAP_adult$
CAP_CCCT_num

CAP_adult$CAP_CCCT_SD <-
((CAP_adult$CAP_CC_SD*CAP_adult$CAP_CC_num)+(CAP_adult$CAP_CT_SD*CAP_adult$CAP_CT_num))/CAP_adult$CAP_
CCCT_num

CAP_adult$CAP_CTTT_num <- CAP_adult$CAP_TT_num+CAP_adult$CAP_CT_num

CAP_adult$CAP_CTTT_mean <-
((CAP_adult$CAP_TT_mean*CAP_adult$CAP_TT_num)+(CAP_adult$CAP_CT_mean*CAP_adult$CAP_CT_num))/CAP_adult$C
AP_CTTT_num
```

```
CAP_adult$CAP_CTTT_SD <-
((CAP_adult$CAP_TT_SD*CAP_adult$CAP_TT_num)+(CAP_adult$CAP_CT_SD*CAP_adult$CAP_CT_num))/CAP_adult$CAP_C
TTT_num

CAP_adult_add <- read_excel("CAP_adult_add.xlsx")
CAP_adult_add_reg <- metagen(Beta, SE, data = CAP_adult_add, studlab = CAP_adult_add$Author, sm = "ZCOR", method.tau =
"DL")
pdf(file="CAP_adult_add_reg.pdf",width=9,height=4)
forest(CAP_adult_add_reg, comb.fixed=FALSE, col.random="red", col.diamond="red", xlab="Beta (95% CI) per T-allele",
digits.se=2, digits.pval=3, scientific.pval=FALSE, sortvar=TE, test.overall.random=TRUE, overall=TRUE, digits=2, print.zval=TRUE,
colgap.studlab="3cm", print.tau2=FALSE, rightlabs = c("Beta", "95% CI", "Weight"), smlab="Steatosis score \n from CAP/US",
leftcols = c("studlab", "num"), leftlabs = c("Study", "Total"), print.subgroup.labels = TRUE, xlim=c(-0.1,0.1),
test.effect.subgroup.random=TRUE)
dev.off()

CAP_adult_rec_reg <- metacont(CAP_TT_num, CAP_TT_mean, CAP_TT_SD, CAP_CCCT_num, CAP_CCCT_mean,
CAP_CCCT_SD, data = CAP_adult, studlab = paste(Author), comb.fixed = FALSE, comb.random = TRUE, method.tau = "DL",
hakn = TRUE, prediction = FALSE, sm = "MD")
```

```
CAP_adult_rec_reg <- update(CAP_adult_rec_reg, byvar = Ethnicity, bylab = "Ethnicity")


CAP_adult_dom_reg <- metacont(CAP_CTTT_num, CAP_CTTT_mean, CAP_CTTT_SD, CAP_CC_num, CAP_CC_mean,
CAP_CC_SD, data = CAP_adult, studlab = paste(Author), comb.fixed = FALSE, comb.random = TRUE, method.tau = "DL", hakn =
TRUE, prediction = FALSE, sm = "MD")
CAP_adult_dom_reg <- update(CAP_adult_dom_reg, byvar = Ethnicity, bylab = "Ethnicity")


CAP_adult_add_tab <- data.frame(CAP_adult_add_reg[["TE.random"]])
row.names(CAP_adult_add_tab) <- "Overall"
CAP_adult_add_tab$MD <- CAP_adult_add_tab$CAP_adult_add_reg...TE.random...
CAP_adult_add_tab <- CAP_adult_add_tab[-c(1)]
CAP_adult_add_tab$lower <- CAP_adult_add_reg[["lower.random"]]
CAP_adult_add_tab$upper <- CAP_adult_add_reg[["upper.random"]]
CAP_adult_add_tab$k <- CAP_adult_add_reg[["k"]]
CAP_adult_add_tab$p_z <- CAP_adult_add_reg[["pval.random"]]
CAP_adult_add_tab$I2 <- CAP_adult_add_reg[["I2"]]
CAP_adult_add_tab$p_q <- CAP_adult_add_reg[["pval.Q"]]
CAP_adult_add_tab$group <- "Overall"
```

```
CAP_adult_add_tab$model <- "Additive"

CAP_adult_add_tab$outcome <- "CAP"


CAP_adult_dom_tab <- data.frame(CAP_adult_dom_reg[["TE.random"]])

row.names(CAP_adult_dom_tab) <- "Overall"

CAP_adult_dom_tab$MD <- CAP_adult_dom_tab$CAP_adult_dom_reg...TE.random...

CAP_adult_dom_tab <- CAP_adult_dom_tab[-c(1)]

CAP_adult_dom_tab$lower <- CAP_adult_dom_reg[["lower.random"]]

CAP_adult_dom_tab$upper <- CAP_adult_dom_reg[["upper.random"]]

CAP_adult_dom_tab$k <- CAP_adult_dom_reg[["k"]]

CAP_adult_dom_tab$p_z <- CAP_adult_dom_reg[["pval.random"]]

CAP_adult_dom_tab$I2 <- CAP_adult_dom_reg[["I2"]]

CAP_adult_dom_tab$p_q <- CAP_adult_dom_reg[["pval.Q"]]

CAP_adult_dom_tab$group <- "Overall"

CAP_adult_dom_tab$model <- "Dominant"

CAP_adult_dom_tab$outcome <- "CAP"


CAP_adult_rec_tab <- data.frame(CAP_adult_rec_reg[["TE.random"]])
```

```
row.names(CAP_adult_rec_tab) <- "Overall"

CAP_adult_rec_tab$MD <- CAP_adult_rec_tab$CAP_adult_rec_reg...TE.random...

CAP_adult_rec_tab <- CAP_adult_rec_tab[-c(1)]

CAP_adult_rec_tab$lower <- CAP_adult_rec_reg[["lower.random"]]

CAP_adult_rec_tab$upper <- CAP_adult_rec_reg[["upper.random"]]

CAP_adult_rec_tab$k <- CAP_adult_rec_reg[["k"]]

CAP_adult_rec_tab$p_z <- CAP_adult_rec_reg[["pval.random"]]

CAP_adult_rec_tab$I2 <- CAP_adult_rec_reg[["I2"]]

CAP_adult_rec_tab$p_q <- CAP_adult_rec_reg[["pval.Q"]]

CAP_adult_rec_tab$group <- "Overall"

CAP_adult_rec_tab$model <- "Recessive"

CAP_adult_rec_tab$outcome <- "CAP"



HFF_adult_sumtab <- rbind(HFF_adult_add_tab, HFF_adult_add_tab2, HFF_adult_dom_tab, HFF_adult_dom_tab2,

HFF_adult_rec_tab, HFF_adult_rec_tab2, CAP_adult_add_tab, CAP_adult_dom_tab, CAP_adult_rec_tab)

write.table(HFF_adult_sumtab, file="HFF_adult_sumtab.csv",sep=",")
```

```
HFF_paed <- read_excel("HFF_paed.xlsx")


HFF_paed$HFF_CCCT_num <- HFF_paed$HFF_CC_num+HFF_paed$HFF_CT_num
HFF_paed$HFF_CCCT_mean <-
((HFF_paed$HFF_CC_mean*HFF_paed$HFF_CC_num)+(HFF_paed$HFF_CT_mean*HFF_paed$HFF_CT_num))/HFF_paed$HF
F_CCCT_num
HFF_paed$HFF_CCCT_SD <-
((HFF_paed$HFF_CC_SD*HFF_paed$HFF_CC_num)+(HFF_paed$HFF_CT_SD*HFF_paed$HFF_CT_num))/HFF_paed$HFF_C
CCT_num
HFF_paed$HFF_CTTT_num <- HFF_paed$HFF_TT_num+HFF_paed$HFF_CT_num
HFF_paed$HFF_CTTT_mean <-
((HFF_paed$HFF_TT_mean*HFF_paed$HFF_TT_num)+(HFF_paed$HFF_CT_mean*HFF_paed$HFF_CT_num))/HFF_paed$HF
F_CTTT_num
HFF_paed$HFF_CTTT_SD <-
((HFF_paed$HFF_TT_SD*HFF_paed$HFF_TT_num)+(HFF_paed$HFF_CT_SD*HFF_paed$HFF_CT_num))/HFF_paed$HFF_CT
TT_num
```

```
HFF_paed_add <- read_excel("HFF_paed_add.xlsx")

HFF_paed_add_reg <- metagen(Beta, SE, data = HFF_paed_add, studlab = HFF_paed_add$Author, sm = "ZCOR", method.tau =
"DL")

HFF_paed_add_reg_ethnic <- update(HFF_paed_add_reg, byvar = Ethnicity, bylab = "Ethnicity")

pdf(file="HFF_paed_add_reg_ethnic.pdf",width=10,height=6)

forest(HFF_paed_add_reg_ethnic, comb.fixed=FALSE, col.random="red", col.diamond="red", xlab="Beta (95% CI) per T-allele",
digits.se=2, digits.pval=3, scientific.pval=FALSE, sortvar=TE, test.overall.random=TRUE, overall=TRUE, digits=2, print.zval=TRUE,
colgap.studlab="2.5cm", print.tau2=FALSE, rightlabs = c("Beta", "95% CI", "Weight"), smlab="Hepatic fat on CT/MRI", leftcols =
c("studlab", "num"), leftlabs = c("Study", "Total"), print.subgroup.labels = TRUE, xlim=c(-0.2,0.2),
test.effect.subgroup.random=TRUE)

dev.off()


HFF_paed_add_reg_mod <- update(HFF_paed_add_reg, byvar = Method, bylab = "Modality")

pdf(file="HFF_paed_add_reg_mod.pdf",width=10,height=6)

forest(HFF_paed_add_reg_mod, comb.fixed=FALSE, col.random="red", col.diamond="red", xlab="Beta (95% CI) per T-allele",
digits.se=2, digits.pval=3, scientific.pval=FALSE, sortvar=TE, test.overall.random=TRUE, overall=TRUE, digits=2, print.zval=TRUE,
colgap.studlab="2.5cm", print.tau2=FALSE, rightlabs = c("Beta", "95% CI", "Weight"), smlab="Hepatic fat on CT/MRI", leftcols =
```

```
c("studlab", "num"), leftlabs = c("Study", "Total"), print.subgroup.labels = TRUE, xlim=c(-0.2,0.2),
test.effect.subgroup.random=TRUE)
dev.off()

HFF_paed_rec_reg <- metacont(HFF_TT_num, HFF_TT_mean, HFF_TT_SD, HFF_CCCT_num, HFF_CCCT_mean,
HFF_CCCT_SD, data = HFF_paed, studlab = paste(Author), comb.fixed = FALSE, comb.random = TRUE, method.tau = "DL",
hakn = TRUE, prediction = FALSE, sm = "MD")
HFF_paed_rec_reg_ethnic <- update(HFF_paed_rec_reg, byvar = Ethnicity, bylab = "Ethnicity")

HFF_paed_dom_reg <- metacont(HFF_CTTT_num, HFF_CTTT_mean, HFF_CTTT_SD, HFF_CC_num, HFF_CC_mean,
HFF_CC_SD, data = HFF_paed, studlab = paste(Author), comb.fixed = FALSE, comb.random = TRUE, method.tau = "DL", hakn =
TRUE, prediction = FALSE, sm = "MD")
HFF_paed_dom_reg_ethnic <- update(HFF_paed_dom_reg, byvar = Ethnicity, bylab = "Ethnicity")

HFF_paed_add_tab <- data.frame(HFF_paed_add_reg_ethnic[["TE.random"]])
row.names(HFF_paed_add_tab) <- "Overall"
HFF_paed_add_tab$MD <- HFF_paed_add_tab$HFF_paed_add_reg_ethnic...TE.random...
HFF_paed_add_tab <- HFF_paed_add_tab[-c(1)]
```

```
HFF_paed_add_tab$lower <- HFF_paed_add_reg_ethnic[["lower.random"]]

HFF_paed_add_tab$upper <- HFF_paed_add_reg_ethnic[["upper.random"]]

HFF_paed_add_tab$k <- HFF_paed_add_reg_ethnic[["k"]]

HFF_paed_add_tab$p_z <- HFF_paed_add_reg_ethnic[["pval.random"]]

HFF_paed_add_tab$I2 <- HFF_paed_add_reg_ethnic[["I2"]]

HFF_paed_add_tab$p_q <- HFF_paed_add_reg_ethnic[["pval.Q"]]

HFF_paed_add_tab$group <- "Overall"

HFF_paed_add_tab$model <- "Additive"

HFF_paed_add_tab$outcome <- "HFF"


HFF_paed_add_tab2 <- data.frame(HFF_paed_add_reg_ethnic[["TE.random.w"]])

row.names(HFF_paed_add_tab2) <- HFF_paed_add_reg_ethnic[["bylevs"]]

HFF_paed_add_tab2$MD <- HFF_paed_add_tab2$HFF_paed_add_reg_ethnic...TE.random.w...

HFF_paed_add_tab2 <- HFF_paed_add_tab2[-c(1)]

HFF_paed_add_tab2$lower <- HFF_paed_add_reg_ethnic[["lower.random.w"]]

HFF_paed_add_tab2$upper <- HFF_paed_add_reg_ethnic[["upper.random.w"]]

HFF_paed_add_tab2$k <- HFF_paed_add_reg_ethnic[["k.w"]]

HFF_paed_add_tab2$p_z <- HFF_paed_add_reg_ethnic[["pval.random.w"]]
```

```
HFF_paed_add_tab2$I2 <- HFF_paed_add_reg_ethnic[["I2.w"]]

HFF_paed_add_tab2$p_q <- HFF_paed_add_reg_ethnic[["pval.Q.w"]]

HFF_paed_add_tab2$group <- row.names(HFF_paed_add_tab2)

HFF_paed_add_tab2$model <- "Additive"

HFF_paed_add_tab2$outcome <- "HFF"


HFF_paed_dom_tab <- data.frame(HFF_paed_dom_reg_ethnic[["TE.random"]])

row.names(HFF_paed_dom_tab) <- "Overall"

HFF_paed_dom_tab$MD <- HFF_paed_dom_tab$HFF_paed_dom_reg_ethnic...TE.random...

HFF_paed_dom_tab <- HFF_paed_dom_tab[-c(1)]

HFF_paed_dom_tab$lower <- HFF_paed_dom_reg_ethnic[["lower.random"]]

HFF_paed_dom_tab$upper <- HFF_paed_dom_reg_ethnic[["upper.random"]]

HFF_paed_dom_tab$k <- HFF_paed_dom_reg_ethnic[["k"]]

HFF_paed_dom_tab$p_z <- HFF_paed_dom_reg_ethnic[["pval.random"]]

HFF_paed_dom_tab$I2 <- HFF_paed_dom_reg_ethnic[["I2"]]

HFF_paed_dom_tab$p_q <- HFF_paed_dom_reg_ethnic[["pval.Q"]]

HFF_paed_dom_tab$group <- "Overall"

HFF_paed_dom_tab$model <- "Dominant"
```

```
HFF_paed_dom_tab$outcome <- "HFF"


HFF_paed_dom_tab2 <- data.frame(HFF_paed_dom_reg_ethnic[["TE.random.w"]])

row.names(HFF_paed_dom_tab2) <- HFF_paed_dom_reg_ethnic[["bylevs"]]

HFF_paed_dom_tab2$MD <- HFF_paed_dom_tab2$HFF_paed_dom_reg_ethnic...TE.random.w...

HFF_paed_dom_tab2 <- HFF_paed_dom_tab2[-c(1)]

HFF_paed_dom_tab2$lower <- HFF_paed_dom_reg_ethnic[["lower.random.w"]]

HFF_paed_dom_tab2$upper <- HFF_paed_dom_reg_ethnic[["upper.random.w"]]

HFF_paed_dom_tab2$k <- HFF_paed_dom_reg_ethnic[["k.w"]]

HFF_paed_dom_tab2$p_z <- HFF_paed_dom_reg_ethnic[["pval.random.w"]]

HFF_paed_dom_tab2$I2 <- HFF_paed_dom_reg_ethnic[["I2.w"]]

HFF_paed_dom_tab2$p_q <- HFF_paed_dom_reg_ethnic[["pval.Q.w"]]

HFF_paed_dom_tab2$group <- row.names(HFF_paed_dom_tab2)

HFF_paed_dom_tab2$model <- "Dominant"

HFF_paed_dom_tab2$outcome <- "HFF"


HFF_paed_rec_tab <- data.frame(HFF_paed_rec_reg_ethnic[["TE.random"]])

row.names(HFF_paed_rec_tab) <- "Overall"
```

```
HFF_paed_rec_tab$MD <- HFF_paed_rec_tab$HFF_paed_rec_reg_ethnic...TE.random...

HFF_paed_rec_tab <- HFF_paed_rec_tab[-c(1)]

HFF_paed_rec_tab$lower <- HFF_paed_rec_reg_ethnic[["lower.random"]]

HFF_paed_rec_tab$upper <- HFF_paed_rec_reg_ethnic[["upper.random"]]

HFF_paed_rec_tab$k <- HFF_paed_rec_reg_ethnic[["k"]]

HFF_paed_rec_tab$p_z <- HFF_paed_rec_reg_ethnic[["pval.random"]]

HFF_paed_rec_tab$I2 <- HFF_paed_rec_reg_ethnic[["I2"]]

HFF_paed_rec_tab$p_q <- HFF_paed_rec_reg_ethnic[["pval.Q"]]

HFF_paed_rec_tab$group <- "Overall"

HFF_paed_rec_tab$model <- "Recessive"

HFF_paed_rec_tab$outcome <- "HFF"


HFF_paed_rec_tab2 <- data.frame(HFF_paed_rec_reg_ethnic[["TE.random.w"]])

row.names(HFF_paed_rec_tab2) <- HFF_paed_rec_reg_ethnic[["bylevs"]]

HFF_paed_rec_tab2$MD <- HFF_paed_rec_tab2$HFF_paed_rec_reg_ethnic...TE.random.w...

HFF_paed_rec_tab2 <- HFF_paed_rec_tab2[-c(1)]

HFF_paed_rec_tab2$lower <- HFF_paed_rec_reg_ethnic[["lower.random.w"]]

HFF_paed_rec_tab2$upper <- HFF_paed_rec_reg_ethnic[["upper.random.w"]]
```

```
HFF_paed_rec_tab2$k <- HFF_paed_rec_reg_ethnic[["k.w"]]

HFF_paed_rec_tab2$p_z <- HFF_paed_rec_reg_ethnic[["pval.random.w"]]

HFF_paed_rec_tab2$I2 <- HFF_paed_rec_reg_ethnic[["I2.w"]]

HFF_paed_rec_tab2$p_q <- HFF_paed_rec_reg_ethnic[["pval.Q.w"]]

HFF_paed_rec_tab2$group <- row.names(HFF_paed_rec_tab2)

HFF_paed_rec_tab2$model <- "Recessive"

HFF_paed_rec_tab2$outcome <- "HFF"



HFF_paed_sumtab <- rbind(HFF_paed_add_tab, HFF_paed_add_tab2, HFF_paed_dom_tab, HFF_paed_dom_tab2,
HFF_paed_rec_tab, HFF_paed_rec_tab2)
write.table(HFF_paed_sumtab, file="HFF_paed_sumtab.csv",sep=",")



**bias analysis of NAFLD_Dx_adult


NAFLD_Dx_adult_rec_eggers <- eggers.test(x = NAFLD_Dx_adult_rec)
```

```
sink("NAFLD_Dx_adult_rec_eggers.txt")

print(NAFLD_Dx_adult_rec_eggers)

sink()


NAFLD_Dx_adult_rec_trimfill <- trimfill(NAFLD_Dx_adult_rec)

sink("NAFLD_Dx_adult_rec_trimfill.txt")

print(NAFLD_Dx_adult_rec_trimfill)

sink()


pdf(file="NAFLD_Dx_adult_rec_trimfill.pdf")

funnel(NAFLD_Dx_adult_rec_trimfill, xlab="Odds of NAFLD", contour = c(0.95,0.975,0.99),

col.contour=c("darkblue","blue","lightblue")) + legend(0.01, 0.0, c("p < 0.05", "p < 0.025", "p < 0.01"), bty = "n",

fill=c("darkblue","blue","lightblue"))

dev.off()


pdf(file="NAFLD_Dx_adult_rec_baujat.pdf")

baujat(NAFLD_Dx_adult_rec)

dev.off()
```

```
**** perform meta-regressions

NAFLD_Dx_female <- metareg(NAFLD_Dx_adult_rec, FemalePer)
NAFLD_Dx_age <- metareg(NAFLD_Dx_adult_rec, Age)
NAFLD_Dx_t2dm <- metareg(NAFLD_Dx_adult_rec, T2DMPer)
NAFLD_Dx_bmi <- metareg(NAFLD_Dx_adult_rec, BMI)
NAFLD_Dx_pnp <- metareg(NAFLD_Dx_adult_rec, PNPLA3)

NAFLD_Dx_female_tab <- data.frame(NAFLD_Dx_female[["beta"]])
NAFLD_Dx_female_tab$se <- NAFLD_Dx_female[["se"]]
NAFLD_Dx_female_tab$pval <- NAFLD_Dx_female[["pval"]]
NAFLD_Dx_female_tab$beta <- NAFLD_Dx_female[["beta"]]
NAFLD_Dx_female_tab <- NAFLD_Dx_female_tab[-c(1),]
NAFLD_Dx_female_tab <- NAFLD_Dx_female_tab[-c(1)]
NAFLD_Dx_female_tab$k <- NAFLD_Dx_female[["k"]]
NAFLD_Dx_female_tab$r2 <- NAFLD_Dx_female[["R2"]]
```

```
NAFLD_Dx_female_tab$var <- "Female"


NAFLD_Dx_age_tab <- data.frame(NAFLD_Dx_age[["beta"]])

NAFLD_Dx_age_tab$se <- NAFLD_Dx_age[["se"]]

NAFLD_Dx_age_tab$pval <- NAFLD_Dx_age[["pval"]]

NAFLD_Dx_age_tab$beta <- NAFLD_Dx_age[["beta"]]

NAFLD_Dx_age_tab <- NAFLD_Dx_age_tab[-c(1),]

NAFLD_Dx_age_tab <- NAFLD_Dx_age_tab[-c(1)]

NAFLD_Dx_age_tab$k <- NAFLD_Dx_age[["k"]]

NAFLD_Dx_age_tab$r2 <- NAFLD_Dx_age[["R2"]]

NAFLD_Dx_age_tab$var <- "Age"


NAFLD_Dx_bmi_tab <- data.frame(NAFLD_Dx_bmi[["beta"]])

NAFLD_Dx_bmi_tab$se <- NAFLD_Dx_bmi[["se"]]

NAFLD_Dx_bmi_tab$pval <- NAFLD_Dx_bmi[["pval"]]

NAFLD_Dx_bmi_tab$beta <- NAFLD_Dx_bmi[["beta"]]

NAFLD_Dx_bmi_tab <- NAFLD_Dx_bmi_tab[-c(1),]

NAFLD_Dx_bmi_tab <- NAFLD_Dx_bmi_tab[-c(1)]
```

```
NAFLD_Dx_bmi_tab$k <- NAFLD_Dx_bmi[["k"]]

NAFLD_Dx_bmi_tab$r2 <- NAFLD_Dx_bmi[["R2"]]

NAFLD_Dx_bmi_tab$var <- "BMI"


NAFLD_Dx_t2dm_tab <- data.frame(NAFLD_Dx_t2dm[["beta"]])

NAFLD_Dx_t2dm_tab$se <- NAFLD_Dx_t2dm[["se"]]

NAFLD_Dx_t2dm_tab$pval <- NAFLD_Dx_t2dm[["pval"]]

NAFLD_Dx_t2dm_tab$beta <- NAFLD_Dx_t2dm[["beta"]]

NAFLD_Dx_t2dm_tab <- NAFLD_Dx_t2dm_tab[-c(1),]

NAFLD_Dx_t2dm_tab <- NAFLD_Dx_t2dm_tab[-c(1)]

NAFLD_Dx_t2dm_tab$k <- NAFLD_Dx_t2dm[["k"]]

NAFLD_Dx_t2dm_tab$r2 <- NAFLD_Dx_t2dm[["R2"]]

NAFLD_Dx_t2dm_tab$var <- "T2DM"


NAFLD_Dx_pnp_tab <- data.frame(NAFLD_Dx_pnp[["beta"]])

NAFLD_Dx_pnp_tab$se <- NAFLD_Dx_pnp[["se"]]

NAFLD_Dx_pnp_tab$pval <- NAFLD_Dx_pnp[["pval"]]

NAFLD_Dx_pnp_tab$beta <- NAFLD_Dx_pnp[["beta"]]
```

```
NAFLD_Dx_pnp_tab <- NAFLD_Dx_pnp_tab[-c(1),]

NAFLD_Dx_pnp_tab <- NAFLD_Dx_pnp_tab[-c(1)]

NAFLD_Dx_pnp_tab$k <- NAFLD_Dx_pnp[["k"]]

NAFLD_Dx_pnp_tab$r2 <- NAFLD_Dx_pnp[["R2"]]

NAFLD_Dx_pnp_tab$var <- "PNPLA3"


NAFLD_Dx_metareg <- rbind(NAFLD_Dx_female_tab, NAFLD_Dx_age_tab, NAFLD_Dx_bmi_tab, NAFLD_Dx_t2dm_tab,
NAFLD_Dx_pnp_tab)

NAFLD_Dx_metareg$Outcome <- "NAFLD_Dx"


Steat_female <- metareg(Steat_adult_rec, FemalePer)

Steat_age <- metareg(Steat_adult_rec, Age)

Steat_t2dm <- metareg(Steat_adult_rec, T2DMPer)

Steat_bmi <- metareg(Steat_adult_rec, BMI)

Steat_pnp <- metareg(Steat_adult_rec, PNPLA3)


Steat_female_tab <- data.frame(Steat_female[["beta"]])

Steat_female_tab$se <- Steat_female[["se"]]
```

```
Steat_female_tab$pval <- Steat_female[["pval"]]

Steat_female_tab$beta <- Steat_female[["beta"]]

Steat_female_tab <- Steat_female_tab[-c(1),]

Steat_female_tab <- Steat_female_tab[-c(1)]

Steat_female_tab$k <- Steat_female[["k"]]

Steat_female_tab$r2 <- Steat_female[["R2"]]

Steat_female_tab$var <- "Female"


Steat_age_tab <- data.frame(Steat_age[["beta"]])

Steat_age_tab$se <- Steat_age[["se"]]

Steat_age_tab$pval <- Steat_age[["pval"]]

Steat_age_tab$beta <- Steat_age[["beta"]]

Steat_age_tab <- Steat_age_tab[-c(1),]

Steat_age_tab <- Steat_age_tab[-c(1)]

Steat_age_tab$k <- Steat_age[["k"]]

Steat_age_tab$r2 <- Steat_age[["R2"]]

Steat_age_tab$var <- "Age"
```

```
Steat_bmi_tab <- data.frame(Steat_bmi[["beta"]])

Steat_bmi_tab$se <- Steat_bmi[["se"]]

Steat_bmi_tab$pval <- Steat_bmi[["pval"]]

Steat_bmi_tab$beta <- Steat_bmi[["beta"]]

Steat_bmi_tab <- Steat_bmi_tab[-c(1),]

Steat_bmi_tab <- Steat_bmi_tab[-c(1)]

Steat_bmi_tab$k <- Steat_bmi[["k"]]

Steat_bmi_tab$r2 <- Steat_bmi[["R2"]]

Steat_bmi_tab$var <- "BMI"


Steat_t2dm_tab <- data.frame(Steat_t2dm[["beta"]])

Steat_t2dm_tab$se <- Steat_t2dm[["se"]]

Steat_t2dm_tab$pval <- Steat_t2dm[["pval"]]

Steat_t2dm_tab$beta <- Steat_t2dm[["beta"]]

Steat_t2dm_tab <- Steat_t2dm_tab[-c(1),]

Steat_t2dm_tab <- Steat_t2dm_tab[-c(1)]

Steat_t2dm_tab$k <- Steat_t2dm[["k"]]

Steat_t2dm_tab$r2 <- Steat_t2dm[["R2"]]
```

```
Steat_t2dm_tab$var <- "T2DM"

Steat_pnp_tab <- data.frame(Steat_pnp[["beta"]])
Steat_pnp_tab$se <- Steat_pnp[["se"]]
Steat_pnp_tab$pval <- Steat_pnp[["pval"]]
Steat_pnp_tab$beta <- Steat_pnp[["beta"]]
Steat_pnp_tab <- Steat_pnp_tab[-c(1),]
Steat_pnp_tab <- Steat_pnp_tab[-c(1)]
Steat_pnp_tab$k <- Steat_pnp[["k"]]
Steat_pnp_tab$r2 <- Steat_pnp[["R2"]]
Steat_pnp_tab$var <- "PNPLA3"

Steat_metareg <- rbind(Steat_female_tab, Steat_age_tab, Steat_bmi_tab, Steat_t2dm_tab, Steat_pnp_tab)
Steat_metareg$Outcome <- "Steat"

NASH_female <- metareg(NASH_adult_rec, FemalePer)
NASH_age <- metareg(NASH_adult_rec, Age)
NASH_t2dm <- metareg(NASH_adult_rec, T2DMPer)
```

```
NASH_bmi <- metareg(NASH_adult_rec, BMI)

NASH_pnp <- metareg(NASH_adult_rec, PNPLA3)


NASH_female_tab <- data.frame(NASH_female[["beta"]])

NASH_female_tab$se <- NASH_female[["se"]]

NASH_female_tab$pval <- NASH_female[["pval"]]

NASH_female_tab$beta <- NASH_female[["beta"]]

NASH_female_tab <- NASH_female_tab[-c(1),]

NASH_female_tab <- NASH_female_tab[-c(1)]

NASH_female_tab$k <- NASH_female[["k"]]

NASH_female_tab$r2 <- NASH_female[["R2"]]

NASH_female_tab$var <- "Female"


NASH_age_tab <- data.frame(NASH_age[["beta"]])

NASH_age_tab$se <- NASH_age[["se"]]

NASH_age_tab$pval <- NASH_age[["pval"]]

NASH_age_tab$beta <- NASH_age[["beta"]]

NASH_age_tab <- NASH_age_tab[-c(1),]
```

```
NASH_age_tab <- NASH_age_tab[-c(1)]

NASH_age_tab$k <- NASH_age[["k"]]

NASH_age_tab$r2 <- NASH_age[["R2"]]

NASH_age_tab$var <- "Age"


NASH_bmi_tab <- data.frame(NASH_bmi[["beta"]])

NASH_bmi_tab$se <- NASH_bmi[["se"]]

NASH_bmi_tab$pval <- NASH_bmi[["pval"]]

NASH_bmi_tab$beta <- NASH_bmi[["beta"]]

NASH_bmi_tab <- NASH_bmi_tab[-c(1),]

NASH_bmi_tab <- NASH_bmi_tab[-c(1)]

NASH_bmi_tab$k <- NASH_bmi[["k"]]

NASH_bmi_tab$r2 <- NASH_bmi[["R2"]]

NASH_bmi_tab$var <- "BMI"


NASH_t2dm_tab <- data.frame(NASH_t2dm[["beta"]])

NASH_t2dm_tab$se <- NASH_t2dm[["se"]]

NASH_t2dm_tab$pval <- NASH_t2dm[["pval"]]
```

```
NASH_t2dm_tab$beta <- NASH_t2dm[["beta"]]

NASH_t2dm_tab <- NASH_t2dm_tab[-c(1),]

NASH_t2dm_tab <- NASH_t2dm_tab[-c(1)]

NASH_t2dm_tab$k <- NASH_t2dm[["k"]]

NASH_t2dm_tab$r2 <- NASH_t2dm[["R2"]]

NASH_t2dm_tab$var <- "T2DM"


NASH_pnp_tab <- data.frame(NASH_pnp[["beta"]])

NASH_pnp_tab$se <- NASH_pnp[["se"]]

NASH_pnp_tab$pval <- NASH_pnp[["pval"]]

NASH_pnp_tab$beta <- NASH_pnp[["beta"]]

NASH_pnp_tab <- NASH_pnp_tab[-c(1),]

NASH_pnp_tab <- NASH_pnp_tab[-c(1)]

NASH_pnp_tab$k <- NASH_pnp[["k"]]

NASH_pnp_tab$r2 <- NASH_pnp[["R2"]]

NASH_pnp_tab$var <- "PNPLA3"


NASH_metareg <- rbind(NASH_female_tab, NASH_age_tab, NASH_bmi_tab, NASH_t2dm_tab, NASH_pnp_tab)
```

```
NASH_metareg$Outcome <- "NASH"


AnyFib_female <- metareg(AnyFib_adult_rec, FemalePer)

AnyFib_age <- metareg(AnyFib_adult_rec, Age)

AnyFib_t2dm <- metareg(AnyFib_adult_rec, T2DMPer)

AnyFib_bmi <- metareg(AnyFib_adult_rec, BMI)

AnyFib_pnp <- metareg(AnyFib_adult_rec, PNPLA3)


AnyFib_female_tab <- data.frame(AnyFib_female[["beta"]])

AnyFib_female_tab$se <- AnyFib_female[["se"]]

AnyFib_female_tab$pval <- AnyFib_female[["pval"]]

AnyFib_female_tab$beta <- AnyFib_female[["beta"]]

AnyFib_female_tab <- AnyFib_female_tab[-c(1),]

AnyFib_female_tab <- AnyFib_female_tab[-c(1)]

AnyFib_female_tab$k <- AnyFib_female[["k"]]

AnyFib_female_tab$r2 <- AnyFib_female[["R2"]]

AnyFib_female_tab$var <- "Female"
```

```
AnyFib_age_tab <- data.frame(AnyFib_age[["beta"]])

AnyFib_age_tab$se <- AnyFib_age[["se"]]

AnyFib_age_tab$pval <- AnyFib_age[["pval"]]

AnyFib_age_tab$beta <- AnyFib_age[["beta"]]

AnyFib_age_tab <- AnyFib_age_tab[-c(1),]

AnyFib_age_tab <- AnyFib_age_tab[-c(1)]

AnyFib_age_tab$k <- AnyFib_age[["k"]]

AnyFib_age_tab$r2 <- AnyFib_age[["R2"]]

AnyFib_age_tab$var <- "Age"


AnyFib_bmi_tab <- data.frame(AnyFib_bmi[["beta"]])

AnyFib_bmi_tab$se <- AnyFib_bmi[["se"]]

AnyFib_bmi_tab$pval <- AnyFib_bmi[["pval"]]

AnyFib_bmi_tab$beta <- AnyFib_bmi[["beta"]]

AnyFib_bmi_tab <- AnyFib_bmi_tab[-c(1),]

AnyFib_bmi_tab <- AnyFib_bmi_tab[-c(1)]

AnyFib_bmi_tab$k <- AnyFib_bmi[["k"]]

AnyFib_bmi_tab$r2 <- AnyFib_bmi[["R2"]]
```

```
AnyFib_bmi_tab$var <- "BMI"


AnyFib_t2dm_tab <- data.frame(AnyFib_t2dm[["beta"]])

AnyFib_t2dm_tab$se <- AnyFib_t2dm[["se"]]

AnyFib_t2dm_tab$pval <- AnyFib_t2dm[["pval"]]

AnyFib_t2dm_tab$beta <- AnyFib_t2dm[["beta"]]

AnyFib_t2dm_tab <- AnyFib_t2dm_tab[-c(1),]

AnyFib_t2dm_tab <- AnyFib_t2dm_tab[-c(1)]

AnyFib_t2dm_tab$k <- AnyFib_t2dm[["k"]]

AnyFib_t2dm_tab$r2 <- AnyFib_t2dm[["R2"]]

AnyFib_t2dm_tab$var <- "T2DM"


AnyFib_pnp_tab <- data.frame(AnyFib_pnp[["beta"]])

AnyFib_pnp_tab$se <- AnyFib_pnp[["se"]]

AnyFib_pnp_tab$pval <- AnyFib_pnp[["pval"]]

AnyFib_pnp_tab$beta <- AnyFib_pnp[["beta"]]

AnyFib_pnp_tab <- AnyFib_pnp_tab[-c(1),]

AnyFib_pnp_tab <- AnyFib_pnp_tab[-c(1)]
```

```
AnyFib_pnp_tab$k <- AnyFib_pnp[["k"]]

AnyFib_pnp_tab$r2 <- AnyFib_pnp[["R2"]]

AnyFib_pnp_tab$var <- "PNPLA3"


AnyFib_metareg <- rbind(AnyFib_female_tab, AnyFib_age_tab, AnyFib_bmi_tab, AnyFib_t2dm_tab, AnyFib_pnp_tab)

AnyFib_metareg$Outcome <- "AnyFib"


FibAdv_female <- metareg(FibAdv_adult_rec, FemalePer)

FibAdv_age <- metareg(FibAdv_adult_rec, Age)

FibAdv_t2dm <- metareg(FibAdv_adult_rec, T2DMPer)

FibAdv_bmi <- metareg(FibAdv_adult_rec, BMI)

FibAdv_pnp <- metareg(FibAdv_adult_rec, PNPLA3)


FibAdv_female_tab <- data.frame(FibAdv_female[["beta"]])

FibAdv_female_tab$se <- FibAdv_female[["se"]]

FibAdv_female_tab$pval <- FibAdv_female[["pval"]]

FibAdv_female_tab$beta <- FibAdv_female[["beta"]]

FibAdv_female_tab <- FibAdv_female_tab[-c(1),]
```

```
FibAdv_female_tab <- FibAdv_female_tab[-c(1)]

FibAdv_female_tab$k <- FibAdv_female[["k"]]

FibAdv_female_tab$r2 <- FibAdv_female[["R2"]]

FibAdv_female_tab$var <- "Female"


FibAdv_age_tab <- data.frame(FibAdv_age[["beta"]])

FibAdv_age_tab$se <- FibAdv_age[["se"]]

FibAdv_age_tab$pval <- FibAdv_age[["pval"]]

FibAdv_age_tab$beta <- FibAdv_age[["beta"]]

FibAdv_age_tab <- FibAdv_age_tab[-c(1),]

FibAdv_age_tab <- FibAdv_age_tab[-c(1)]

FibAdv_age_tab$k <- FibAdv_age[["k"]]

FibAdv_age_tab$r2 <- FibAdv_age[["R2"]]

FibAdv_age_tab$var <- "Age"


FibAdv_bmi_tab <- data.frame(FibAdv_bmi[["beta"]])

FibAdv_bmi_tab$se <- FibAdv_bmi[["se"]]

FibAdv_bmi_tab$pval <- FibAdv_bmi[["pval"]]
```

```
FibAdv_bmi_tab$beta <- FibAdv_bmi[["beta"]]

FibAdv_bmi_tab <- FibAdv_bmi_tab[-c(1),]

FibAdv_bmi_tab <- FibAdv_bmi_tab[-c(1)]

FibAdv_bmi_tab$k <- FibAdv_bmi[["k"]]

FibAdv_bmi_tab$r2 <- FibAdv_bmi[["R2"]]

FibAdv_bmi_tab$var <- "BMI"


FibAdv_t2dm_tab <- data.frame(FibAdv_t2dm[["beta"]])

FibAdv_t2dm_tab$se <- FibAdv_t2dm[["se"]]

FibAdv_t2dm_tab$pval <- FibAdv_t2dm[["pval"]]

FibAdv_t2dm_tab$beta <- FibAdv_t2dm[["beta"]]

FibAdv_t2dm_tab <- FibAdv_t2dm_tab[-c(1),]

FibAdv_t2dm_tab <- FibAdv_t2dm_tab[-c(1)]

FibAdv_t2dm_tab$k <- FibAdv_t2dm[["k"]]

FibAdv_t2dm_tab$r2 <- FibAdv_t2dm[["R2"]]

FibAdv_t2dm_tab$var <- "T2DM"


FibAdv_pnp_tab <- data.frame(FibAdv_pnp[["beta"]])
```

```
FibAdv_pnp_tab$se <- FibAdv_pnp[["se"]]

FibAdv_pnp_tab$pval <- FibAdv_pnp[["pval"]]

FibAdv_pnp_tab$beta <- FibAdv_pnp[["beta"]]

FibAdv_pnp_tab <- FibAdv_pnp_tab[-c(1),]

FibAdv_pnp_tab <- FibAdv_pnp_tab[-c(1)]

FibAdv_pnp_tab$k <- FibAdv_pnp[["k"]]

FibAdv_pnp_tab$r2 <- FibAdv_pnp[["R2"]]

FibAdv_pnp_tab$var <- "PNPLA3"


FibAdv_metareg <- rbind(FibAdv_female_tab, FibAdv_age_tab, FibAdv_bmi_tab, FibAdv_t2dm_tab, FibAdv_pnp_tab)

FibAdv_metareg$Outcome <- "FibAdv"



HCC_female <- metareg(HCC_adult_rec, FemalePer)

HCC_age <- metareg(HCC_adult_rec, Age)

HCC_t2dm <- metareg(HCC_adult_rec, T2DMPer)

HCC_cirrh <- metareg(HCC_adult_rec, Cirrhosis)
```

```
HCC_female_tab <- data.frame(HCC_female[["beta"]])

HCC_female_tab$se <- HCC_female[["se"]]

HCC_female_tab$pval <- HCC_female[["pval"]]

HCC_female_tab$beta <- HCC_female[["beta"]]

HCC_female_tab <- HCC_female_tab[-c(1),]

HCC_female_tab <- HCC_female_tab[-c(1)]

HCC_female_tab$k <- HCC_female[["k"]]

HCC_female_tab$r2 <- HCC_female[["R2"]]

HCC_female_tab$var <- "Female"


HCC_age_tab <- data.frame(HCC_age[["beta"]])

HCC_age_tab$se <- HCC_age[["se"]]

HCC_age_tab$pval <- HCC_age[["pval"]]

HCC_age_tab$beta <- HCC_age[["beta"]]

HCC_age_tab <- HCC_age_tab[-c(1),]

HCC_age_tab <- HCC_age_tab[-c(1)]

HCC_age_tab$k <- HCC_age[["k"]]

HCC_age_tab$r2 <- HCC_age[["R2"]]
```

```
HCC_age_tab$var <- "Age"


HCC_t2dm_tab <- data.frame(HCC_t2dm[["beta"]])

HCC_t2dm_tab$se <- HCC_t2dm[["se"]]

HCC_t2dm_tab$pval <- HCC_t2dm[["pval"]]

HCC_t2dm_tab$beta <- HCC_t2dm[["beta"]]

HCC_t2dm_tab <- HCC_t2dm_tab[-c(1),]

HCC_t2dm_tab <- HCC_t2dm_tab[-c(1)]

HCC_t2dm_tab$k <- HCC_t2dm[["k"]]

HCC_t2dm_tab$r2 <- HCC_t2dm[["R2"]]

HCC_t2dm_tab$var <- "T2DM"


HCC_cirrh_tab <- data.frame(HCC_cirrh[["beta"]])

HCC_cirrh_tab$se <- HCC_cirrh[["se"]]

HCC_cirrh_tab$pval <- HCC_cirrh[["pval"]]

HCC_cirrh_tab$beta <- HCC_cirrh[["beta"]]

HCC_cirrh_tab <- HCC_cirrh_tab[-c(1),]

HCC_cirrh_tab <- HCC_cirrh_tab[-c(1)]
```

```
HCC_cirrh_tab$k <- HCC_cirrh[["k"]]

HCC_cirrh_tab$r2 <- HCC_cirrh[["R2"]]

HCC_cirrh_tab$var <- "Cirrhosis"



HCC_metareg <- rbind(HCC_female_tab, HCC_age_tab, HCC_t2dm_tab, HCC_cirrh_tab)

HCC_metareg$Outcome <- "HCC"



Metareg_summary <- rbind(NAFLD_Dx_metareg, Steat_metareg, NASH_metareg, AnyFib_metareg, FibAdv_metareg,
HCC_metareg)

write.table(Metareg_summary, file="Metareg_summary.csv",sep=",")


pdf(file="NASH_T2DM_bubble.pdf")

NASH_T2DM_bubble <- bubble(NASH_t2dm, xlab = "Proportion with diabetes", ylab = "LogOR NASH", col.line = "blue", lwd = 3,
studlab = FALSE, box = FALSE, pos.studlab = 4, offset = 1.5, xlim = c(0, .6), ylim = c(-0.25,1))

NASH_T2DM_bubble

dev.off()
```

```
pdf(file="AnyFib_age_bubble.pdf")

AnyFib_age_bubble <- bubble(AnyFib_age, xlab = "Age (years)", ylab = "LogOR Any Fibrosis (F0 vs F1-4)", col.line = "blue", lwd =
3, studlab = FALSE, box = FALSE, pos.studlab = 4, offset = 1.5, xlim = c(35, 55), ylim = c(-0.25, 1))

AnyFib_age_bubble

dev.off()


pdf(file="AnyFib_T2DM_bubble.pdf")

AnyFib_T2DM_bubble  <- bubble(AnyFib_t2dm, xlab = "Proportion with diabetes", ylab = "LogOR Any Fibrosis (F0 vs F1-4)",
col.line = "blue", lwd = 3, studlab = FALSE, box = FALSE, pos.studlab = 4, offset = 1.5, xlim = c(0, .5), ylim = c(-0.25, 1))

AnyFib_T2DM_bubble

dev.off()


pdf(file="Steat_T2DM_bubble.pdf")

Steat_T2DM_bubble  <- bubble(Steat_t2dm, xlab = "Proportion with diabetes", ylab = "LogOR Severe Steatosis (S1-2 vs S3)",
col.line = "blue", lwd = 3, studlab = FALSE, box = FALSE, pos.studlab = 4, offset = 1.5, xlim = c(0, .5), ylim = c(-.75, 1))

Steat_T2DM_bubble

dev.off()
```

```
pdf(file="NAFLD_Dx_pnp_bubble.pdf")

NAFLD_Dx_pnp_bubble  <- bubble(NAFLD_Dx_pnp, xlab = "Proportion with diabetes", ylab = "LogOR Severe Steatosis (S1-2 vs S3)", col.line = "blue", lwd = 3, studlab = FALSE, box = FALSE, pos.studlab = 4, offset = 1.5, xlim = c(0, .5), ylim = c(-.75, 1))

NAFLD_Dx_pnp_bubble

dev.off()
```

****

```
**gwas summary stats

*ALT

alt_GWAS_sum <- read_excel("alt_GWAS_sum.xlsx")

alt_gwas <- metagen(Beta, SE, data = alt_GWAS_sum, studlab = alt_GWAS_sum$Study, sm = "ZCOR", method.tau = "DL")

alt_gwas_ethnic <- update(alt_gwas, byvar = Ethnicity, bylab = "Ethnicity")
```

```
pdf(file="alt_gwas_ethnic_v2.pdf",width=10,height=7)

forest(alt_gwas_ethnic, comb.fixed=FALSE, col.random="red", col.diamond="red", xlab="Beta (95% CI) per T-allele", digits.se=2,

digits.pval=3, scientific.pval=FALSE, sortvar=TE, test.overall.random=TRUE, test.overall.fixed=FALSE, overall=TRUE, digits=4,

print.zval=TRUE, colgap.studlab="3cm", print.tau2=FALSE, rightlabs = c("Beta", "95% CI", "Weight"), smlab="Alanine \n

aminotransferase", leftcols = c("studlab", "num"), leftlabs = c("Study", "Total"), print.subgroup.labels = TRUE, xlim=c(-0.05,0.05),

test.effect.subgroup.random=TRUE)

dev.off()


** ALT - overall

alt_gwas_tab <- data.frame(alt_gwas_ethnic[["TE.random"]])

row.names(alt_gwas_tab) <- "Overall"

alt_gwas_tab$OR <- alt_gwas_tab$alt_gwas_ethnic...TE.random...

alt_gwas_tab <- alt_gwas_tab[-c(1)]

alt_gwas_tab$lower <- alt_gwas_ethnic[["lower.random"]]

alt_gwas_tab$upper <- alt_gwas_ethnic[["upper.random"]]

alt_gwas_tab$k <- alt_gwas_ethnic[["k"]]

alt_gwas_tab$p_z <- alt_gwas_ethnic[["pval.random"]]

alt_gwas_tab$I2 <- alt_gwas_ethnic[["I2"]]
```

```
alt_gwas_tab$p_q <- alt_gwas_ethnic[["pval.Q"]]

alt_gwas_tab$group <- "Overall"

alt_gwas_tab$model <- "Linear"

alt_gwas_tab$outcome <- "ALT"


** ALT - subgroups

alt_gwas_tab2 <- data.frame(alt_gwas_ethnic[["TE.random.w"]])

row.names(alt_gwas_tab2) <- alt_gwas_ethnic[["bylevs"]]

alt_gwas_tab2$OR <- alt_gwas_tab2$alt_gwas_ethnic...TE.random.w...

alt_gwas_tab2 <- alt_gwas_tab2[-c(1)]

alt_gwas_tab2$lower <- alt_gwas_ethnic[["lower.random.w"]]

alt_gwas_tab2$upper <- alt_gwas_ethnic[["upper.random.w"]]

alt_gwas_tab2$k <- alt_gwas_ethnic[["k.w"]]

alt_gwas_tab2$p_z <- alt_gwas_ethnic[["pval.random.w"]]

alt_gwas_tab2$I2 <- alt_gwas_ethnic[["I2.w"]]

alt_gwas_tab2$p_q <- alt_gwas_ethnic[["pval.Q.w"]]

alt_gwas_tab2$group <- row.names(alt_gwas_tab2)

alt_gwas_tab2$model <- "Linear"
```

```
alt_gwas_tab2$outcome <- "ALT"


*Total cholesterol

tchol_GWAS_sum <- read_excel("tchol_GWAS_sum.xlsx")

tchol_gwas <- metagen(Beta, SE, data = tchol_GWAS_sum, studlab = tchol_GWAS_sum$Study, sm = "ZCOR", method.tau =
"DL")

tchol_gwas_ethnic <- update(tchol_gwas, byvar = Ethnicity, bylab = "Ethnicity")

pdf(file="tchol_gwas_ethnic_v2.pdf",width=10,height=6)

forest(tchol_gwas_ethnic, comb.fixed=FALSE, col.random="red", col.diamond="red", xlab="Beta (95% CI) per T-allele", digits.se=2,
digits.pval=2, scientific.pval=TRUE, sortvar=TE, test.overall.random=TRUE, test.overall.fixed=FALSE, overall=TRUE, digits=4,
print.zval=TRUE, colgap.studlab="3cm", print.tau2=FALSE, rightlabs = c("Beta", "95% CI", "Weight"), smlab="Total \n cholesterol",
leftcols = c("studlab", "num"), leftlabs = c("Study", "Total"), print.subgroup.labels = TRUE, xlim=c(-0.05,0.05),
test.effect.subgroup.random=TRUE)

dev.off()


** Total cholesterol - overall

tchol_gwas_tab <- data.frame(tchol_gwas_ethnic[["TE.random"]])

row.names(tchol_gwas_tab) <- "Overall"
```

```
tchol_gwas_tab$OR <- tchol_gwas_tab$tchol_gwas_ethnic...TE.random...

tchol_gwas_tab <- tchol_gwas_tab[-c(1)]

tchol_gwas_tab$lower <- tchol_gwas_ethnic[["lower.random"]]

tchol_gwas_tab$upper <- tchol_gwas_ethnic[["upper.random"]]

tchol_gwas_tab$k <- tchol_gwas_ethnic[["k"]]

tchol_gwas_tab$p_z <- tchol_gwas_ethnic[["pval.random"]]

tchol_gwas_tab$I2 <- tchol_gwas_ethnic[["I2"]]

tchol_gwas_tab$p_q <- tchol_gwas_ethnic[["pval.Q"]]

tchol_gwas_tab$group <- "Overall"

tchol_gwas_tab$model <- "Linear"

tchol_gwas_tab$outcome <- "Total cholesterol"


** Total cholesterol - subgroups

tchol_gwas_tab2 <- data.frame(tchol_gwas_ethnic[["TE.random.w"]])

row.names(tchol_gwas_tab2) <- tchol_gwas_ethnic[["bylevs"]]

tchol_gwas_tab2$OR <- tchol_gwas_tab2$tchol_gwas_ethnic...TE.random.w...

tchol_gwas_tab2 <- tchol_gwas_tab2[-c(1)]

tchol_gwas_tab2$lower <- tchol_gwas_ethnic[["lower.random.w"]]
```

```
tchol_gwas_tab2$upper <- tchol_gwas_ethnic[["upper.random.w"]]

tchol_gwas_tab2$k <- tchol_gwas_ethnic[["k.w"]]

tchol_gwas_tab2$p_z <- tchol_gwas_ethnic[["pval.random.w"]]

tchol_gwas_tab2$I2 <- tchol_gwas_ethnic[["I2.w"]]

tchol_gwas_tab2$p_q <- tchol_gwas_ethnic[["pval.Q.w"]]

tchol_gwas_tab2$group <- row.names(tchol_gwas_tab2)

tchol_gwas_tab2$model <- "Linear"

tchol_gwas_tab2$outcome <- "Total cholesterol"


*HDL

hdl_GWAS_sum <- read_excel("hdl_GWAS_sum.xlsx")

hdl_gwas <- metagen(Beta, SE, data = hdl_GWAS_sum, studlab = hdl_GWAS_sum$Study, sm = "ZCOR", method.tau = "DL")

hdl_gwas_ethnic <- update(hdl_gwas, byvar = Ethnicity, bylab = "Ethnicity")


** HDL cholesterol - overall

hdl_gwas_tab <- data.frame(hdl_gwas_ethnic[["TE.random"]])

row.names(hdl_gwas_tab) <- "Overall"

hdl_gwas_tab$OR <- hdl_gwas_tab$hdl_gwas_ethnic...TE.random...
```

```
hdl_gwas_tab <- hdl_gwas_tab[-c(1)]

hdl_gwas_tab$lower <- hdl_gwas_ethnic[["lower.random"]]

hdl_gwas_tab$upper <- hdl_gwas_ethnic[["upper.random"]]

hdl_gwas_tab$k <- hdl_gwas_ethnic[["k"]]

hdl_gwas_tab$p_z <- hdl_gwas_ethnic[["pval.random"]]

hdl_gwas_tab$I2 <- hdl_gwas_ethnic[["I2"]]

hdl_gwas_tab$p_q <- hdl_gwas_ethnic[["pval.Q"]]

hdl_gwas_tab$group <- "Overall"

hdl_gwas_tab$model <- "Linear"

hdl_gwas_tab$outcome <- "HDL cholesterol"


** HDL cholesterol - subgroups

hdl_gwas_tab2 <- data.frame(hdl_gwas_ethnic[["TE.random.w"]])

row.names(hdl_gwas_tab2) <- hdl_gwas_ethnic[["bylevs"]]

hdl_gwas_tab2$OR <- hdl_gwas_tab2$hdl_gwas_ethnic...TE.random.w...

hdl_gwas_tab2 <- hdl_gwas_tab2[-c(1)]

hdl_gwas_tab2$lower <- hdl_gwas_ethnic[["lower.random.w"]]

hdl_gwas_tab2$upper <- hdl_gwas_ethnic[["upper.random.w"]]
```

```
hdl_gwas_tab2$k <- hdl_gwas_ethnic[["k.w"]]

hdl_gwas_tab2$p_z <- hdl_gwas_ethnic[["pval.random.w"]]

hdl_gwas_tab2$I2 <- hdl_gwas_ethnic[["I2.w"]]

hdl_gwas_tab2$p_q <- hdl_gwas_ethnic[["pval.Q.w"]]

hdl_gwas_tab2$group <- row.names(hdl_gwas_tab2)

hdl_gwas_tab2$model <- "Linear"

hdl_gwas_tab2$outcome <- "HDL cholesterol"



*LDL

ldl_GWAS_sum <- read_excel("ldl_GWAS_sum.xlsx")

ldl_gwas <- metagen(Beta, SE, data = ldl_GWAS_sum, studlab = ldl_GWAS_sum$Study, sm = "ZCOR", method.tau = "DL")

ldl_gwas_ethnic <- update(ldl_gwas, byvar = Ethnicity, bylab = "Ethnicity")


** LDL cholesterol - overall

ldl_gwas_tab <- data.frame(ldl_gwas_ethnic[["TE.random"]])

row.names(ldl_gwas_tab) <- "Overall"

ldl_gwas_tab$OR <- ldl_gwas_tab$ldl_gwas_ethnic...TE.random...
```

```
ldl_gwas_tab <- ldl_gwas_tab[-c(1)]

ldl_gwas_tab$lower <- ldl_gwas_ethnic[["lower.random"]]

ldl_gwas_tab$upper <- ldl_gwas_ethnic[["upper.random"]]

ldl_gwas_tab$k <- ldl_gwas_ethnic[["k"]]

ldl_gwas_tab$p_z <- ldl_gwas_ethnic[["pval.random"]]

ldl_gwas_tab$I2 <- ldl_gwas_ethnic[["I2"]]

ldl_gwas_tab$p_q <- ldl_gwas_ethnic[["pval.Q"]]

ldl_gwas_tab$group <- "Overall"

ldl_gwas_tab$model <- "Linear"

ldl_gwas_tab$outcome <- "LDL cholesterol"


** LDL cholesterol - subgroups

ldl_gwas_tab2 <- data.frame(ldl_gwas_ethnic[["TE.random.w"]])

row.names(ldl_gwas_tab2) <- ldl_gwas_ethnic[["bylevs"]]

ldl_gwas_tab2$OR <- ldl_gwas_tab2$ldl_gwas_ethnic...TE.random.w...

ldl_gwas_tab2 <- ldl_gwas_tab2[-c(1)]

ldl_gwas_tab2$lower <- ldl_gwas_ethnic[["lower.random.w"]]

ldl_gwas_tab2$upper <- ldl_gwas_ethnic[["upper.random.w"]]
```

```
ldl_gwas_tab2$k <- ldl_gwas_ethnic[["k.w"]]

ldl_gwas_tab2$p_z <- ldl_gwas_ethnic[["pval.random.w"]]

ldl_gwas_tab2$I2 <- ldl_gwas_ethnic[["I2.w"]]

ldl_gwas_tab2$p_q <- ldl_gwas_ethnic[["pval.Q.w"]]

ldl_gwas_tab2$group <- row.names(ldl_gwas_tab2)

ldl_gwas_tab2$model <- "Linear"

ldl_gwas_tab2$outcome <- "LDL cholesterol"


*Triglycerides

trig_GWAS_sum <- read_excel("trig_GWAS_sum.xlsx")

trig_gwas <- metagen(Beta, SE, data = trig_GWAS_sum, studlab = trig_GWAS_sum$Study, sm = "ZCOR", method.tau = "DL")

trig_gwas_ethnic <- update(trig_gwas, byvar = Ethnicity, bylab = "Ethnicity")

pdf(file="trig_gwas_ethnic_v2.pdf",width=10,height=6)

forest(trig_gwas_ethnic, comb.fixed=FALSE, col.random="red", col.diamond="red", xlab="Beta (95% CI) per T-allele", digits.se=2,
digits.pval=2, scientific.pval=TRUE, sortvar=TE, test.overall.random=TRUE, test.overall.fixed=FALSE, overall=TRUE, digits=4,
print.zval=TRUE, colgap.studlab="2cm", print.tau2=FALSE, rightlabs = c("Beta", "95% CI", "Weight"), smlab="Triglycerides", leftcols
= c("studlab", "num"), leftlabs = c("Study", "Total"), print.subgroup.labels = TRUE, xlim=c(-0.05,0.05),
test.effect.subgroup.random=TRUE)
```

```
dev.off()


** Trigylcerides - overall

trig_gwas_tab <- data.frame(trig_gwas_ethnic[["TE.random"]])

row.names(trig_gwas_tab) <- "Overall"

trig_gwas_tab$OR <- trig_gwas_tab$trig_gwas_ethnic...TE.random...

trig_gwas_tab <- trig_gwas_tab[-c(1)]

trig_gwas_tab$lower <- trig_gwas_ethnic[["lower.random"]]

trig_gwas_tab$upper <- trig_gwas_ethnic[["upper.random"]]

trig_gwas_tab$k <- trig_gwas_ethnic[["k"]]

trig_gwas_tab$p_z <- trig_gwas_ethnic[["pval.random"]]

trig_gwas_tab$I2 <- trig_gwas_ethnic[["I2"]]

trig_gwas_tab$p_q <- trig_gwas_ethnic[["pval.Q"]]

trig_gwas_tab$group <- "Overall"

trig_gwas_tab$model <- "Linear"

trig_gwas_tab$outcome <- "Trigylcerides"


** Trigylcerides - subgroups
```

```
trig_gwas_tab2 <- data.frame(trig_gwas_ethnic[["TE.random.w"]])

row.names(trig_gwas_tab2) <- trig_gwas_ethnic[["bylevs"]]

trig_gwas_tab2$OR <- trig_gwas_tab2$trig_gwas_ethnic...TE.random.w...

trig_gwas_tab2 <- trig_gwas_tab2[-c(1)]

trig_gwas_tab2$lower <- trig_gwas_ethnic[["lower.random.w"]]

trig_gwas_tab2$upper <- trig_gwas_ethnic[["upper.random.w"]]

trig_gwas_tab2$k <- trig_gwas_ethnic[["k.w"]]

trig_gwas_tab2$p_z <- trig_gwas_ethnic[["pval.random.w"]]

trig_gwas_tab2$I2 <- trig_gwas_ethnic[["I2.w"]]

trig_gwas_tab2$p_q <- trig_gwas_ethnic[["pval.Q.w"]]

trig_gwas_tab2$group <- row.names(trig_gwas_tab2)

trig_gwas_tab2$model <- "Linear"

trig_gwas_tab2$outcome <- "Trigylcerides"


*Insulin

insul_GWAS_sum <- read_excel("insul_GWAS_sum.xlsx")

insul_gwas <- metagen(Beta, SE, data = insul_GWAS_sum, studlab = insul_GWAS_sum$Study, sm = "ZCOR", method.tau = "DL")
```

** Insulin - overall

```
insul_gwas_tab <- data.frame(insul_gwas[["TE.random"]])

row.names(insul_gwas_tab) <- "Overall"

insul_gwas_tab$OR <- insul_gwas_tab$insul_gwas...TE.random...

insul_gwas_tab <- insul_gwas_tab[-c(1)]

insul_gwas_tab$lower <- insul_gwas[["lower.random"]]

insul_gwas_tab$upper <- insul_gwas[["upper.random"]]

insul_gwas_tab$k <- insul_gwas[["k"]]

insul_gwas_tab$p_z <- insul_gwas[["pval.random"]]

insul_gwas_tab$I2 <- insul_gwas[["I2"]]

insul_gwas_tab$p_q <- insul_gwas[["pval.Q"]]

insul_gwas_tab$group <- "Overall"

insul_gwas_tab$model <- "Linear"

insul_gwas_tab$outcome <- "Insulin"
```

** make summary table for GWAS meta-analyses

```
GWAS_sumtab <- rbind(alt_gwas_tab, alt_gwas_tab2, tchol_gwas_tab, tchol_gwas_tab2, hdl_gwas_tab, hdl_gwas_tab2,
ldl_gwas_tab, ldl_gwas_tab2, trig_gwas_tab, trig_gwas_tab2, insul_gwas_tab)
write.table(GWAS_sumtab, file="GWAS_sumtab.csv",sep=",")
```

*** Biochemistry meta-analysis from candidate gene (i.e. non-GWAS)

```
ALT_adult <- read_excel("ALT_adult.xlsx")

ALT_adult$ALT_CCCT_num <- ALT_adult$ALT_CC_num+ALT_adult$ALT_CT_num
ALT_adult$ALT_CCCT_mean <-
((ALT_adult$ALT_CC_mean*ALT_adult$ALT_CC_num)+(ALT_adult$ALT_CT_mean*ALT_adult$ALT_CT_num))/ALT_adult$ALT_
CCCT_num
ALT_adult$ALT_CCCT_SD <-
((ALT_adult$ALT_CC_SD*ALT_adult$ALT_CC_num)+(ALT_adult$ALT_CT_SD*ALT_adult$ALT_CT_num))/ALT_adult$ALT_CCC
T_num
```

```
ALT_adult$ALT_CTTT_num <- ALT_adult$ALT_TT_num+ALT_adult$ALT_CT_num
ALT_adult$ALT_CTTT_mean <-
((ALT_adult$ALT_TT_mean*ALT_adult$ALT_TT_num)+(ALT_adult$ALT_CT_mean*ALT_adult$ALT_CT_num))/ALT_adult$ALT_C
TTT_num
ALT_adult$ALT_CTTT_SD <-
((ALT_adult$ALT_TT_SD*ALT_adult$ALT_TT_num)+(ALT_adult$ALT_CT_SD*ALT_adult$ALT_CT_num))/ALT_adult$ALT_CTTT
_num

ALT_adult_add <- read_excel("ALT_adult_add.xlsx")
ALT_adult_add_reg <- metagen(Beta, SE, data = ALT_adult_add, studlab = ALT_adult_add$Paper, sm = "ZCOR", method.tau =
"DL")
ALT_adult_add_reg_ethnic <- update(ALT_adult_add_reg, byvar = Ethnicity, bylab = "Ethnicity")

ALT_adult_rec_reg <- metacont(ALT_TT_num, ALT_TT_mean, ALT_TT_SD, ALT_CCCT_num, ALT_CCCT_mean,
ALT_CCCT_SD, data = ALT_adult, studlab = paste(Paper), comb.fixed = FALSE, comb.random = TRUE, method.tau = "DL", hakn
= TRUE, prediction = FALSE, sm = "MD")
ALT_adult_rec_reg_ethnic <- update(ALT_adult_rec_reg, byvar = Ethnicity, bylab = "Ethnicity")
```

```
ALT_adult_dom_reg <- metacont(ALT_CTTT_num, ALT_CTTT_mean, ALT_CTTT_SD, ALT_CC_num, ALT_CC_mean,
ALT_CC_SD, data = ALT_adult, studlab = paste(Paper), comb.fixed = FALSE, comb.random = TRUE, method.tau = "DL", hakn =
TRUE, prediction = FALSE, sm = "MD")
ALT_adult_dom_reg_ethnic <- update(ALT_adult_dom_reg, byvar = Ethnicity, bylab = "Ethnicity")

ALT_adult_add_tab <- data.frame(ALT_adult_add_reg_ethnic[["TE.random"]])
row.names(ALT_adult_add_tab) <- "Overall"
ALT_adult_add_tab$MD <- ALT_adult_add_tab$ALT_adult_add_reg_ethnic...TE.random...
ALT_adult_add_tab <- ALT_adult_add_tab[-c(1)]
ALT_adult_add_tab$lower <- ALT_adult_add_reg_ethnic[["lower.random"]]
ALT_adult_add_tab$upper <- ALT_adult_add_reg_ethnic[["upper.random"]]
ALT_adult_add_tab$k <- ALT_adult_add_reg_ethnic[["k"]]
ALT_adult_add_tab$p_z <- ALT_adult_add_reg_ethnic[["pval.random"]]
ALT_adult_add_tab$I2 <- ALT_adult_add_reg_ethnic[["I2"]]
ALT_adult_add_tab$p_q <- ALT_adult_add_reg_ethnic[["pval.Q"]]
ALT_adult_add_tab$group <- "Overall"
ALT_adult_add_tab$model <- "Additive"
ALT_adult_add_tab$outcome <- "ALT"
```

```
ALT_adult_add_tab2 <- data.frame(ALT_adult_add_reg_ethnic[["TE.random.w"]])

row.names(ALT_adult_add_tab2) <- ALT_adult_add_reg_ethnic[["bylevs"]]

ALT_adult_add_tab2$MD <- ALT_adult_add_tab2$ALT_adult_add_reg_ethnic...TE.random.w...

ALT_adult_add_tab2 <- ALT_adult_add_tab2[-c(1)]

ALT_adult_add_tab2$lower <- ALT_adult_add_reg_ethnic[["lower.random.w"]]

ALT_adult_add_tab2$upper <- ALT_adult_add_reg_ethnic[["upper.random.w"]]

ALT_adult_add_tab2$k <- ALT_adult_add_reg_ethnic[["k.w"]]

ALT_adult_add_tab2$p_z <- ALT_adult_add_reg_ethnic[["pval.random.w"]]

ALT_adult_add_tab2$I2 <- ALT_adult_add_reg_ethnic[["I2.w"]]

ALT_adult_add_tab2$p_q <- ALT_adult_add_reg_ethnic[["pval.Q.w"]]

ALT_adult_add_tab2$group <- row.names(ALT_adult_add_tab2)

ALT_adult_add_tab2$model <- "Additive"

ALT_adult_add_tab2$outcome <- "ALT"


ALT_adult_dom_tab <- data.frame(ALT_adult_dom_reg_ethnic[["TE.random"]])

row.names(ALT_adult_dom_tab) <- "Overall"

ALT_adult_dom_tab$MD <- ALT_adult_dom_tab$ALT_adult_dom_reg_ethnic...TE.random...
```

```
ALT_adult_dom_tab <- ALT_adult_dom_tab[-c(1)]

ALT_adult_dom_tab$lower <- ALT_adult_dom_reg_ethnic[["lower.random"]]

ALT_adult_dom_tab$upper <- ALT_adult_dom_reg_ethnic[["upper.random"]]

ALT_adult_dom_tab$k <- ALT_adult_dom_reg_ethnic[["k"]]

ALT_adult_dom_tab$p_z <- ALT_adult_dom_reg_ethnic[["pval.random"]]

ALT_adult_dom_tab$I2 <- ALT_adult_dom_reg_ethnic[["I2"]]

ALT_adult_dom_tab$p_q <- ALT_adult_dom_reg_ethnic[["pval.Q"]]

ALT_adult_dom_tab$group <- "Overall"

ALT_adult_dom_tab$model <- "Dominant"

ALT_adult_dom_tab$outcome <- "ALT"


ALT_adult_dom_tab2 <- data.frame(ALT_adult_dom_reg_ethnic[["TE.random.w"]])

row.names(ALT_adult_dom_tab2) <- ALT_adult_dom_reg_ethnic[["bylevs"]]

ALT_adult_dom_tab2$MD <- ALT_adult_dom_tab2$ALT_adult_dom_reg_ethnic...TE.random.w...

ALT_adult_dom_tab2 <- ALT_adult_dom_tab2[-c(1)]

ALT_adult_dom_tab2$lower <- ALT_adult_dom_reg_ethnic[["lower.random.w"]]

ALT_adult_dom_tab2$upper <- ALT_adult_dom_reg_ethnic[["upper.random.w"]]

ALT_adult_dom_tab2$k <- ALT_adult_dom_reg_ethnic[["k.w"]]
```

```
ALT_adult_dom_tab2$p_z <- ALT_adult_dom_reg_ethnic[["pval.random.w"]]

ALT_adult_dom_tab2$I2 <- ALT_adult_dom_reg_ethnic[["I2.w"]]

ALT_adult_dom_tab2$p_q <- ALT_adult_dom_reg_ethnic[["pval.Q.w"]]

ALT_adult_dom_tab2$group <- row.names(ALT_adult_dom_tab2)

ALT_adult_dom_tab2$model <- "Dominant"

ALT_adult_dom_tab2$outcome <- "ALT"


ALT_adult_rec_tab <- data.frame(ALT_adult_rec_reg_ethnic[["TE.random"]])

row.names(ALT_adult_rec_tab) <- "Overall"

ALT_adult_rec_tab$MD <- ALT_adult_rec_tab$ALT_adult_rec_reg_ethnic...TE.random...

ALT_adult_rec_tab <- ALT_adult_rec_tab[-c(1)]

ALT_adult_rec_tab$lower <- ALT_adult_rec_reg_ethnic[["lower.random"]]

ALT_adult_rec_tab$upper <- ALT_adult_rec_reg_ethnic[["upper.random"]]

ALT_adult_rec_tab$k <- ALT_adult_rec_reg_ethnic[["k"]]

ALT_adult_rec_tab$p_z <- ALT_adult_rec_reg_ethnic[["pval.random"]]

ALT_adult_rec_tab$I2 <- ALT_adult_rec_reg_ethnic[["I2"]]

ALT_adult_rec_tab$p_q <- ALT_adult_rec_reg_ethnic[["pval.Q"]]

ALT_adult_rec_tab$group <- "Overall"
```

```
ALT_adult_rec_tab$model <- "Recessive"

ALT_adult_rec_tab$outcome <- "ALT"


ALT_adult_rec_tab2 <- data.frame(ALT_adult_rec_reg_ethnic[["TE.random.w"]])

row.names(ALT_adult_rec_tab2) <- ALT_adult_rec_reg_ethnic[["bylevs"]]

ALT_adult_rec_tab2$MD <- ALT_adult_rec_tab2$ALT_adult_rec_reg_ethnic...TE.random.w...

ALT_adult_rec_tab2 <- ALT_adult_rec_tab2[-c(1)]

ALT_adult_rec_tab2$lower <- ALT_adult_rec_reg_ethnic[["lower.random.w"]]

ALT_adult_rec_tab2$upper <- ALT_adult_rec_reg_ethnic[["upper.random.w"]]

ALT_adult_rec_tab2$k <- ALT_adult_rec_reg_ethnic[["k.w"]]

ALT_adult_rec_tab2$p_z <- ALT_adult_rec_reg_ethnic[["pval.random.w"]]

ALT_adult_rec_tab2$I2 <- ALT_adult_rec_reg_ethnic[["I2.w"]]

ALT_adult_rec_tab2$p_q <- ALT_adult_rec_reg_ethnic[["pval.Q.w"]]

ALT_adult_rec_tab2$group <- row.names(ALT_adult_rec_tab2)

ALT_adult_rec_tab2$model <- "Recessive"

ALT_adult_rec_tab2$outcome <- "ALT"
```

```
ALT_adult_sumtab <- rbind(ALT_adult_add_tab, ALT_adult_add_tab2, ALT_adult_dom_tab, ALT_adult_dom_tab2,
ALT_adult_rec_tab, ALT_adult_rec_tab2)



ALT_paed <- read_excel("ALT_paed.xlsx")


ALT_paed$ALT_CCCT_num <- ALT_paed$ALT_CC_num+ALT_paed$ALT_CT_num
ALT_paed$ALT_CCCT_mean <-
((ALT_paed$ALT_CC_mean*ALT_paed$ALT_CC_num)+(ALT_paed$ALT_CT_mean*ALT_paed$ALT_CT_num))/ALT_paed$ALT_
CCCT_num
ALT_paed$ALT_CCCT_SD <-
((ALT_paed$ALT_CC_SD*ALT_paed$ALT_CC_num)+(ALT_paed$ALT_CT_SD*ALT_paed$ALT_CT_num))/ALT_paed$ALT_CCC
T_num
ALT_paed$ALT_CTTT_num <- ALT_paed$ALT_TT_num+ALT_paed$ALT_CT_num
ALT_paed$ALT_CTTT_mean <-
((ALT_paed$ALT_TT_mean*ALT_paed$ALT_TT_num)+(ALT_paed$ALT_CT_mean*ALT_paed$ALT_CT_num))/ALT_paed$ALT_
CTTT_num
```

```
ALT_paed$ALT_CTTT_SD <-
((ALT_paed$ALT_TT_SD*ALT_paed$ALT_TT_num)+(ALT_paed$ALT_CT_SD*ALT_paed$ALT_CT_num))/ALT_paed$ALT_CTTT
_num

ALT_paed_add <- read_excel("ALT_paed_add.xlsx")
ALT_paed_add_reg <- metagen(Beta, SE, data = ALT_paed_add, studlab = ALT_paed_add$Paper, sm = "ZCOR", method.tau =
"DL")
ALT_paed_add_reg_ethnic <- update(ALT_paed_add_reg, byvar = Ethnicity, bylab = "Ethnicity")

ALT_paed_rec_reg <- metacont(ALT_TT_num, ALT_TT_mean, ALT_TT_SD, ALT_CCCT_num, ALT_CCCT_mean,
ALT_CCCT_SD, data = ALT_paed, studlab = paste(Paper), comb.fixed = FALSE, comb.random = TRUE, method.tau = "DL", hakn
= TRUE, prediction = FALSE, sm = "MD")
ALT_paed_rec_reg_ethnic <- update(ALT_paed_rec_reg, byvar = Ethnicity, bylab = "Ethnicity")

ALT_paed_dom_reg <- metacont(ALT_CTTT_num, ALT_CTTT_mean, ALT_CTTT_SD, ALT_CC_num, ALT_CC_mean,
ALT_CC_SD, data = ALT_paed, studlab = paste(Paper), comb.fixed = FALSE, comb.random = TRUE, method.tau = "DL", hakn =
TRUE, prediction = FALSE, sm = "MD")
ALT_paed_dom_reg_ethnic <- update(ALT_paed_dom_reg, byvar = Ethnicity, bylab = "Ethnicity")
```

```
ALT_paed_add_tab <- data.frame(ALT_paed_add_reg_ethnic[["TE.random"]])

row.names(ALT_paed_add_tab) <- "Overall"

ALT_paed_add_tab$MD <- ALT_paed_add_tab$ALT_paed_add_reg_ethnic...TE.random...

ALT_paed_add_tab <- ALT_paed_add_tab[-c(1)]

ALT_paed_add_tab$lower <- ALT_paed_add_reg_ethnic[["lower.random"]]

ALT_paed_add_tab$upper <- ALT_paed_add_reg_ethnic[["upper.random"]]

ALT_paed_add_tab$k <- ALT_paed_add_reg_ethnic[["k"]]

ALT_paed_add_tab$p_z <- ALT_paed_add_reg_ethnic[["pval.random"]]

ALT_paed_add_tab$I2 <- ALT_paed_add_reg_ethnic[["I2"]]

ALT_paed_add_tab$p_q <- ALT_paed_add_reg_ethnic[["pval.Q"]]

ALT_paed_add_tab$group <- "Overall"

ALT_paed_add_tab$model <- "Additive"

ALT_paed_add_tab$outcome <- "ALT"


ALT_paed_add_tab2 <- data.frame(ALT_paed_add_reg_ethnic[["TE.random.w"]])

row.names(ALT_paed_add_tab2) <- ALT_paed_add_reg_ethnic[["bylevs"]]

ALT_paed_add_tab2$MD <- ALT_paed_add_tab2$ALT_paed_add_reg_ethnic...TE.random.w...
```

```
ALT_paed_add_tab2 <- ALT_paed_add_tab2[-c(1)]

ALT_paed_add_tab2$lower <- ALT_paed_add_reg_ethnic[["lower.random.w"]]

ALT_paed_add_tab2$upper <- ALT_paed_add_reg_ethnic[["upper.random.w"]]

ALT_paed_add_tab2$k <- ALT_paed_add_reg_ethnic[["k.w"]]

ALT_paed_add_tab2$p_z <- ALT_paed_add_reg_ethnic[["pval.random.w"]]

ALT_paed_add_tab2$I2 <- ALT_paed_add_reg_ethnic[["I2.w"]]

ALT_paed_add_tab2$p_q <- ALT_paed_add_reg_ethnic[["pval.Q.w"]]

ALT_paed_add_tab2$group <- row.names(ALT_paed_add_tab2)

ALT_paed_add_tab2$model <- "Additive"

ALT_paed_add_tab2$outcome <- "ALT"


ALT_paed_dom_tab <- data.frame(ALT_paed_dom_reg_ethnic[["TE.random"]])

row.names(ALT_paed_dom_tab) <- "Overall"

ALT_paed_dom_tab$MD <- ALT_paed_dom_tab$ALT_paed_dom_reg_ethnic...TE.random...

ALT_paed_dom_tab <- ALT_paed_dom_tab[-c(1)]

ALT_paed_dom_tab$lower <- ALT_paed_dom_reg_ethnic[["lower.random"]]

ALT_paed_dom_tab$upper <- ALT_paed_dom_reg_ethnic[["upper.random"]]

ALT_paed_dom_tab$k <- ALT_paed_dom_reg_ethnic[["k"]]
```

```
ALT_paed_dom_tab$p_z <- ALT_paed_dom_reg_ethnic[["pval.random"]]

ALT_paed_dom_tab$I2 <- ALT_paed_dom_reg_ethnic[["I2"]]

ALT_paed_dom_tab$p_q <- ALT_paed_dom_reg_ethnic[["pval.Q"]]

ALT_paed_dom_tab$group <- "Overall"

ALT_paed_dom_tab$model <- "Dominant"

ALT_paed_dom_tab$outcome <- "ALT"


ALT_paed_dom_tab2 <- data.frame(ALT_paed_dom_reg_ethnic[["TE.random.w"]])

row.names(ALT_paed_dom_tab2) <- ALT_paed_dom_reg_ethnic[["bylevs"]]

ALT_paed_dom_tab2$MD <- ALT_paed_dom_tab2$ALT_paed_dom_reg_ethnic...TE.random.w...

ALT_paed_dom_tab2 <- ALT_paed_dom_tab2[-c(1)]

ALT_paed_dom_tab2$lower <- ALT_paed_dom_reg_ethnic[["lower.random.w"]]

ALT_paed_dom_tab2$upper <- ALT_paed_dom_reg_ethnic[["upper.random.w"]]

ALT_paed_dom_tab2$k <- ALT_paed_dom_reg_ethnic[["k.w"]]

ALT_paed_dom_tab2$p_z <- ALT_paed_dom_reg_ethnic[["pval.random.w"]]

ALT_paed_dom_tab2$I2 <- ALT_paed_dom_reg_ethnic[["I2.w"]]

ALT_paed_dom_tab2$p_q <- ALT_paed_dom_reg_ethnic[["pval.Q.w"]]

ALT_paed_dom_tab2$group <- row.names(ALT_paed_dom_tab2)
```

```
ALT_paed_dom_tab2$model <- "Dominant"

ALT_paed_dom_tab2$outcome <- "ALT"


ALT_paed_rec_tab <- data.frame(ALT_paed_rec_reg_ethnic[["TE.random"]])

row.names(ALT_paed_rec_tab) <- "Overall"

ALT_paed_rec_tab$MD <- ALT_paed_rec_tab$ALT_paed_rec_reg_ethnic...TE.random...

ALT_paed_rec_tab <- ALT_paed_rec_tab[-c(1)]

ALT_paed_rec_tab$lower <- ALT_paed_rec_reg_ethnic[["lower.random"]]

ALT_paed_rec_tab$upper <- ALT_paed_rec_reg_ethnic[["upper.random"]]

ALT_paed_rec_tab$k <- ALT_paed_rec_reg_ethnic[["k"]]

ALT_paed_rec_tab$p_z <- ALT_paed_rec_reg_ethnic[["pval.random"]]

ALT_paed_rec_tab$I2 <- ALT_paed_rec_reg_ethnic[["I2"]]

ALT_paed_rec_tab$p_q <- ALT_paed_rec_reg_ethnic[["pval.Q"]]

ALT_paed_rec_tab$group <- "Overall"

ALT_paed_rec_tab$model <- "Recessive"

ALT_paed_rec_tab$outcome <- "ALT"


ALT_paed_rec_tab2 <- data.frame(ALT_paed_rec_reg_ethnic[["TE.random.w"]])
```

```
row.names(ALT_paed_rec_tab2) <- ALT_paed_rec_reg_ethnic[["bylevs"]]

ALT_paed_rec_tab2$MD <- ALT_paed_rec_tab2$ALT_paed_rec_reg_ethnic...TE.random.w...

ALT_paed_rec_tab2 <- ALT_paed_rec_tab2[-c(1)]

ALT_paed_rec_tab2$lower <- ALT_paed_rec_reg_ethnic[["lower.random.w"]]

ALT_paed_rec_tab2$upper <- ALT_paed_rec_reg_ethnic[["upper.random.w"]]

ALT_paed_rec_tab2$k <- ALT_paed_rec_reg_ethnic[["k.w"]]

ALT_paed_rec_tab2$p_z <- ALT_paed_rec_reg_ethnic[["pval.random.w"]]

ALT_paed_rec_tab2$I2 <- ALT_paed_rec_reg_ethnic[["I2.w"]]

ALT_paed_rec_tab2$p_q <- ALT_paed_rec_reg_ethnic[["pval.Q.w"]]

ALT_paed_rec_tab2$group <- row.names(ALT_paed_rec_tab2)

ALT_paed_rec_tab2$model <- "Recessive"

ALT_paed_rec_tab2$outcome <- "ALT"


ALT_paed_sumtab <- rbind(ALT_paed_add_tab, ALT_paed_add_tab2, ALT_paed_dom_tab, ALT_paed_dom_tab2,

ALT_paed_rec_tab, ALT_paed_rec_tab2)
```

```
Chol_adult <- read_excel("Chol_adult.xlsx")

Chol_adult$Chol_CCCT_num <- Chol_adult$Chol_CC_num+Chol_adult$Chol_CT_num
Chol_adult$Chol_CCCT_mean <-
((Chol_adult$Chol_CC_mean*Chol_adult$Chol_CC_num)+(Chol_adult$Chol_CT_mean*Chol_adult$Chol_CT_num))/Chol_adult$
Chol_CCCT_num
Chol_adult$Chol_CCCT_SD <-
((Chol_adult$Chol_CC_SD*Chol_adult$Chol_CC_num)+(Chol_adult$Chol_CT_SD*Chol_adult$Chol_CT_num))/Chol_adult$Chol_
CCCT_num
Chol_adult$Chol_CTTT_num <- Chol_adult$Chol_TT_num+Chol_adult$Chol_CT_num
Chol_adult$Chol_CTTT_mean <-
((Chol_adult$Chol_TT_mean*Chol_adult$Chol_TT_num)+(Chol_adult$Chol_CT_mean*Chol_adult$Chol_CT_num))/Chol_adult$C
hol_CTTT_num
Chol_adult$Chol_CTTT_SD <-
((Chol_adult$Chol_TT_SD*Chol_adult$Chol_TT_num)+(Chol_adult$Chol_CT_SD*Chol_adult$Chol_CT_num))/Chol_adult$Chol_C
TTT_num
```

```
Chol_adult_add <- read_excel("Chol_adult_add.xlsx")
Chol_adult_add_reg <- metagen(Beta, SE, data = Chol_adult_add, studlab = Chol_adult_add$Paper, sm = "ZCOR", method.tau =
"DL")
Chol_adult_add_reg_ethnic <- update(Chol_adult_add_reg, byvar = Ethnicity, bylab = "Ethnicity")

Chol_adult_rec_reg <- metacont(Chol_TT_num, Chol_TT_mean, Chol_TT_SD, Chol_CCCT_num, Chol_CCCT_mean,
Chol_CCCT_SD, data = Chol_adult, studlab = paste(Paper), comb.fixed = FALSE, comb.random = TRUE, method.tau = "DL", hakn
= TRUE, prediction = FALSE, sm = "MD")
Chol_adult_rec_reg_ethnic <- update(Chol_adult_rec_reg, byvar = Ethnicity, bylab = "Ethnicity")

Chol_adult_dom_reg <- metacont(Chol_CTTT_num, Chol_CTTT_mean, Chol_CTTT_SD, Chol_CC_num, Chol_CC_mean,
Chol_CC_SD, data = Chol_adult, studlab = paste(Paper), comb.fixed = FALSE, comb.random = TRUE, method.tau = "DL", hakn =
TRUE, prediction = FALSE, sm = "MD")
Chol_adult_dom_reg_ethnic <- update(Chol_adult_dom_reg, byvar = Ethnicity, bylab = "Ethnicity")

Chol_adult_add_tab <- data.frame(Chol_adult_add_reg_ethnic[["TE.random"]])
row.names(Chol_adult_add_tab) <- "Overall"
```

```
Chol_adult_add_tab$MD <- Chol_adult_add_tab$Chol_adult_add_reg_ethnic...TE.random...

Chol_adult_add_tab <- Chol_adult_add_tab[-c(1)]

Chol_adult_add_tab$lower <- Chol_adult_add_reg_ethnic[["lower.random"]]

Chol_adult_add_tab$upper <- Chol_adult_add_reg_ethnic[["upper.random"]]

Chol_adult_add_tab$k <- Chol_adult_add_reg_ethnic[["k"]]

Chol_adult_add_tab$p_z <- Chol_adult_add_reg_ethnic[["pval.random"]]

Chol_adult_add_tab$I2 <- Chol_adult_add_reg_ethnic[["I2"]]

Chol_adult_add_tab$p_q <- Chol_adult_add_reg_ethnic[["pval.Q"]]

Chol_adult_add_tab$group <- "Overall"

Chol_adult_add_tab$model <- "Additive"

Chol_adult_add_tab$outcome <- "Chol"


Chol_adult_add_tab2 <- data.frame(Chol_adult_add_reg_ethnic[["TE.random.w"]])

row.names(Chol_adult_add_tab2) <- Chol_adult_add_reg_ethnic[["bylevs"]]

Chol_adult_add_tab2$MD <- Chol_adult_add_tab2$Chol_adult_add_reg_ethnic...TE.random.w...

Chol_adult_add_tab2 <- Chol_adult_add_tab2[-c(1)]

Chol_adult_add_tab2$lower <- Chol_adult_add_reg_ethnic[["lower.random.w"]]

Chol_adult_add_tab2$upper <- Chol_adult_add_reg_ethnic[["upper.random.w"]]
```

```
Chol_adult_add_tab2$k <- Chol_adult_add_reg_ethnic[["k.w"]]

Chol_adult_add_tab2$p_z <- Chol_adult_add_reg_ethnic[["pval.random.w"]]

Chol_adult_add_tab2$I2 <- Chol_adult_add_reg_ethnic[["I2.w"]]

Chol_adult_add_tab2$p_q <- Chol_adult_add_reg_ethnic[["pval.Q.w"]]

Chol_adult_add_tab2$group <- row.names(Chol_adult_add_tab2)

Chol_adult_add_tab2$model <- "Additive"

Chol_adult_add_tab2$outcome <- "Chol"


Chol_adult_dom_tab <- data.frame(Chol_adult_dom_reg_ethnic[["TE.random"]])

row.names(Chol_adult_dom_tab) <- "Overall"

Chol_adult_dom_tab$MD <- Chol_adult_dom_tab$Chol_adult_dom_reg_ethnic...TE.random...

Chol_adult_dom_tab <- Chol_adult_dom_tab[-c(1)]

Chol_adult_dom_tab$lower <- Chol_adult_dom_reg_ethnic[["lower.random"]]

Chol_adult_dom_tab$upper <- Chol_adult_dom_reg_ethnic[["upper.random"]]

Chol_adult_dom_tab$k <- Chol_adult_dom_reg_ethnic[["k"]]

Chol_adult_dom_tab$p_z <- Chol_adult_dom_reg_ethnic[["pval.random"]]

Chol_adult_dom_tab$I2 <- Chol_adult_dom_reg_ethnic[["I2"]]

Chol_adult_dom_tab$p_q <- Chol_adult_dom_reg_ethnic[["pval.Q"]]
```

```
Chol_adult_dom_tab$group <- "Overall"

Chol_adult_dom_tab$model <- "Dominant"

Chol_adult_dom_tab$outcome <- "Chol"


Chol_adult_dom_tab2 <- data.frame(Chol_adult_dom_reg_ethnic[["TE.random.w"]])

row.names(Chol_adult_dom_tab2) <- Chol_adult_dom_reg_ethnic[["bylevs"]]

Chol_adult_dom_tab2$MD <- Chol_adult_dom_tab2$Chol_adult_dom_reg_ethnic...TE.random.w...

Chol_adult_dom_tab2 <- Chol_adult_dom_tab2[-c(1)]

Chol_adult_dom_tab2$lower <- Chol_adult_dom_reg_ethnic[["lower.random.w"]]

Chol_adult_dom_tab2$upper <- Chol_adult_dom_reg_ethnic[["upper.random.w"]]

Chol_adult_dom_tab2$k <- Chol_adult_dom_reg_ethnic[["k.w"]]

Chol_adult_dom_tab2$p_z <- Chol_adult_dom_reg_ethnic[["pval.random.w"]]

Chol_adult_dom_tab2$I2 <- Chol_adult_dom_reg_ethnic[["I2.w"]]

Chol_adult_dom_tab2$p_q <- Chol_adult_dom_reg_ethnic[["pval.Q.w"]]

Chol_adult_dom_tab2$group <- row.names(Chol_adult_dom_tab2)

Chol_adult_dom_tab2$model <- "Dominant"

Chol_adult_dom_tab2$outcome <- "Chol"
```

```
Chol_adult_rec_tab <- data.frame(Chol_adult_rec_reg_ethnic[["TE.random"]])

row.names(Chol_adult_rec_tab) <- "Overall"

Chol_adult_rec_tab$MD <- Chol_adult_rec_tab$Chol_adult_rec_reg_ethnic...TE.random...

Chol_adult_rec_tab <- Chol_adult_rec_tab[-c(1)]

Chol_adult_rec_tab$lower <- Chol_adult_rec_reg_ethnic[["lower.random"]]

Chol_adult_rec_tab$upper <- Chol_adult_rec_reg_ethnic[["upper.random"]]

Chol_adult_rec_tab$k <- Chol_adult_rec_reg_ethnic[["k"]]

Chol_adult_rec_tab$p_z <- Chol_adult_rec_reg_ethnic[["pval.random"]]

Chol_adult_rec_tab$I2 <- Chol_adult_rec_reg_ethnic[["I2"]]

Chol_adult_rec_tab$p_q <- Chol_adult_rec_reg_ethnic[["pval.Q"]]

Chol_adult_rec_tab$group <- "Overall"

Chol_adult_rec_tab$model <- "Recessive"

Chol_adult_rec_tab$outcome <- "Chol"


Chol_adult_rec_tab2 <- data.frame(Chol_adult_rec_reg_ethnic[["TE.random.w"]])

row.names(Chol_adult_rec_tab2) <- Chol_adult_rec_reg_ethnic[["bylevs"]]

Chol_adult_rec_tab2$MD <- Chol_adult_rec_tab2$Chol_adult_rec_reg_ethnic...TE.random.w...

Chol_adult_rec_tab2 <- Chol_adult_rec_tab2[-c(1)]
```

```
Chol_adult_rec_tab2$lower <- Chol_adult_rec_reg_ethnic[["lower.random.w"]]

Chol_adult_rec_tab2$upper <- Chol_adult_rec_reg_ethnic[["upper.random.w"]]

Chol_adult_rec_tab2$k <- Chol_adult_rec_reg_ethnic[["k.w"]]

Chol_adult_rec_tab2$p_z <- Chol_adult_rec_reg_ethnic[["pval.random.w"]]

Chol_adult_rec_tab2$I2 <- Chol_adult_rec_reg_ethnic[["I2.w"]]

Chol_adult_rec_tab2$p_q <- Chol_adult_rec_reg_ethnic[["pval.Q.w"]]

Chol_adult_rec_tab2$group <- row.names(Chol_adult_rec_tab2)

Chol_adult_rec_tab2$model <- "Recessive"

Chol_adult_rec_tab2$outcome <- "Chol"


Chol_adult_sumtab <- rbind(Chol_adult_add_tab, Chol_adult_add_tab2, Chol_adult_dom_tab, Chol_adult_dom_tab2,
Chol_adult_rec_tab, Chol_adult_rec_tab2)




Chol_paed <- read_excel("Chol_paed.xlsx")
```

```
Chol_paed$Chol_CCCT_num <- Chol_paed$Chol_CC_num+Chol_paed$Chol_CT_num
Chol_paed$Chol_CCCT_mean <-
((Chol_paed$Chol_CC_mean*Chol_paed$Chol_CC_num)+(Chol_paed$Chol_CT_mean*Chol_paed$Chol_CT_num))/Chol_paed$
Chol_CCCT_num
Chol_paed$Chol_CCCT_SD <-
((Chol_paed$Chol_CC_SD*Chol_paed$Chol_CC_num)+(Chol_paed$Chol_CT_SD*Chol_paed$Chol_CT_num))/Chol_paed$Chol_
CCCT_num
Chol_paed$Chol_CTTT_num <- Chol_paed$Chol_TT_num+Chol_paed$Chol_CT_num
Chol_paed$Chol_CTTT_mean <-
((Chol_paed$Chol_TT_mean*Chol_paed$Chol_TT_num)+(Chol_paed$Chol_CT_mean*Chol_paed$Chol_CT_num))/Chol_paed$C
hol_CTTT_num
Chol_paed$Chol_CTTT_SD <-
((Chol_paed$Chol_TT_SD*Chol_paed$Chol_TT_num)+(Chol_paed$Chol_CT_SD*Chol_paed$Chol_CT_num))/Chol_paed$Chol_
CTTT_num

Chol_paed_add <- read_excel("Chol_paed_add.xlsx")
Chol_paed_add_reg <- metagen(Beta, SE, data = Chol_paed_add, studlab = Chol_paed_add$Paper, sm = "ZCOR", method.tau =
"DL")
```

```
Chol_paed_add_reg_ethnic <- update(Chol_paed_add_reg, byvar = Ethnicity, bylab = "Ethnicity")

Chol_paed_rec_reg <- metacont(Chol_TT_num, Chol_TT_mean, Chol_TT_SD, Chol_CCCT_num, Chol_CCCT_mean,
Chol_CCCT_SD, data = Chol_paed, studlab = paste(Paper), comb.fixed = FALSE, comb.random = TRUE, method.tau = "DL", hakn
= TRUE, prediction = FALSE, sm = "MD")
Chol_paed_rec_reg_ethnic <- update(Chol_paed_rec_reg, byvar = Ethnicity, bylab = "Ethnicity")

Chol_paed_dom_reg <- metacont(Chol_CTTT_num, Chol_CTTT_mean, Chol_CTTT_SD, Chol_CC_num, Chol_CC_mean,
Chol_CC_SD, data = Chol_paed, studlab = paste(Paper), comb.fixed = FALSE, comb.random = TRUE, method.tau = "DL", hakn =
TRUE, prediction = FALSE, sm = "MD")
Chol_paed_dom_reg_ethnic <- update(Chol_paed_dom_reg, byvar = Ethnicity, bylab = "Ethnicity")

Chol_paed_add_tab <- data.frame(Chol_paed_add_reg_ethnic[["TE.random"]])
row.names(Chol_paed_add_tab) <- "Overall"
Chol_paed_add_tab$MD <- Chol_paed_add_tab$Chol_paed_add_reg_ethnic...TE.random...
Chol_paed_add_tab <- Chol_paed_add_tab[-c(1)]
Chol_paed_add_tab$lower <- Chol_paed_add_reg_ethnic[["lower.random"]]
Chol_paed_add_tab$upper <- Chol_paed_add_reg_ethnic[["upper.random"]]
```

```
Chol_paed_add_tab$k <- Chol_paed_add_reg_ethnic[["k"]]

Chol_paed_add_tab$p_z <- Chol_paed_add_reg_ethnic[["pval.random"]]

Chol_paed_add_tab$I2 <- Chol_paed_add_reg_ethnic[["I2"]]

Chol_paed_add_tab$p_q <- Chol_paed_add_reg_ethnic[["pval.Q"]]

Chol_paed_add_tab$group <- "Overall"

Chol_paed_add_tab$model <- "Additive"

Chol_paed_add_tab$outcome <- "Chol"


Chol_paed_add_tab2 <- data.frame(Chol_paed_add_reg_ethnic[["TE.random.w"]])

row.names(Chol_paed_add_tab2) <- Chol_paed_add_reg_ethnic[["bylevs"]]

Chol_paed_add_tab2$MD <- Chol_paed_add_tab2$Chol_paed_add_reg_ethnic...TE.random.w...

Chol_paed_add_tab2 <- Chol_paed_add_tab2[-c(1)]

Chol_paed_add_tab2$lower <- Chol_paed_add_reg_ethnic[["lower.random.w"]]

Chol_paed_add_tab2$upper <- Chol_paed_add_reg_ethnic[["upper.random.w"]]

Chol_paed_add_tab2$k <- Chol_paed_add_reg_ethnic[["k.w"]]

Chol_paed_add_tab2$p_z <- Chol_paed_add_reg_ethnic[["pval.random.w"]]

Chol_paed_add_tab2$I2 <- Chol_paed_add_reg_ethnic[["I2.w"]]

Chol_paed_add_tab2$p_q <- Chol_paed_add_reg_ethnic[["pval.Q.w"]]
```

```
Chol_paed_add_tab2$group <- row.names(Chol_paed_add_tab2)

Chol_paed_add_tab2$model <- "Additive"

Chol_paed_add_tab2$outcome <- "Chol"


Chol_paed_dom_tab <- data.frame(Chol_paed_dom_reg_ethnic[["TE.random"]])

row.names(Chol_paed_dom_tab) <- "Overall"

Chol_paed_dom_tab$MD <- Chol_paed_dom_tab$Chol_paed_dom_reg_ethnic...TE.random...

Chol_paed_dom_tab <- Chol_paed_dom_tab[-c(1)]

Chol_paed_dom_tab$lower <- Chol_paed_dom_reg_ethnic[["lower.random"]]

Chol_paed_dom_tab$upper <- Chol_paed_dom_reg_ethnic[["upper.random"]]

Chol_paed_dom_tab$k <- Chol_paed_dom_reg_ethnic[["k"]]

Chol_paed_dom_tab$p_z <- Chol_paed_dom_reg_ethnic[["pval.random"]]

Chol_paed_dom_tab$I2 <- Chol_paed_dom_reg_ethnic[["I2"]]

Chol_paed_dom_tab$p_q <- Chol_paed_dom_reg_ethnic[["pval.Q"]]

Chol_paed_dom_tab$group <- "Overall"

Chol_paed_dom_tab$model <- "Dominant"

Chol_paed_dom_tab$outcome <- "Chol"
```

```
Chol_paed_dom_tab2 <- data.frame(Chol_paed_dom_reg_ethnic[["TE.random.w"]])

row.names(Chol_paed_dom_tab2) <- Chol_paed_dom_reg_ethnic[["bylevs"]]

Chol_paed_dom_tab2$MD <- Chol_paed_dom_tab2$Chol_paed_dom_reg_ethnic...TE.random.w...

Chol_paed_dom_tab2 <- Chol_paed_dom_tab2[-c(1)]

Chol_paed_dom_tab2$lower <- Chol_paed_dom_reg_ethnic[["lower.random.w"]]

Chol_paed_dom_tab2$upper <- Chol_paed_dom_reg_ethnic[["upper.random.w"]]

Chol_paed_dom_tab2$k <- Chol_paed_dom_reg_ethnic[["k.w"]]

Chol_paed_dom_tab2$p_z <- Chol_paed_dom_reg_ethnic[["pval.random.w"]]

Chol_paed_dom_tab2$I2 <- Chol_paed_dom_reg_ethnic[["I2.w"]]

Chol_paed_dom_tab2$p_q <- Chol_paed_dom_reg_ethnic[["pval.Q.w"]]

Chol_paed_dom_tab2$group <- row.names(Chol_paed_dom_tab2)

Chol_paed_dom_tab2$model <- "Dominant"

Chol_paed_dom_tab2$outcome <- "Chol"


Chol_paed_rec_tab <- data.frame(Chol_paed_rec_reg_ethnic[["TE.random"]])

row.names(Chol_paed_rec_tab) <- "Overall"

Chol_paed_rec_tab$MD <- Chol_paed_rec_tab$Chol_paed_rec_reg_ethnic...TE.random...

Chol_paed_rec_tab <- Chol_paed_rec_tab[-c(1)]
```

```
Chol_paed_rec_tab$lower <- Chol_paed_rec_reg_ethnic[["lower.random"]]
Chol_paed_rec_tab$upper <- Chol_paed_rec_reg_ethnic[["upper.random"]]
Chol_paed_rec_tab$k <- Chol_paed_rec_reg_ethnic[["k"]]
Chol_paed_rec_tab$p_z <- Chol_paed_rec_reg_ethnic[["pval.random"]]
Chol_paed_rec_tab$I2 <- Chol_paed_rec_reg_ethnic[["I2"]]
Chol_paed_rec_tab$p_q <- Chol_paed_rec_reg_ethnic[["pval.Q"]]
Chol_paed_rec_tab$group <- "Overall"
Chol_paed_rec_tab$model <- "Recessive"
Chol_paed_rec_tab$outcome <- "Chol"

Chol_paed_rec_tab2 <- data.frame(Chol_paed_rec_reg_ethnic[["TE.random.w"]])
row.names(Chol_paed_rec_tab2) <- Chol_paed_rec_reg_ethnic[["bylevs"]]
Chol_paed_rec_tab2$MD <- Chol_paed_rec_tab2$Chol_paed_rec_reg_ethnic...TE.random.w...
Chol_paed_rec_tab2 <- Chol_paed_rec_tab2[-c(1)]
Chol_paed_rec_tab2$lower <- Chol_paed_rec_reg_ethnic[["lower.random.w"]]
Chol_paed_rec_tab2$upper <- Chol_paed_rec_reg_ethnic[["upper.random.w"]]
Chol_paed_rec_tab2$k <- Chol_paed_rec_reg_ethnic[["k.w"]]
Chol_paed_rec_tab2$p_z <- Chol_paed_rec_reg_ethnic[["pval.random.w"]]
```

```
Chol_paed_rec_tab2$I2 <- Chol_paed_rec_reg_ethnic[["I2.w"]]

Chol_paed_rec_tab2$p_q <- Chol_paed_rec_reg_ethnic[["pval.Q.w"]]

Chol_paed_rec_tab2$group <- row.names(Chol_paed_rec_tab2)

Chol_paed_rec_tab2$model <- "Recessive"

Chol_paed_rec_tab2$outcome <- "Chol"


Chol_paed_sumtab <- rbind(Chol_paed_add_tab, Chol_paed_add_tab2, Chol_paed_dom_tab, Chol_paed_dom_tab2,
Chol_paed_rec_tab, Chol_paed_rec_tab2)


HDL_adult <- read_excel("HDL_adult.xlsx")


HDL_adult$HDL_CCCT_num <- HDL_adult$HDL_CC_num+HDL_adult$HDL_CT_num
```

```
HDL_adult$HDL_CCCT_mean <-
((HDL_adult$HDL_CC_mean*HDL_adult$HDL_CC_num)+(HDL_adult$HDL_CT_mean*HDL_adult$HDL_CT_num))/HDL_adult$H
DL_CCCT_num
HDL_adult$HDL_CCCT_SD <-
((HDL_adult$HDL_CC_SD*HDL_adult$HDL_CC_num)+(HDL_adult$HDL_CT_SD*HDL_adult$HDL_CT_num))/HDL_adult$HDL_C
CCT_num
HDL_adult$HDL_CTTT_num <- HDL_adult$HDL_TT_num+HDL_adult$HDL_CT_num
HDL_adult$HDL_CTTT_mean <-
((HDL_adult$HDL_TT_mean*HDL_adult$HDL_TT_num)+(HDL_adult$HDL_CT_mean*HDL_adult$HDL_CT_num))/HDL_adult$HD
L_CTTT_num
HDL_adult$HDL_CTTT_SD <-
((HDL_adult$HDL_TT_SD*HDL_adult$HDL_TT_num)+(HDL_adult$HDL_CT_SD*HDL_adult$HDL_CT_num))/HDL_adult$HDL_C
TTT_num

HDL_adult_add <- read_excel("HDL_adult_add.xlsx")
HDL_adult_add_reg <- metagen(Beta, SE, data = HDL_adult_add, studlab = HDL_adult_add$Paper, sm = "ZCOR", method.tau =
"DL")
HDL_adult_add_reg_ethnic <- update(HDL_adult_add_reg, byvar = Ethnicity, bylab = "Ethnicity")
```

```
HDL_adult_rec_reg <- metacont(HDL_TT_num, HDL_TT_mean, HDL_TT_SD, HDL_CCCT_num, HDL_CCCT_mean,
HDL_CCCT_SD, data = HDL_adult, studlab = paste(Paper), comb.fixed = FALSE, comb.random = TRUE, method.tau = "DL", hakn
= TRUE, prediction = FALSE, sm = "MD")
HDL_adult_rec_reg_ethnic <- update(HDL_adult_rec_reg, byvar = Ethnicity, bylab = "Ethnicity")

HDL_adult_dom_reg <- metacont(HDL_CTTT_num, HDL_CTTT_mean, HDL_CTTT_SD, HDL_CC_num, HDL_CC_mean,
HDL_CC_SD, data = HDL_adult, studlab = paste(Paper), comb.fixed = FALSE, comb.random = TRUE, method.tau = "DL", hakn =
TRUE, prediction = FALSE, sm = "MD")
HDL_adult_dom_reg_ethnic <- update(HDL_adult_dom_reg, byvar = Ethnicity, bylab = "Ethnicity")

HDL_adult_add_tab <- data.frame(HDL_adult_add_reg_ethnic[["TE.random"]])
row.names(HDL_adult_add_tab) <- "Overall"
HDL_adult_add_tab$MD <- HDL_adult_add_tab$HDL_adult_add_reg_ethnic...TE.random...
HDL_adult_add_tab <- HDL_adult_add_tab[-c(1)]
HDL_adult_add_tab$lower <- HDL_adult_add_reg_ethnic[["lower.random"]]
HDL_adult_add_tab$upper <- HDL_adult_add_reg_ethnic[["upper.random"]]
HDL_adult_add_tab$k <- HDL_adult_add_reg_ethnic[["k"]]
```

```
HDL_adult_add_tab$p_z <- HDL_adult_add_reg_ethnic[["pval.random"]]

HDL_adult_add_tab$I2 <- HDL_adult_add_reg_ethnic[["I2"]]

HDL_adult_add_tab$p_q <- HDL_adult_add_reg_ethnic[["pval.Q"]]

HDL_adult_add_tab$group <- "Overall"

HDL_adult_add_tab$model <- "Additive"

HDL_adult_add_tab$outcome <- "HDL"


HDL_adult_add_tab2 <- data.frame(HDL_adult_add_reg_ethnic[["TE.random.w"]])

row.names(HDL_adult_add_tab2) <- HDL_adult_add_reg_ethnic[["bylevs"]]

HDL_adult_add_tab2$MD <- HDL_adult_add_tab2$HDL_adult_add_reg_ethnic...TE.random.w...

HDL_adult_add_tab2 <- HDL_adult_add_tab2[-c(1)]

HDL_adult_add_tab2$lower <- HDL_adult_add_reg_ethnic[["lower.random.w"]]

HDL_adult_add_tab2$upper <- HDL_adult_add_reg_ethnic[["upper.random.w"]]

HDL_adult_add_tab2$k <- HDL_adult_add_reg_ethnic[["k.w"]]

HDL_adult_add_tab2$p_z <- HDL_adult_add_reg_ethnic[["pval.random.w"]]

HDL_adult_add_tab2$I2 <- HDL_adult_add_reg_ethnic[["I2.w"]]

HDL_adult_add_tab2$p_q <- HDL_adult_add_reg_ethnic[["pval.Q.w"]]

HDL_adult_add_tab2$group <- row.names(HDL_adult_add_tab2)
```

```
HDL_adult_add_tab2$model <- "Additive"

HDL_adult_add_tab2$outcome <- "HDL"


HDL_adult_dom_tab <- data.frame(HDL_adult_dom_reg_ethnic[["TE.random"]])

row.names(HDL_adult_dom_tab) <- "Overall"

HDL_adult_dom_tab$MD <- HDL_adult_dom_tab$HDL_adult_dom_reg_ethnic...TE.random...

HDL_adult_dom_tab <- HDL_adult_dom_tab[-c(1)]

HDL_adult_dom_tab$lower <- HDL_adult_dom_reg_ethnic[["lower.random"]]

HDL_adult_dom_tab$upper <- HDL_adult_dom_reg_ethnic[["upper.random"]]

HDL_adult_dom_tab$k <- HDL_adult_dom_reg_ethnic[["k"]]

HDL_adult_dom_tab$p_z <- HDL_adult_dom_reg_ethnic[["pval.random"]]

HDL_adult_dom_tab$I2 <- HDL_adult_dom_reg_ethnic[["I2"]]

HDL_adult_dom_tab$p_q <- HDL_adult_dom_reg_ethnic[["pval.Q"]]

HDL_adult_dom_tab$group <- "Overall"

HDL_adult_dom_tab$model <- "Dominant"

HDL_adult_dom_tab$outcome <- "HDL"


HDL_adult_dom_tab2 <- data.frame(HDL_adult_dom_reg_ethnic[["TE.random.w"]])
```

```
row.names(HDL_adult_dom_tab2) <- HDL_adult_dom_reg_ethnic[["bylevs"]]

HDL_adult_dom_tab2$MD <- HDL_adult_dom_tab2$HDL_adult_dom_reg_ethnic...TE.random.w...

HDL_adult_dom_tab2 <- HDL_adult_dom_tab2[-c(1)]

HDL_adult_dom_tab2$lower <- HDL_adult_dom_reg_ethnic[["lower.random.w"]]

HDL_adult_dom_tab2$upper <- HDL_adult_dom_reg_ethnic[["upper.random.w"]]

HDL_adult_dom_tab2$k <- HDL_adult_dom_reg_ethnic[["k.w"]]

HDL_adult_dom_tab2$p_z <- HDL_adult_dom_reg_ethnic[["pval.random.w"]]

HDL_adult_dom_tab2$I2 <- HDL_adult_dom_reg_ethnic[["I2.w"]]

HDL_adult_dom_tab2$p_q <- HDL_adult_dom_reg_ethnic[["pval.Q.w"]]

HDL_adult_dom_tab2$group <- row.names(HDL_adult_dom_tab2)

HDL_adult_dom_tab2$model <- "Dominant"

HDL_adult_dom_tab2$outcome <- "HDL"


HDL_adult_rec_tab <- data.frame(HDL_adult_rec_reg_ethnic[["TE.random"]])

row.names(HDL_adult_rec_tab) <- "Overall"

HDL_adult_rec_tab$MD <- HDL_adult_rec_tab$HDL_adult_rec_reg_ethnic...TE.random...

HDL_adult_rec_tab <- HDL_adult_rec_tab[-c(1)]

HDL_adult_rec_tab$lower <- HDL_adult_rec_reg_ethnic[["lower.random"]]
```

```
HDL_adult_rec_tab$upper <- HDL_adult_rec_reg_ethnic[["upper.random"]]

HDL_adult_rec_tab$k <- HDL_adult_rec_reg_ethnic[["k"]]

HDL_adult_rec_tab$p_z <- HDL_adult_rec_reg_ethnic[["pval.random"]]

HDL_adult_rec_tab$I2 <- HDL_adult_rec_reg_ethnic[["I2"]]

HDL_adult_rec_tab$p_q <- HDL_adult_rec_reg_ethnic[["pval.Q"]]

HDL_adult_rec_tab$group <- "Overall"

HDL_adult_rec_tab$model <- "Recessive"

HDL_adult_rec_tab$outcome <- "HDL"


HDL_adult_rec_tab2 <- data.frame(HDL_adult_rec_reg_ethnic[["TE.random.w"]])

row.names(HDL_adult_rec_tab2) <- HDL_adult_rec_reg_ethnic[["bylevs"]]

HDL_adult_rec_tab2$MD <- HDL_adult_rec_tab2$HDL_adult_rec_reg_ethnic...TE.random.w...

HDL_adult_rec_tab2 <- HDL_adult_rec_tab2[-c(1)]

HDL_adult_rec_tab2$lower <- HDL_adult_rec_reg_ethnic[["lower.random.w"]]

HDL_adult_rec_tab2$upper <- HDL_adult_rec_reg_ethnic[["upper.random.w"]]

HDL_adult_rec_tab2$k <- HDL_adult_rec_reg_ethnic[["k.w"]]

HDL_adult_rec_tab2$p_z <- HDL_adult_rec_reg_ethnic[["pval.random.w"]]

HDL_adult_rec_tab2$I2 <- HDL_adult_rec_reg_ethnic[["I2.w"]]
```

```
HDL_adult_rec_tab2$p_q <- HDL_adult_rec_reg_ethnic[["pval.Q.w"]]

HDL_adult_rec_tab2$group <- row.names(HDL_adult_rec_tab2)

HDL_adult_rec_tab2$model <- "Recessive"

HDL_adult_rec_tab2$outcome <- "HDL"


HDL_adult_sumtab <- rbind(HDL_adult_add_tab, HDL_adult_add_tab2, HDL_adult_dom_tab, HDL_adult_dom_tab2,
HDL_adult_rec_tab, HDL_adult_rec_tab2)




HDL_paed <- read_excel("HDL_paed.xlsx")


HDL_paed$HDL_CCCT_num <- HDL_paed$HDL_CC_num+HDL_paed$HDL_CT_num

HDL_paed$HDL_CCCT_mean <-
((HDL_paed$HDL_CC_mean*HDL_paed$HDL_CC_num)+(HDL_paed$HDL_CT_mean*HDL_paed$HDL_CT_num))/HDL_paed$H
DL_CCCT_num
```

```
HDL_paed$HDL_CCCT_SD <-
((HDL_paed$HDL_CC_SD*HDL_paed$HDL_CC_num)+(HDL_paed$HDL_CT_SD*HDL_paed$HDL_CT_num))/HDL_paed$HDL_
CCCT_num
HDL_paed$HDL_CTTT_num <- HDL_paed$HDL_TT_num+HDL_paed$HDL_CT_num
HDL_paed$HDL_CTTT_mean <-
((HDL_paed$HDL_TT_mean*HDL_paed$HDL_TT_num)+(HDL_paed$HDL_CT_mean*HDL_paed$HDL_CT_num))/HDL_paed$H
DL_CTTT_num
HDL_paed$HDL_CTTT_SD <-
((HDL_paed$HDL_TT_SD*HDL_paed$HDL_TT_num)+(HDL_paed$HDL_CT_SD*HDL_paed$HDL_CT_num))/HDL_paed$HDL_C
TTT_num

HDL_paed_add <- read_excel("HDL_paed_add.xlsx")
HDL_paed_add_reg <- metagen(Beta, SE, data = HDL_paed_add, studlab = HDL_paed_add$Paper, sm = "ZCOR", method.tau =
"DL")
HDL_paed_add_reg_ethnic <- update(HDL_paed_add_reg, byvar = Ethnicity, bylab = "Ethnicity")
```

```
HDL_paed_rec_reg <- metacont(HDL_TT_num, HDL_TT_mean, HDL_TT_SD, HDL_CCCT_num, HDL_CCCT_mean,
HDL_CCCT_SD, data = HDL_paed, studlab = paste(Paper), comb.fixed = FALSE, comb.random = TRUE, method.tau = "DL", hakn
= TRUE, prediction = FALSE, sm = "MD")
HDL_paed_rec_reg_ethnic <- update(HDL_paed_rec_reg, byvar = Ethnicity, bylab = "Ethnicity")

HDL_paed_dom_reg <- metacont(HDL_CTTT_num, HDL_CTTT_mean, HDL_CTTT_SD, HDL_CC_num, HDL_CC_mean,
HDL_CC_SD, data = HDL_paed, studlab = paste(Paper), comb.fixed = FALSE, comb.random = TRUE, method.tau = "DL", hakn =
TRUE, prediction = FALSE, sm = "MD")
HDL_paed_dom_reg_ethnic <- update(HDL_paed_dom_reg, byvar = Ethnicity, bylab = "Ethnicity")

HDL_paed_add_tab <- data.frame(HDL_paed_add_reg_ethnic[["TE.random"]])
row.names(HDL_paed_add_tab) <- "Overall"
HDL_paed_add_tab$MD <- HDL_paed_add_tab$HDL_paed_add_reg_ethnic...TE.random...
HDL_paed_add_tab <- HDL_paed_add_tab[-c(1)]
HDL_paed_add_tab$lower <- HDL_paed_add_reg_ethnic[["lower.random"]]
HDL_paed_add_tab$upper <- HDL_paed_add_reg_ethnic[["upper.random"]]
HDL_paed_add_tab$k <- HDL_paed_add_reg_ethnic[["k"]]
HDL_paed_add_tab$p_z <- HDL_paed_add_reg_ethnic[["pval.random"]]
```

```
HDL_paed_add_tab$I2 <- HDL_paed_add_reg_ethnic[["I2"]]

HDL_paed_add_tab$p_q <- HDL_paed_add_reg_ethnic[["pval.Q"]]

HDL_paed_add_tab$group <- "Overall"

HDL_paed_add_tab$model <- "Additive"

HDL_paed_add_tab$outcome <- "HDL"


HDL_paed_add_tab2 <- data.frame(HDL_paed_add_reg_ethnic[["TE.random.w"]])

row.names(HDL_paed_add_tab2) <- HDL_paed_add_reg_ethnic[["bylevs"]]

HDL_paed_add_tab2$MD <- HDL_paed_add_tab2$HDL_paed_add_reg_ethnic...TE.random.w...

HDL_paed_add_tab2 <- HDL_paed_add_tab2[-c(1)]

HDL_paed_add_tab2$lower <- HDL_paed_add_reg_ethnic[["lower.random.w"]]

HDL_paed_add_tab2$upper <- HDL_paed_add_reg_ethnic[["upper.random.w"]]

HDL_paed_add_tab2$k <- HDL_paed_add_reg_ethnic[["k.w"]]

HDL_paed_add_tab2$p_z <- HDL_paed_add_reg_ethnic[["pval.random.w"]]

HDL_paed_add_tab2$I2 <- HDL_paed_add_reg_ethnic[["I2.w"]]

HDL_paed_add_tab2$p_q <- HDL_paed_add_reg_ethnic[["pval.Q.w"]]

HDL_paed_add_tab2$group <- row.names(HDL_paed_add_tab2)

HDL_paed_add_tab2$model <- "Additive"
```

```
HDL_paed_add_tab2$outcome <- "HDL"


HDL_paed_dom_tab <- data.frame(HDL_paed_dom_reg_ethnic[["TE.random"]])

row.names(HDL_paed_dom_tab) <- "Overall"

HDL_paed_dom_tab$MD <- HDL_paed_dom_tab$HDL_paed_dom_reg_ethnic...TE.random...

HDL_paed_dom_tab <- HDL_paed_dom_tab[-c(1)]

HDL_paed_dom_tab$lower <- HDL_paed_dom_reg_ethnic[["lower.random"]]

HDL_paed_dom_tab$upper <- HDL_paed_dom_reg_ethnic[["upper.random"]]

HDL_paed_dom_tab$k <- HDL_paed_dom_reg_ethnic[["k"]]

HDL_paed_dom_tab$p_z <- HDL_paed_dom_reg_ethnic[["pval.random"]]

HDL_paed_dom_tab$I2 <- HDL_paed_dom_reg_ethnic[["I2"]]

HDL_paed_dom_tab$p_q <- HDL_paed_dom_reg_ethnic[["pval.Q"]]

HDL_paed_dom_tab$group <- "Overall"

HDL_paed_dom_tab$model <- "Dominant"

HDL_paed_dom_tab$outcome <- "HDL"


HDL_paed_dom_tab2 <- data.frame(HDL_paed_dom_reg_ethnic[["TE.random.w"]])

row.names(HDL_paed_dom_tab2) <- HDL_paed_dom_reg_ethnic[["bylevs"]]
```

```
HDL_paed_dom_tab2$MD <- HDL_paed_dom_tab2$HDL_paed_dom_reg_ethnic...TE.random.w...

HDL_paed_dom_tab2 <- HDL_paed_dom_tab2[-c(1)]

HDL_paed_dom_tab2$lower <- HDL_paed_dom_reg_ethnic[["lower.random.w"]]

HDL_paed_dom_tab2$upper <- HDL_paed_dom_reg_ethnic[["upper.random.w"]]

HDL_paed_dom_tab2$k <- HDL_paed_dom_reg_ethnic[["k.w"]]

HDL_paed_dom_tab2$p_z <- HDL_paed_dom_reg_ethnic[["pval.random.w"]]

HDL_paed_dom_tab2$I2 <- HDL_paed_dom_reg_ethnic[["I2.w"]]

HDL_paed_dom_tab2$p_q <- HDL_paed_dom_reg_ethnic[["pval.Q.w"]]

HDL_paed_dom_tab2$group <- row.names(HDL_paed_dom_tab2)

HDL_paed_dom_tab2$model <- "Dominant"

HDL_paed_dom_tab2$outcome <- "HDL"


HDL_paed_rec_tab <- data.frame(HDL_paed_rec_reg_ethnic[["TE.random"]])

row.names(HDL_paed_rec_tab) <- "Overall"

HDL_paed_rec_tab$MD <- HDL_paed_rec_tab$HDL_paed_rec_reg_ethnic...TE.random...

HDL_paed_rec_tab <- HDL_paed_rec_tab[-c(1)]

HDL_paed_rec_tab$lower <- HDL_paed_rec_reg_ethnic[["lower.random"]]

HDL_paed_rec_tab$upper <- HDL_paed_rec_reg_ethnic[["upper.random"]]
```

```
HDL_paed_rec_tab$k <- HDL_paed_rec_reg_ethnic[["k"]]

HDL_paed_rec_tab$p_z <- HDL_paed_rec_reg_ethnic[["pval.random"]]

HDL_paed_rec_tab$I2 <- HDL_paed_rec_reg_ethnic[["I2"]]

HDL_paed_rec_tab$p_q <- HDL_paed_rec_reg_ethnic[["pval.Q"]]

HDL_paed_rec_tab$group <- "Overall"

HDL_paed_rec_tab$model <- "Recessive"

HDL_paed_rec_tab$outcome <- "HDL"


HDL_paed_rec_tab2 <- data.frame(HDL_paed_rec_reg_ethnic[["TE.random.w"]])

row.names(HDL_paed_rec_tab2) <- HDL_paed_rec_reg_ethnic[["bylevs"]]

HDL_paed_rec_tab2$MD <- HDL_paed_rec_tab2$HDL_paed_rec_reg_ethnic...TE.random.w...

HDL_paed_rec_tab2 <- HDL_paed_rec_tab2[-c(1)]

HDL_paed_rec_tab2$lower <- HDL_paed_rec_reg_ethnic[["lower.random.w"]]

HDL_paed_rec_tab2$upper <- HDL_paed_rec_reg_ethnic[["upper.random.w"]]

HDL_paed_rec_tab2$k <- HDL_paed_rec_reg_ethnic[["k.w"]]

HDL_paed_rec_tab2$p_z <- HDL_paed_rec_reg_ethnic[["pval.random.w"]]

HDL_paed_rec_tab2$I2 <- HDL_paed_rec_reg_ethnic[["I2.w"]]

HDL_paed_rec_tab2$p_q <- HDL_paed_rec_reg_ethnic[["pval.Q.w"]]
```

```
HDL_paed_rec_tab2$group <- row.names(HDL_paed_rec_tab2)

HDL_paed_rec_tab2$model <- "Recessive"

HDL_paed_rec_tab2$outcome <- "HDL"


HDL_paed_sumtab <- rbind(HDL_paed_add_tab, HDL_paed_add_tab2, HDL_paed_dom_tab, HDL_paed_dom_tab2,
HDL_paed_rec_tab, HDL_paed_rec_tab2)


LDL_adult <- read_excel("LDL_adult.xlsx")


LDL_adult$LDL_CCCT_num <- LDL_adult$LDL_CC_num+LDL_adult$LDL_CT_num

LDL_adult$LDL_CCCT_mean <-
((LDL_adult$LDL_CC_mean*LDL_adult$LDL_CC_num)+(LDL_adult$LDL_CT_mean*LDL_adult$LDL_CT_num))/LDL_adult$LDL_
CCCT_num
```

```
LDL_adult$LDL_CCCT_SD <-
((LDL_adult$LDL_CC_SD*LDL_adult$LDL_CC_num)+(LDL_adult$LDL_CT_SD*LDL_adult$LDL_CT_num))/LDL_adult$LDL_CCC
T_num
LDL_adult$LDL_CTTT_num <- LDL_adult$LDL_TT_num+LDL_adult$LDL_CT_num
LDL_adult$LDL_CTTT_mean <-
((LDL_adult$LDL_TT_mean*LDL_adult$LDL_TT_num)+(LDL_adult$LDL_CT_mean*LDL_adult$LDL_CT_num))/LDL_adult$LDL_C
TTT_num
LDL_adult$LDL_CTTT_SD <-
((LDL_adult$LDL_TT_SD*LDL_adult$LDL_TT_num)+(LDL_adult$LDL_CT_SD*LDL_adult$LDL_CT_num))/LDL_adult$LDL_CTTT
_num

LDL_adult_add <- read_excel("LDL_adult_add.xlsx")
LDL_adult_add_reg <- metagen(Beta, SE, data = LDL_adult_add, studlab = LDL_adult_add$Paper, sm = "ZCOR", method.tau =
"DL")
LDL_adult_add_reg_ethnic <- update(LDL_adult_add_reg, byvar = Ethnicity, bylab = "Ethnicity")
```

```
LDL_adult_rec_reg <- metacont(LDL_TT_num, LDL_TT_mean, LDL_TT_SD, LDL_CCCT_num, LDL_CCCT_mean,
LDL_CCCT_SD, data = LDL_adult, studlab = paste(Paper), comb.fixed = FALSE, comb.random = TRUE, method.tau = "DL", hakn
= TRUE, prediction = FALSE, sm = "MD")
LDL_adult_rec_reg_ethnic <- update(LDL_adult_rec_reg, byvar = Ethnicity, bylab = "Ethnicity")

LDL_adult_dom_reg <- metacont(LDL_CTTT_num, LDL_CTTT_mean, LDL_CTTT_SD, LDL_CC_num, LDL_CC_mean,
LDL_CC_SD, data = LDL_adult, studlab = paste(Paper), comb.fixed = FALSE, comb.random = TRUE, method.tau = "DL", hakn =
TRUE, prediction = FALSE, sm = "MD")
LDL_adult_dom_reg_ethnic <- update(LDL_adult_dom_reg, byvar = Ethnicity, bylab = "Ethnicity")

LDL_adult_add_tab <- data.frame(LDL_adult_add_reg_ethnic[["TE.random"]])
row.names(LDL_adult_add_tab) <- "Overall"
LDL_adult_add_tab$MD <- LDL_adult_add_tab$LDL_adult_add_reg_ethnic...TE.random...
LDL_adult_add_tab <- LDL_adult_add_tab[-c(1)]
LDL_adult_add_tab$lower <- LDL_adult_add_reg_ethnic[["lower.random"]]
LDL_adult_add_tab$upper <- LDL_adult_add_reg_ethnic[["upper.random"]]
LDL_adult_add_tab$k <- LDL_adult_add_reg_ethnic[["k"]]
LDL_adult_add_tab$p_z <- LDL_adult_add_reg_ethnic[["pval.random"]]
```

```
LDL_adult_add_tab$I2 <- LDL_adult_add_reg_ethnic[["I2"]]

LDL_adult_add_tab$p_q <- LDL_adult_add_reg_ethnic[["pval.Q"]]

LDL_adult_add_tab$group <- "Overall"

LDL_adult_add_tab$model <- "Additive"

LDL_adult_add_tab$outcome <- "LDL"


LDL_adult_add_tab2 <- data.frame(LDL_adult_add_reg_ethnic[["TE.random.w"]])

row.names(LDL_adult_add_tab2) <- LDL_adult_add_reg_ethnic[["bylevs"]]

LDL_adult_add_tab2$MD <- LDL_adult_add_tab2$LDL_adult_add_reg_ethnic...TE.random.w...

LDL_adult_add_tab2 <- LDL_adult_add_tab2[-c(1)]

LDL_adult_add_tab2$lower <- LDL_adult_add_reg_ethnic[["lower.random.w"]]

LDL_adult_add_tab2$upper <- LDL_adult_add_reg_ethnic[["upper.random.w"]]

LDL_adult_add_tab2$k <- LDL_adult_add_reg_ethnic[["k.w"]]

LDL_adult_add_tab2$p_z <- LDL_adult_add_reg_ethnic[["pval.random.w"]]

LDL_adult_add_tab2$I2 <- LDL_adult_add_reg_ethnic[["I2.w"]]

LDL_adult_add_tab2$p_q <- LDL_adult_add_reg_ethnic[["pval.Q.w"]]

LDL_adult_add_tab2$group <- row.names(LDL_adult_add_tab2)

LDL_adult_add_tab2$model <- "Additive"
```

```
LDL_adult_add_tab2$outcome <- "LDL"


LDL_adult_dom_tab <- data.frame(LDL_adult_dom_reg_ethnic[["TE.random"]])

row.names(LDL_adult_dom_tab) <- "Overall"

LDL_adult_dom_tab$MD <- LDL_adult_dom_tab$LDL_adult_dom_reg_ethnic...TE.random...

LDL_adult_dom_tab <- LDL_adult_dom_tab[-c(1)]

LDL_adult_dom_tab$lower <- LDL_adult_dom_reg_ethnic[["lower.random"]]

LDL_adult_dom_tab$upper <- LDL_adult_dom_reg_ethnic[["upper.random"]]

LDL_adult_dom_tab$k <- LDL_adult_dom_reg_ethnic[["k"]]

LDL_adult_dom_tab$p_z <- LDL_adult_dom_reg_ethnic[["pval.random"]]

LDL_adult_dom_tab$I2 <- LDL_adult_dom_reg_ethnic[["I2"]]

LDL_adult_dom_tab$p_q <- LDL_adult_dom_reg_ethnic[["pval.Q"]]

LDL_adult_dom_tab$group <- "Overall"

LDL_adult_dom_tab$model <- "Dominant"

LDL_adult_dom_tab$outcome <- "LDL"


LDL_adult_dom_tab2 <- data.frame(LDL_adult_dom_reg_ethnic[["TE.random.w"]])

row.names(LDL_adult_dom_tab2) <- LDL_adult_dom_reg_ethnic[["bylevs"]]
```

```
LDL_adult_dom_tab2$MD <- LDL_adult_dom_tab2$LDL_adult_dom_reg_ethnic...TE.random.w...

LDL_adult_dom_tab2 <- LDL_adult_dom_tab2[-c(1)]

LDL_adult_dom_tab2$lower <- LDL_adult_dom_reg_ethnic[["lower.random.w"]]

LDL_adult_dom_tab2$upper <- LDL_adult_dom_reg_ethnic[["upper.random.w"]]

LDL_adult_dom_tab2$k <- LDL_adult_dom_reg_ethnic[["k.w"]]

LDL_adult_dom_tab2$p_z <- LDL_adult_dom_reg_ethnic[["pval.random.w"]]

LDL_adult_dom_tab2$I2 <- LDL_adult_dom_reg_ethnic[["I2.w"]]

LDL_adult_dom_tab2$p_q <- LDL_adult_dom_reg_ethnic[["pval.Q.w"]]

LDL_adult_dom_tab2$group <- row.names(LDL_adult_dom_tab2)

LDL_adult_dom_tab2$model <- "Dominant"

LDL_adult_dom_tab2$outcome <- "LDL"


LDL_adult_rec_tab <- data.frame(LDL_adult_rec_reg_ethnic[["TE.random"]])

row.names(LDL_adult_rec_tab) <- "Overall"

LDL_adult_rec_tab$MD <- LDL_adult_rec_tab$LDL_adult_rec_reg_ethnic...TE.random...

LDL_adult_rec_tab <- LDL_adult_rec_tab[-c(1)]

LDL_adult_rec_tab$lower <- LDL_adult_rec_reg_ethnic[["lower.random"]]

LDL_adult_rec_tab$upper <- LDL_adult_rec_reg_ethnic[["upper.random"]]
```

```
LDL_adult_rec_tab$k <- LDL_adult_rec_reg_ethnic[["k"]]

LDL_adult_rec_tab$p_z <- LDL_adult_rec_reg_ethnic[["pval.random"]]

LDL_adult_rec_tab$I2 <- LDL_adult_rec_reg_ethnic[["I2"]]

LDL_adult_rec_tab$p_q <- LDL_adult_rec_reg_ethnic[["pval.Q"]]

LDL_adult_rec_tab$group <- "Overall"

LDL_adult_rec_tab$model <- "Recessive"

LDL_adult_rec_tab$outcome <- "LDL"


LDL_adult_rec_tab2 <- data.frame(LDL_adult_rec_reg_ethnic[["TE.random.w"]])

row.names(LDL_adult_rec_tab2) <- LDL_adult_rec_reg_ethnic[["bylevs"]]

LDL_adult_rec_tab2$MD <- LDL_adult_rec_tab2$LDL_adult_rec_reg_ethnic...TE.random.w...

LDL_adult_rec_tab2 <- LDL_adult_rec_tab2[-c(1)]

LDL_adult_rec_tab2$lower <- LDL_adult_rec_reg_ethnic[["lower.random.w"]]

LDL_adult_rec_tab2$upper <- LDL_adult_rec_reg_ethnic[["upper.random.w"]]

LDL_adult_rec_tab2$k <- LDL_adult_rec_reg_ethnic[["k.w"]]

LDL_adult_rec_tab2$p_z <- LDL_adult_rec_reg_ethnic[["pval.random.w"]]

LDL_adult_rec_tab2$I2 <- LDL_adult_rec_reg_ethnic[["I2.w"]]

LDL_adult_rec_tab2$p_q <- LDL_adult_rec_reg_ethnic[["pval.Q.w"]]
```

```
LDL_adult_rec_tab2$group <- row.names(LDL_adult_rec_tab2)

LDL_adult_rec_tab2$model <- "Recessive"

LDL_adult_rec_tab2$outcome <- "LDL"


LDL_adult_sumtab <- rbind(LDL_adult_add_tab, LDL_adult_add_tab2, LDL_adult_dom_tab, LDL_adult_dom_tab2,
LDL_adult_rec_tab, LDL_adult_rec_tab2)


LDL_paed <- read_excel("LDL_paed.xlsx")


LDL_paed$LDL_CCCT_num <- LDL_paed$LDL_CC_num+LDL_paed$LDL_CT_num

LDL_paed$LDL_CCCT_mean <-
((LDL_paed$LDL_CC_mean*LDL_paed$LDL_CC_num)+(LDL_paed$LDL_CT_mean*LDL_paed$LDL_CT_num))/LDL_paed$LDL_
CCCT_num
```

```
LDL_paed$LDL_CCCT_SD <-
((LDL_paed$LDL_CC_SD*LDL_paed$LDL_CC_num)+(LDL_paed$LDL_CT_SD*LDL_paed$LDL_CT_num))/LDL_paed$LDL_CCC
T_num
LDL_paed$LDL_CTTT_num <- LDL_paed$LDL_TT_num+LDL_paed$LDL_CT_num
LDL_paed$LDL_CTTT_mean <-
((LDL_paed$LDL_TT_mean*LDL_paed$LDL_TT_num)+(LDL_paed$LDL_CT_mean*LDL_paed$LDL_CT_num))/LDL_paed$LDL_
CTTT_num
LDL_paed$LDL_CTTT_SD <-
((LDL_paed$LDL_TT_SD*LDL_paed$LDL_TT_num)+(LDL_paed$LDL_CT_SD*LDL_paed$LDL_CT_num))/LDL_paed$LDL_CTTT
_num

LDL_paed_add <- read_excel("LDL_paed_add.xlsx")
LDL_paed_add_reg <- metagen(Beta, SE, data = LDL_paed_add, studlab = LDL_paed_add$Paper, sm = "ZCOR", method.tau =
"DL")
LDL_paed_add_reg_ethnic <- update(LDL_paed_add_reg, byvar = Ethnicity, bylab = "Ethnicity")
```

```
LDL_paed_rec_reg <- metacont(LDL_TT_num, LDL_TT_mean, LDL_TT_SD, LDL_CCCT_num, LDL_CCCT_mean,
LDL_CCCT_SD, data = LDL_paed, studlab = paste(Paper), comb.fixed = FALSE, comb.random = TRUE, method.tau = "DL", hakn
= TRUE, prediction = FALSE, sm = "MD")
LDL_paed_rec_reg_ethnic <- update(LDL_paed_rec_reg, byvar = Ethnicity, bylab = "Ethnicity")

LDL_paed_dom_reg <- metacont(LDL_CTTT_num, LDL_CTTT_mean, LDL_CTTT_SD, LDL_CC_num, LDL_CC_mean,
LDL_CC_SD, data = LDL_paed, studlab = paste(Paper), comb.fixed = FALSE, comb.random = TRUE, method.tau = "DL", hakn =
TRUE, prediction = FALSE, sm = "MD")
LDL_paed_dom_reg_ethnic <- update(LDL_paed_dom_reg, byvar = Ethnicity, bylab = "Ethnicity")

LDL_paed_add_tab <- data.frame(LDL_paed_add_reg_ethnic[["TE.random"]])
row.names(LDL_paed_add_tab) <- "Overall"
LDL_paed_add_tab$MD <- LDL_paed_add_tab$LDL_paed_add_reg_ethnic...TE.random...
LDL_paed_add_tab <- LDL_paed_add_tab[-c(1)]
LDL_paed_add_tab$lower <- LDL_paed_add_reg_ethnic[["lower.random"]]
LDL_paed_add_tab$upper <- LDL_paed_add_reg_ethnic[["upper.random"]]
LDL_paed_add_tab$k <- LDL_paed_add_reg_ethnic[["k"]]
LDL_paed_add_tab$p_z <- LDL_paed_add_reg_ethnic[["pval.random"]]
```

```
LDL_paed_add_tab$I2 <- LDL_paed_add_reg_ethnic[["I2"]]

LDL_paed_add_tab$p_q <- LDL_paed_add_reg_ethnic[["pval.Q"]]

LDL_paed_add_tab$group <- "Overall"

LDL_paed_add_tab$model <- "Additive"

LDL_paed_add_tab$outcome <- "LDL"


LDL_paed_add_tab2 <- data.frame(LDL_paed_add_reg_ethnic[["TE.random.w"]])

row.names(LDL_paed_add_tab2) <- LDL_paed_add_reg_ethnic[["bylevs"]]

LDL_paed_add_tab2$MD <- LDL_paed_add_tab2$LDL_paed_add_reg_ethnic...TE.random.w...

LDL_paed_add_tab2 <- LDL_paed_add_tab2[-c(1)]

LDL_paed_add_tab2$lower <- LDL_paed_add_reg_ethnic[["lower.random.w"]]

LDL_paed_add_tab2$upper <- LDL_paed_add_reg_ethnic[["upper.random.w"]]

LDL_paed_add_tab2$k <- LDL_paed_add_reg_ethnic[["k.w"]]

LDL_paed_add_tab2$p_z <- LDL_paed_add_reg_ethnic[["pval.random.w"]]

LDL_paed_add_tab2$I2 <- LDL_paed_add_reg_ethnic[["I2.w"]]

LDL_paed_add_tab2$p_q <- LDL_paed_add_reg_ethnic[["pval.Q.w"]]

LDL_paed_add_tab2$group <- row.names(LDL_paed_add_tab2)

LDL_paed_add_tab2$model <- "Additive"
```

```
LDL_paed_add_tab2$outcome <- "LDL"


LDL_paed_dom_tab <- data.frame(LDL_paed_dom_reg_ethnic[["TE.random"]])

row.names(LDL_paed_dom_tab) <- "Overall"

LDL_paed_dom_tab$MD <- LDL_paed_dom_tab$LDL_paed_dom_reg_ethnic...TE.random...

LDL_paed_dom_tab <- LDL_paed_dom_tab[-c(1)]

LDL_paed_dom_tab$lower <- LDL_paed_dom_reg_ethnic[["lower.random"]]

LDL_paed_dom_tab$upper <- LDL_paed_dom_reg_ethnic[["upper.random"]]

LDL_paed_dom_tab$k <- LDL_paed_dom_reg_ethnic[["k"]]

LDL_paed_dom_tab$p_z <- LDL_paed_dom_reg_ethnic[["pval.random"]]

LDL_paed_dom_tab$I2 <- LDL_paed_dom_reg_ethnic[["I2"]]

LDL_paed_dom_tab$p_q <- LDL_paed_dom_reg_ethnic[["pval.Q"]]

LDL_paed_dom_tab$group <- "Overall"

LDL_paed_dom_tab$model <- "Dominant"

LDL_paed_dom_tab$outcome <- "LDL"


LDL_paed_dom_tab2 <- data.frame(LDL_paed_dom_reg_ethnic[["TE.random.w"]])

row.names(LDL_paed_dom_tab2) <- LDL_paed_dom_reg_ethnic[["bylevs"]]
```

```
LDL_paed_dom_tab2$MD <- LDL_paed_dom_tab2$LDL_paed_dom_reg_ethnic...TE.random.w...

LDL_paed_dom_tab2 <- LDL_paed_dom_tab2[-c(1)]

LDL_paed_dom_tab2$lower <- LDL_paed_dom_reg_ethnic[["lower.random.w"]]

LDL_paed_dom_tab2$upper <- LDL_paed_dom_reg_ethnic[["upper.random.w"]]

LDL_paed_dom_tab2$k <- LDL_paed_dom_reg_ethnic[["k.w"]]

LDL_paed_dom_tab2$p_z <- LDL_paed_dom_reg_ethnic[["pval.random.w"]]

LDL_paed_dom_tab2$I2 <- LDL_paed_dom_reg_ethnic[["I2.w"]]

LDL_paed_dom_tab2$p_q <- LDL_paed_dom_reg_ethnic[["pval.Q.w"]]

LDL_paed_dom_tab2$group <- row.names(LDL_paed_dom_tab2)

LDL_paed_dom_tab2$model <- "Dominant"

LDL_paed_dom_tab2$outcome <- "LDL"


LDL_paed_rec_tab <- data.frame(LDL_paed_rec_reg_ethnic[["TE.random"]])

row.names(LDL_paed_rec_tab) <- "Overall"

LDL_paed_rec_tab$MD <- LDL_paed_rec_tab$LDL_paed_rec_reg_ethnic...TE.random...

LDL_paed_rec_tab <- LDL_paed_rec_tab[-c(1)]

LDL_paed_rec_tab$lower <- LDL_paed_rec_reg_ethnic[["lower.random"]]

LDL_paed_rec_tab$upper <- LDL_paed_rec_reg_ethnic[["upper.random"]]
```

```
LDL_paed_rec_tab$k <- LDL_paed_rec_reg_ethnic[["k"]]

LDL_paed_rec_tab$p_z <- LDL_paed_rec_reg_ethnic[["pval.random"]]

LDL_paed_rec_tab$I2 <- LDL_paed_rec_reg_ethnic[["I2"]]

LDL_paed_rec_tab$p_q <- LDL_paed_rec_reg_ethnic[["pval.Q"]]

LDL_paed_rec_tab$group <- "Overall"

LDL_paed_rec_tab$model <- "Recessive"

LDL_paed_rec_tab$outcome <- "LDL"


LDL_paed_rec_tab2 <- data.frame(LDL_paed_rec_reg_ethnic[["TE.random.w"]])

row.names(LDL_paed_rec_tab2) <- LDL_paed_rec_reg_ethnic[["bylevs"]]

LDL_paed_rec_tab2$MD <- LDL_paed_rec_tab2$LDL_paed_rec_reg_ethnic...TE.random.w...

LDL_paed_rec_tab2 <- LDL_paed_rec_tab2[-c(1)]

LDL_paed_rec_tab2$lower <- LDL_paed_rec_reg_ethnic[["lower.random.w"]]

LDL_paed_rec_tab2$upper <- LDL_paed_rec_reg_ethnic[["upper.random.w"]]

LDL_paed_rec_tab2$k <- LDL_paed_rec_reg_ethnic[["k.w"]]

LDL_paed_rec_tab2$p_z <- LDL_paed_rec_reg_ethnic[["pval.random.w"]]

LDL_paed_rec_tab2$I2 <- LDL_paed_rec_reg_ethnic[["I2.w"]]

LDL_paed_rec_tab2$p_q <- LDL_paed_rec_reg_ethnic[["pval.Q.w"]]
```

```
LDL_paed_rec_tab2$group <- row.names(LDL_paed_rec_tab2)

LDL_paed_rec_tab2$model <- "Recessive"

LDL_paed_rec_tab2$outcome <- "LDL"


LDL_paed_sumtab <- rbind(LDL_paed_add_tab, LDL_paed_add_tab2, LDL_paed_dom_tab, LDL_paed_dom_tab2,
LDL_paed_rec_tab, LDL_paed_rec_tab2)


Insul_adult <- read_excel("Insul_adult.xlsx")


Insul_adult$Insul_CCCT_num <- Insul_adult$Insul_CC_num+Insul_adult$Insul_CT_num

Insul_adult$Insul_CCCT_mean <-
((Insul_adult$Insul_CC_mean*Insul_adult$Insul_CC_num)+(Insul_adult$Insul_CT_mean*Insul_adult$Insul_CT_num))/Insul_adult$
Insul_CCCT_num
```

```
Insul_adult$Insul_CCCT_SD <-
((Insul_adult$Insul_CC_SD*Insul_adult$Insul_CC_num)+(Insul_adult$Insul_CT_SD*Insul_adult$Insul_CT_num))/Insul_adult$Insul
_CCCT_num
Insul_adult$Insul_CTTT_num <- Insul_adult$Insul_TT_num+Insul_adult$Insul_CT_num
Insul_adult$Insul_CTTT_mean <-
((Insul_adult$Insul_TT_mean*Insul_adult$Insul_TT_num)+(Insul_adult$Insul_CT_mean*Insul_adult$Insul_CT_num))/Insul_adult$I
nsul_CTTT_num
Insul_adult$Insul_CTTT_SD <-
((Insul_adult$Insul_TT_SD*Insul_adult$Insul_TT_num)+(Insul_adult$Insul_CT_SD*Insul_adult$Insul_CT_num))/Insul_adult$Insul_
CTTT_num

Insul_adult_add <- read_excel("Insul_adult_add.xlsx")
Insul_adult_add_reg <- metagen(Beta, SE, data = Insul_adult_add, studlab = Insul_adult_add$Paper, sm = "ZCOR", method.tau =
"DL")
Insul_adult_add_reg_ethnic <- update(Insul_adult_add_reg, byvar = Ethnicity, bylab = "Ethnicity")
```

```
Insul_adult_rec_reg <- metacont(Insul_TT_num, Insul_TT_mean, Insul_TT_SD, Insul_CCCT_num, Insul_CCCT_mean,
Insul_CCCT_SD, data = Insul_adult, studlab = paste(Paper), comb.fixed = FALSE, comb.random = TRUE, method.tau = "DL",
hakn = TRUE, prediction = FALSE, sm = "MD")
Insul_adult_rec_reg_ethnic <- update(Insul_adult_rec_reg, byvar = Ethnicity, bylab = "Ethnicity")

Insul_adult_dom_reg <- metacont(Insul_CTTT_num, Insul_CTTT_mean, Insul_CTTT_SD, Insul_CC_num, Insul_CC_mean,
Insul_CC_SD, data = Insul_adult, studlab = paste(Paper), comb.fixed = FALSE, comb.random = TRUE, method.tau = "DL", hakn =
TRUE, prediction = FALSE, sm = "MD")
Insul_adult_dom_reg_ethnic <- update(Insul_adult_dom_reg, byvar = Ethnicity, bylab = "Ethnicity")

Insul_adult_add_tab <- data.frame(Insul_adult_add_reg_ethnic[["TE.random"]])
row.names(Insul_adult_add_tab) <- "Overall"
Insul_adult_add_tab$MD <- Insul_adult_add_tab$Insul_adult_add_reg_ethnic...TE.random...
Insul_adult_add_tab <- Insul_adult_add_tab[-c(1)]
Insul_adult_add_tab$lower <- Insul_adult_add_reg_ethnic[["lower.random"]]
Insul_adult_add_tab$upper <- Insul_adult_add_reg_ethnic[["upper.random"]]
Insul_adult_add_tab$k <- Insul_adult_add_reg_ethnic[["k"]]
Insul_adult_add_tab$p_z <- Insul_adult_add_reg_ethnic[["pval.random"]]
```

```
Insul_adult_add_tab$I2 <- Insul_adult_add_reg_ethnic[["I2"]]

Insul_adult_add_tab$p_q <- Insul_adult_add_reg_ethnic[["pval.Q"]]

Insul_adult_add_tab$group <- "Overall"

Insul_adult_add_tab$model <- "Additive"

Insul_adult_add_tab$outcome <- "Insul"


Insul_adult_add_tab2 <- data.frame(Insul_adult_add_reg_ethnic[["TE.random.w"]])

row.names(Insul_adult_add_tab2) <- Insul_adult_add_reg_ethnic[["bylevs"]]

Insul_adult_add_tab2$MD <- Insul_adult_add_tab2$Insul_adult_add_reg_ethnic...TE.random.w...

Insul_adult_add_tab2 <- Insul_adult_add_tab2[-c(1)]

Insul_adult_add_tab2$lower <- Insul_adult_add_reg_ethnic[["lower.random.w"]]

Insul_adult_add_tab2$upper <- Insul_adult_add_reg_ethnic[["upper.random.w"]]

Insul_adult_add_tab2$k <- Insul_adult_add_reg_ethnic[["k.w"]]

Insul_adult_add_tab2$p_z <- Insul_adult_add_reg_ethnic[["pval.random.w"]]

Insul_adult_add_tab2$I2 <- Insul_adult_add_reg_ethnic[["I2.w"]]

Insul_adult_add_tab2$p_q <- Insul_adult_add_reg_ethnic[["pval.Q.w"]]

Insul_adult_add_tab2$group <- row.names(Insul_adult_add_tab2)

Insul_adult_add_tab2$model <- "Additive"
```

```
Insul_adult_add_tab2$outcome <- "Insul"

Insul_adult_dom_tab <- data.frame(Insul_adult_dom_reg_ethnic[["TE.random"]])
row.names(Insul_adult_dom_tab) <- "Overall"
Insul_adult_dom_tab$MD <- Insul_adult_dom_tab$Insul_adult_dom_reg_ethnic...TE.random...
Insul_adult_dom_tab <- Insul_adult_dom_tab[-c(1)]
Insul_adult_dom_tab$lower <- Insul_adult_dom_reg_ethnic[["lower.random"]]
Insul_adult_dom_tab$upper <- Insul_adult_dom_reg_ethnic[["upper.random"]]
Insul_adult_dom_tab$k <- Insul_adult_dom_reg_ethnic[["k"]]
Insul_adult_dom_tab$p_z <- Insul_adult_dom_reg_ethnic[["pval.random"]]
Insul_adult_dom_tab$I2 <- Insul_adult_dom_reg_ethnic[["I2"]]
Insul_adult_dom_tab$p_q <- Insul_adult_dom_reg_ethnic[["pval.Q"]]
Insul_adult_dom_tab$group <- "Overall"
Insul_adult_dom_tab$model <- "Dominant"
Insul_adult_dom_tab$outcome <- "Insul"

Insul_adult_dom_tab2 <- data.frame(Insul_adult_dom_reg_ethnic[["TE.random.w"]])
row.names(Insul_adult_dom_tab2) <- Insul_adult_dom_reg_ethnic[["bylevs"]]
```

```
Insul_adult_dom_tab2$MD <- Insul_adult_dom_tab2$Insul_adult_dom_reg_ethnic...TE.random.w...

Insul_adult_dom_tab2 <- Insul_adult_dom_tab2[-c(1)]

Insul_adult_dom_tab2$lower <- Insul_adult_dom_reg_ethnic[["lower.random.w"]]

Insul_adult_dom_tab2$upper <- Insul_adult_dom_reg_ethnic[["upper.random.w"]]

Insul_adult_dom_tab2$k <- Insul_adult_dom_reg_ethnic[["k.w"]]

Insul_adult_dom_tab2$p_z <- Insul_adult_dom_reg_ethnic[["pval.random.w"]]

Insul_adult_dom_tab2$I2 <- Insul_adult_dom_reg_ethnic[["I2.w"]]

Insul_adult_dom_tab2$p_q <- Insul_adult_dom_reg_ethnic[["pval.Q.w"]]

Insul_adult_dom_tab2$group <- row.names(Insul_adult_dom_tab2)

Insul_adult_dom_tab2$model <- "Dominant"

Insul_adult_dom_tab2$outcome <- "Insul"


Insul_adult_rec_tab <- data.frame(Insul_adult_rec_reg_ethnic[["TE.random"]])

row.names(Insul_adult_rec_tab) <- "Overall"

Insul_adult_rec_tab$MD <- Insul_adult_rec_tab$Insul_adult_rec_reg_ethnic...TE.random...

Insul_adult_rec_tab <- Insul_adult_rec_tab[-c(1)]

Insul_adult_rec_tab$lower <- Insul_adult_rec_reg_ethnic[["lower.random"]]

Insul_adult_rec_tab$upper <- Insul_adult_rec_reg_ethnic[["upper.random"]]
```

```
Insul_adult_rec_tab$k <- Insul_adult_rec_reg_ethnic[["k"]]

Insul_adult_rec_tab$p_z <- Insul_adult_rec_reg_ethnic[["pval.random"]]

Insul_adult_rec_tab$I2 <- Insul_adult_rec_reg_ethnic[["I2"]]

Insul_adult_rec_tab$p_q <- Insul_adult_rec_reg_ethnic[["pval.Q"]]

Insul_adult_rec_tab$group <- "Overall"

Insul_adult_rec_tab$model <- "Recessive"

Insul_adult_rec_tab$outcome <- "Insul"


Insul_adult_rec_tab2 <- data.frame(Insul_adult_rec_reg_ethnic[["TE.random.w"]])

row.names(Insul_adult_rec_tab2) <- Insul_adult_rec_reg_ethnic[["bylevs"]]

Insul_adult_rec_tab2$MD <- Insul_adult_rec_tab2$Insul_adult_rec_reg_ethnic...TE.random.w...

Insul_adult_rec_tab2 <- Insul_adult_rec_tab2[-c(1)]

Insul_adult_rec_tab2$lower <- Insul_adult_rec_reg_ethnic[["lower.random.w"]]

Insul_adult_rec_tab2$upper <- Insul_adult_rec_reg_ethnic[["upper.random.w"]]

Insul_adult_rec_tab2$k <- Insul_adult_rec_reg_ethnic[["k.w"]]

Insul_adult_rec_tab2$p_z <- Insul_adult_rec_reg_ethnic[["pval.random.w"]]

Insul_adult_rec_tab2$I2 <- Insul_adult_rec_reg_ethnic[["I2.w"]]

Insul_adult_rec_tab2$p_q <- Insul_adult_rec_reg_ethnic[["pval.Q.w"]]
```

```
Insul_adult_rec_tab2$group <- row.names(Insul_adult_rec_tab2)

Insul_adult_rec_tab2$model <- "Recessive"

Insul_adult_rec_tab2$outcome <- "Insul"


Insul_adult_sumtab <- rbind(Insul_adult_add_tab, Insul_adult_add_tab2, Insul_adult_dom_tab, Insul_adult_dom_tab2,

Insul_adult_rec_tab, Insul_adult_rec_tab2)


Insul_paed <- read_excel("Insul_paed.xlsx")


Insul_paed$Insul_CCCT_num <- Insul_paed$Insul_CC_num+Insul_paed$Insul_CT_num

Insul_paed$Insul_CCCT_mean <-

((Insul_paed$Insul_CC_mean*Insul_paed$Insul_CC_num)+(Insul_paed$Insul_CT_mean*Insul_paed$Insul_CT_num))/Insul_paed

$Insul_CCCT_num
```

```
Insul_paed$Insul_CCCT_SD <-
((Insul_paed$Insul_CC_SD*Insul_paed$Insul_CC_num)+(Insul_paed$Insul_CT_SD*Insul_paed$Insul_CT_num))/Insul_paed$Insul
_CCCT_num
Insul_paed$Insul_CTTT_num <- Insul_paed$Insul_TT_num+Insul_paed$Insul_CT_num
Insul_paed$Insul_CTTT_mean <-
((Insul_paed$Insul_TT_mean*Insul_paed$Insul_TT_num)+(Insul_paed$Insul_CT_mean*Insul_paed$Insul_CT_num))/Insul_paed$I
nsul_CTTT_num
Insul_paed$Insul_CTTT_SD <-
((Insul_paed$Insul_TT_SD*Insul_paed$Insul_TT_num)+(Insul_paed$Insul_CT_SD*Insul_paed$Insul_CT_num))/Insul_paed$Insul
_CTTT_num

Insul_paed_add <- read_excel("Insul_paed_add.xlsx")
Insul_paed_add_reg <- metagen(Beta, SE, data = Insul_paed_add, studlab = Insul_paed_add$Paper, sm = "ZCOR", method.tau =
"DL")
Insul_paed_add_reg_ethnic <- update(Insul_paed_add_reg, byvar = Ethnicity, bylab = "Ethnicity")
```

```
Insul_paed_rec_reg <- metacont(Insul_TT_num, Insul_TT_mean, Insul_TT_SD, Insul_CCCT_num, Insul_CCCT_mean,
Insul_CCCT_SD, data = Insul_paed, studlab = paste(Paper), comb.fixed = FALSE, comb.random = TRUE, method.tau = "DL",
hakn = TRUE, prediction = FALSE, sm = "MD")
Insul_paed_rec_reg_ethnic <- update(Insul_paed_rec_reg, byvar = Ethnicity, bylab = "Ethnicity")

Insul_paed_dom_reg <- metacont(Insul_CTTT_num, Insul_CTTT_mean, Insul_CTTT_SD, Insul_CC_num, Insul_CC_mean,
Insul_CC_SD, data = Insul_paed, studlab = paste(Paper), comb.fixed = FALSE, comb.random = TRUE, method.tau = "DL", hakn =
TRUE, prediction = FALSE, sm = "MD")
Insul_paed_dom_reg_ethnic <- update(Insul_paed_dom_reg, byvar = Ethnicity, bylab = "Ethnicity")

Insul_paed_add_tab <- data.frame(Insul_paed_add_reg_ethnic[["TE.random"]])
row.names(Insul_paed_add_tab) <- "Overall"
Insul_paed_add_tab$MD <- Insul_paed_add_tab$Insul_paed_add_reg_ethnic...TE.random...
Insul_paed_add_tab <- Insul_paed_add_tab[-c(1)]
Insul_paed_add_tab$lower <- Insul_paed_add_reg_ethnic[["lower.random"]]
Insul_paed_add_tab$upper <- Insul_paed_add_reg_ethnic[["upper.random"]]
Insul_paed_add_tab$k <- Insul_paed_add_reg_ethnic[["k"]]
Insul_paed_add_tab$p_z <- Insul_paed_add_reg_ethnic[["pval.random"]]
```

```
Insul_paed_add_tab$I2 <- Insul_paed_add_reg_ethnic[["I2"]]

Insul_paed_add_tab$p_q <- Insul_paed_add_reg_ethnic[["pval.Q"]]

Insul_paed_add_tab$group <- "Overall"

Insul_paed_add_tab$model <- "Additive"

Insul_paed_add_tab$outcome <- "Insul"


Insul_paed_add_tab2 <- data.frame(Insul_paed_add_reg_ethnic[["TE.random.w"]])

row.names(Insul_paed_add_tab2) <- Insul_paed_add_reg_ethnic[["bylevs"]]

Insul_paed_add_tab2$MD <- Insul_paed_add_tab2$Insul_paed_add_reg_ethnic...TE.random.w...

Insul_paed_add_tab2 <- Insul_paed_add_tab2[-c(1)]

Insul_paed_add_tab2$lower <- Insul_paed_add_reg_ethnic[["lower.random.w"]]

Insul_paed_add_tab2$upper <- Insul_paed_add_reg_ethnic[["upper.random.w"]]

Insul_paed_add_tab2$k <- Insul_paed_add_reg_ethnic[["k.w"]]

Insul_paed_add_tab2$p_z <- Insul_paed_add_reg_ethnic[["pval.random.w"]]

Insul_paed_add_tab2$I2 <- Insul_paed_add_reg_ethnic[["I2.w"]]

Insul_paed_add_tab2$p_q <- Insul_paed_add_reg_ethnic[["pval.Q.w"]]

Insul_paed_add_tab2$group <- row.names(Insul_paed_add_tab2)

Insul_paed_add_tab2$model <- "Additive"
```

```
Insul_paed_add_tab2$outcome <- "Insul"

Insul_paed_dom_tab <- data.frame(Insul_paed_dom_reg_ethnic[["TE.random"]])
row.names(Insul_paed_dom_tab) <- "Overall"
Insul_paed_dom_tab$MD <- Insul_paed_dom_tab$Insul_paed_dom_reg_ethnic...TE.random...
Insul_paed_dom_tab <- Insul_paed_dom_tab[-c(1)]
Insul_paed_dom_tab$lower <- Insul_paed_dom_reg_ethnic[["lower.random"]]
Insul_paed_dom_tab$upper <- Insul_paed_dom_reg_ethnic[["upper.random"]]
Insul_paed_dom_tab$k <- Insul_paed_dom_reg_ethnic[["k"]]
Insul_paed_dom_tab$p_z <- Insul_paed_dom_reg_ethnic[["pval.random"]]
Insul_paed_dom_tab$I2 <- Insul_paed_dom_reg_ethnic[["I2"]]
Insul_paed_dom_tab$p_q <- Insul_paed_dom_reg_ethnic[["pval.Q"]]
Insul_paed_dom_tab$group <- "Overall"
Insul_paed_dom_tab$model <- "Dominant"
Insul_paed_dom_tab$outcome <- "Insul"

Insul_paed_dom_tab2 <- data.frame(Insul_paed_dom_reg_ethnic[["TE.random.w"]])
row.names(Insul_paed_dom_tab2) <- Insul_paed_dom_reg_ethnic[["bylevs"]]
```

```
Insul_paed_dom_tab2$MD <- Insul_paed_dom_tab2$Insul_paed_dom_reg_ethnic...TE.random.w...

Insul_paed_dom_tab2 <- Insul_paed_dom_tab2[-c(1)]

Insul_paed_dom_tab2$lower <- Insul_paed_dom_reg_ethnic[["lower.random.w"]]

Insul_paed_dom_tab2$upper <- Insul_paed_dom_reg_ethnic[["upper.random.w"]]

Insul_paed_dom_tab2$k <- Insul_paed_dom_reg_ethnic[["k.w"]]

Insul_paed_dom_tab2$p_z <- Insul_paed_dom_reg_ethnic[["pval.random.w"]]

Insul_paed_dom_tab2$I2 <- Insul_paed_dom_reg_ethnic[["I2.w"]]

Insul_paed_dom_tab2$p_q <- Insul_paed_dom_reg_ethnic[["pval.Q.w"]]

Insul_paed_dom_tab2$group <- row.names(Insul_paed_dom_tab2)

Insul_paed_dom_tab2$model <- "Dominant"

Insul_paed_dom_tab2$outcome <- "Insul"


Insul_paed_rec_tab <- data.frame(Insul_paed_rec_reg_ethnic[["TE.random"]])

row.names(Insul_paed_rec_tab) <- "Overall"

Insul_paed_rec_tab$MD <- Insul_paed_rec_tab$Insul_paed_rec_reg_ethnic...TE.random...

Insul_paed_rec_tab <- Insul_paed_rec_tab[-c(1)]

Insul_paed_rec_tab$lower <- Insul_paed_rec_reg_ethnic[["lower.random"]]

Insul_paed_rec_tab$upper <- Insul_paed_rec_reg_ethnic[["upper.random"]]
```

```
Insul_paed_rec_tab$k <- Insul_paed_rec_reg_ethnic[["k"]]

Insul_paed_rec_tab$p_z <- Insul_paed_rec_reg_ethnic[["pval.random"]]

Insul_paed_rec_tab$I2 <- Insul_paed_rec_reg_ethnic[["I2"]]

Insul_paed_rec_tab$p_q <- Insul_paed_rec_reg_ethnic[["pval.Q"]]

Insul_paed_rec_tab$group <- "Overall"

Insul_paed_rec_tab$model <- "Recessive"

Insul_paed_rec_tab$outcome <- "Insul"


Insul_paed_rec_tab2 <- data.frame(Insul_paed_rec_reg_ethnic[["TE.random.w"]])

row.names(Insul_paed_rec_tab2) <- Insul_paed_rec_reg_ethnic[["bylevs"]]

Insul_paed_rec_tab2$MD <- Insul_paed_rec_tab2$Insul_paed_rec_reg_ethnic...TE.random.w...

Insul_paed_rec_tab2 <- Insul_paed_rec_tab2[-c(1)]

Insul_paed_rec_tab2$lower <- Insul_paed_rec_reg_ethnic[["lower.random.w"]]

Insul_paed_rec_tab2$upper <- Insul_paed_rec_reg_ethnic[["upper.random.w"]]

Insul_paed_rec_tab2$k <- Insul_paed_rec_reg_ethnic[["k.w"]]

Insul_paed_rec_tab2$p_z <- Insul_paed_rec_reg_ethnic[["pval.random.w"]]

Insul_paed_rec_tab2$I2 <- Insul_paed_rec_reg_ethnic[["I2.w"]]

Insul_paed_rec_tab2$p_q <- Insul_paed_rec_reg_ethnic[["pval.Q.w"]]
```

```
Insul_paed_rec_tab2$group <- row.names(Insul_paed_rec_tab2)

Insul_paed_rec_tab2$model <- "Recessive"

Insul_paed_rec_tab2$outcome <- "Insul"


Insul_paed_sumtab <- rbind(Insul_paed_add_tab, Insul_paed_add_tab2, Insul_paed_dom_tab, Insul_paed_dom_tab2,

Insul_paed_rec_tab, Insul_paed_rec_tab2)


TG_adult <- read_excel("TG_adult.xlsx")


TG_adult$TG_CCCT_num <- TG_adult$TG_CC_num+TG_adult$TG_CT_num

TG_adult$TG_CCCT_mean <-

((TG_adult$TG_CC_mean*TG_adult$TG_CC_num)+(TG_adult$TG_CT_mean*TG_adult$TG_CT_num))/TG_adult$TG_CCCT_nu

m

TG_adult$TG_CCCT_SD <-

((TG_adult$TG_CC_SD*TG_adult$TG_CC_num)+(TG_adult$TG_CT_SD*TG_adult$TG_CT_num))/TG_adult$TG_CCCT_num
```

```
TG_adult$TG_CTTT_num <- TG_adult$TG_TT_num+TG_adult$TG_CT_num
TG_adult$TG_CTTT_mean <-
((TG_adult$TG_TT_mean*TG_adult$TG_TT_num)+(TG_adult$TG_CT_mean*TG_adult$TG_CT_num))/TG_adult$TG_CTTT_num
TG_adult$TG_CTTT_SD <-
((TG_adult$TG_TT_SD*TG_adult$TG_TT_num)+(TG_adult$TG_CT_SD*TG_adult$TG_CT_num))/TG_adult$TG_CTTT_num

TG_adult_add <- read_excel("TG_adult_add.xlsx")
TG_adult_add_reg <- metagen(Beta, SE, data = TG_adult_add, studlab = TG_adult_add$Paper, sm = "ZCOR", method.tau = "DL")
TG_adult_add_reg_ethnic <- update(TG_adult_add_reg, byvar = Ethnicity, bylab = "Ethnicity")

TG_adult_rec_reg <- metacont(TG_TT_num, TG_TT_mean, TG_TT_SD, TG_CCCT_num, TG_CCCT_mean, TG_CCCT_SD, data
= TG_adult, studlab = paste(Paper), comb.fixed = FALSE, comb.random = TRUE, method.tau = "DL", hakn = TRUE, prediction =
FALSE, sm = "MD")
TG_adult_rec_reg_ethnic <- update(TG_adult_rec_reg, byvar = Ethnicity, bylab = "Ethnicity")

TG_adult_dom_reg <- metacont(TG_CTTT_num, TG_CTTT_mean, TG_CTTT_SD, TG_CC_num, TG_CC_mean, TG_CC_SD,
data = TG_adult, studlab = paste(Paper), comb.fixed = FALSE, comb.random = TRUE, method.tau = "DL", hakn = TRUE,
prediction = FALSE, sm = "MD")
```

```
TG_adult_dom_reg_ethnic <- update(TG_adult_dom_reg, byvar = Ethnicity, bylab = "Ethnicity")


TG_adult_add_tab <- data.frame(TG_adult_add_reg_ethnic[["TE.random"]])

row.names(TG_adult_add_tab) <- "Overall"

TG_adult_add_tab$MD <- TG_adult_add_tab$TG_adult_add_reg_ethnic...TE.random...

TG_adult_add_tab <- TG_adult_add_tab[-c(1)]

TG_adult_add_tab$lower <- TG_adult_add_reg_ethnic[["lower.random"]]

TG_adult_add_tab$upper <- TG_adult_add_reg_ethnic[["upper.random"]]

TG_adult_add_tab$k <- TG_adult_add_reg_ethnic[["k"]]

TG_adult_add_tab$p_z <- TG_adult_add_reg_ethnic[["pval.random"]]

TG_adult_add_tab$I2 <- TG_adult_add_reg_ethnic[["I2"]]

TG_adult_add_tab$p_q <- TG_adult_add_reg_ethnic[["pval.Q"]]

TG_adult_add_tab$group <- "Overall"

TG_adult_add_tab$model <- "Additive"

TG_adult_add_tab$outcome <- "TG"


TG_adult_add_tab2 <- data.frame(TG_adult_add_reg_ethnic[["TE.random.w"]])

row.names(TG_adult_add_tab2) <- TG_adult_add_reg_ethnic[["bylevs"]]
```

```
TG_adult_add_tab2$MD <- TG_adult_add_tab2$TG_adult_add_reg_ethnic...TE.random.w...

TG_adult_add_tab2 <- TG_adult_add_tab2[-c(1)]

TG_adult_add_tab2$lower <- TG_adult_add_reg_ethnic[["lower.random.w"]]

TG_adult_add_tab2$upper <- TG_adult_add_reg_ethnic[["upper.random.w"]]

TG_adult_add_tab2$k <- TG_adult_add_reg_ethnic[["k.w"]]

TG_adult_add_tab2$p_z <- TG_adult_add_reg_ethnic[["pval.random.w"]]

TG_adult_add_tab2$I2 <- TG_adult_add_reg_ethnic[["I2.w"]]

TG_adult_add_tab2$p_q <- TG_adult_add_reg_ethnic[["pval.Q.w"]]

TG_adult_add_tab2$group <- row.names(TG_adult_add_tab2)

TG_adult_add_tab2$model <- "Additive"

TG_adult_add_tab2$outcome <- "TG"


TG_adult_dom_tab <- data.frame(TG_adult_dom_reg_ethnic[["TE.random"]])

row.names(TG_adult_dom_tab) <- "Overall"

TG_adult_dom_tab$MD <- TG_adult_dom_tab$TG_adult_dom_reg_ethnic...TE.random...

TG_adult_dom_tab <- TG_adult_dom_tab[-c(1)]

TG_adult_dom_tab$lower <- TG_adult_dom_reg_ethnic[["lower.random"]]

TG_adult_dom_tab$upper <- TG_adult_dom_reg_ethnic[["upper.random"]]
```

```
TG_adult_dom_tab$k <- TG_adult_dom_reg_ethnic[["k"]]

TG_adult_dom_tab$p_z <- TG_adult_dom_reg_ethnic[["pval.random"]]

TG_adult_dom_tab$I2 <- TG_adult_dom_reg_ethnic[["I2"]]

TG_adult_dom_tab$p_q <- TG_adult_dom_reg_ethnic[["pval.Q"]]

TG_adult_dom_tab$group <- "Overall"

TG_adult_dom_tab$model <- "Dominant"

TG_adult_dom_tab$outcome <- "TG"


TG_adult_dom_tab2 <- data.frame(TG_adult_dom_reg_ethnic[["TE.random.w"]])

row.names(TG_adult_dom_tab2) <- TG_adult_dom_reg_ethnic[["bylevs"]]

TG_adult_dom_tab2$MD <- TG_adult_dom_tab2$TG_adult_dom_reg_ethnic...TE.random.w...

TG_adult_dom_tab2 <- TG_adult_dom_tab2[-c(1)]

TG_adult_dom_tab2$lower <- TG_adult_dom_reg_ethnic[["lower.random.w"]]

TG_adult_dom_tab2$upper <- TG_adult_dom_reg_ethnic[["upper.random.w"]]

TG_adult_dom_tab2$k <- TG_adult_dom_reg_ethnic[["k.w"]]

TG_adult_dom_tab2$p_z <- TG_adult_dom_reg_ethnic[["pval.random.w"]]

TG_adult_dom_tab2$I2 <- TG_adult_dom_reg_ethnic[["I2.w"]]

TG_adult_dom_tab2$p_q <- TG_adult_dom_reg_ethnic[["pval.Q.w"]]
```

```
TG_adult_dom_tab2$group <- row.names(TG_adult_dom_tab2)

TG_adult_dom_tab2$model <- "Dominant"

TG_adult_dom_tab2$outcome <- "TG"


TG_adult_rec_tab <- data.frame(TG_adult_rec_reg_ethnic[["TE.random"]])

row.names(TG_adult_rec_tab) <- "Overall"

TG_adult_rec_tab$MD <- TG_adult_rec_tab$TG_adult_rec_reg_ethnic...TE.random...

TG_adult_rec_tab <- TG_adult_rec_tab[-c(1)]

TG_adult_rec_tab$lower <- TG_adult_rec_reg_ethnic[["lower.random"]]

TG_adult_rec_tab$upper <- TG_adult_rec_reg_ethnic[["upper.random"]]

TG_adult_rec_tab$k <- TG_adult_rec_reg_ethnic[["k"]]

TG_adult_rec_tab$p_z <- TG_adult_rec_reg_ethnic[["pval.random"]]

TG_adult_rec_tab$I2 <- TG_adult_rec_reg_ethnic[["I2"]]

TG_adult_rec_tab$p_q <- TG_adult_rec_reg_ethnic[["pval.Q"]]

TG_adult_rec_tab$group <- "Overall"

TG_adult_rec_tab$model <- "Recessive"

TG_adult_rec_tab$outcome <- "TG"
```

```
TG_adult_rec_tab2 <- data.frame(TG_adult_rec_reg_ethnic[["TE.random.w"]])

row.names(TG_adult_rec_tab2) <- TG_adult_rec_reg_ethnic[["bylevs"]]

TG_adult_rec_tab2$MD <- TG_adult_rec_tab2$TG_adult_rec_reg_ethnic...TE.random.w...

TG_adult_rec_tab2 <- TG_adult_rec_tab2[-c(1)]

TG_adult_rec_tab2$lower <- TG_adult_rec_reg_ethnic[["lower.random.w"]]

TG_adult_rec_tab2$upper <- TG_adult_rec_reg_ethnic[["upper.random.w"]]

TG_adult_rec_tab2$k <- TG_adult_rec_reg_ethnic[["k.w"]]

TG_adult_rec_tab2$p_z <- TG_adult_rec_reg_ethnic[["pval.random.w"]]

TG_adult_rec_tab2$I2 <- TG_adult_rec_reg_ethnic[["I2.w"]]

TG_adult_rec_tab2$p_q <- TG_adult_rec_reg_ethnic[["pval.Q.w"]]

TG_adult_rec_tab2$group <- row.names(TG_adult_rec_tab2)

TG_adult_rec_tab2$model <- "Recessive"

TG_adult_rec_tab2$outcome <- "TG"


TG_adult_sumtab <- rbind(TG_adult_add_tab, TG_adult_add_tab2, TG_adult_dom_tab, TG_adult_dom_tab2, TG_adult_rec_tab,

TG_adult_rec_tab2)
```

```
TG_paed <- read_excel("TG_paed.xlsx")

TG_paed$TG_CCCT_num <- TG_paed$TG_CC_num+TG_paed$TG_CT_num
TG_paed$TG_CCCT_mean <-
((TG_paed$TG_CC_mean*TG_paed$TG_CC_num)+(TG_paed$TG_CT_mean*TG_paed$TG_CT_num))/TG_paed$TG_CCCT_nu
m
TG_paed$TG_CCCT_SD <-
((TG_paed$TG_CC_SD*TG_paed$TG_CC_num)+(TG_paed$TG_CT_SD*TG_paed$TG_CT_num))/TG_paed$TG_CCCT_num
TG_paed$TG_CTTT_num <- TG_paed$TG_TT_num+TG_paed$TG_CT_num
TG_paed$TG_CTTT_mean <-
((TG_paed$TG_TT_mean*TG_paed$TG_TT_num)+(TG_paed$TG_CT_mean*TG_paed$TG_CT_num))/TG_paed$TG_CTTT_nu
m
TG_paed$TG_CTTT_SD <-
((TG_paed$TG_TT_SD*TG_paed$TG_TT_num)+(TG_paed$TG_CT_SD*TG_paed$TG_CT_num))/TG_paed$TG_CTTT_num

TG_paed_add <- read_excel("TG_paed_add.xlsx")
```

```
TG_paed_add_reg <- metagen(Beta, SE, data = TG_paed_add, studlab = TG_paed_add$Paper, sm = "ZCOR", method.tau =
"DL")
TG_paed_add_reg_ethnic <- update(TG_paed_add_reg, byvar = Ethnicity, bylab = "Ethnicity")

TG_paed_rec_reg <- metacont(TG_TT_num, TG_TT_mean, TG_TT_SD, TG_CCCT_num, TG_CCCT_mean, TG_CCCT_SD, data
= TG_paed, studlab = paste(Paper), comb.fixed = FALSE, comb.random = TRUE, method.tau = "DL", hakn = TRUE, prediction =
FALSE, sm = "MD")
TG_paed_rec_reg_ethnic <- update(TG_paed_rec_reg, byvar = Ethnicity, bylab = "Ethnicity")

TG_paed_dom_reg <- metacont(TG_CTTT_num, TG_CTTT_mean, TG_CTTT_SD, TG_CC_num, TG_CC_mean, TG_CC_SD,
data = TG_paed, studlab = paste(Paper), comb.fixed = FALSE, comb.random = TRUE, method.tau = "DL", hakn = TRUE,
prediction = FALSE, sm = "MD")
TG_paed_dom_reg_ethnic <- update(TG_paed_dom_reg, byvar = Ethnicity, bylab = "Ethnicity")

TG_paed_add_tab <- data.frame(TG_paed_add_reg_ethnic[["TE.random"]])
row.names(TG_paed_add_tab) <- "Overall"
TG_paed_add_tab$MD <- TG_paed_add_tab$TG_paed_add_reg_ethnic...TE.random...
TG_paed_add_tab <- TG_paed_add_tab[-c(1)]
```

```
TG_paed_add_tab$lower <- TG_paed_add_reg_ethnic[["lower.random"]]

TG_paed_add_tab$upper <- TG_paed_add_reg_ethnic[["upper.random"]]

TG_paed_add_tab$k <- TG_paed_add_reg_ethnic[["k"]]

TG_paed_add_tab$p_z <- TG_paed_add_reg_ethnic[["pval.random"]]

TG_paed_add_tab$I2 <- TG_paed_add_reg_ethnic[["I2"]]

TG_paed_add_tab$p_q <- TG_paed_add_reg_ethnic[["pval.Q"]]

TG_paed_add_tab$group <- "Overall"

TG_paed_add_tab$model <- "Additive"

TG_paed_add_tab$outcome <- "TG"


TG_paed_add_tab2 <- data.frame(TG_paed_add_reg_ethnic[["TE.random.w"]])

row.names(TG_paed_add_tab2) <- TG_paed_add_reg_ethnic[["bylevs"]]

TG_paed_add_tab2$MD <- TG_paed_add_tab2$TG_paed_add_reg_ethnic...TE.random.w...

TG_paed_add_tab2 <- TG_paed_add_tab2[-c(1)]

TG_paed_add_tab2$lower <- TG_paed_add_reg_ethnic[["lower.random.w"]]

TG_paed_add_tab2$upper <- TG_paed_add_reg_ethnic[["upper.random.w"]]

TG_paed_add_tab2$k <- TG_paed_add_reg_ethnic[["k.w"]]

TG_paed_add_tab2$p_z <- TG_paed_add_reg_ethnic[["pval.random.w"]]
```

```
TG_paed_add_tab2$I2 <- TG_paed_add_reg_ethnic[["I2.w"]]

TG_paed_add_tab2$p_q <- TG_paed_add_reg_ethnic[["pval.Q.w"]]

TG_paed_add_tab2$group <- row.names(TG_paed_add_tab2)

TG_paed_add_tab2$model <- "Additive"

TG_paed_add_tab2$outcome <- "TG"


TG_paed_dom_tab <- data.frame(TG_paed_dom_reg_ethnic[["TE.random"]])

row.names(TG_paed_dom_tab) <- "Overall"

TG_paed_dom_tab$MD <- TG_paed_dom_tab$TG_paed_dom_reg_ethnic...TE.random...

TG_paed_dom_tab <- TG_paed_dom_tab[-c(1)]

TG_paed_dom_tab$lower <- TG_paed_dom_reg_ethnic[["lower.random"]]

TG_paed_dom_tab$upper <- TG_paed_dom_reg_ethnic[["upper.random"]]

TG_paed_dom_tab$k <- TG_paed_dom_reg_ethnic[["k"]]

TG_paed_dom_tab$p_z <- TG_paed_dom_reg_ethnic[["pval.random"]]

TG_paed_dom_tab$I2 <- TG_paed_dom_reg_ethnic[["I2"]]

TG_paed_dom_tab$p_q <- TG_paed_dom_reg_ethnic[["pval.Q"]]

TG_paed_dom_tab$group <- "Overall"

TG_paed_dom_tab$model <- "Dominant"
```

```
TG_paed_dom_tab$outcome <- "TG"


TG_paed_dom_tab2 <- data.frame(TG_paed_dom_reg_ethnic[["TE.random.w"]])
row.names(TG_paed_dom_tab2) <- TG_paed_dom_reg_ethnic[["bylevs"]]
TG_paed_dom_tab2$MD <- TG_paed_dom_tab2$TG_paed_dom_reg_ethnic...TE.random.w...
TG_paed_dom_tab2 <- TG_paed_dom_tab2[-c(1)]
TG_paed_dom_tab2$lower <- TG_paed_dom_reg_ethnic[["lower.random.w"]]
TG_paed_dom_tab2$upper <- TG_paed_dom_reg_ethnic[["upper.random.w"]]
TG_paed_dom_tab2$k <- TG_paed_dom_reg_ethnic[["k.w"]]
TG_paed_dom_tab2$p_z <- TG_paed_dom_reg_ethnic[["pval.random.w"]]
TG_paed_dom_tab2$I2 <- TG_paed_dom_reg_ethnic[["I2.w"]]
TG_paed_dom_tab2$p_q <- TG_paed_dom_reg_ethnic[["pval.Q.w"]]
TG_paed_dom_tab2$group <- row.names(TG_paed_dom_tab2)
TG_paed_dom_tab2$model <- "Dominant"
TG_paed_dom_tab2$outcome <- "TG"


TG_paed_rec_tab <- data.frame(TG_paed_rec_reg_ethnic[["TE.random"]])
row.names(TG_paed_rec_tab) <- "Overall"
```

```
TG_paed_rec_tab$MD <- TG_paed_rec_tab$TG_paed_rec_reg_ethnic...TE.random...

TG_paed_rec_tab <- TG_paed_rec_tab[-c(1)]

TG_paed_rec_tab$lower <- TG_paed_rec_reg_ethnic[["lower.random"]]

TG_paed_rec_tab$upper <- TG_paed_rec_reg_ethnic[["upper.random"]]

TG_paed_rec_tab$k <- TG_paed_rec_reg_ethnic[["k"]]

TG_paed_rec_tab$p_z <- TG_paed_rec_reg_ethnic[["pval.random"]]

TG_paed_rec_tab$I2 <- TG_paed_rec_reg_ethnic[["I2"]]

TG_paed_rec_tab$p_q <- TG_paed_rec_reg_ethnic[["pval.Q"]]

TG_paed_rec_tab$group <- "Overall"

TG_paed_rec_tab$model <- "Recessive"

TG_paed_rec_tab$outcome <- "TG"


TG_paed_rec_tab2 <- data.frame(TG_paed_rec_reg_ethnic[["TE.random.w"]])

row.names(TG_paed_rec_tab2) <- TG_paed_rec_reg_ethnic[["bylevs"]]

TG_paed_rec_tab2$MD <- TG_paed_rec_tab2$TG_paed_rec_reg_ethnic...TE.random.w...

TG_paed_rec_tab2 <- TG_paed_rec_tab2[-c(1)]

TG_paed_rec_tab2$lower <- TG_paed_rec_reg_ethnic[["lower.random.w"]]

TG_paed_rec_tab2$upper <- TG_paed_rec_reg_ethnic[["upper.random.w"]]
```

```
TG_paed_rec_tab2$k <- TG_paed_rec_reg_ethnic[["k.w"]]

TG_paed_rec_tab2$p_z <- TG_paed_rec_reg_ethnic[["pval.random.w"]]

TG_paed_rec_tab2$I2 <- TG_paed_rec_reg_ethnic[["I2.w"]]

TG_paed_rec_tab2$p_q <- TG_paed_rec_reg_ethnic[["pval.Q.w"]]

TG_paed_rec_tab2$group <- row.names(TG_paed_rec_tab2)

TG_paed_rec_tab2$model <- "Recessive"

TG_paed_rec_tab2$outcome <- "TG"


TG_paed_sumtab <- rbind(TG_paed_add_tab, TG_paed_add_tab2, TG_paed_dom_tab, TG_paed_dom_tab2, TG_paed_rec_tab,
TG_paed_rec_tab2)


Biochem_paed_sumtab <- rbind(TG_paed_sumtab, ALT_paed_sumtab, HDL_paed_sumtab, LDL_paed_sumtab,
Insul_paed_sumtab, Chol_paed_sumtab)

write.table(Biochem_paed_sumtab, file="Biochem_paed_sumtab.csv",sep=",")


Biochem_adult_sumtab <- rbind(TG_adult_sumtab, ALT_adult_sumtab, HDL_adult_sumtab, LDL_adult_sumtab,
Insul_adult_sumtab, Chol_adult_sumtab)

write.table(Biochem_adult_sumtab, file="Biochem_adult_sumtab.csv",sep=",")
```

CTAT methods

Tables for a "Complete, Transparent, Accurate and Timely account" (CTAT) are now mandatory for all revised submissions. The aim is to enhance the reproducibility of methods.

- Only include the parts relevant to your study
- Refer to the CTAT in the main text as 'Supplementary CTAT Table'
- Do not add subheadings
- Add as many rows as needed to include all information
- Only include one item per row

If the CTAT form is not relevant to your study, please outline the reasons why:

1.1 Antibodies

Name	Citation	Supplier	Cat no.	Clone no.

1.2 Cell lines

Name	Citation	Supplier	Cat no.	Passage no.	Authentication test method

1.3 Organisms

Name	Citation	Supplier	Strain	Sex	Age	Overall n number

1.4 Sequence based reagents

Name	Sequence	Supplier

1.5 Biological samples

Description	Source	Identifier

1.6 Deposited data

Name of repository	Identifier	Link

1.7 Software

Software name	Manufacturer	Version
**R**	**The R Foundation for Statistical Computing**	3.6.1
**STATA**	StataCorp	**v14**

1.8 Other (e.g. drugs, proteins, vectors etc.)

1.9 Please provide the details of the corresponding methods author for the manuscript:

Dr Jake P. Mann, University of Cambridge, Institute of Metabolic Science, Addenbrooke's Hospital, Cambridge, United Kingdom. jm2032@cam.ac.uk, Tel: +44 1223 336792

2.0 Please confirm for randomised controlled trials all versions of the clinical protocol are included in the submission. These will be published online as supplementary information.

