# Supplementary material for: rs641738C>T near MBOAT7 is associated with liver fat, ALT and fibrosis in NAFLD: A meta-analysis
Source: J Hepatol. 2021 Jan;74(1):20–30. doi: 10.1016/j.jhep.2020.08.027 (PMC7755037; doi:10.1016/j.jhep.2020.08.027)
Supplement: CTAT table.pdf [file mmc2.pdf]

JOURNAL
OF HEPATOLOGY

Journal of Hepatology

CTAT methods

Tables for a "Complete, Transparent, Accurate and Timely account" (CTAT) are now mandatory for all revised submissions. The aim is to enhance the reproducibility of methods.

- Only include the parts relevant to your study
- Refer to the CTAT in the main text as 'Supplementary CTAT Table'
- Do not add subheadings
- Add as many rows as needed to include all information
- Only include one item per row

If the CTAT form is not relevant to your study, please outline the reasons why:

1.1 Antibodies

Name	Citation	Supplier	Cat no.	Clone no.

1.2 Cell lines

Name	Citation	Supplier	Cat no.	Passage no.	Authentication test method

1.3 Organisms

Name	Citation	Supplier	Strain	Sex	Age	Overall n number

1.4 Sequence based reagents

Name	Sequence	Supplier

1.5 Biological samples

Description	Source	Identifier

1.6 Deposited data

Name of repository	Identifier	Link

1.7 Software

Software name	Manufacturer	Version
R	The R Foundation for Statistical Computing	3.6.1
STATA	StataCorp	v14

1.8 Other (e.g. drugs, proteins, vectors etc.)

1.9 Please provide the details of the corresponding methods author for the manuscript:

Dr Jake P. Mann, University of Cambridge, Institute of Metabolic Science, Addenbrooke's Hospital, Cambridge, United Kingdom. jm2032@cam.ac.uk, Tel: +44 1223 336792

2.0 Please confirm for randomised controlled trials all versions of the clinical protocol are included in the submission. These will be published online as supplementary information.
